# Structural effects of meso-halogenation on porphyrins

**DOI:** 10.3762/bjoc.17.88

**Published:** 2021-05-14

**Authors:** Keith J Flanagan, Maximilian Paradiz Dominguez, Zoi Melissari, Hans-Georg Eckhardt, René M Williams, Dáire Gibbons, Caroline Prior, Gemma M Locke, Alina Meindl, Aoife A Ryan, Mathias O Senge

**Affiliations:** 1 School of Chemistry, Chair of Organic Chemistry, Trinity Biomedical Sciences Institute, 152–160 Pearse Street, Trinity College Dublin, The University of Dublin, Dublin 2, Ireland; 2 Van ‘t Hoff Institute for Molecular Sciences, University of Amsterdam, P.O. Box 94157, 1090 GD Amsterdam, The Netherlands

**Keywords:** conformational analysis, crystal engineering, halogenation, macrocycles, porphyrins

## Abstract

The use of halogens in the crystal engineering of supramolecular porphyrin assemblies has been a topic of strong interest over the past decades. With this in mind we have characterized a series of direct meso-halogenated porphyrins using single crystal X-ray crystallography. This is accompanied by a detailed conformational analysis of all deposited meso-halogenated porphyrins in the CSD. In this study we have used the Hirshfeld fingerprint plots together with normal-coordinate structural decomposition and determined crystal structures to elucidate the conformation, present intermolecular interactions, and compare respective contacts within the crystalline architectures. Additionally, we have used density functional theory calculations to determine the structure of several halogenated porphyrins. This contrasts conformational analysis with existing X-ray structures and gives a method to characterize samples that are difficult to crystallize. By using the methods outlined above we were able to deduce the impact a meso halogen has on a porphyrin, for example, meso-halogenation is dependent on the type of alternate substituents present when forming supramolecular assemblies. Furthermore, we have designed a method to predict the conformation of halogenated porphyrins, without need of crystallization, using DFT calculations with a high degree of accuracy.

## Introduction

Crystal engineering using porphyrins as a scaffolding unit has been a topic of increasing interest over the past few decades. Byrn et al. were the first to observe that porphyrins have a high propensity to form “porous” clathrates and suggested that they can be used as a “porphyrin sponge” [1]. They reported that 5,10,15,20-tetraphenylporphyrin could act like a ‘host’ which could trap a variety of solvent ‘guests’ within the crystal lattice, through hydrogen-bonding and van der Waals forces. Thereafter, research over the crystal engineering of porphyrins has focused on the noncovalent interactions, such as hydrogen bonds and halogen bonds, or metal coordination interactions [2–13]. Such complexes have been reported for potential application in materials sciences (e.g., molecular sieves) as they form a three-dimensional lattice where more than 50% of the crystal capacity consists of open straight channels [5–6]. Noteworthy, Titi et al. reported that a chiral architecture based on C–I···N and C–I···π interactions was formed through halogen bonding in the lattice of self-assembly of meso-tetraarylporphyrins [7].

In recent years there has been a strong uprising interest for substituting hydrogen-bonding motifs with their halogen-bonding counterparts. This is due to their relative versatility in areas such as directionality, the tunability of the σ-hole, hydrophobicity, and donor atom size [14]. These traits allow for the design of novel supramolecular architectures which are directive and reproducible, without relying on the hydrogen-bonding functionality which in some cases can be undesirable. Recent studies from our group have been carried out on the use of halogens as a binding motif in cubanes [15], bicyclo[1.1.1]pentane [16], and nonplanar porphyrins [17]. Another reason to investigate the effect of meso-halogenation of porphyrins is due to the potential of induced distortion of the macrocyclic core. Due to their relatively large size and high electronegativity, halogens induce a conformational distortion of the porphyrin core that is significant compared to those induced by other substitution groups. As recently illustrated in a review by Kielmann and Senge [18], the conformation of the porphyrin core can play a key role in the binding of small molecules or on its efficiency as an organocatalyst as demonstrated by Roucan et al. [19]. With the continuing interest in nonplanar porphyrins [20] and their relevance for the in vivo functioning of porphyrin cofactors [21], there is a significant importance of the knowledge of the conformation when designing supramolecular materials.

Studies on the effect of meso-halogenation have focused on the use of crystal data as a means of characterization [22], discriminating between enantiomers [23], or as a self-contained structural discussion [24]. However, with all the advances made in the crystal engineering of planar porphyrins, investigation through exploration into the effect of meso-halogenation on the macrocycle architecture have, to date, not been carried out. Herein we present a comparative analysis of the crystallographic structural characteristics of 13 meso*_x_*-halo-substituted porphyrins. Data obtained from the CSD crystal structure database accompanied by data obtained from our group are discussed [25]. Furthermore, any general structural motif that appears consistent among the meso*_x_*-halo-substituted porphyrins were investigated. Hirshfeld fingerprints [26] were obtained to elucidate the intermolecular interactions and compare the respective contacts. Hirshfeld surface analysis (HSA) displayed that H···H, X···C, and X···H (where X is a halogen atom) interactions vastly contribute to the formation of the crystal networks. This is complemented with the use of density functional theory (DFT) calculation of structures that have currently not been determined due to difficulties of obtained crystals of sufficient quality for X-ray determination. Additionally, DFT calculations were conducted for further exploration of the ground state geometries and concurrently frontier molecular orbitals (FMOs) and sigma-holes have been displayed.

## Results and Discussion

### Crystal structure analyses

#### 5-Halo-substituted porphyrins

**Figure 1:**
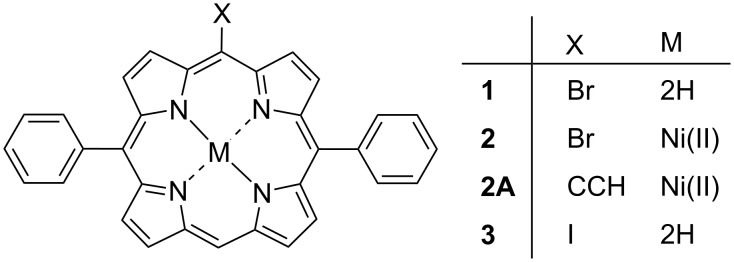
5-Halo-substituted porphyrins.

First, considering the 5-halo-10,20-diphenylporphyrins (Figure 1); in this series, we obtained structures of the free base bromo derivative **1** and discuss the nickel(II) bromo derivative **2** [27] and the free base iodo derivative **3** [22] from the CSD [25]. Additionally, we have included the substituted derivative of **2** with an acetylene moiety (**2A**) to investigate the differences observed between alternative substitution types. The labelled displacement ellipsoid plot and packing diagrams for compounds **1** and **2A** are available in Supporting Information File 1 (Figures S1–S4).

**Figure 2:**
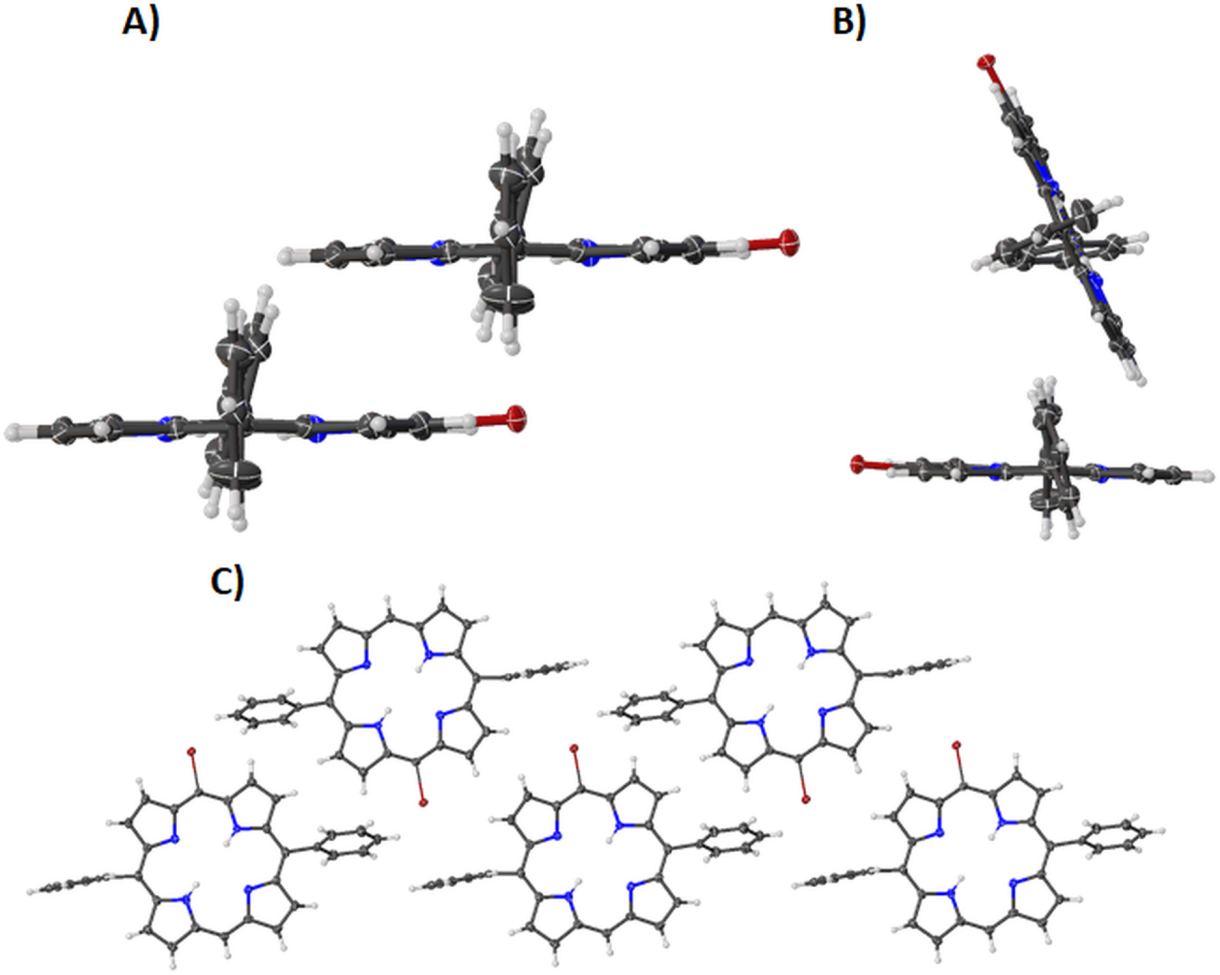
Expanded view (thermal ellipsoid) of compound **1** in the crystal showing (A) stacking, (B) tilted edge-on interaction and (C) layers of porphyrin with ↑↓↑↓ repeating pattern.

The first feature which is of interest is the crystal packing of each of the 5-halo-substituted compounds which appears to be affected by both halogen type and metal insertion. In compound **1** the main motifs seen are the face-to-face stacking at 3.326(4) Å (Figure 2A) and the tilted edge-on interaction (≈64°) (Figure 2B). The other motif seen is the inline interactions where layers of porphyrins are lined up with the bromine atoms pointing towards the phenyl rings in a ↑↓↑↓ repeating pattern (Figure 2C). However, in this structure, there is no evidence for halogen bonding.

**Figure 3:**
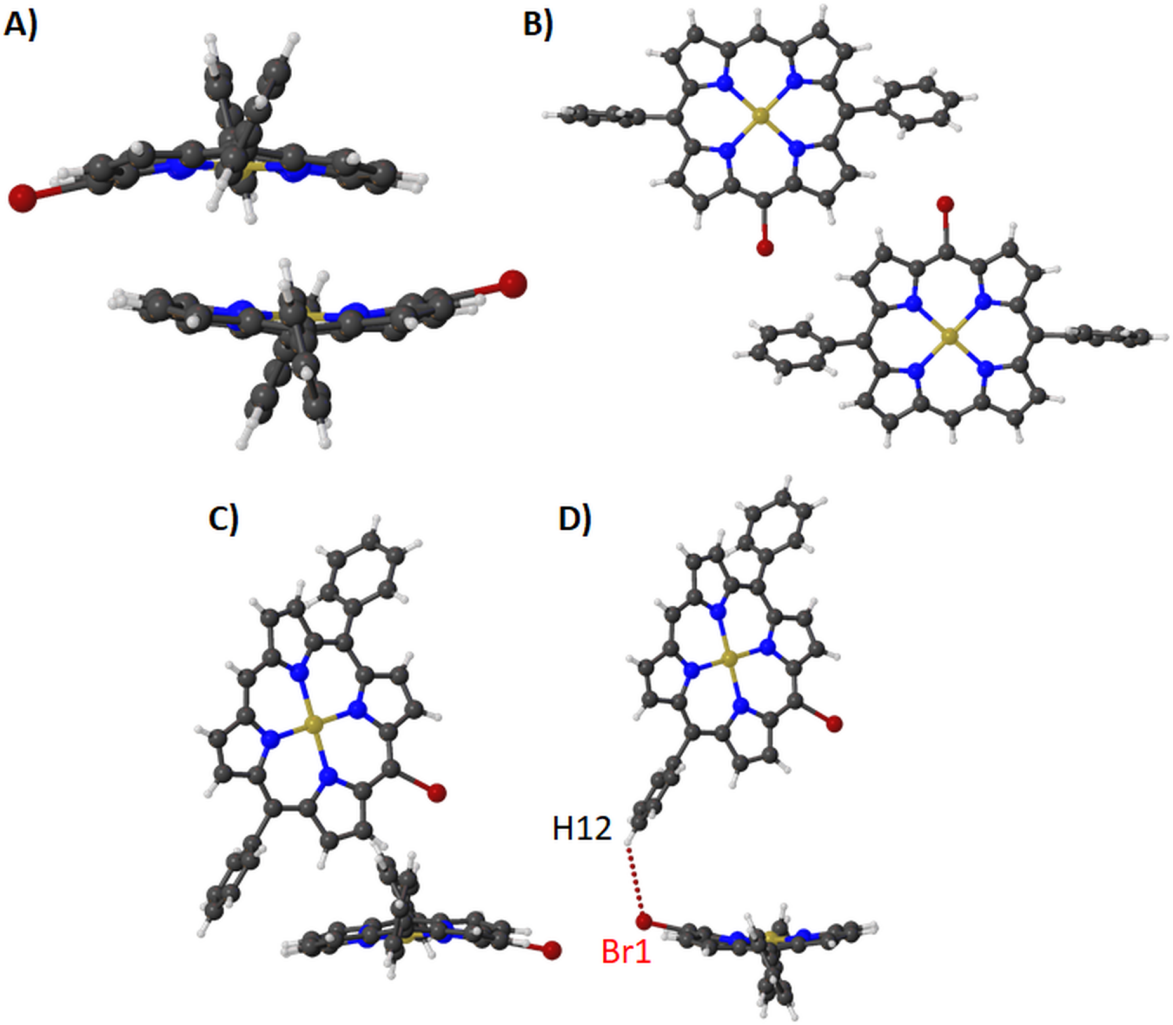
Expanded view (ball and stick) of compound **2** in the crystal showing (A) stacking, (B) bromine atoms pointing towards the phenyl rings, (C) the edge-on interaction between the porphyrin macrocycles and (D) the H···Br interaction between the porphyrin macrocycles.

By the addition of a nickel(II) metal center to compound **1**, we obtain the structure of compound **2** [27]. In this structure a variety of changes in the overall porphyrin conformation are observed due to the metal center insertion. The first of these is the stacking between the porphyrin rings (at 3.581(3) Å), which now features the bromine atoms pointing in opposite directions with a ruffled conformation of the porphyrin macrocycle (Figure 3A). Moreover, a similar motif of bromine atoms pointing towards the phenyl rings is visible in this structure and seems to be a staple of this series (Figure 3B). Secondly, the edge-on interaction in this structure has changed, where the pyrrole moiety is pointing towards the face of the porphyrin macrocycle as seen in Figure 3C. This is accompanied by a Br···H short contact [Br1···H12–C24; 3.291 (1) Å, 137.4 (7)°] (Figure 3D) which combines to form the packing seen in Supporting Information File 1, Figure S5.

**Figure 4:**
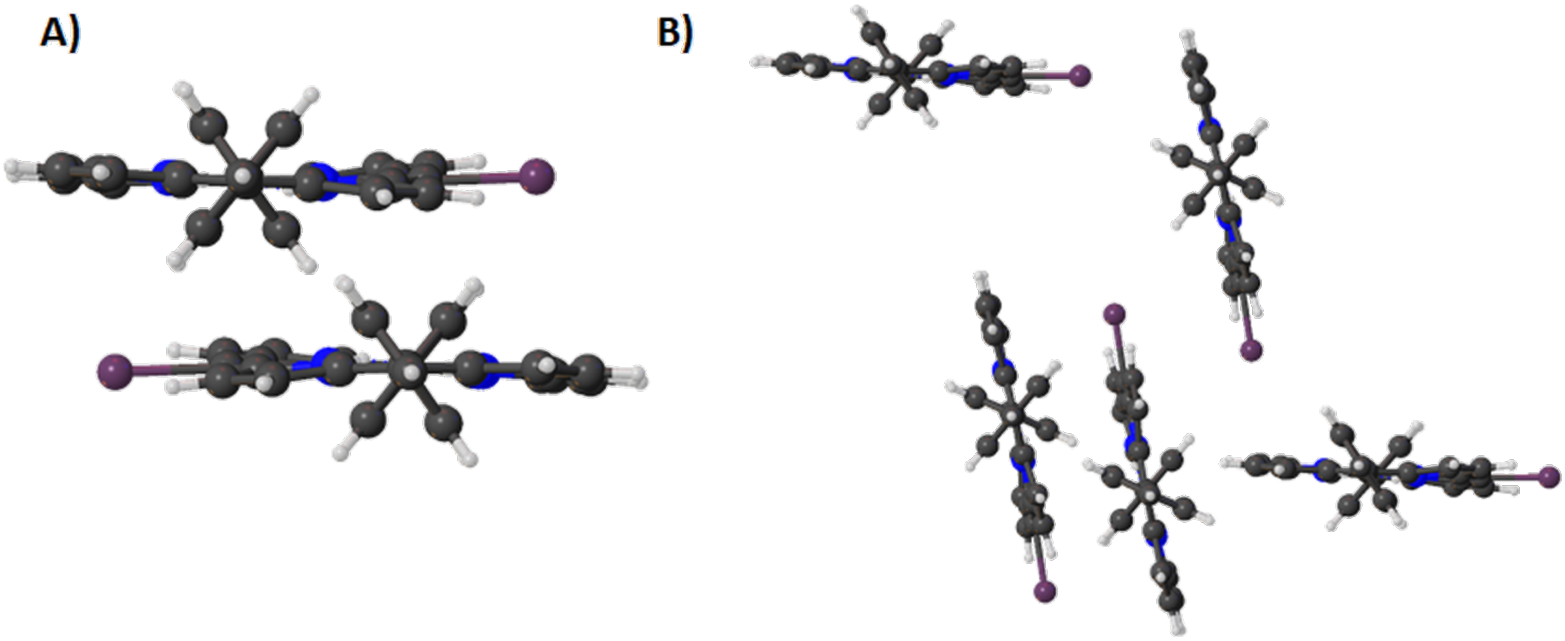
Expanded view (ball and stick) of compound **3** in the crystal showing (A) stacking and (B) edge-on interactions.

In compound **3** the packing of these structural archetypes is changed [22]. The stacking between macrocycle planes is similar to that of **2** (at 3.569(2) Å) (Figure 4A). Additionally, the edge-on interactions show the same tilted motif seen in compound **1**.

**Figure 5:**
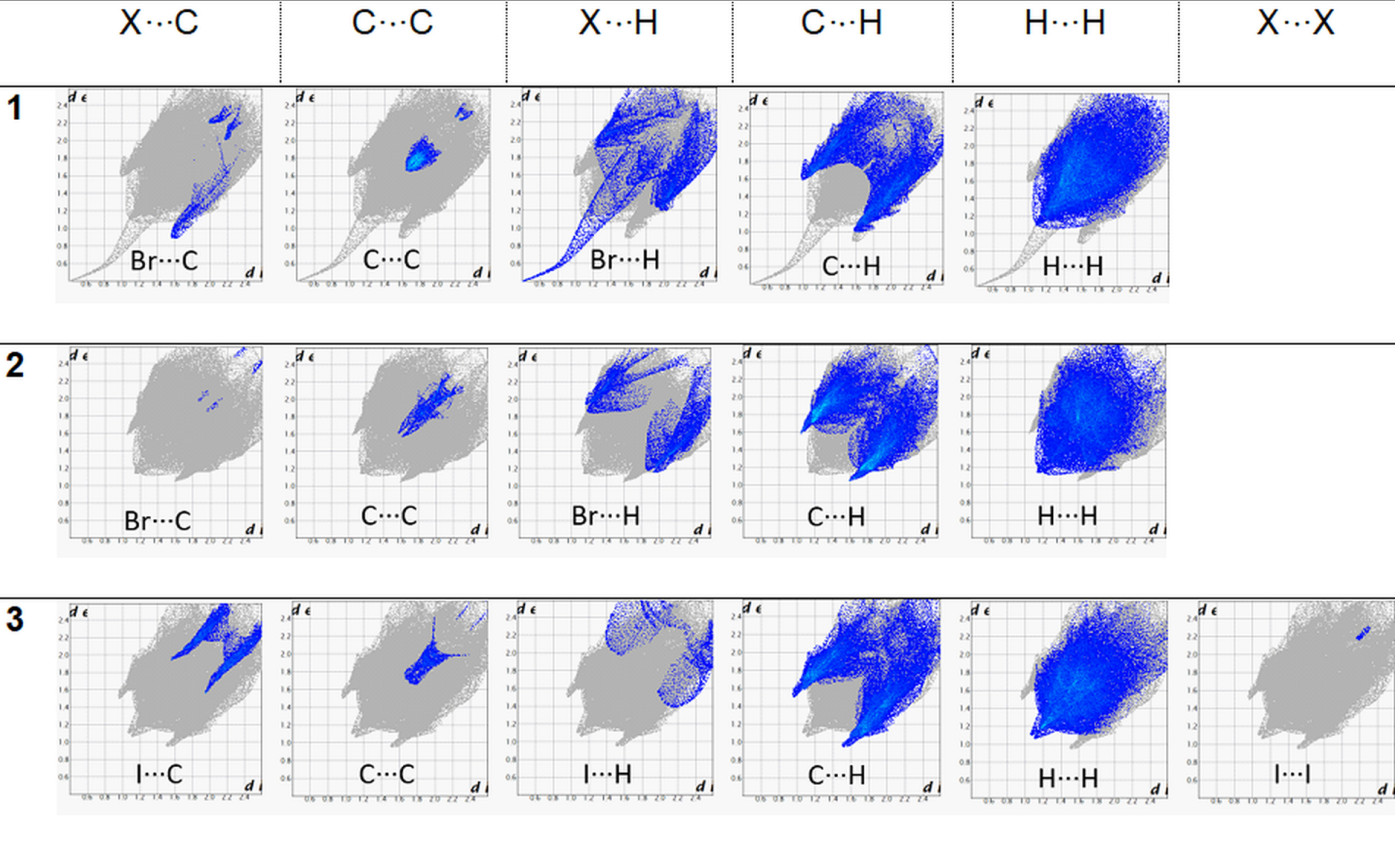
Hirshfeld surfaces of compounds **1**–**3**.

**Figure 6:**
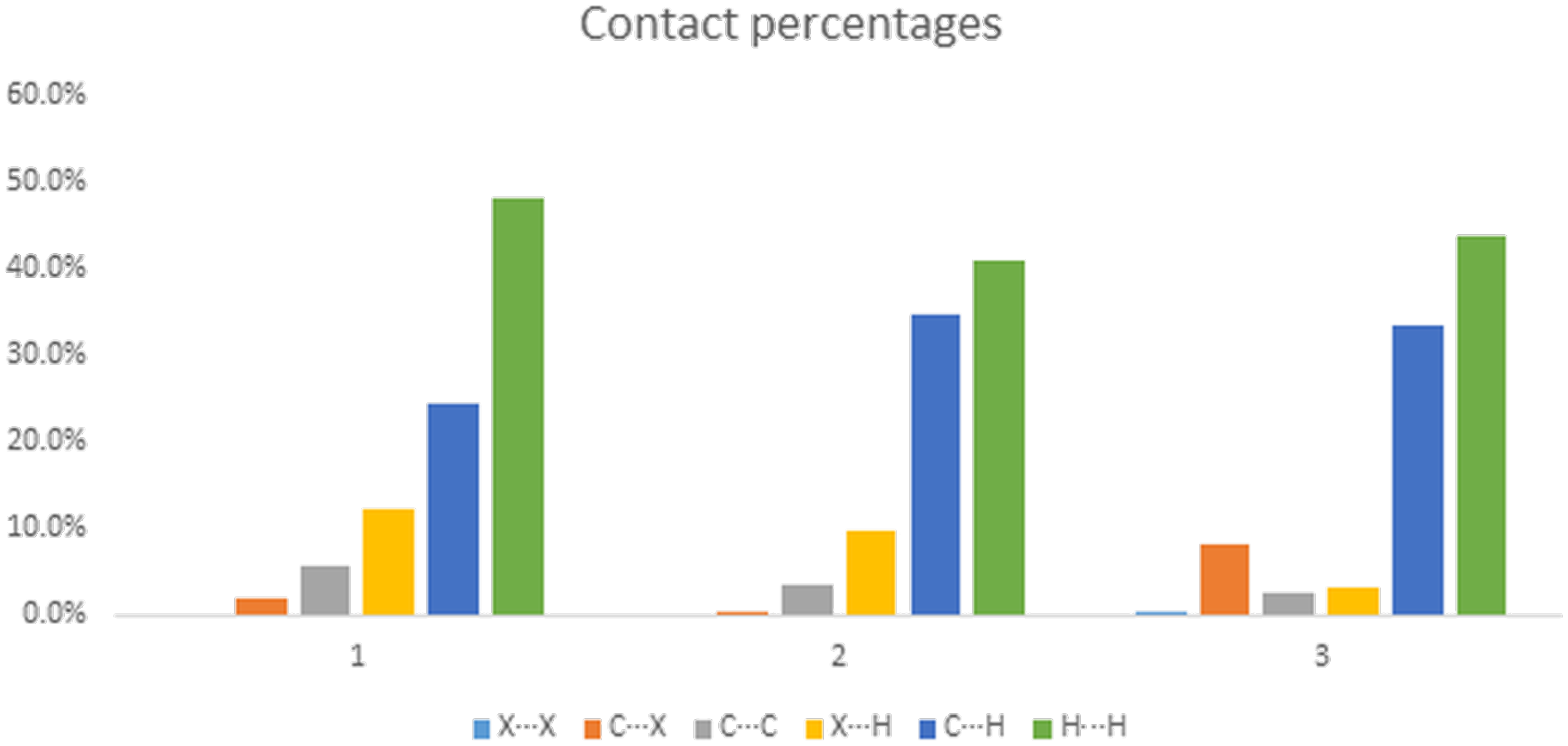
Contact percentages of compounds **1**–**3**.

However, this is combined with a new halogen directed close packing between the iodine atom and the edge of the porphyrin ring in a repeating step-wise pattern which is not seen in the other two structures (Figure 4B). While this may be difficult to visualize in the crystal packing (Supporting Information File 1, Figure S6), it is clear from the Hirshfeld surface analysis that there are marked decreases in the X···H contacts and an increase in the X···C (where X is a halogen atom) between compounds **1** and **3** (Figure 5 and Figure 6).

**Figure 7:**
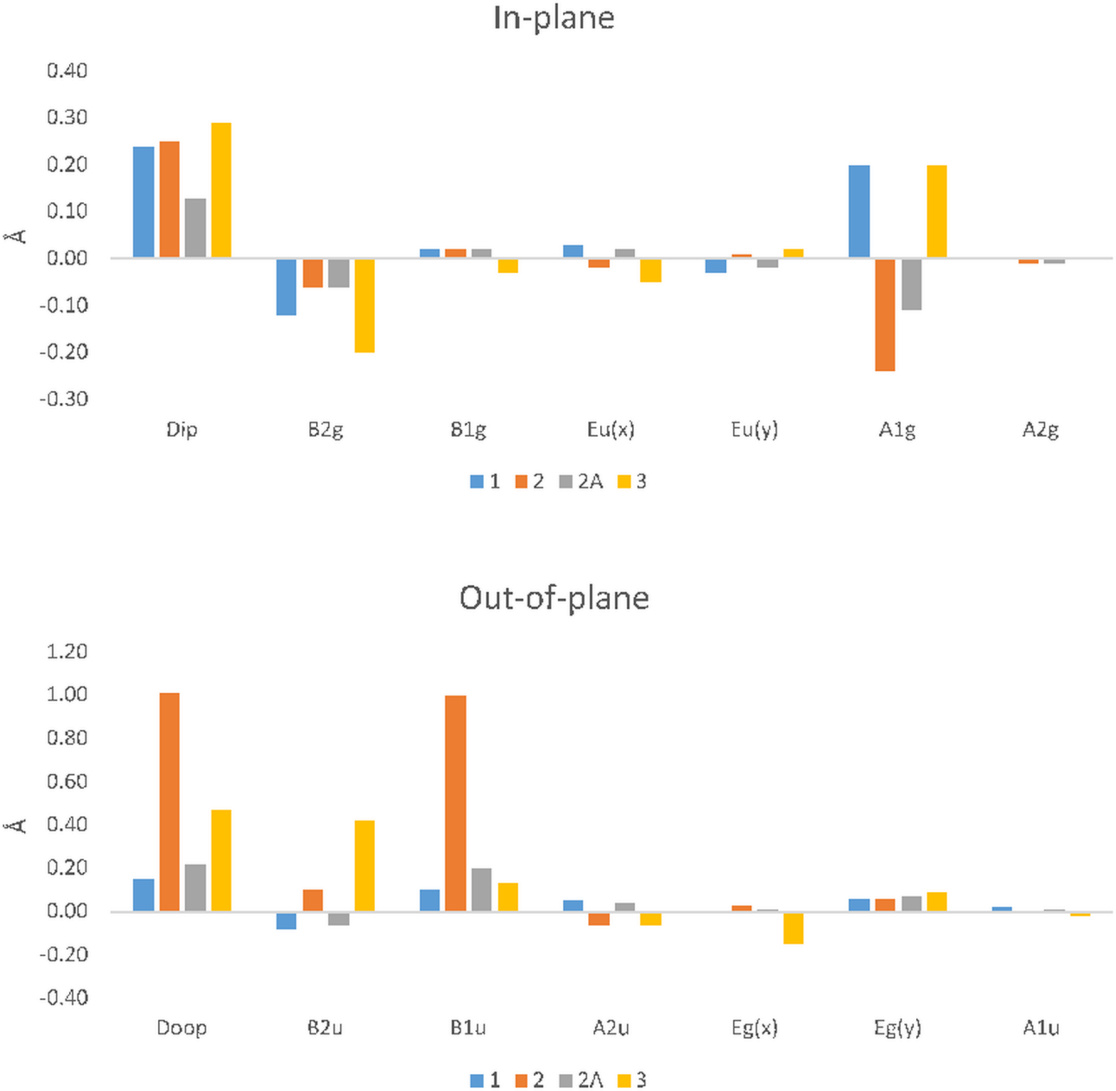
NSD charts for compounds **1**–**3**.

The differences observed for bond lengths and angles incurred by increasing the atom size are minimal between Br (**1**) and I (**3**); in most cases it is less than 0.01 Å change (Supporting Information File 1, Table S1). Of note, in this case, is the marked decrease in the 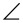
C_a_C_m_C_a_ of 2° seen in compound **3**. Generally, all atom displacements (Δ24, ΔN, ΔC_a_, ΔC_b_, and ΔC_m_) [20] show a significant increase along with the pyrrole tilt angle which are moderately increasing when substituting a bromine atom for an iodine atom. This, however, does not change the core size to any large degree. To further investigate these subtle differences, we can look at their normal-coordinate structural decomposition (NSD) profiles (Figure 7) [28].

It can be observed that in the out-of-plane (OOP) distortion modes, the overall DOOP is almost tripled between compound **1** and **3** due to an increase in the saddle (*B2u*) conformation, while little change has occurred in the in-plane (IP) with a moderate increase in the *B2g*. While an increase in the size of the substituted halogen has a moderate effect, a much larger effect is observed due to metal insertion. This is seen as a general decrease in the bond lengths, bond angles, and core size, corresponding to a significant increase in atom displacement and pyrrole tilt angles. This is essentially due to the ruffling caused by inserting a nickel(II) center [20,29–31] and is reflected in the NSD by a large increase in the contribution of the *B1u* OOP mode.

**Figure 8:**
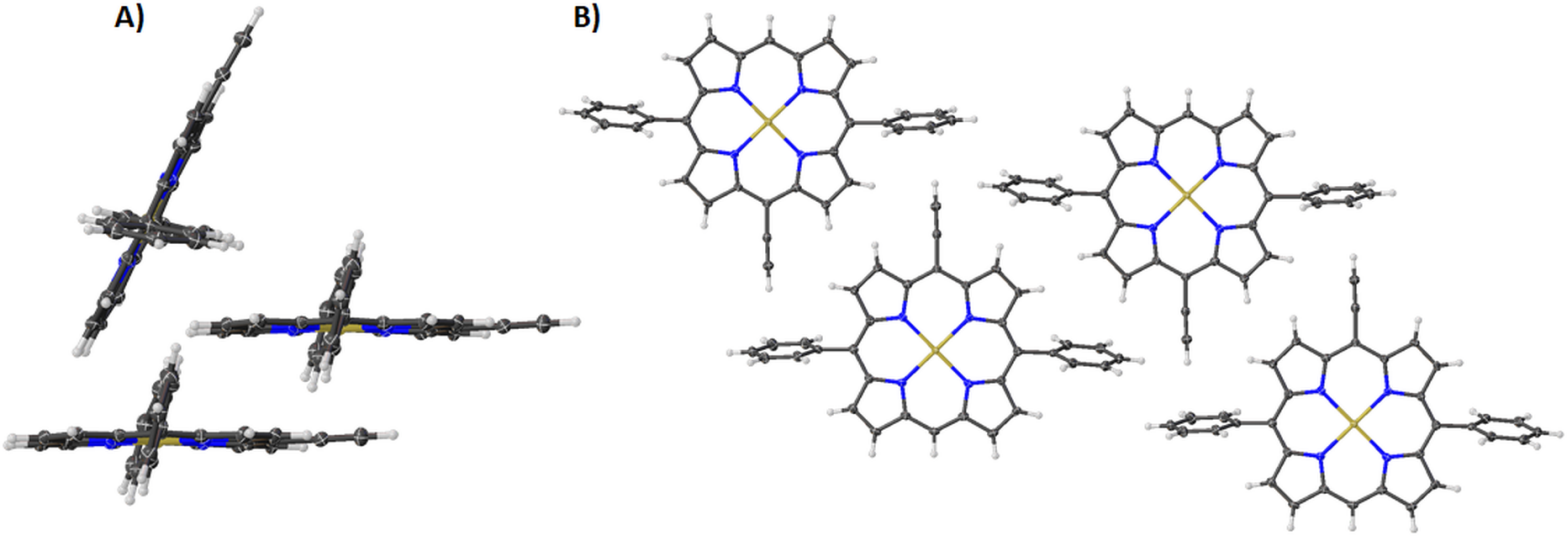
Expanded view (thermal ellipsoid plot) of compound **2A** showing (A) the edge-on and stacking interaction between the porphyrin macrocycles and (B) layers of porphyrin with ↑↓↑↓ repeating pattern.

While the differences between halogen size and metal insertion are apparent, we were fortunate to obtain the structure of the acetylene-substituted derivative of compound **2** (**2A**). In this structure, we can look at the changes between **2A** and its bromo derivative **2** and take a closer look at how the halogen influences the conformation of the porphyrin macrocycle. The first thing to note is how planar this structure of **2A** is compared to **2** (see Supporting Information File 2, Table S1). Even with the inclusion of nickel(II) in compound **2A** most of its bond lengths, bond angles, and core size are reduced, similar to compound **2,** as seen in Supporting Information File 2, Table S1. However, the atom displacement is more akin to that of compound **3** showing little deviation within the macrocycle core. The combination of these features results in a packing like the one observed in compound **1** (Supporting Information File 1, Figure S4) with the same stacking, edge-on interaction and layers of porphyrin with ↑↓↑↓ repeating pattern (Figure 8). This is also reflected in the NSD where the largest contribution is seen in the *B1u* OOP mode, but it is not much more significant than compound **3**. In the IP distortion modes, it has the lowest DIP contribution. This indicates that the effect a meso-substituted halogen has on the porphyrin macrocycle can be observed in the NSD with increasing halogen size evident in both the OOP and IP (Figure 7). Additionally, the combination of a metal center and a halogen atom seems to have a greater impact than the sum of its individual effects in the OOP, while marginal differences are observed in the IP.

#### 5-Halo-15-phenyl-substituted porphyrins

Regarding the 5-halo-15-phenylporphyrins with a varied selection of 10,20-substitution; we have obtained the crystal data for a selection of Cl, Br, and I porphyrins with either tolyl, hexyl, or phenyl substituents in the 10,20-positions (compounds **4**–**8**) (Figure 9). The labelled displacement ellipsoid plot and packing diagrams for compounds **4**–**8** are available in Supporting Information File 1 (Figures S7–S16).

**Figure 9:**
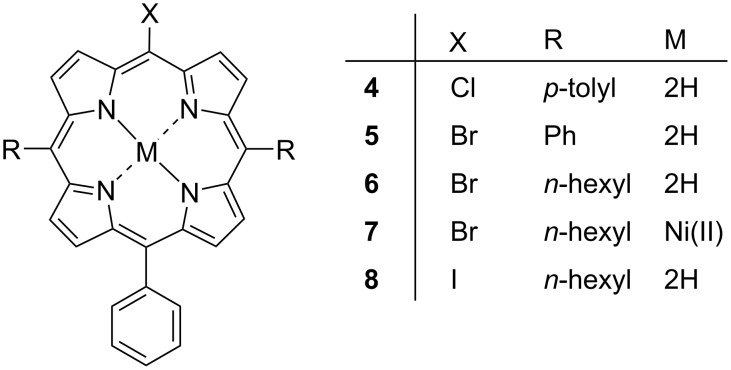
5-Halo-15-phenyl-substituted porphyrins.

**Figure 10:**
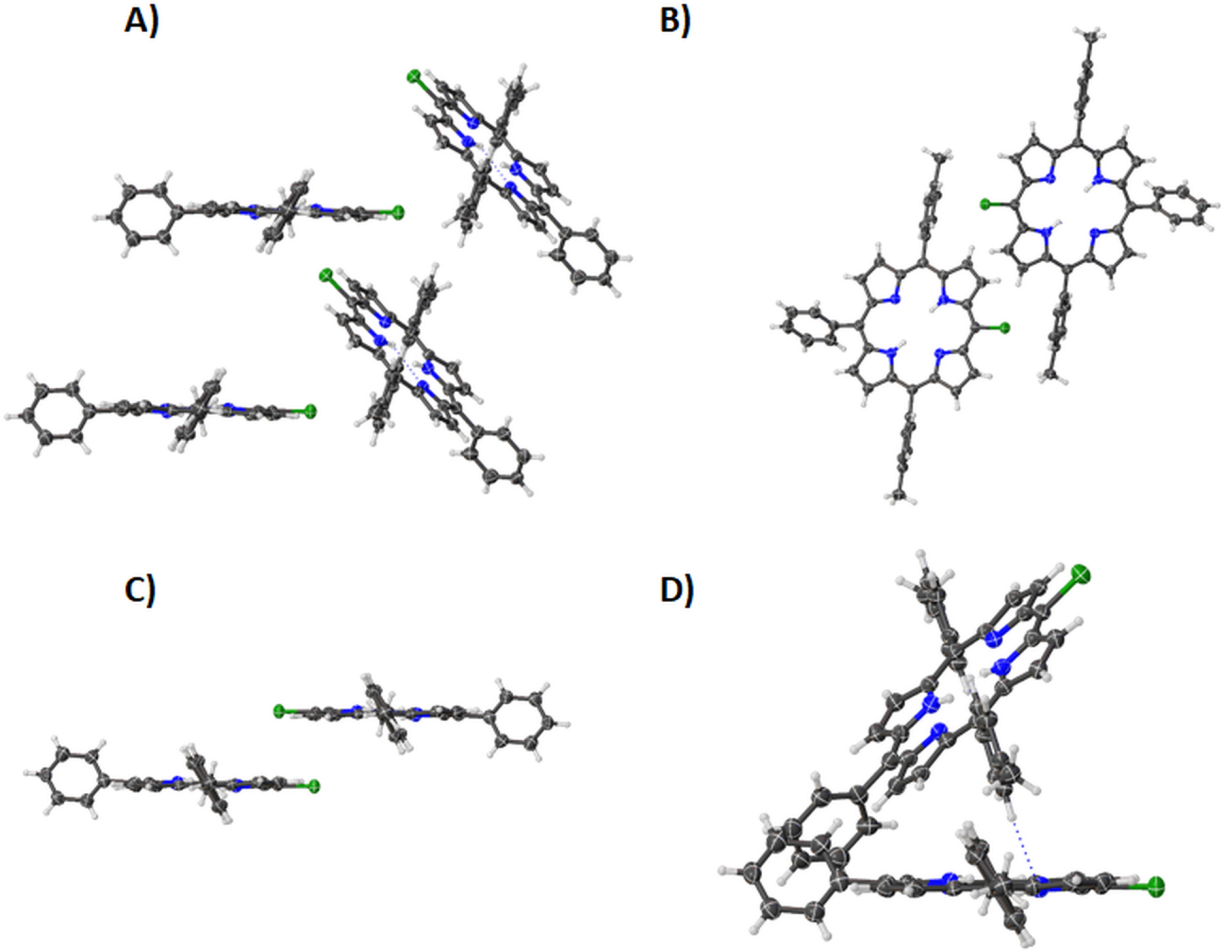
Expanded view (thermal ellipsoid plot) of compound **4** showing (A) tilted alignment of porphyrin rings, (B) chlorine atoms pointing towards the tolyl groups, (C) chlorine overlap and (D) phenyl overlap.

The structure of compound **4** is a shift from the previous 5-halo-10,20-diphenylporphyrin series. Due to the phenyl substituent added the edge-on interactions previously observed are no longer present. There is a combination of four motifs: a tilted alignment of porphyrin rings (Figure 10A), chlorine atoms pointing towards the tolyl group (Figure 10B), the chlorine overlap (Figure 10C), and the tilted overlap between phenyl rings (Figure 10D). These features combine to make a complex packing system seen in Supporting Information File 1, Figure S8. The addition of the tolyl group breaks the high-ordered packing previously observed. The chlorine atom does not appear to have any substantial interactions present in the crystal structure.

**Figure 11:**
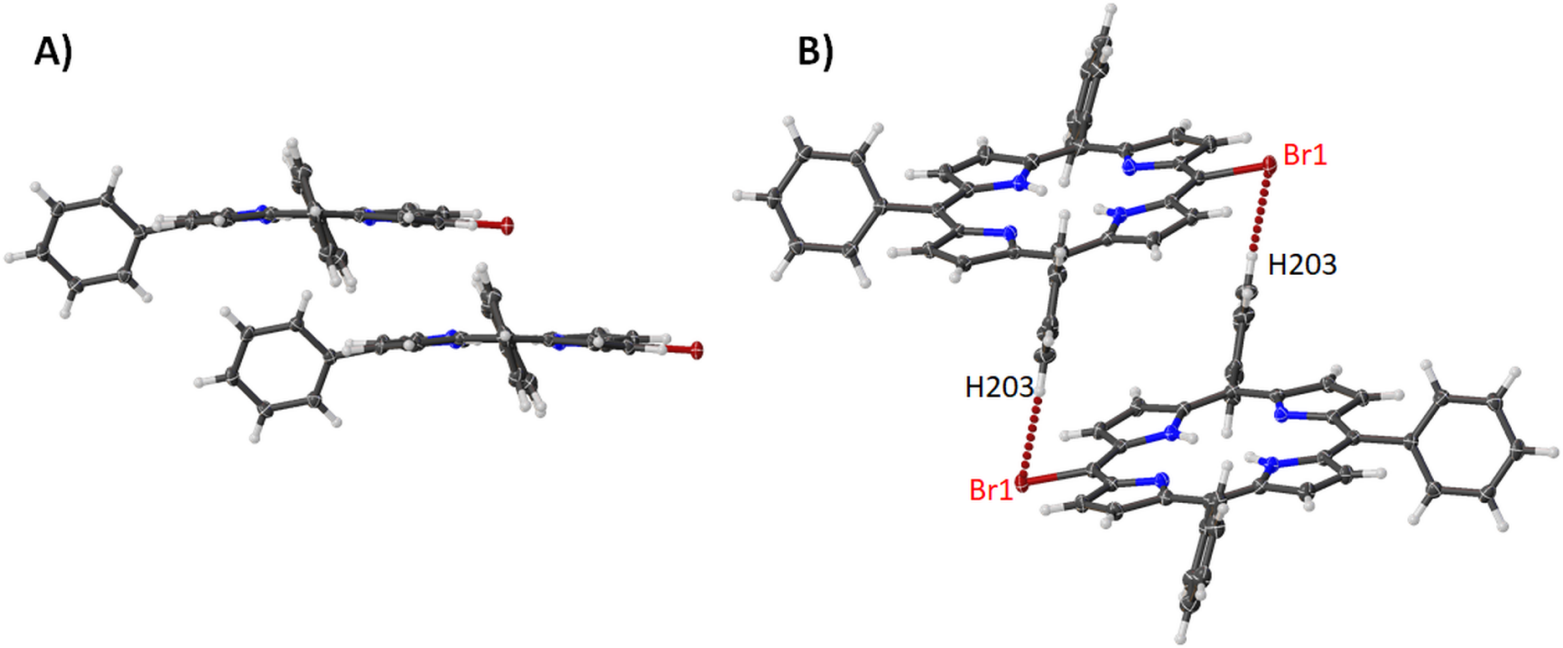
Expanded view (thermal ellipsoid plot) of compound **5** showing (A) porphyrin stacking and (B) Br···H interactions.

The structure of compound **5** only differs from the structure of compound **1** by the addition of a phenyl moiety. In this structure, the same porphyrin overlap seen in Figure 2A is apparent (Figure 11A) with a separation of 3.359(2) Å between the porphyrin layers. This is coupled with a short contact between the bromine atoms and the *meta*-hydrogen of the phenyl ring tethering porphyrin molecules to the one above it (Figure 11B, Table 1). Considering the bond lengths, angles, and atom displacement there are only marginal differences in each case except for a notable increase in the atom displacement and pyrrole tilt angles (Supporting Information File 2, Table S2). This is represented by an increase in OOP distortion mainly in the *Eg*(*y*) distortion mode and a second contribution to the *Eg*(*y*), while there are no changes to the IP modes (Supporting Information File 1, Figure S17).

**Table 1:** List of hydrogen···halogen interactions seen in compounds **5–7**.

	D···H–A	Distance (Å)	Angle (°)

**5**	Br1···H203–C203	3.138(3)	136.1(1)
**6**	Br1···H7–C7	3.191(2)	123.9(5)
**7**	Br1···H20F–C202	2.979(4)	134.0(2)

**Figure 12:**
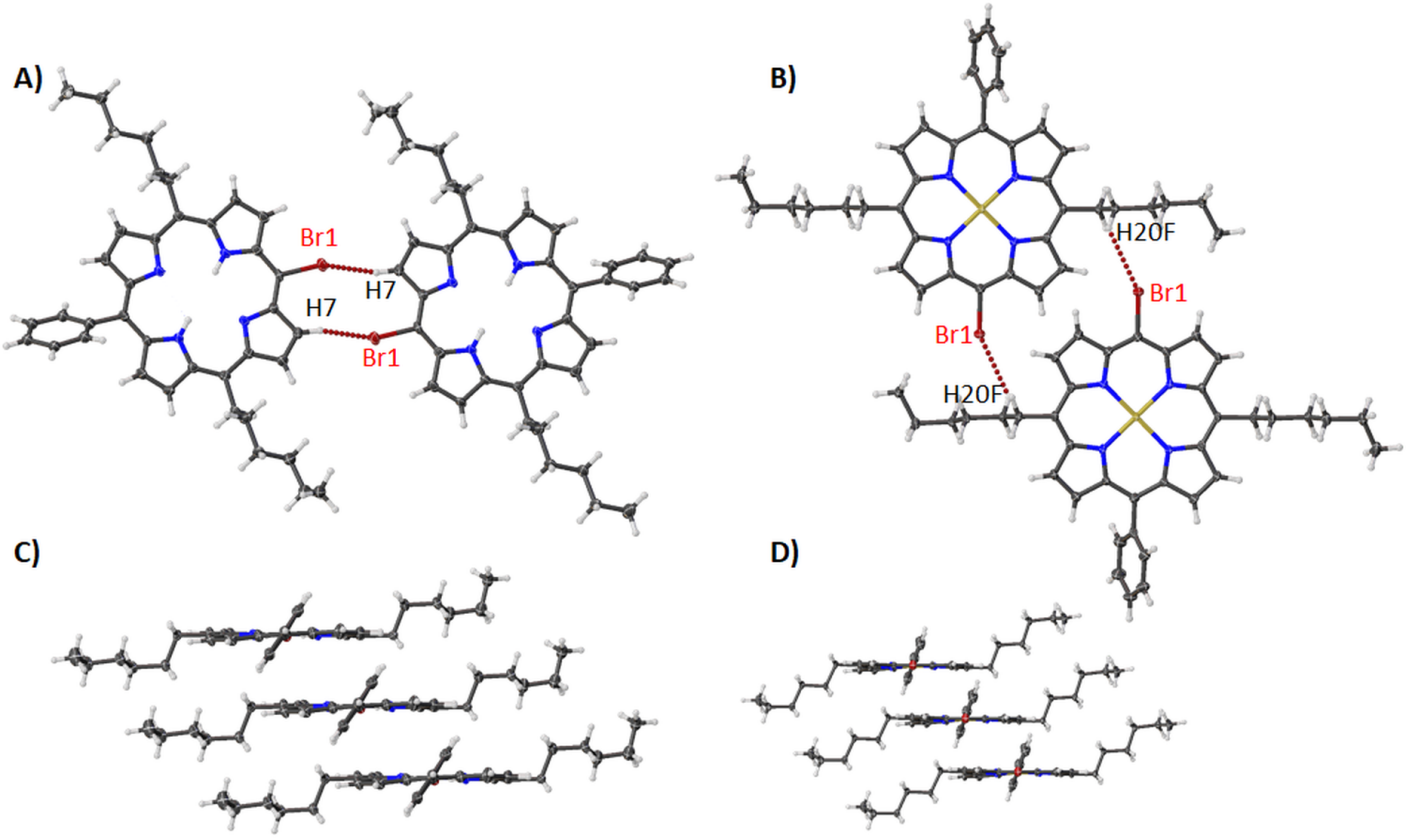
Expanded view (thermal ellipsoid plot) of compounds **6** (A and C) and **7** (B and D) showing (A) Br···H interactions with pyrrole units, (B) Br···H interactions with hexyl units and C and D)porphyrin stacking.

With the introduction of a hexyl group to the 10,20-positions of the porphyrin ring, the complete packing arrangement is changed. Compounds **6** and **7** both show a preference for interacting in a head-to-head fashion with a Br···H interaction seen between the bromine atoms and the pyrrole unit for **6** (Figure 12A) or the hexyl chain for **7** (Figure 12B) (see Table 1 for distances and angles). However, the main feature of this type is the packing, where the porphyrin macrocycles are stacked with hexyl chains aggregating with each other (**6**, 3.270(5) Å, Figure 12C) (**7**, 3.316(4) Å, Figure 12D). Looking at the bond lengths and angles (Supporting Information File 2, Table S2) there are common changes observed when introducing a nickel(II) metal center. However, the atom displacements and pyrrole tilt angles are reasonably constant. This suggests that the hexyl groups have a larger impact on the conformation and tend to alleviate ring strain by inducing a more planar conformation. This can be visualized in the NSD where the OOP of **6** and **7** are almost equal and it is only in the IP modes that they differ with an increase in the *B2g* and a corresponding decrease in the *A1g* distortion modes (Supporting Information File 1, Figure S17).

The final structure in this series is the iodine and hexyl combination (compound **8**). In this structure, the stacking is above with a separation of 3.255(5) Å (Supporting Information File 1, Figure S18). Interestingly, this structure features both arrangements previously seen with a close packing observed between both pyrrole (Supporting Information File 1, Figure S19A) and hexyl (Supporting Information File 1, Figure S19B) units depending on the residue in the asymmetric unit. Considering the bond lengths and angles (Supporting Information File 2, Table S2), there are almost no differences between compound **6** and **8** suggesting that little effect is caused by increasing the size of the halogen atom. This is also seen in the NSD where the overall profiles are similar to compound **6**, in the IP with only a small difference observed in the OOP due to a shift from the *Eg*(*x*) to the *Eg*(*y*) closer aligned to compound **4**.

To elucidate the intermolecular interactions, the Hirshfeld surface analyses were obtained (Supporting Information File 1, Figure S20). These fingerprint plots showed that X···H (halogen···hydrogen) contacts appear in a relatively similar percentage for compounds **4**–**8** (Supporting Information File 1, Figure S21) and X···C (halogen···carbon) contacts for compounds **4** and **5** display a relatively low percentage compared with compounds **6**–**8** where no interaction appear. Additionally, C···H (carbon···hydrogen) contacts displayed a decrease in the percentage with a descending order from compound **4** to **8** whereas H···H (hydrogen···hydrogen) contacts are the most numerous contributors between the interacting atoms. Regarding the substitution at positions 10 and 20, it is pointed out that the hexyl groups affect the pattern of C···H (decrease of the percentage) and H···H (increase of the percentage) contacts.

#### 5,15-Di-halo-substituted porphyrins

**Figure 13:**
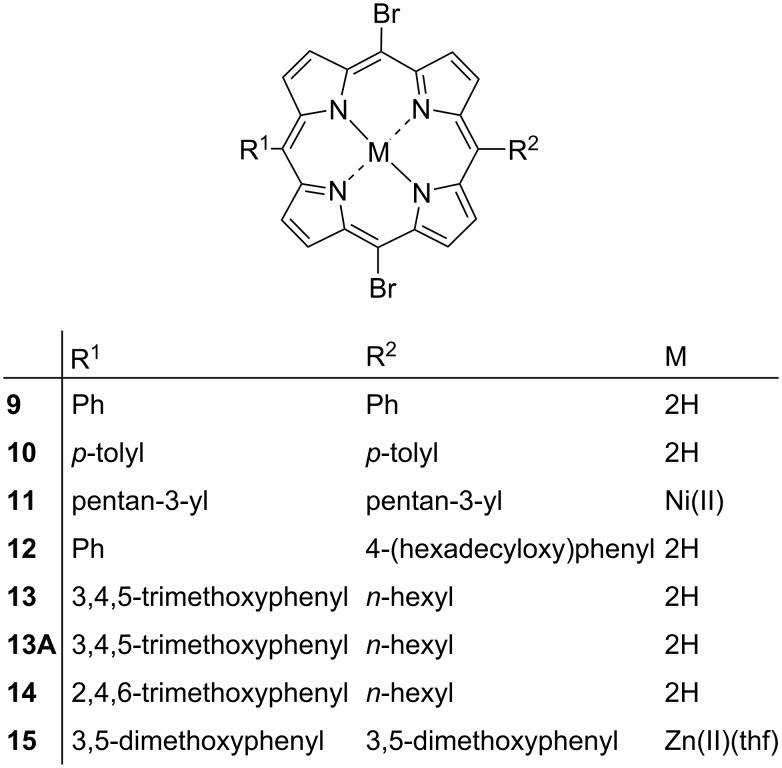
5,15-Di-halo-substituted porphyrins.

In this series, we investigate the effects of 5,15-di-halo-substitution that contain a variety of aryl or alkyl substituents at the 10,20-positions (compounds **9**–**15**) (Figure 13). The labelled displacement ellipsoid plot and packing diagrams for compounds **9**, **10**, **11**, and **13** are available in Supporting Information File 1 (Figures S22–S29). The main feature of this series is the increase in the number of X···H interactions and the appearance of X···X (halogen···halogen) interactions which are outlined in Supporting Information File 2, Table S3.

**Figure 14:**
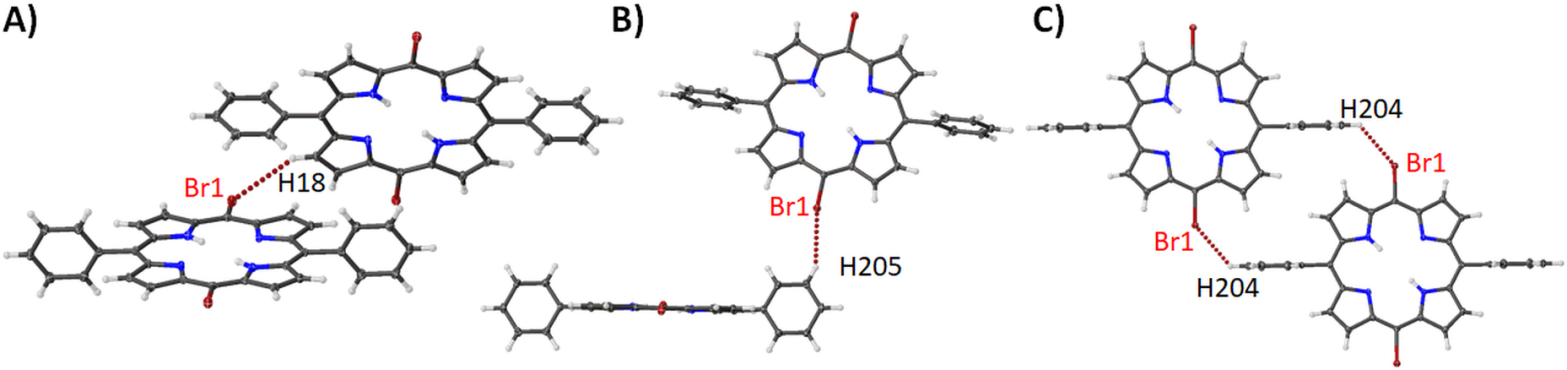
Expanded view (thermal ellipsoid plot) of compound **9** showing the Br···H interactions with (A) pyrrole unit, (B) *meta-*hydrogen and (C) *para-*hydrogen.

**Figure 15:**
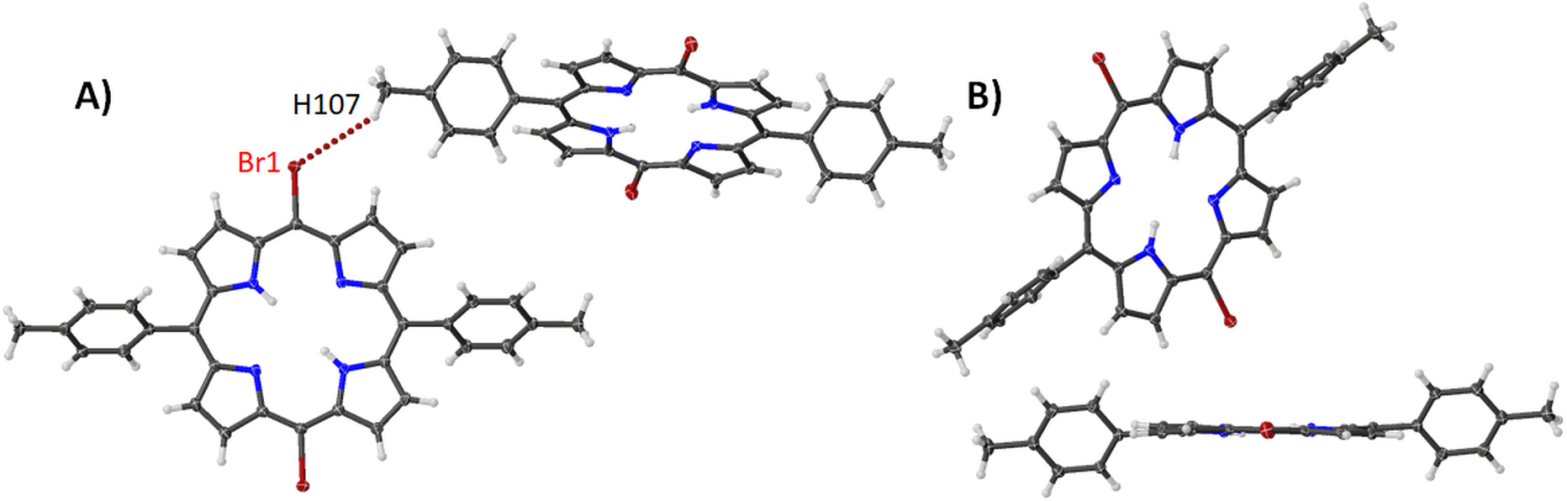
Expanded view (thermal ellipsoid plot) of compound **10** showing the (A) Br···H interactions with tolyl moiety and (B) edge-on interaction between the porphyrin macrocycles.

The structure of compound **9** shows three motifs driven by Br···H interactions (Figure 14) that when combined (Supporting Information File 1, Figure S30) establish the packing pattern seen in Supporting Information File 1, Figure S23. As with compound **1,** the stacking between the porphyrin layers is organized with the bromine atoms overlapping with each other at a separation of 3.306(3) Å among the porphyrin macrocycles. In compound **9** we see a structure that is similar to both compounds **1** and **5** in basic architecture. This is reflected in the bond lengths, angles, and atom displacement (Supporting Information File 2, Table S4) where a similar distance is observed for both compounds **1** and **5**. It is in the atom displacement that the effect of the di-bromination is evident. The addition of a phenyl ring to the 15-position (compound **5**) increases the disorder observed. However, when this is changed to a bromine atom, an alleviation of ring strain is observed resulting in a structure that is more planar than compound **1**. Substituting the phenyl groups for tolyl groups as in compound **10** results in a similar structure with bond lengths, etc. (Supporting Information File 2, Table S4), except for a marginal increase in the atom displacement and pyrrole tilt angles. While these changes are only minimal, there is a variation of the packing compared to **9** with a more V-shaped pattern (Supporting Information File 1, Figure S25). This is directed by a combination of Br···H interaction between the bromine and the methyl moiety rather than aromatic hydrogens (Figure 15A) and the tilted edge-on interaction (Figure 15B). Comparing the NSD profiles of both samples their IP profiles are almost identical, but the extra distortion can be seen in the OOP with an increasing contribution of the *Eg*(*x*).

**Figure 16:**
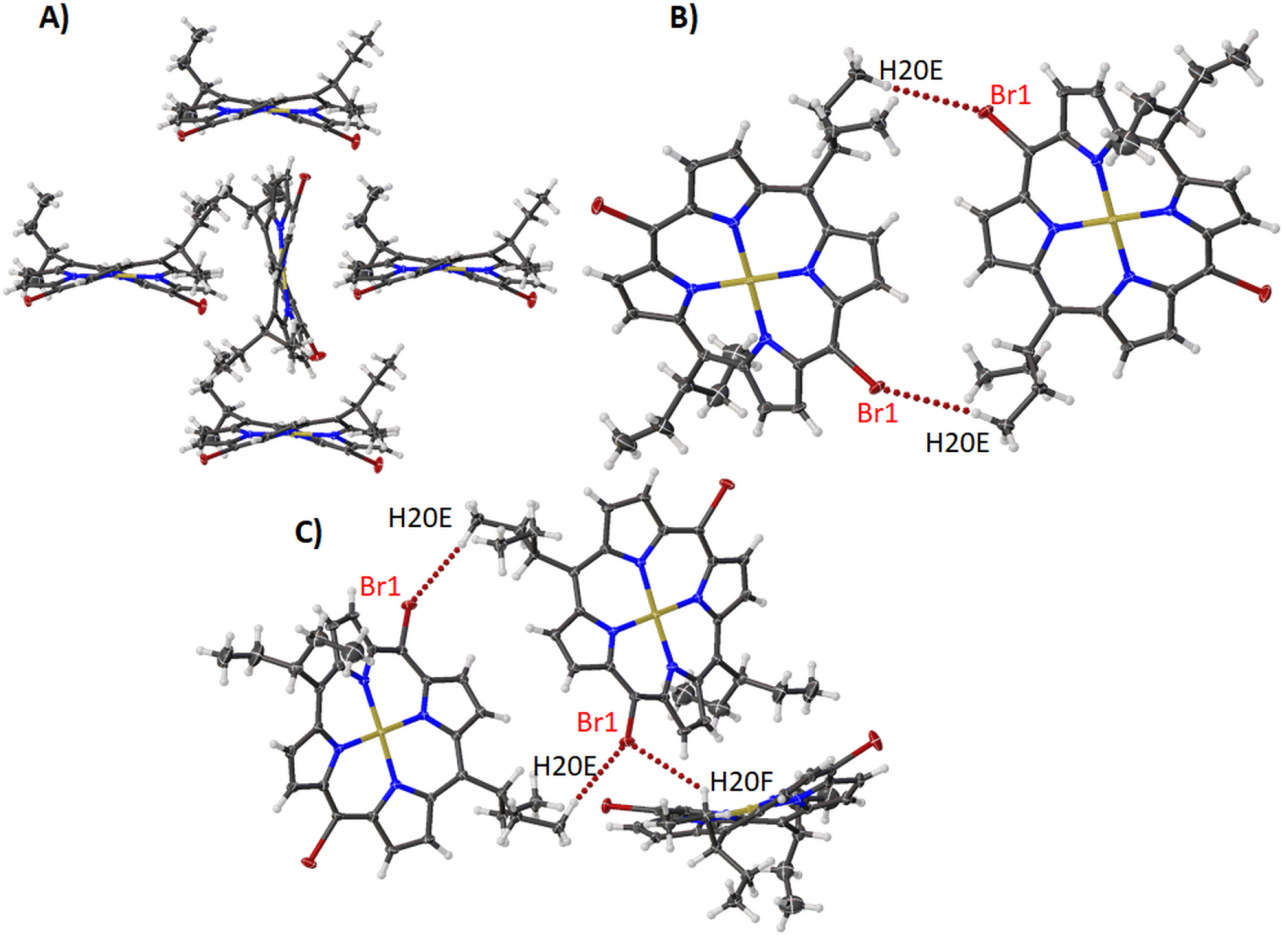
Expanded view (thermal ellipsoid plot) of compound **11** showing the (A) edge-on interactions, (B) edge-on Br···H interactions and (C) Br···Br contact.

Compound **11** adopts a packing arrangement that is unique among the compounds studied in this section. As seen in Figure 16A, one central porphyrin is surrounded on four sides by four other porphyrin molecules. This is tethered through a selection of Br···H interactions which are outlined in Supporting Information File 2, Table S3. The second motif seen in this structure is a Br···H interaction which is reciprocated between two porphyrin molecules (Figure 16B) and Br···Br contact on the opposite side (Figure 16C). The bond lengths etc. are typical of nickel(II) porphyrins [32], however, it appears that the distortion of compound **11** is much greater than the nickel(II) porphyrins within this series. The distortion of this structure is almost twice large as the second-largest distortion (compound **2**) which is reflected in both the OOP and IP (Supporting Information File 1, Figure S31) and can be seen in the larger increase of the atom displacements and pyrrole tilt angles (Supporting Information File 2, Table S4).

The structure of compound **12** is an interesting example of the effects that long alkyl chains have on the overall packing. This is seen as the preferred alignment of alkyl chain with each other, reminiscent of a lipid bilayer (Supporting Information File 1, Figure S32) [23]. There are four motifs that contribute to the overall packing seen in Supporting Information File 1, Figure S33. The first two (Supporting Information File 1, Figure S32A and B) are directed through alkyl interactions with a head-to-head overlap (Supporting Information File 1, Figure S32A) and the stacked system (Supporting Information File 1, Figure S32B). The third motif is the close-packed side-on alignment which is due to the space occupied by the bromine atom, although there is no indication of potential halogen interactions. The final motif is a head-to-head Br···H contact between the porphyrin macrocycles (Supporting Information File 1, Figure S34) which tether the porphyrin together extending the network to be an alkyl···porphyrin···alkyl···porphyrin type system.

**Figure 17:**
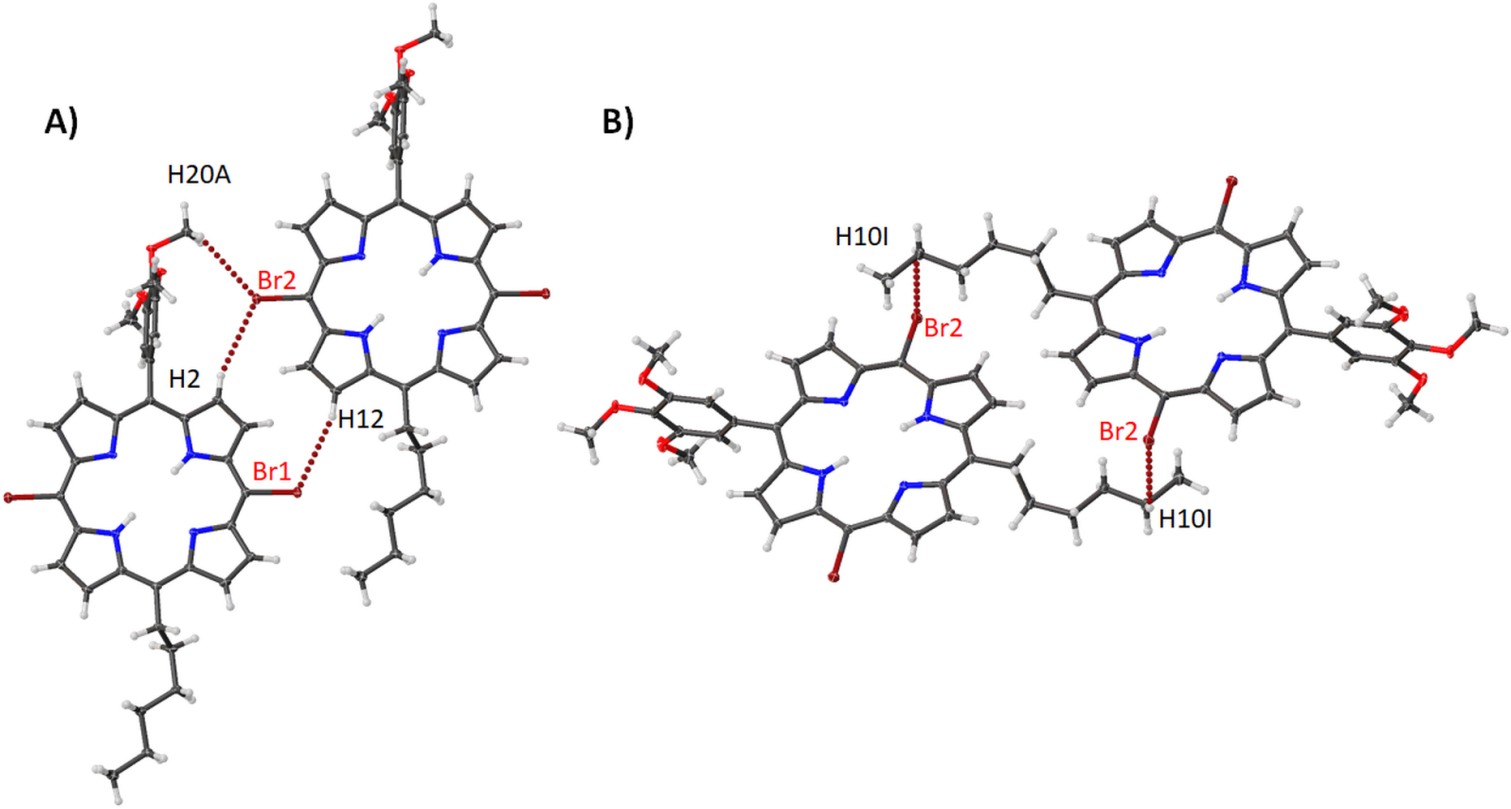
Expanded view (thermal ellipsoid plot) of compound **13** showing (A) Br···H interactions with pyrrole units and methoxy group and (B) Br···H interactions with hexyl chains.

**Figure 18:**
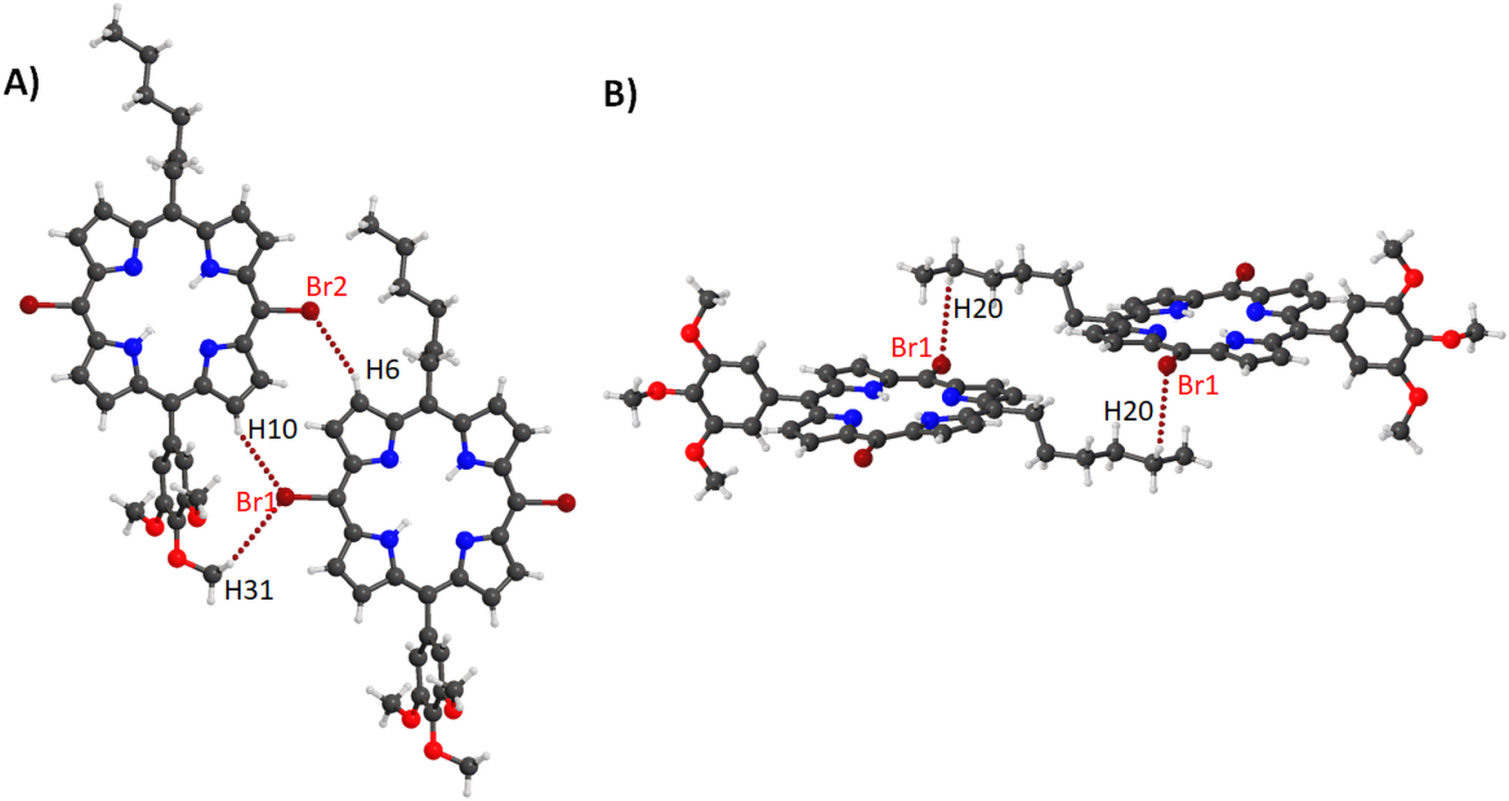
Expanded view (ball and stick) of compound **13A** showing (A) Br···H interactions with pyrrole units and methoxy group and (B) Br···H interactions with hexyl chains.

In the next three compounds **13**, **13A**, and **14** the main structure only differs by the steric demand due to one meso aryl unit (3,4,5- trimethoxyphenyl (**13** and **13A**), 2,4,6-trimethoxyphenyl (**14**)) on one side [24]. Compound **13** is a recollection of the older sample **13A** and is almost identical in structure. Both feature a parallel alignment of porphyrins tied together with the reciprocated Br···H contacts with the pyrrole hydrogens and a second contact between the bromine and the *p*-methoxy hydrogens (Figure 17A, Figure 18A). Both structures also feature the hexyl chain overlapping with the face of the porphyrin ring (Figure 17B, Figure 18B, Supporting Information File 1, Figure S35). Compound **14** forms a similar parallel alignment as the one seen in compounds **13** and **13A** (Supporting Information File 1, Figure S36A) with the *o*-methoxy hydrogens interacting with the bromine atom. Additionally, there is the presence of weakly π-stacked dimers in the crystal with a hexyl chain on the same face as a neighboring aryl group (Supporting Information File 1, Figure S36B). This results in a closer contact between the porphyrin layers compared to the packing of compounds **13** and **13A** (Supporting Information File 1, Figure S37). However, these deviations are in line with crystal packing and minor steric effects to be expected for this class of compounds with little to no deviations seen in the bond lengths, angles, and atom displacement (Supporting Information File 2, Table S5).

The last compound in this series is **15**. In this structure, the porphyrins rings are set up in an edge-on interaction with both sides of the porphyrin ring tethered through the bromine atoms and the aryl units (Supporting Information File 1, Figure S38) [33]. On the side containing the axial THF molecule these interactions hold the pyrrole unit towards the face of the porphyrin (Supporting Information File 1, Figure S38A). On the opposite side, the pyrrole units are held above the central metal atom through one Br···H contact (Figure S38B). This forms a repeating pattern of face-to-edge interactions with only minimal interference from the axial ligand (Supporting Information File 1, Figure S39A) and a hydrogen-bonded network between the methoxy moieties (Supporting Information File 1, Figure S39B). Both features are expressed throughout the crystal packing (Supporting Information File 1, Figure S40).

Hirshfeld surface analysis maps the corresponding intermolecular interactions with H···H contacts being the dominant ones with minor alterations in the percentage between this series of halogenated porphyrins (Supporting Information File 1, Figures S41 and S42). Only compound **12** displays a higher percentage of H···H contacts together with a lower percentage of X···H contacts compared with the rest of the compounds. C···H contacts for compounds **9**–**11** are slightly higher than the respective ones of compounds **12**–**15,** and X···C contacts do not display any significant difference amongst them. It is shown that the alkyl group, this time the hexadecyloxy group, can affect the pattern of C···H (decrease of the percentage) and H···H (increase of the percentage) contacts.

#### 5,10-Di-halo-substituted porphyrins

**Figure 19:**
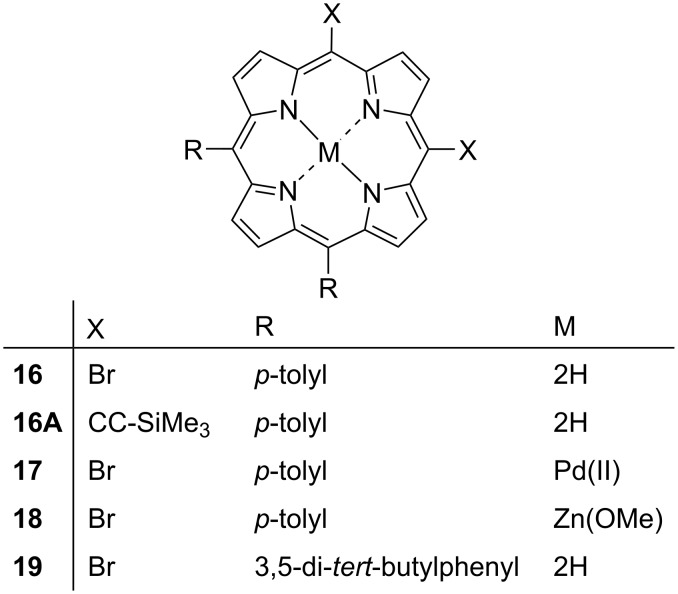
5,10-Di-halo-substituted porphyrins.

In this series, we are investigating the effects 5,10-di-halo-substitution that contain either a *p*-tolyl or 3,5-di-*tert*-butylphenyl group in the 15,20-positions (compounds **16**–**19**) (Figure 19). The structure of compound **16A** is a TMS-acetylene derivative of compound **16** which is included to analyze the difference in halogen and alternative substitutes. While the structure of **16A** is of low quality, it was included in this section for a continuity of discussion and this structure should be attempted with better crystal data in the future. The labelled displacement ellipsoid plot and packing diagrams for compound **16A** are available in Supporting Information File 1 (Figure S43 and Figure S44). The hydrogen···halogen interactions are listed in Table 2.

**Table 2:** List of hydrogen···halogen interactions in compounds **16**–**19**.

	D···H–A	Distance (Å)	Angle (°)

**16**	Br2···H9–C12	3.076(17)	138.2(4)
	Br2···H26–C34	3.115(17)	171.6(4)
**17**	Br2···H11–C25	2.915(16)	148.6(5)
**18**	Br1···H19–C33	3.053(9)	134.6(3)
	Br2···H23–C35	3.113(8)	159.3(3)
**19**	Br1···H13–C19	3.032(5)	159.3(2)
	Br1···H20–C23	3.118(4)	133.6(5)

**Figure 20:**
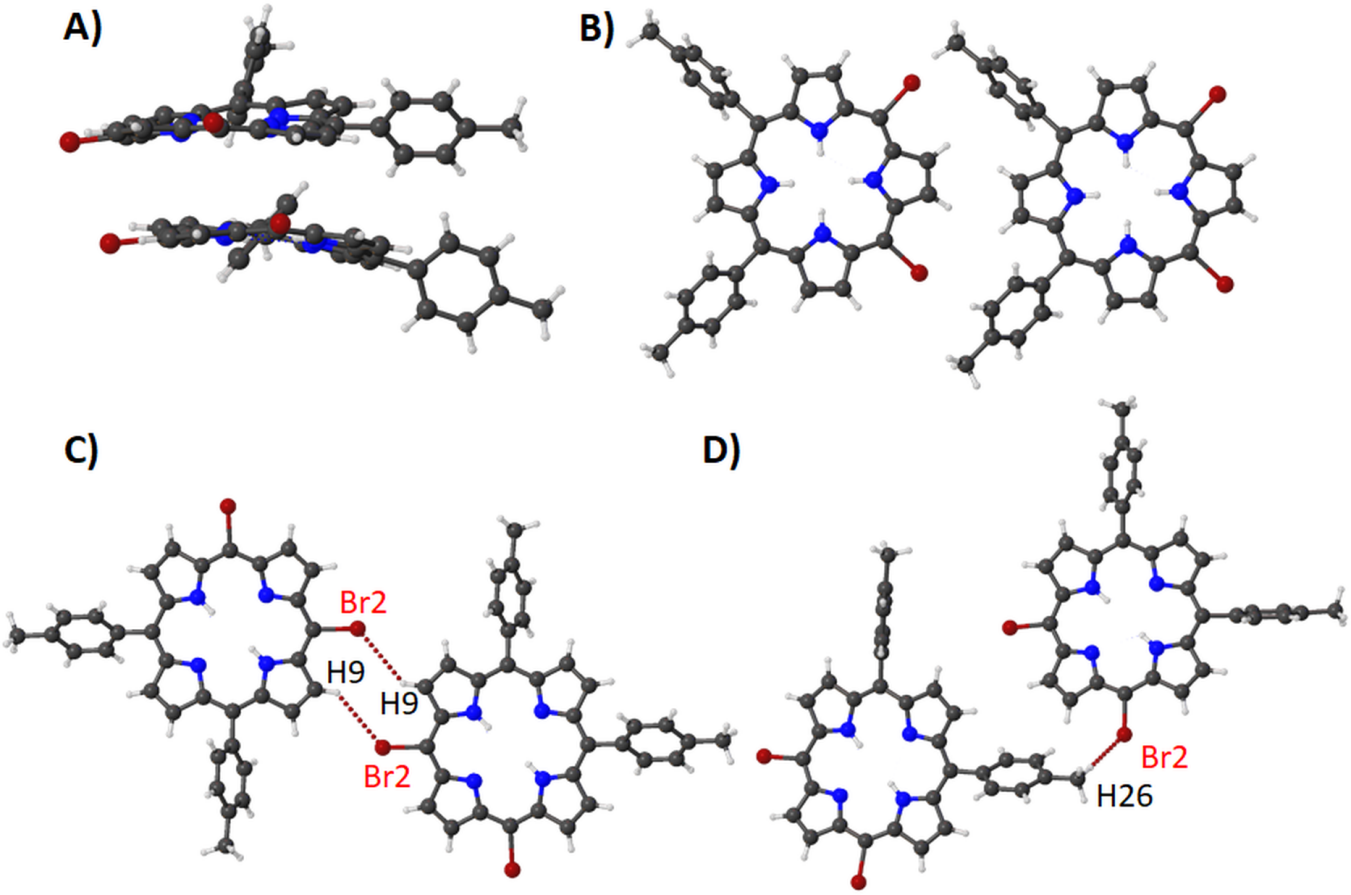
Expanded view (ball and stick) of compound **16** showing (A) stacking, (B) head-to-tail alignment, (C) Br···H interactions with pyrroles and (D) Br···H interactions with tolyl moiety.

Compounds **16**–**18** are a series of structures that only differ by the type of metal center in the porphyrin core [34–35]. In compound **16** the stacked porphyrin layers are orientated in a parallel arrangement with a 3.704(4) Å separation (Figure 20A) whilst a lateral alignment where the bromine atoms are pointing towards the tolyl groups in a linear network is observed (Figure 20B). The two other motifs seen are the bromine pyrrole interactions which is reciprocated in a head-to-head alignment (Figure 20C) and the bromine tolyl interaction in a head-to-tail alignment (Figure 20D), which combine to form the packing illustrated in Supporting Information File 1, Figure S45.

By substituting the bromine atoms with a TMS-acetylene group the apparent lateral alignment of **16A** is distorted due to the larger steric demand of the TMS moiety (Supporting Information File 1, Figure S46A). Additionally, while this structure expresses the same head-to-tail overlap between stacked moieties (separation between layers at 3.573(3) Å), shows a greater degree of overlap (Supporting Information File 1, Figure S46B). In compound **17**, the stacking has alternated to a head-to-tail overlap with 3.562(5) Å separation between the porphyrin layers (Supporting Information File 1, Figure S47A). Moreover, there is a head-to-tail linear network (Supporting Information File 1, Figure S47B) which is tied together with a Br···H contact with the aryl hydrogens. The combination of the vertical stacking and the lateral network forms the crystal packing (Supporting Information File 1, Figure S48). Compound **18** exhibits the same lateral alignment seen in the previous two structures, with all axial solvents pointing in the same direction (Supporting Information File 1, Figure S49A). The differences occur due to the presence of the axial methanol solvent in which the solvent hydrogen atoms interact with the bromine atom creating a staggering network (Supporting Information File 1, Figure S49B). Two other stacking motifs are noted in this structure on either side of the porphyrin face. The first is the stacking between two molecules where axial solvents are sandwiched between the porphyrin layers at a separation of 4.432(4) Å (Supporting Information File 1, Figure S50A). On the opposite side of the porphyrin, there is a weak stacking interaction at a separation of 3.215(3) Å (Supporting Information File 1, Figure S50B). These features are expressed in the crystal packing in a repeating pattern (Supporting Information File 1, Figure S51).

The last compound in this series contains a 3,5-di-*tert*-butylphenyl in the 15,20-positions of the porphyrin ring (compound **19**) [36]. This structure is a step up in steric bulk compared to compounds **16**–**18**. With this addition, the inline alignment of porphyrins previously seen has changed to a step-wise pattern though a Br···H interaction with the four nearest neighbors (Supporting Information File 1, Figure S52). The stacking seen in this structure is directed mainly to accommodate the large size of the aryl subunit creating a slightly offset stacking pattern with a separation of 3.4526(7) Å (Supporting Information File 1, Figure S53). This creates a tightly packed structure (Supporting Information File 1, Figure S54).

Overall, in this series one of the main features that is consistent is the network between the bromine atoms and the tolyl groups with only minor differences due to the insertion of a metal center and axial solvent molecules. This is seen in the Hirshfeld surface analysis, where only small variations in the percentage of contacts formed between compounds **16**–**18** with a marginal increase in the H···H and a decrease in C···H contacts in compound **19** due to the bulky *tert*-butyl groups (Supporting Information File 1, Figure S55). With regards to bond lengths, angles, and atom displacement, there are only minor differences seen due to the inclusion of the metal center with compound **18** showing the lowest distortion with a small decrease in the atom displacement (Supporting Information File 2, Table S6). This is further compounded when moving to compound **19** with the large steric groups appearing to cause much more planar conformation of the porphyrin macrocycle. This is exemplified in the NSD profiles (Supporting Information File 1, Figure S56) where there is a general trend of decreasing distortion in the OOP from **16** to **19** with the only outlier seen in the minor increase of *B1u* contribution for the palladium derivative **17**. In the IP this is further demonstrated with a significant decrease in the *A1g* contribution of **17** giving the palladium derivative the lowest overall DIP contribution. This indicates that while there are minor differences within conformation of these porphyrins, overall crystal packing is more reliant on the substituent groups and axial ligands. A final point in this series is the large increase in distortion comparing 5,10-di-halo-substituted (**16**) to 5,15-di-halo-substituted (**9**) derivatives due to the ‘*trans*’ nature of the substituents.

#### Other halogeno-substituted porphyrins

**Figure 21:**
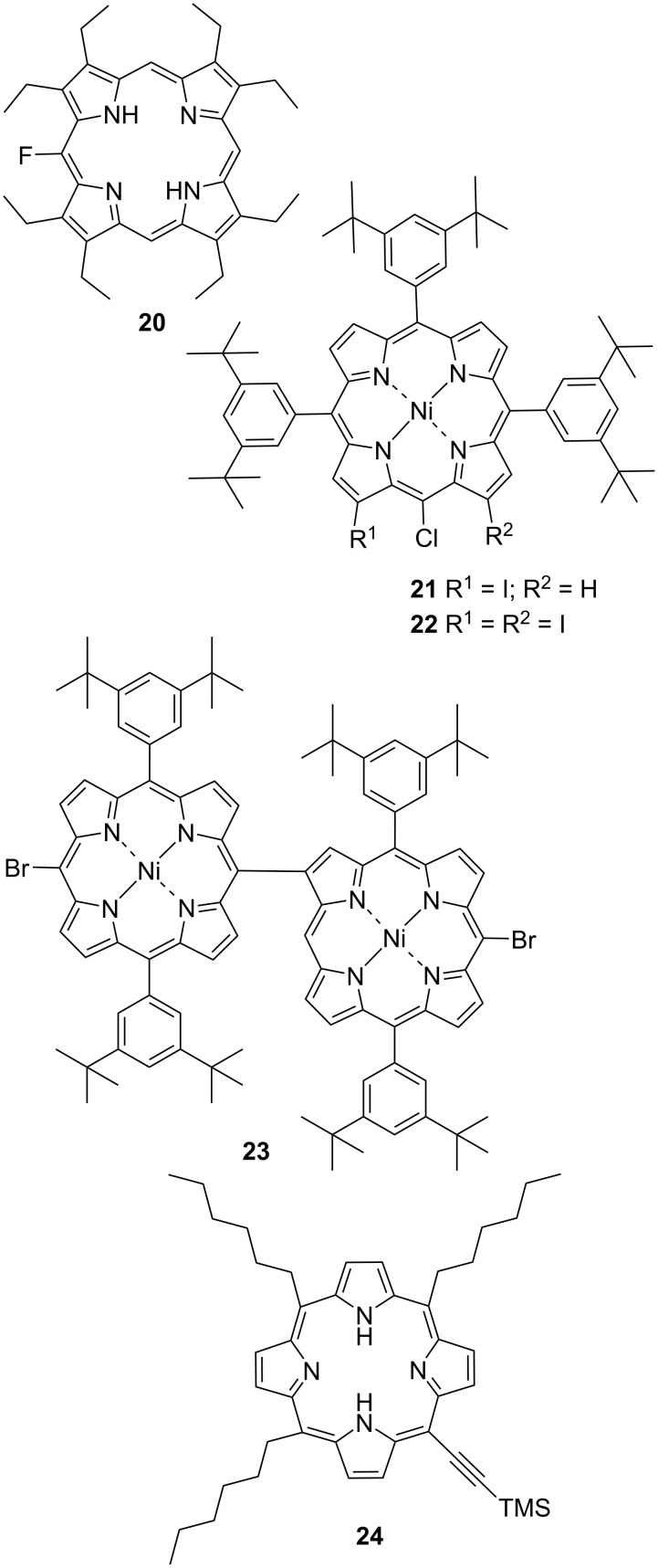
Honorable mentions of halogen-substituted porphyrins taken from the CSD database.

In the CSD database, there are a few other notable examples which whilst they are interesting, they do not constitute a significant library to support a complete discussion (compounds **20**–**24**, Figure 21) [37–39]. Other examples do exist in the CSD, such as strapped systems [40], but have been omitted due to the alternate functionality muting any effect the halogen would have on the porphyrin molecule. The bond lengths, angles, and atom displacements are outlined in Supporting Information File 2, Table S7.

In the structure of compound **20**, while containing a halogen, it is apparent that the fluorine atom is too small to overly impact the overall packing. This structure has a planar conformation similar to that of 2,3,7,8,12,13,17,18-octaethylporphyrin [41]. Due to the high disorder in the crystal structure (the fluorine atom is disordered over the four meso positions) an accurate accounting of the interaction profile is not possible. However, there is the appearance of an F···H interaction between the ethyl groups and the fluorine atom which is projected throughout the crystal packing (Supporting Information File 1, Figure S57). In compounds **21** and **22,** the number of β-halogen atoms create two different packing patterns. The first is the head-to-head interactions directed through the chlorine atoms interacting with the *tert*-butyl hydrogens (Supporting Information File 1, Figure S58A). With the addition of a second beta halogen, this motif drastically changes to become a face-to-edge interaction with the halogen atoms pointing towards the center of the porphyrin ring (Supporting Information File 1, Figure S58B). This second β-halogen creates a much more ordered packing pattern compared to **21** (Supporting Information File 1, Figure S59). The final structure (**23**) is the only example of a porphyrin dimer with a di-halogeno-substitution. Interestingly, this structure despite the extended dual-core and tilt of the porphyrin rings behaves quite like a 5,15-di-halo-substituted porphyrin. The bromine atoms are seen to interact with the *tert*-butyl and pyrrole hydrogen atoms in a head-to-tail arrangement (Supporting Information File 1, Figure S60) which is propagated through the crystal packing (Supporting Information File 1, Figure S61). Finally, the structure of **24** is included due to the presence of **2A** and **16A** (Supporting Information File 1, Figures S62–S63). As a series of TMS-ethynyl structures, the features are generally similar to each other.

### DFT calculations and NSD analysis

**Figure 22:**
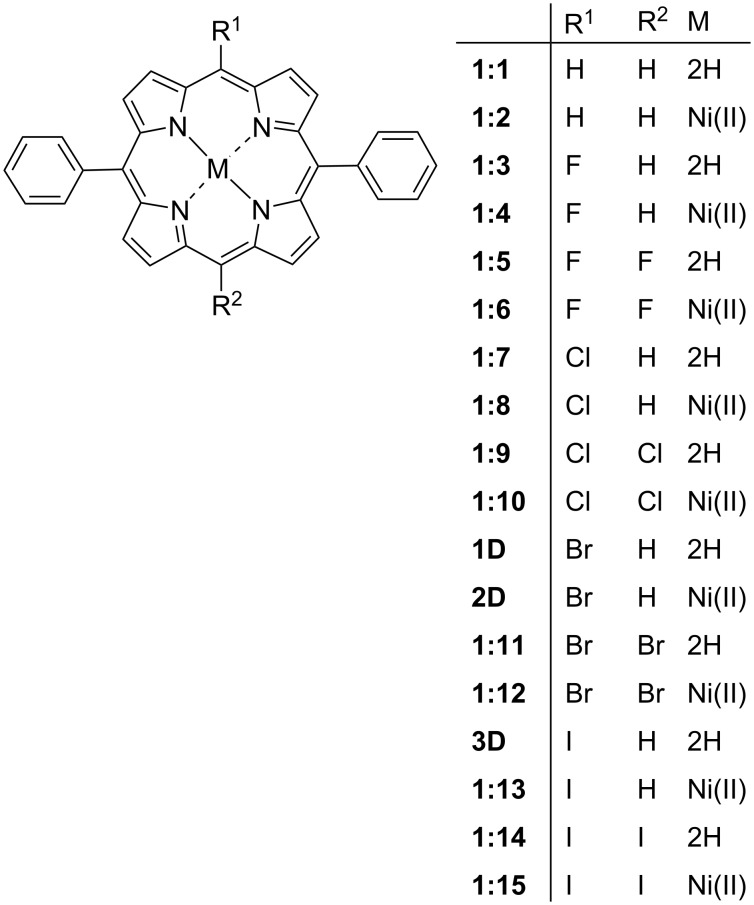
Series 1 – 5,15-di-halo-substituted porphyrins.

**Figure 23:**
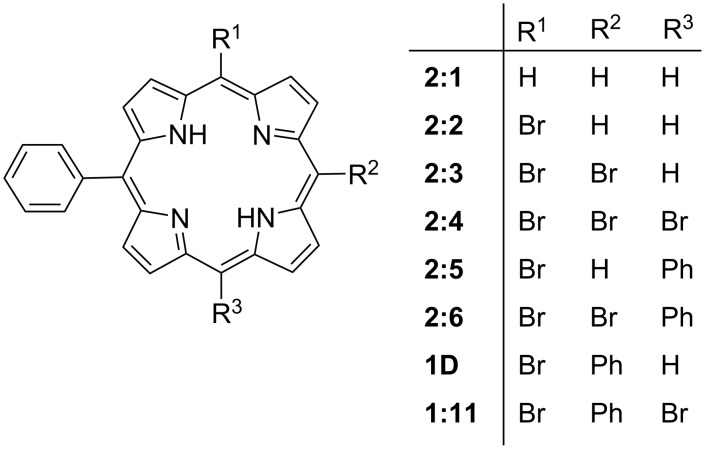
Series 2 – increasing number of halogen substituents.

**Figure 24:**
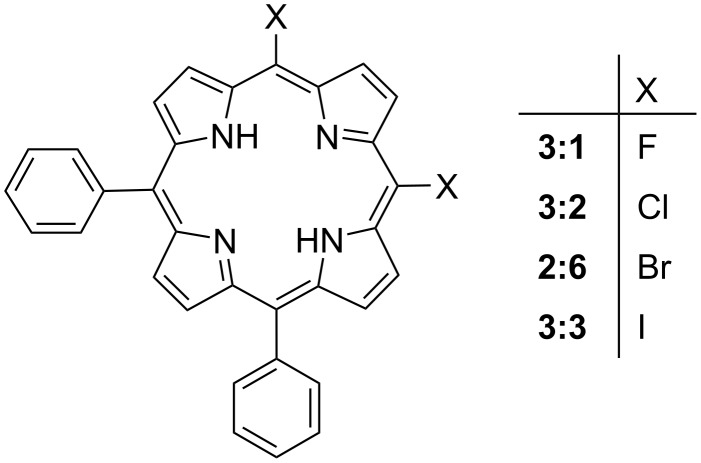
Series 3 – 5,10-di-halo-substituted porphyrins.

The limited availability of meso-halogenic porphyrin crystal structures in the CSD makes it difficult to study a complete series. While we can develop certain trends based on the current data sets, the gaps present are a current limitation. To combat this, we decided to expand these series with ab-initio computational molecular modelling. We focused on the structural modelling of three series. The ground-state geometries of several members of these series were optimized at the wB97-XD/cc-pVDZ level of theory and combined with NSD analysis to approximate the IP and OOP distortion modes. The wB97XD functional was chosen because it has been shown to produce reliable energy minimized structures for organometallic complexes and metalloporphyrins [42–44]. With the first series (series 1) we looked at the effect of increasing the size and number of the halogen substituents (Figure 22) beginning with 5,15-diphenylporphyrin (**1:1**) and moving through the halogen series with both mono- and di-halo-substituted porphyrins and their metal counterparts. Series 2 contains a selection of 5-phenyl substituted porphyrins with 1–3 meso bromine substitutes on the alternate positions (Figure 23). This is combined with the 5,10-diphenyl substituted porphyrins with either one or two bromine atoms substituted at the alternate meso positions. The final series (series 3, Figure 24) demonstrates the increasing size of halogen atoms for 5,10-di-halo-15,20-diphenyl substituted porphyrins which were carried out to demonstrate the difference in halogen size has on the overall impact of the porphyrin macrocycle. The frontier molecular orbitals (HOMO and LUMO) at the ground-state optimized geometries, as well as figures of the electrostatic potential maps showcasing the sigma-hole regions, can be found in Supporting Information File 2, Tables S8–S10 and Supporting Information File 1, Figures S64–S71. While an in-depth systematic analysis of the electronic structure is out of the scope of this work, we have included this data in the supporting information for completion. However, we were able to extract atom coordinates and NSD data which can be compared to the existing crystal structures (Supporting Information File 1, Figures S72–S74 and S75–S129) [28,45].

#### Series 1

In series 1 (Figure 22) the conformations of the mono- and di-halo-substituted porphyrins are only marginally different. The difference between the meso-free and fluorine derivatives is minimal. For the chloro to iodo derivatives, the disubstitution only exhibits a marginal decrease in the out-of-plane distortion relative to the mono-substitution, with the larger halogen inducing more planar conformation (see Supporting Information File 1, Figure S72, and Supporting Information File 2, Table S11–S13). Upon the introduction of a metal center [nickel(II) in this case] the inverse is observed. The insertion of nickel introduces distortions of the porphyrin core similar to those that have been previously reported [29]. However, there is a secondary trend observed with the increase of halogen size showing a larger out-of-plane distortion, whilst the disubstitution mode having a larger impact in general. Interestingly, it appears that the effect of disubstitution is more pronounced than that of the mono-substitution with the immediate larger halogen. For example, di-Cl (**1:10**) has a larger distortion than the mono-substituted bromoporphyrin (**1D**). In the in-plane distortion modes, the story is much simpler. The introduction of a metal center reduced the overall impact in the in-plane distortion modes until this is alleviated by the effect of a larger halogen. Increasing the halogen size shows a larger increase in the *A1g* contribution which helps alleviate the decrease in *B2g* contributions as a result of Ni(II) insertion. As for bond lengths and angles, there are little differences observed due to the increase in halogen size or the number of substituents. It is only in the Δ24 and the pyrrole tilt angles both of which decrease with larger halogen atoms. Comparing mono- to disubstitutions the general trend is a decrease in pyrrole tilt angle and Δ24.

Finally, when comparing the calculated data (**1D**–**3D**) to that obtained via crystal structure analysis, there are small discrepancies (Supporting Information File 2, Table S14). In essence, the calculated data is a good approximation; however, the use of an idealized structure rather than an actual structure loses some of the accuracies that trends in NSD and other crystallographic explorations rely on. For example, most of the calculated data is only accurate to the first second decimal place while in some cases large differences are observed. This is most apparent when looking at the Δ24 of **3** and **3D** where and difference of 5 times is observed between the simulated and crystal data. A clear trend is observed for the dihedral angle between the porphyrin and the phenyl rings. This dihedral angle is 69.2° for the unsubstituted diphenyl and increases as the halogen size and number of substituents increases, becoming almost perpendicular for the di-iodo substituted (87.6°) (Supporting Information File 2, Table S8). Interestingly, this trend disappears when the metal is inserted, as a dihedral angle of approximately 81° is observed regardless of substitution. In conclusion, while the more desirable method is to use the actual crystal structures, the use of calculated data for structures that do not exist currently in the CSD is a good approximation of the actual structure.

#### Series 2

In this series, we considered progressively increasing the number of halogens, taking 5-phenylporphyrin as the base, and analyzed the effect on in-plane and out-of-plane distortions (Figure 23). With the addition of one to three bromine atoms to the free meso-positions of the porphyrin, there is only a marginal shift in the out-of-plane distortion modes, with 5,10,15-substituted derivative having the largest (**2:6**) and 5,10-substituted derivative having the smallest contributions (**1:11**) (Supporting Information File 1, Figure S73, and Supporting Information File 2, Table S15). The largest of these contributions is seen in the *B2u* mode with a smaller but equal contribution to the wave x and y modes. This is also represented in the bond lengths and angles with only minor deviations apparent (Supporting Information File 2, Table S15). For the in-plane distortion modes, while there is a larger contribution than seen in the out-of-plane modes, there is little difference by increasing the number of bromine atoms. The most obvious change is seen in the *B2g* mode where the largest contribution is **2:1** with **2:3** being the second largest. Both **2:2** and **2:4** have much smaller contributions. The largest contributions for the in-plane is seen in the *A1g* modes where a small increase from **2:1** to **2:4** is observed.

With the addition of the phenyl group to this series, there are much more apparent changes in both the in-plane and out-of-plane distortion modes. The structure of **2:5** and **2:6** (15,20-diphenyl) both show a marked increase in the out-of-plane distortion with the largest contribution seen in the *B2u* and *Eg*(*y*) modes. With the second addition of a bromine atom (**2:6**), there is a much larger contribution to the *B2g* mode while no change is observed in the *Eg*(*y*) mode. In the in-plane modes, there is a minor decrease in the DIP, which is due to a complete absence of a contribution in the *B2g* mode seen in compound **2:6** while both have almost equal contributions in the *A1g* mode. Moving towards the 10,20-diphenyl derivatives (**1D** and **1:11**), it appears that these structures are more comparable to the **2:1**–**2:4**. However, instead of the *B2u* being the largest mode, the contributions are seen in the *Eg*(*x*) and *Eg*(*y*) modes. In the in-plane mode, the largest contribution is seen in the *A1g* with a secondary contribution in the *B2g*. This indicates that while there is a minor effect of increasing the number of substituents, a larger impact is seen by the phenyl derivatives. Therefore, the halogen substituents only marginally impact the porphyrin core with the in-plane demonstrating the largest shift.

#### Series 3

When considering the 5,10-di-halo-15,20-diphenyl substituted porphyrins (Figure 24) there is a clear trend with regards to the influence of increasing halogen size on bond lengths, angles, and out-of-plane displacements (Supporting Information File 2, Table S16). The general trend is a marginal increase in the bond lengths and angles with the fluorine derivative being the lowest and iodine being the largest suggesting the influence of larger halogens has only a minor increasing effect. In the atom displacement, this trend is continued except for ΔN decreasing from fluorine to iodine derivatives. A clearer picture emerges when examining the NSD charts (Supporting Information File 1, Figure S74).

In the distortion modes, the steady increase from fluorine to iodine is evident in both the in-plane and out-of-plane NSD profiles. This is due to the increased *Eu*(*x*), (pyr-translation), and *A1g* (breathing) contribution and a drop in the *B1g* (*N*-stretching) in the in-plane distortions. An increase in the *B2u* (saddle) distortion mode out-of-plane is accompanied by a minor decrease in the *A2u* (domed) and *Eg*(*y*) (wave y) modes. This indicates that the introduction of halogen atoms to the 5,15-position on the porphyrin macrocycle increases the saddling present, compared to 5,15-derivatives as mentioned above. However, there is only a marginal impact on this by introducing larger halogen atoms.

## Conclusion

In the simplest models (5-halo examples), increasing halogen size changes the overall packing and moves from a close packing arrangement to a directional stacking within the crystal structure. While there are no obvious interactions between halogens, there does appear to be a marked increase with contacts between larger halogens and the porphyrin ring. From a conformational standpoint, most bond lengths and angles are not significantly altered by increasing the halogen size; however, there is a noticeable increase in the atom displacements, which increases distortion. However, these effects are not as considerable as the introduction of a nickel(II) metal center to the porphyrin core. Additionally, it is apparent that while the halogen may be a large part of each of these structures, it is highly dependent on the other substituents present in forming the interaction profile. For example, phenyl moieties generally form close packing complexes rather than direct interactions and alkyl moieties form a plethora of halogen···hydrogen interactions. Additionally, the substitution pattern greatly affects the overall conformation. By comparing the DFT calculated geometries with crystal structures we have found that DFT is a good approximation. The use of an idealized structure results in the loss of some accuracy as seen in the NSD. Most of the DFT calculated data is only accurate to the first or second decimal while in other cases larger differences are observed. While the more desirable method is to use the actual crystal structures, the use of DFT calculated geometries for structures that currently do not exist in the CSD is a good approximation.

Future work in this area should focus on optimizing the DFT calculations in order to minimize errors and expanding on the investigations done using this method. Furthermore, we can focus on increasing the library of simulated data and use this as a model to predict the most appealing structures for generating halogen bonded supramolecular networks followed by targeted synthesis and crystallization of such compounds.

## Experimental

### General information

All commercial chemicals used were of analytical grade and supplied by Sigma-Aldrich, Frontier Scientific, Inc., Tokyo Chemical Company and Acros chemicals and used without further purification unless otherwise stated. Flash column chromatography was carried out using Fluka Silica Gel 60 (230–400 mesh; Merck. Melting points are uncorrected and were measured with a Stuart SP-10 melting point apparatus. A Bruker Advance III 400 MHz spectrometer was employed for ^1^H (400.13 MHz), and ^13^C (100.61 MHz) NMR spectra. All NMR experiments were performed at room temperature. Mass spectrometry analysis was performed with a Q-Tof Premier Waters MALDI quadrupole time-of-flight (Q-TOF) mass spectrometer equipped with Z-spray electrospray ionization (ESI) and matrix-assisted laser desorption ionization (MALDI) sources either in a positive or negative mode with DCTB (*trans*-2-[3-(4-*tert*-butylphenyl)-2-methyl-2-propenylidene]malononitrile) as the matrix. UV-visible absorption measurements were performed using a Shimadzu MultiSpec-1501.

### Synthetic procedures

The synthesis of 5-bromo-10,20-diphenylporphyrin (**1**) [46], [5-ethynyl-10,20-diphenylporphyrinato]nickel(II) (**2A**) [47], 5-bromo-10,20-dihexyl-15-phenylporphyrin (**5**) [48], [5-bromo-10,20-dihexyl-15-phenylporphyrinato]nickel(II) (**6**) [48], 5-bromo-10,15,20-triphenylporphyrin (**7**) [49], 5-iodo-10,20-dihexyl-15-phenylporphyrin (**8**) [48], 5,15-dibromo-10,20-diphenylporphyrin (**9**) [50], 5,15-dibromo-10,20-bis(4-methylphenyl)porphyrin (**10**) [51], [5,15-dibromo-10,20-di(pentan-3-yl)porphyrinato]nickel(II) (**11**) [52], 5,15-dibromo-10-hexyl-20-(3,4,5-trimethoxyphenyl)porphyrin (**13**) [24], 5,10-bis(4-methylphenyl)-15,20-bis((trimethylsilyl)ethynyl)porphyrin (**16A**) [53], and 10,15,20-trihexyl-5-((trimethylsilyl)ethynyl) porphyrin (**24**) [54] have previously been reported.

**Synthesis of 5-chloro-10,20-bis(4-methylphenyl)-15-phenylporphyrin (4)** Pre-dried 5,15-bis(4-methylphenyl)porphyrin, (100 mg, 0.2 mmol, 1 equiv) was dissolved in 37 mL dry THF. A solution of 0.7 mL PhLi (1.8 M in dibutyl ether, 1.28 mmol, 6.4 equiv) was added at 0 °C under stirring, resulting in a color change from purple to green/brown. Following the complete addition, the cold bath was removed and stirring was continued for an additional 45 min. Subsequently, pre-dried DDQ (291 mg, 1.28 mmol, 6.4 equiv) was added, resulting in a color change from green/brown to dark purple/brown. Stirring was continued for 1 h, and the solvent was removed under reduced pressure. The residue was filtered through silica gel using DCM. Column chromatography was performed through silica gel (hexane/DCM, 15:1–2:1, v/v). Further column chromatography was carried out on silica gel (hexane/DCM, 15:1–1:1, v/v) yielding the purple solid **4** (51 mg, 51%). To obtain single crystals, recrystallization from DCM and methanol was performed and gave purple crystals of the title compound. Mp > 300 °C; *R*_f_ = 0.48 (SiO_2_, hexane/DCM, 2:1, v/ v); ^1^H NMR (400 MHz, CDCl_3_) δ −2.69 (s, 2H, NH), −2.72 (s, 6H, CH_3_), 7.57 (d, ^3^*J* = 7.7 Hz, 4H, H*_m_*_-tolyl_), 7.72–7.79 (m, 3H, H*_m_*_-phenyl_ and H*_p_*_-phenyl_), 8.08 (d, ^3^*J* = 7.9 Hz, 4H, H*_o_*_-tolyl_), 8.18 (d, ^3^*J* = 6.5 Hz, 2H, H*_o_*_-phenyl_), 8.78 (d, ^3^*J* = 4.7 Hz, 2H, H_β-pyrr_), 8.84 (d, ^3^*J* = 4.7 Hz, 2H, H_β-pyrr_), 8.94 (d, ^3^*J* = 4.7 Hz, 2H, H_β-pyrr_) 9.64 ppm (d, ^3^*J* = 4.7 Hz, 2H, H_β-pyrr_); ^13^C NMR (100 MHz, CDCl_3_) δ 112.2, 120.7, 120.9, 126.8, 127.7, 127.8, 127.9, 134.6, 134.7, 134.8, 137.7, 138.9, 142.1 ppm; UV–vis (DCM) λ_max_ (log ε) 420 (5.30), 519 (3.91), 555 (3.68), 596 (3.38) nm; HRMS−MALDI (*m*/*z*): [M^+^] calcd for C_40_H_29_ClN_4_, 600.2081; found, 600.2050.

#### Note on synthesis of chloro derivatives of porphyrins

The reaction entailed an attempt to prepare a meso–meso linked bisporophyrin via reaction of the starting material with PhLi followed by in situ oxidation to a radical anion and dimerization [55]. While formation of 5,10,15-trisubstituted porphyrins and 5,10,15,20-tetrasubstituted porphyrins was anticipated as side products, the detection of the title compound **4** was unexpected. The mechanism of the formation of **4** is not easy to clarify, and presently, there is no indication of whether the reaction proceeds via a homolytic or heterolytic process. Notably, similar unexpected chlorination reactions have been described in the past [56–57]. While the metallated form of the product has been previously reported [56], this appears to be the first instance of the free base counterpart. One such study, using (5,15-bis(4-methylphenyl)porphyrinato)zinc(II), alludes to nucleophilic substitution by Cl to form the chlorinated product, with the only possible source of the exogenous Cl atom during the study being DCM [56]. Another study suggests that the chlorinated product may be formed from a Cl radical generated during the reaction, although it was not stated if the origin of the radical was DCM or DDQ [57]. Here, it was shown that the source of Cl to yield the meso-chlorinated porphyrin was DDQ, given the absence of DCM. Investigations into why similar compounds as **4** were not frequently observed in previous research, and into the targeted synthesis of species **4** by DDQ are currently ongoing. Alternative methods to achieve the chloro derivatives of porphyrins have been reported by Dimé et al. [58].

### Crystallography

Crystals were grown using techniques following the protocol developed by Hope [59]. Crystals were either grown by allowing the solvent to slowly evaporate over time or by a liquid:liquid diffusion process. The crystal was mounted on a MiTeGen MicroMount and single-crystal X-ray diffraction data were collected on a Bruker APEX 2 DUO CCD, Rigaku CCD, or Bruker D8 Quest ECO diffractometer using graphite-monochromated Mo K_α_ (λ = 0.71073 Å) and Incoatec IμS Cu K_α_ (λ = 1.54178 Å) radiation at 100(2), 112(2), and 123(2) K with an Oxford Cryosystems Cobra low-temperature device. Data were collected by using omega and phi scans and were corrected for Lorentz and polarization effects by using the APEX software suite [60–63]. Using Olex2, the structure was solved with the XS or XT structure solution program, using direct methods and refined against |*F*^2^| with XL using least-squares minimization [64–65]. The C- and N-bound H atoms were placed in their expected calculated positions and refined as riding model: N–H = 0.88 Å, C–H = 0.95–0.98 Å, with U*_iso_* (H) = 1.5U*_eq_* (C) for methyl H atoms and 1.2U*_eq_* (C, N) for all other atoms other H atoms. Details of data refinements can be found in Supporting Information File 2, Tables S17–S20. All images were prepared by using Olex2 [63].

CCDC 2069451–2069463 contain the supplementary crystallographic data for this paper. These data can be obtained free of charge from The Cambridge Crystallographic Data Centre via http://www.ccdc.cam.ac.uk/data_request/cif.

#### Details on refinement and modelling

Compound **1**: The bromine atoms were modelled over both meso positions in a 96:4% occupancy. The phenyl group (C201–C206) was modelled over two position in a 60:40% occupancy and fixed using the restraint ISOR and SADI. The internal nitrogen atoms were modelled over two positions in a 50:50% occupancy. Compound **2A**: No modelling necessary. Compound **4**: The internal nitrogen atoms were modelled over two positions in a 50% occupancy. Compound **5**: The internal nitrogen atoms were modelled over two positions in a 90:10% occupancy. The terminal carbon atom of the hexyl chain (C106) was modelled over two positions using restraints SIMU in a 50% occupancy. The phenyl group was modelled over two positions using restraints ISOR, SIMU, and SADI in a 50% occupancy. Compound **6**: No modelling necessary. Compound **7**: The internal nitrogen atoms were modelled over two positions in a 50% occupancy. Compound **8**: The iodine atom I1_2 was modelled over two positions using restraints SADI and constraint EADP in an 80:20% occupancy. The phenyl group (C151_2–C156_2) was modelled over two positions using restraints SADI and constraint EADP in a 50% occupancy. The phenyl group (C151_1–C156_1) was modelled over two positions using restraints SADI and constraint EADP in a 50% occupancy. The internal nitrogen atoms were modelled over two positions in a 50% occupancy. Compound **9**: The internal nitrogen atoms were modelled over two positions in a 50% occupancy. Compound **10**: No modelling necessary. Compound **11**: The pentan-3-yl moiety (C104–C106) was modelled over two position using restraints SIMU in a 50% occupancy. Compound **13**: The internal nitrogen atoms were modelled over two positions in a 50% occupancy. Compound **16A**: The internal nitrogen atoms were modelled over two positions in a 75:25% occupancy. Compound **24**: The internal nitrogen atoms were modelled over two positions in a 50% occupancy. The methyl groups of the TMS moiety were modelled over two positions using restraints SIMU and SADI in an 80:20% occupancy.

#### Previous structures

Details on the refinement of NESHUO (**2**) [27], UDERUR (**3**) [22], NOGWEN (**12**) [23], HUMWES (**13A**) [24], HUMWAO (**14**) [24], LASMOK (**15**) [33], RAKGAN (**16**) [34], ZOXQUA (**17**) [35], ZOXCAS (**18**) [35], BASDOR (**19**) [36], YISZAD(**20**) [39], QUGMEM (**21**) [38], QUGMIQ (**22**) [38], and MORBEC (**23**) [37] were previously reported.

#### Hirshfeld surface analysis

The two-dimensional fingerprint plots and associated Hirshfeld surfaces [26,66] were calculated using CrystalExplorer [67]. The intermolecular contacts in crystal packing were visualized using d_norm_ surface. The di(outside) and de(outside) represent the distance to the Hirshfeld surface from nuclei. The proportional contribution of the contacts over the surface is visualized by the color gradient (blue to green) in the fingerprint plots.

### Normal-coordinate structural decomposition (NSD) analysis

The theoretical background and development of this method have been described by Shelnutt and co-workers [68–69]. NSD is a method that employs the decomposition of the conformation of the macrocycle by a basis set composed of its various normal modes of vibration, affording clear separation of the contributing distortions to the macrocycle conformation in a quantitative fashion. For calculations, we used our newly developed NSD generation program [28,45]. Supporting Information File 2, Tables S21–S23 contain the complete NSD out-put of compounds herein. Supporting Information File 1, Figures S75–S129 contain the file out-put of all compounds within this paper as obtained from the program.

### DFT calculations

The DFT calculations were performed using Gaussian 16 [70]. The wB97XD functional and a cc-pVDZ (cc-pVDZ-PP for iodine) basis set were used to optimize the ground state (S0) geometries using a tight convergence criterion. Frequency calculations were performed at the optimized geometries in order to confirm that the local minima were found. The molecular electrostatic potential (MEP) surfaces were generated by mapping the electrostatic potentials onto the 0.001 e/au^3^ molecular electron density surfaces using the VMD software [71].

## Supporting Information

File 1 Supplementary graphics.

File 2 Supplementary tables.

File 3 Crystal structure determination compounds **1**, **2a**, **4**–**11**, **13**, **16a** and **24**.
