# Supplementary material for: Structural effects of meso-halogenation on porphyrins
Source: Beilstein J Org Chem. 2021 May 14;17:1149–70. doi: 10.3762/bjoc.17.88 (PMC8144917; doi:10.3762/bjoc.17.88)
Supplement: File 1 — Supplementary graphics. [file Beilstein_J_Org_Chem-17-1149-s001.pdf]

BEILSTEIN JOURNAL OF ORGANIC CHEMISTRY

Supporting Information

for

Structural effects of meso-halogenation on porphyrins

Keith J. Flanagan, Maximilian Paradiz Dominguez, Zoi Melissari, Hans-Georg Eckhardt, René M. Williams, Dáire Gibbons, Caroline Prior, Gemma M. Locke, Alina Meindl, Aoife A. Ryan and Mathias O. Senge

Beilstein J. Org. Chem. **2021,** *17,* 1149–1170. doi:10.3762/bjoc.17.88

Supplementary graphics

License and Terms: This is a supporting information file under the terms of the Creative Commons Attribution License (https://creativecommons.org/licenses/by/4.0). Please note that the reuse, redistribution and reproduction in particular requires that the author(s) and source are credited and that individual graphics may be subject to special legal provisions.
The license is subject to the *Beilstein Journal of Organic Chemistry* terms and conditions: (https://www.beilstein-journals.org/bjoc/terms)

Supporting Information

Table of Contents

Figures from X-ray crystallography and DFT. S9

Figure S1: Thermal ellipsoid plot of compound **1** (thermal displacement given as 50% probability). Minor disordered moieties have been omitted. S9

Figure S2: Packing diagram (thermal ellipsoid plot) of compound **1** looking down the *b*-axis (thermal displacement given as 50% probability). Minor disordered moieties have been omitted. S9

Figure S3: Thermal ellipsoid plot of compound **2A** (thermal displacement given as 50% probability). Minor disordered moieties have been omitted. S10

Figure S4: Packing diagram (thermal ellipsoid plot) of compound **2A** looking down the *b*-axis (thermal displacement given as 50% probability). Minor disordered moieties have been omitted. S10

Figure S5: Packing diagram (ball and stick) of compound **2** looking down the *a*-axis. S11

Figure S6: Packing diagram (ball and stick) of compound **3** looking down the *a*-axis. S11

Figure S7: Thermal ellipsoid plot of compound **4** (thermal displacement given as 50% probability). Minor disordered moieties have been omitted. S12

Figure S8: Packing diagram (thermal ellipsoid plot) of compound **4** looking down the *b*-axis (thermal displacement given as 50% probability). Minor disordered moieties have been omitted. S12

Figure S9: Thermal ellipsoid plot of compound **5** (thermal displacement given as 50% probability). Minor disordered moieties have been omitted. S13

Figure S10: Packing diagram (thermal ellipsoid plot) of compound **5** looking down the *a*-axis (thermal displacement given as 50% probability). Minor disordered moieties have been omitted. S13

Figure S11: Thermal ellipsoid plot of compound **6** (thermal displacement given as 50% probability). Minor disordered moieties have been omitted. S14

Figure S12: Packing diagram (thermal ellipsoid plot) of compound **6** looking down the *a*-axis (thermal displacement given as 50% probability). Minor disordered moieties have been omitted. S14

Figure S13: Thermal ellipsoid plot of compound **7** (thermal displacement given as 50% probability). Minor disordered moieties have been omitted. S15

Figure S14: Packing diagram (thermal ellipsoid plot) of compound **7** looking down the *a*-axis (thermal displacement given as 50% probability). Minor disordered moieties have been omitted. S15

Figure S15: Thermal ellipsoid plot of compound **8** (thermal displacement given as 50% probability). Minor disordered moieties have been omitted. #_1 and #_2 indicate the residue of each molecule in the asymmetric unit. S16

Figure S16: Packing diagram (thermal ellipsoid plot) of compound **8** looking down the *a*-axis (thermal displacement given as 50% probability). Minor disordered moieties have been omitted. S16

Figure S17: NSD charts for compounds **4**–**8**. S17

Figure S18: Expanded view (thermal ellipsoid plot) of compound **8** showing the stacking between porphyrin macrocycles. S18

Figure S19: Expanded view (thermal ellipsoid plot) of compound **8** showing the close packing between (**A**) pyrrole and iodine (**B**) hexyl and iodine. #_X indicated the residue number. S18

Figure S20: Hirshfeld surfaces of compounds **4**–**8**. S19

Figure S21: Contact percentages of compounds **4**–**8**. S20

Figure S22: Thermal ellipsoid plot of compound **9** (thermal displacement given as 50% probability). Minor disordered moieties have been omitted. S20

Figure S23: Packing diagram (thermal ellipsoid plot) of compound **9** looking down the *b*-axis (thermal displacement given as 50% probability). Minor disordered moieties have been omitted. S21

Figure S24: Thermal ellipsoid plot of compound **10** (thermal displacement given as 50% probability). Minor disordered moieties have been omitted. S21

Figure S25: Packing diagram (thermal ellipsoid plot) of compound **10** looking down the *b*-axis (thermal displacement given as 50% probability). Minor disordered moieties have been omitted. S22

Figure S26: Thermal ellipsoid plot of compound **11** (thermal displacement given as 50% probability). Minor disordered moieties have been omitted. S23

Figure S27: Packing diagram (thermal ellipsoid plot) of compound **11** looking down the *b*-axis (thermal displacement given as 50% probability). Minor disordered moieties have been omitted. S23

Figure S28: Thermal ellipsoid plot of compound **13** (thermal displacement given as 50% probability). Minor disordered moieties have been omitted. S24

Figure S29: Packing diagram (thermal ellipsoid plot) of compound **13** looking down the *b*-axis (thermal displacement given as 50% probability). Minor disordered moieties have been omitted. S24

Figure S30: Expanded view (thermal ellipsoid plot) of compound **9** showing the combination of interactions seen in Figure S37. .... S25

Figure S31: NSD charts for compounds **9**–**15**. S26

Figure S32: Expanded view (ball and stick) of compound **12** showing the (**A**) alkyl chain interactions (**B**) porphyrin stacking (**C**) close packing side-on alignment. S27

Figure S33: Packing diagram (ball and stick) of compound **12** looking down the *a*-axis. S28

Figure S34: Expanded view (ball and stick) of compound **12** showing the head-to-head Br···H contact. S28

Figure S35: Packing diagrams for compound **13** (thermal ellipsoid plot) (**A**) and **13A** (ball and stick) (**B**) looking down the *b*-axis. S29

Figure S36: Expanded view (ball and stick) of compound **14** showing (**A**) Br···H interactions with pyrrole units, methoxy group, and the hexyl chains (**B**) Br···H interactions with methoxy group. S29

Figure S37: Packing diagrams (ball and stick) of compound **14** looking down the *b*-axis. S30

Figure S38: Expanded view (ball and stick) of compound **15** showing the above (**A**) and below (**B**) plane interactions between porphyrins.. S30

Figure S39: Expanded view (ball and stick) of compound **15** showing face-to-edge (**A**) and hydrogen bonded network (**B**).. S31

Figure S40: Packing diagrams (ball and stick) for compound **15** looking down the *b*-axis............ S31

Figure S41: Hirshfeld surfaces of compounds **9**-**15**. S32

Figure S42: Contact percentages of compounds **9**-**15**.. S33

Figure S43: Thermal ellipsoid plot of compound **16A** (thermal displacement given as 50% probability). Minor disordered moieties have been omitted. S33

Figure S44: Packing diagram (thermal ellipsoid plot) of compound **16A** looking down the *c*-axis (thermal displacement given as 50% probability). Minor disordered moieties have been omitted. S34

Figure S45: Packing diagram (ball and stick) of compound **16** looking down the *b*-axis............... S34

Figure S46: Expanded view (thermal ellipsoid plot) of compound **16A** showing (**A**) linear alignment and (**B**) stacking.. S35

Figure S47: Expanded view (ball and stick) of compound **17** showing the (**A**) stacking and (**B**) head-to-tail alignment. S35

Figure S48: Packing diagram (ball and stick) of compound **17** looking down the *c*-axis. S35

Figure S49: Expanded view (ball and stick) of compound **18** showing the (**A**) head-to-tail alignment and (**B**) interaction between the axial ligand and the bromine atoms... S36

Figure S50: Expanded view (ball and stick) of compound **18** showing the stacking between (**A**) solvent side of the porphyrin and (**B**) non-solvent side.. S36

Figure S51: Packing diagram (ball and stick) of compound **18** looking down the *b*-axis............... S36

Figure S52: Expanded view (ball and stick) of compound **19** showing the stepwise alignment of porphyrins with Br···H interactions (**A**) top and (**B**) side. S37

Figure S53: Expanded view (ball and stick) of compound **19** showing the (**A**) top view of stacking and (**B**) edge-on view of stacking.. S37

Figure S54: Packing diagram (ball and stick) of compound **19** looking down the *b*-axis............... S38

Figure S55: Hirshfeld surfaces and contact percentages of compounds **16**-**19**. S39

Figure S56: NSD charts for compounds **16**-**19**.. S40

Figure S57: (**A**) Expanded view (ball and stick) of compound **20** showing the F···H interaction and (**B**) crystal packing (ball and stick) of compound **20**. S41

Figure S58: (**A**) Expanded view (ball and stick) of compound **21** showing the head-to-head interaction (**B**) Expanded view (ball and stick) of compound **22** showing the face-to-edge interaction.. S41

Figure S59: Packing diagram (ball and stick) of compound **21** looking down the *a*-axis (**A**) and **22** looking down the *b*-axis (**B**). S42

Figure S60: Expanded view (ball and stick) of compound **23** showing the head-to-tail interaction. S42

Figure S61: Packing diagram (ball and stick) of compound **23** looking down the *b*-axis........ S43

Figure S62: Thermal ellipsoid plot of compound **24** (thermal displacement given as 50% probability). Minor disordered moieties have been omitted........ S43

Figure S63: Packing diagram (thermal ellipsoid plot) of compound **24** looking down the *a*-axis (thermal displacement given as 50% probability). Minor disordered moieties have been omitted. S44

Figure S64: DFT generated images of compounds **1:1-1:4** (**A**) MEP map top view, (**B**) MEP map side view, (**C**) electron density distribution of HOMO, (**D**) electron density distribution of LUMO. S45

Figure S65: DFT generated images of compounds **1:5-1:8** (**A**) MEP map top view, (**B**) MEP map side view, (**C**) electron density distribution of HOMO, (**D**) electron density distribution of LUMO. S45

Figure S66: DFT generated images of compounds **1:9**, **1:10**, **1D**, and **2D** (**A**) MEP map top view, (**B**) MEP map side view, (**C**) electron density distribution of HOMO, (**D**) electron density distribution of LUMO. S46

Figure S67: DFT generated images of compounds **1:11-1:13** and **3D** (**A**) MEP map top view, (**B**) MEP map side view, (**C**) electron density distribution of HOMO, (**D**) electron density distribution of LUMO. S46

Figure S68: DFT generated images of compounds **1:14** and **1:15** (**A**) MEP map top view, (**B**) MEP map side view, (**C**) electron density distribution of HOMO, (**D**) electron density distribution of LUMO. S47

Figure S69: DFT generated images of compounds **2:1-2:4** (**A**) MEP map top view, (**B**) MEP map side view, (**C**) electron density distribution of HOMO, (**D**) electron density distribution of LUMO. S47

Figure S70: DFT generated images of compounds **2:5**, **2:6**, **1D**, and **1:11** (**A**) MEP map top view, (**B**) MEP map side view, (**C**) electron density distribution of HOMO, (**D**) electron density distribution of LUMO. S48

Figure S71: DFT generated images of compounds **3:1-3:3** and **2:6** (**A**) MEP map top view, (**B**) MEP map side view, (**C**) electron density distribution of HOMO, (**D**) electron density distribution of LUMO. S48

Figure S72: NSD charts for compounds **1:1-1:15**........ S49

Figure S73: NSD charts for compounds **2:1-2:6**, **1D**, and **1:11**. S50

Figure S74: NSD charts for compounds **3:1-3:3** and **2:6**........ S51

NSD tables and plots for crystal structures........ S52

Figure S75: NSD result generated from **1** (in Å) (**A**) in-plane and (**B**) out-of-plane skeletal plots of the porphyrin core. Porphyrin is represented in black (C) and blue (N), with the reference structure (CuTPP) in red dotted lines. S52

Figure S76: NSD result generated from **2** (in Å) (**A**) in-plane and (**B**) out-of-plane skeletal plots of the porphyrin core. Porphyrin is represented in black (C) and blue (N), with the reference structure (CuTPP) in red dotted lines. S53

Figure S77: NSD result generated from **2A** (in Å) (**A**) in-plane and (**B**) out-of-plane skeletal plots of the porphyrin core. Porphyrin is represented in black (C) and blue (N), with the reference structure (CuTPP) in red dotted lines. S54

Figure S78: NSD result generated from **3** (in Å) (**A**) in-plane and (**B**) out-of-plane skeletal plots of the porphyrin core. Porphyrin is represented in black (C) and blue (N), with the reference structure (CuTPP) in red dotted lines. S55

Figure S79: NSD result generated from **4** (in Å) (**A**) in-plane and (**B**) out-of-plane skeletal plots of the porphyrin core. Porphyrin is represented in black (C) and blue (N), with the reference structure (CuTPP) in red dotted lines. S56

Figure S80: NSD result generated from **5** (in Å) (**A**) in-plane and (**B**) out-of-plane skeletal plots of the porphyrin core. Porphyrin is represented in black (C) and blue (N), with the reference structure (CuTPP) in red dotted lines. S57

Figure S81: NSD result generated from **6** (in Å) (**A**) in-plane and (**B**) out-of-plane skeletal plots of the porphyrin core. Porphyrin is represented in black (C) and blue (N), with the reference structure (CuTPP) in red dotted lines. S58

Figure S82: NSD result generated from **7** (in Å) (**A**) in-plane and (**B**) out-of-plane skeletal plots of the porphyrin core. Porphyrin is represented in black (C) and blue (N), with the reference structure (CuTPP) in red dotted lines. S59

Figure S83: NSD result generated from **8_1** (in Å) (**A**) in-plane and (**B**) out-of-plane skeletal plots of the porphyrin core. Porphyrin is represented in black (C) and blue (N), with the reference structure (CuTPP) in red dotted lines. S60

Figure S84: NSD result generated from **8_2** (in Å) (**A**) in-plane and (**B**) out-of-plane skeletal plots of the porphyrin core. Porphyrin is represented in black (C) and blue (N), with the reference structure (CuTPP) in red dotted lines. S61

Figure S85: NSD result generated from **9** (in Å) (**A**) in-plane and (**B**) out-of-plane skeletal plots of the porphyrin core. Porphyrin is represented in black (C) and blue (N), with the reference structure (CuTPP) in red dotted lines. S62

Figure S86: NSD result generated from **10** (in Å) (**A**) in-plane and (**B**) out-of-plane skeletal plots of the porphyrin core. Porphyrin is represented in black (C) and blue (N), with the reference structure (CuTPP) in red dotted lines. S63

Figure S87: NSD result generated from **11** (in Å) (**A**) in-plane and (**B**) out-of-plane skeletal plots of the porphyrin core. Porphyrin is represented in black (C) and blue (N), with the reference structure (CuTPP) in red dotted lines. S64

Figure S88: NSD result generated from **12** (in Å) (**A**) in-plane and (**B**) out-of-plane skeletal plots of the porphyrin core. Porphyrin is represented in black (C) and blue (N), with the reference structure (CuTPP) in red dotted lines. S65

Figure S89: NSD result generated from **13** (in Å) (**A**) in-plane and (**B**) out-of-plane skeletal plots of the porphyrin core. Porphyrin is represented in black (C) and blue (N), with the reference structure (CuTPP) in red dotted lines. S66

Figure S90: NSD result generated from **13A** (in Å) (**A**) in-plane and (**B**) out-of-plane skeletal plots of the porphyrin core. Porphyrin is represented in black (C) and blue (N), with the reference structure (CuTPP) in red dotted lines. S67

Figure S91: NSD result generated from **14** (in Å) (**A**) in-plane and (**B**) out-of-plane skeletal plots of the porphyrin core. Porphyrin is represented in black (C) and blue (N), with the reference structure (CuTPP) in red dotted lines. S68

Figure S92: NSD result generated from **15** (in Å) (**A**) in-plane and (**B**) out-of-plane skeletal plots of the porphyrin core. Porphyrin is represented in black (C) and blue (N), with the reference structure (CuTPP) in red dotted lines. S69

Figure S93: NSD result generated from **16** (in Å) (**A**) in-plane and (**B**) out-of-plane skeletal plots of the porphyrin core. Porphyrin is represented in black (C) and blue (N), with the reference structure (CuTPP) in red dotted lines. S70

Figure S94: NSD result generated from **16A** (in Å) (**A**) in-plane and (**B**) out-of-plane skeletal plots of the porphyrin core. Porphyrin is represented in black (C) and blue (N), with the reference structure (CuTPP) in red dotted lines. S71

Figure S95: NSD result generated from **17** (in Å) (**A**) in-plane and (**B**) out-of-plane skeletal plots of the porphyrin core. Porphyrin is represented in black (C) and blue (N), with the reference structure (CuTPP) in red dotted lines. S72

Figure S96: NSD result generated from **18** (in Å) (**A**) in-plane and (**B**) out-of-plane skeletal plots of the porphyrin core. Porphyrin is represented in black (C) and blue (N), with the reference structure (CuTPP) in red dotted lines. S73

Figure S97: NSD result generated from **19** (in Å) (**A**) in-plane and (**B**) out-of-plane skeletal plots of the porphyrin core. Porphyrin is represented in black (C) and blue (N), with the reference structure (CuTPP) in red dotted lines. S74

Figure S98: NSD result generated from **20** (in Å) (**A**) in-plane and (**B**) out-of-plane skeletal plots of the porphyrin core. Porphyrin is represented in black (C) and blue (N), with the reference structure (CuTPP) in red dotted lines. S75

Figure S99: NSD result generated from **21** (in Å) (**A**) in-plane and (**B**) out-of-plane skeletal plots of the porphyrin core. Porphyrin is represented in black (C) and blue (N), with the reference structure (CuTPP) in red dotted lines. S76

Figure S100: NSD result generated from **22** (in Å) (**A**) in-plane and (**B**) out-of-plane skeletal plots of the porphyrin core. Porphyrin is represented in black (C) and blue (N), with the reference structure (CuTPP) in red dotted lines. S77

Figure S101: NSD result generated from **23** (in Å) (**A**) in-plane and (**B**) out-of-plane skeletal plots of the porphyrin core. Porphyrin is represented in black (C) and blue (N), with the reference structure (CuTPP) in red dotted lines. S78

Figure S102: NSD result generated from **24** (in Å) (**A**) in-plane and (**B**) out-of-plane skeletal plots of the porphyrin core. Porphyrin is represented in black (C) and blue (N), with the reference structure (CuTPP) in red dotted lines. S79

NSD tables and plots for DFT structures series 1 .... S80

Figure S103: NSD result generated from **1:1** (in Å) (**A**) in-plane and (**B**) out-of-plane skeletal plots of the porphyrin core. Porphyrin is represented in black (C) and blue (N), with the reference structure (CuTPP) in red dotted lines. S80

Figure S104: NSD result generated from **1:2** (in Å) (**A**) in-plane and (**B**) out-of-plane skeletal plots of the porphyrin core. Porphyrin is represented in black (C) and blue (N), with the reference structure (CuTPP) in red dotted lines. S81

Figure S105: NSD result generated from **1:3** (in Å) (**A**) in-plane and (**B**) out-of-plane skeletal plots of the porphyrin core. Porphyrin is represented in black (C) and blue (N), with the reference structure (CuTPP) in red dotted lines. S82

Figure S106: NSD result generated from **1:4** (in Å) (**A**) in-plane and (**B**) out-of-plane skeletal plots of the porphyrin core. Porphyrin is represented in black (C) and blue (N), with the reference structure (CuTPP) in red dotted lines. S83

Figure S107: NSD result generated from **1:5** (in Å) (**A**) in-plane and (**B**) out-of-plane skeletal plots of the porphyrin core. Porphyrin is represented in black (C) and blue (N), with the reference structure (CuTPP) in red dotted lines. S84

Figure S108: NSD result generated from **1:6** (in Å) (**A**) in-plane and (**B**) out-of-plane skeletal plots of the porphyrin core. Porphyrin is represented in black (C) and blue (N), with the reference structure (CuTPP) in red dotted lines. S85

Figure S109: NSD result generated from **1:7** (in Å) (**A**) in-plane and (**B**) out-of-plane skeletal plots of the porphyrin core. Porphyrin is represented in black (C) and blue (N), with the reference structure (CuTPP) in red dotted lines. S86

Figure S110: NSD result generated from **1:8** (in Å) (**A**) in-plane and (**B**) out-of-plane skeletal plots of the porphyrin core. Porphyrin is represented in black (C) and blue (N), with the reference structure (CuTPP) in red dotted lines. S87

Figure S111: NSD result generated from **1:9** (in Å) (**A**) in-plane and (**B**) out-of-plane skeletal plots of the porphyrin core. Porphyrin is represented in black (C) and blue (N), with the reference structure (CuTPP) in red dotted lines. S88

Figure S112: NSD result generated from **1:10** (in Å) (**A**) in-plane and (**B**) out-of-plane skeletal plots of the porphyrin core. Porphyrin is represented in black (C) and blue (N), with the reference structure (CuTPP) in red dotted lines. S89

Figure S113: NSD result generated from **1D** (in Å) (**A**) in-plane and (**B**) out-of-plane skeletal plots of the porphyrin core. Porphyrin is represented in black (C) and blue (N), with the reference structure (CuTPP) in red dotted lines. S90

Figure S114: NSD result generated from **2D** (in Å) (**A**) in-plane and (**B**) out-of-plane skeletal plots of the porphyrin core. Porphyrin is represented in black (C) and blue (N), with the reference structure (CuTPP) in red dotted lines. S91

Figure S115: NSD result generated from **1:11** (in Å) (**A**) in-plane and (**B**) out-of-plane skeletal plots of the porphyrin core. Porphyrin is represented in black (C) and blue (N), with the reference structure (CuTPP) in red dotted lines. S92

Figure S116: NSD result generated from **1:12** (in Å) (**A**) in-plane and (**B**) out-of-plane skeletal plots of the porphyrin core. Porphyrin is represented in black (C) and blue (N), with the reference structure (CuTPP) in red dotted lines. S93

Figure S117: NSD result generated from **3D** (in Å) (**A**) in-plane and (**B**) out-of-plane skeletal plots of the porphyrin core. Porphyrin is represented in black (C) and blue (N), with the reference structure (CuTPP) in red dotted lines. S94

Figure S118: NSD result generated from **1:13** (in Å) (**A**) in-plane and (**B**) out-of-plane skeletal plots of the porphyrin core. Porphyrin is represented in black (C) and blue (N), with the reference structure (CuTPP) in red dotted lines. S95

Figure S119: NSD result generated from **1:14** (in Å) (**A**) in-plane and (**B**) out-of-plane skeletal plots of the porphyrin core. Porphyrin is represented in black (C) and blue (N), with the reference structure (CuTPP) in red dotted lines. S96

Figure S120: NSD result generated from **1:15** (in Å) (**A**) in-plane and (**B**) out-of-plane skeletal plots of the porphyrin core. Porphyrin is represented in black (C) and blue (N), with the reference structure (CuTPP) in red dotted lines. S97

NSD tables and plots for DFT structures series 2 .... S98

Figure S121: NSD result generated from **2:1** (in Å) (**A**) in-plane and (**B**) out-of-plane skeletal plots of the porphyrin core. Porphyrin is represented in black (C) and blue (N), with the reference structure (CuTPP) in red dotted lines. S98

Figure S122: NSD result generated from **2:2** (in Å) (**A**) in-plane and (**B**) out-of-plane skeletal plots of the porphyrin core. Porphyrin is represented in black (C) and blue (N), with the reference structure (CuTPP) in red dotted lines. S99

Figure S123: NSD result generated from **2:3** (in Å) (**A**) in-plane and (**B**) out-of-plane skeletal plots of the porphyrin core. Porphyrin is represented in black (C) and blue (N), with the reference structure (CuTPP) in red dotted lines. S100

Figure S124: NSD result generated from **2:4** (in Å) (**A**) in-plane and (**B**) out-of-plane skeletal plots of the porphyrin core. Porphyrin is represented in black (C) and blue (N), with the reference structure (CuTPP) in red dotted lines. S101

Figure S125: NSD result generated from **2:5** (in Å) (**A**) in-plane and (**B**) out-of-plane skeletal plots of the porphyrin core. Porphyrin is represented in black (C) and blue (N), with the reference structure (CuTPP) in red dotted lines. S102

Figure S126: NSD result generated from **2:6** (in Å) (**A**) in-plane and (**B**) out-of-plane skeletal plots of the porphyrin core. Porphyrin is represented in black (C) and blue (N), with the reference structure (CuTPP) in red dotted lines. S103

NSD tables and plots for DFT structures series 3 .... S104

Figure S127: NSD result generated from **3:1** (in Å) (**A**) in-plane and (**B**) out-of-plane skeletal plots of the porphyrin core. Porphyrin is represented in black (C) and blue (N), with the reference structure (CuTPP) in red dotted lines. S104

Figure S128: NSD result generated from **3:2** (in Å) (**A**) in-plane and (**B**) out-of-plane skeletal plots of the porphyrin core. Porphyrin is represented in black (C) and blue (N), with the reference structure (CuTPP) in red dotted lines. S105

Figure S129: NSD result generated from **3:3** (in Å) (**A**) in-plane and (**B**) out-of-plane skeletal plots of the porphyrin core. Porphyrin is represented in black (C) and blue (N), with the reference structure (CuTPP) in red dotted lines. S106

NMR and UV spectrum. S107

Figure S130: ^{1}H NMR spectrum of compound **4** in $CDCl_3$. S107

Figure S131: ^{13}C NMR spectrum of compound **4** in $CDCl_3$. S108

Figure S132: UV-visible spectrum of compound **4** in dichloromethane. S108

Figures from X-ray crystallography and DFT.

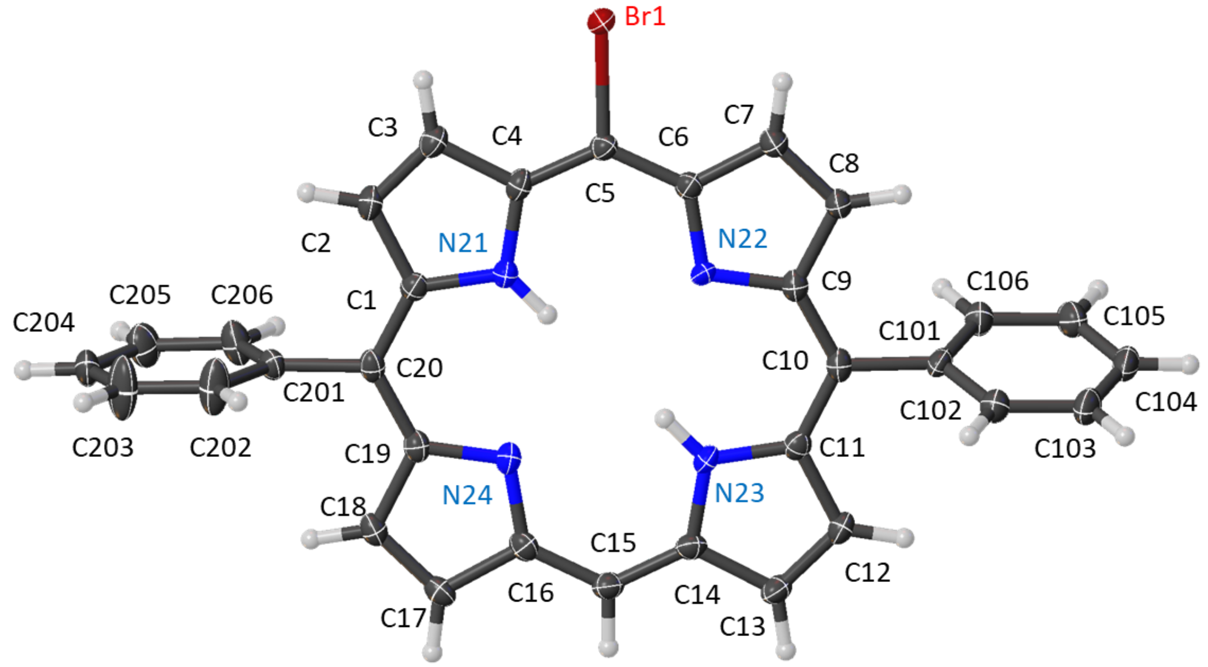

Figure S1: Thermal ellipsoid plot of compound **1** (thermal displacement given as 50% probability). Minor disordered moieties have been omitted.

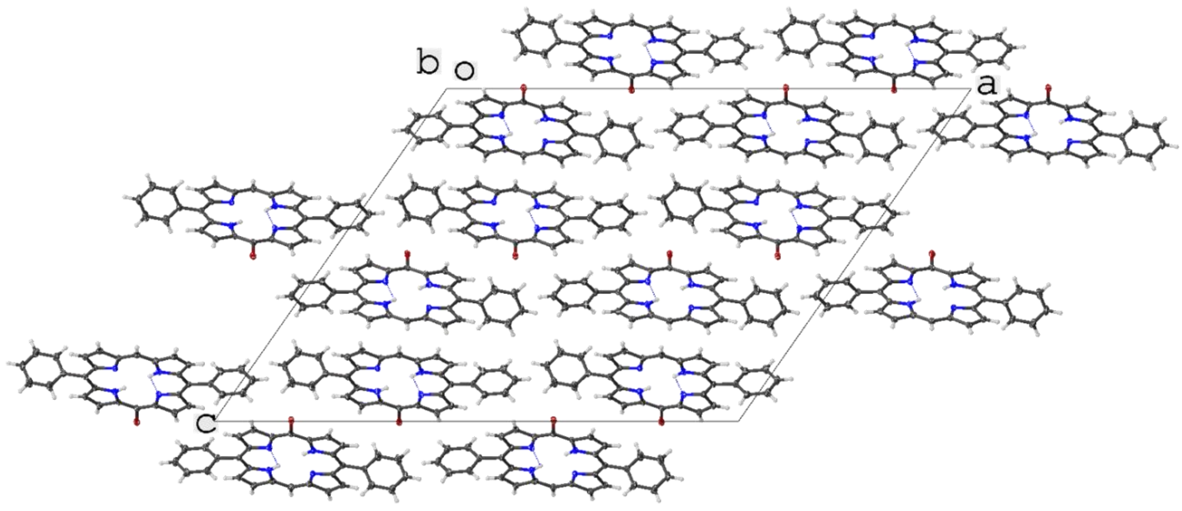

Figure S2: Packing diagram (thermal ellipsoid plot) of compound **1** looking down the *b*-axis (thermal displacement given as 50% probability). Minor disordered moieties have been omitted.

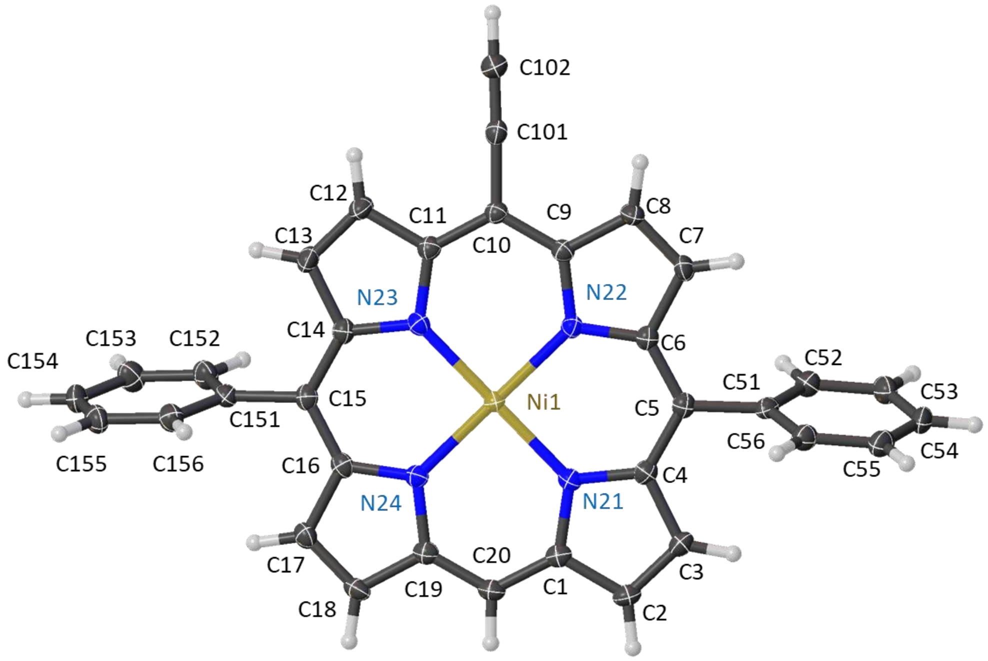

Figure S3: Thermal ellipsoid plot of compound **2A** (thermal displacement given as 50% probability). Minor disordered moieties have been omitted.

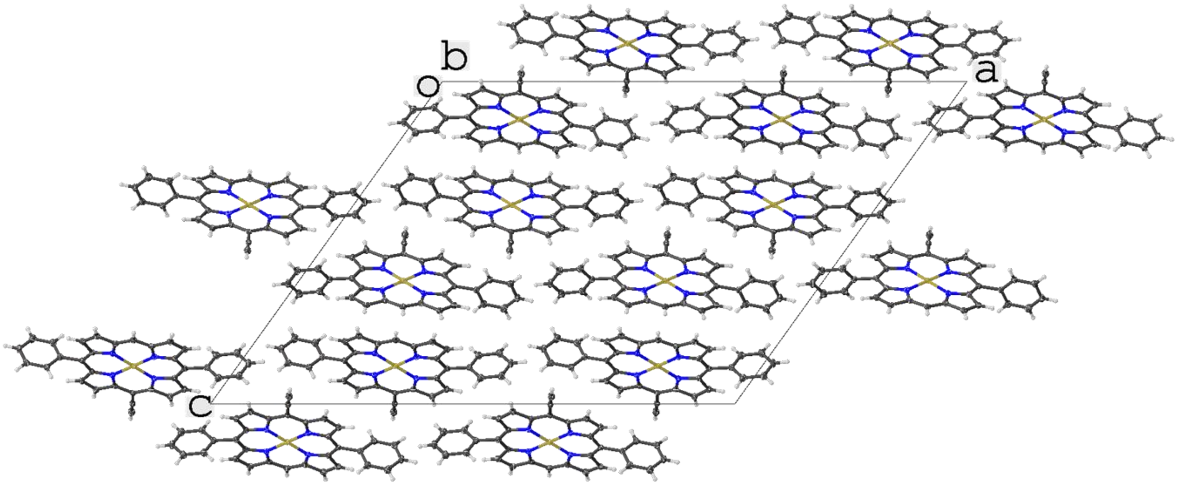

Figure S4: Packing diagram (thermal ellipsoid plot) of compound **2A** looking down the *b*-axis (thermal displacement given as 50% probability). Minor disordered moieties have been omitted.

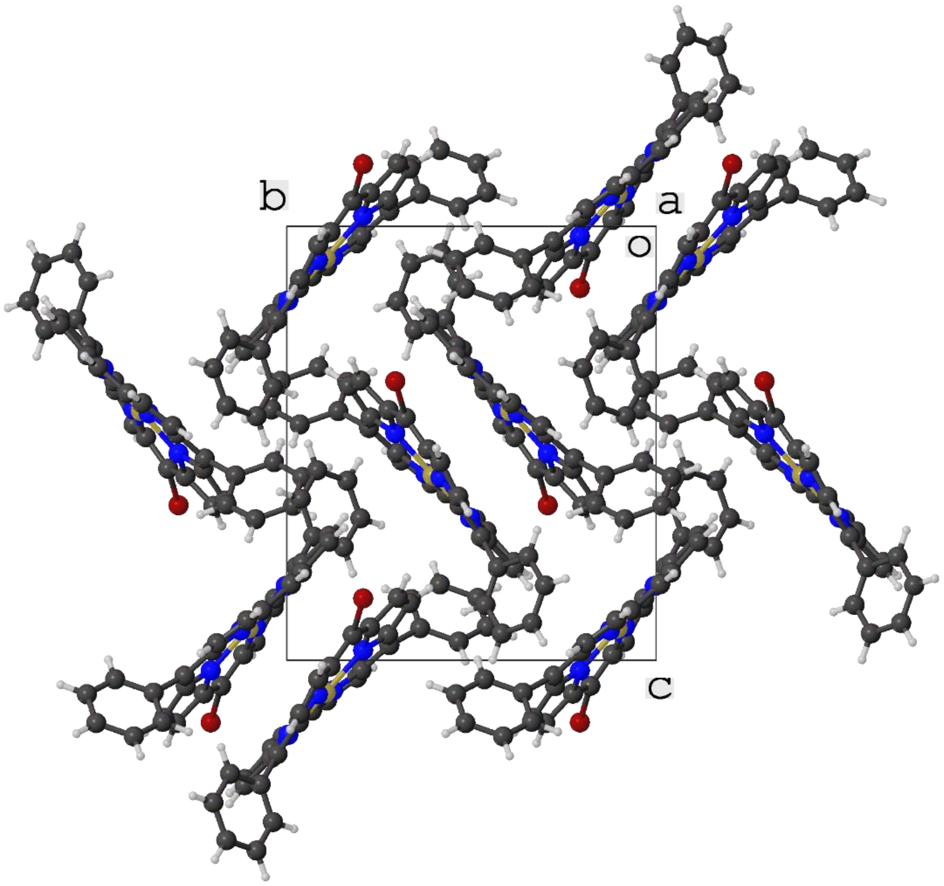

Figure S5: Packing diagram (ball and stick) of compound **2** looking down the *a*-axis.

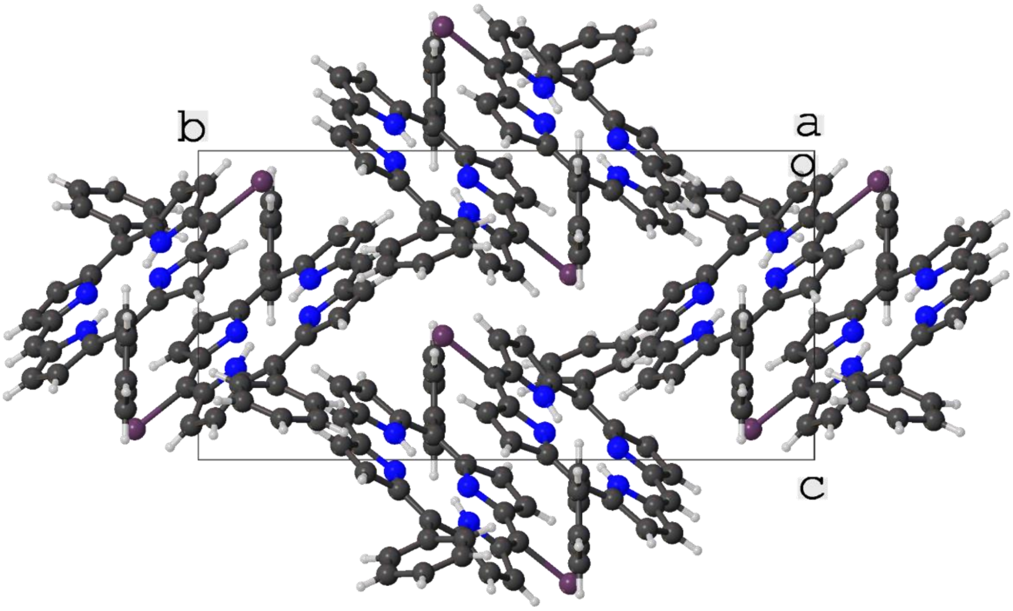

Figure S6: Packing diagram (ball and stick) of compound **3** looking down the *a*-axis.

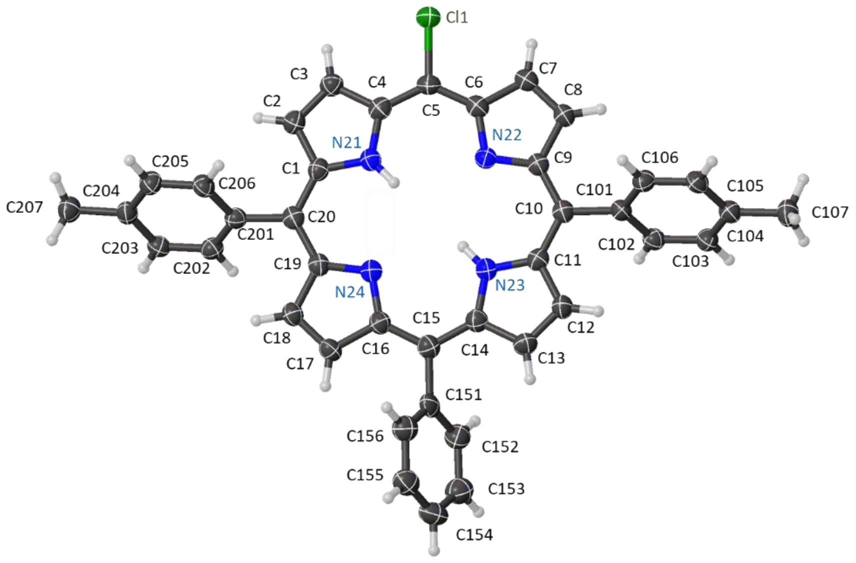

Figure S7: Thermal ellipsoid plot of compound **4** (thermal displacement given as 50% probability). Minor disordered moieties have been omitted.

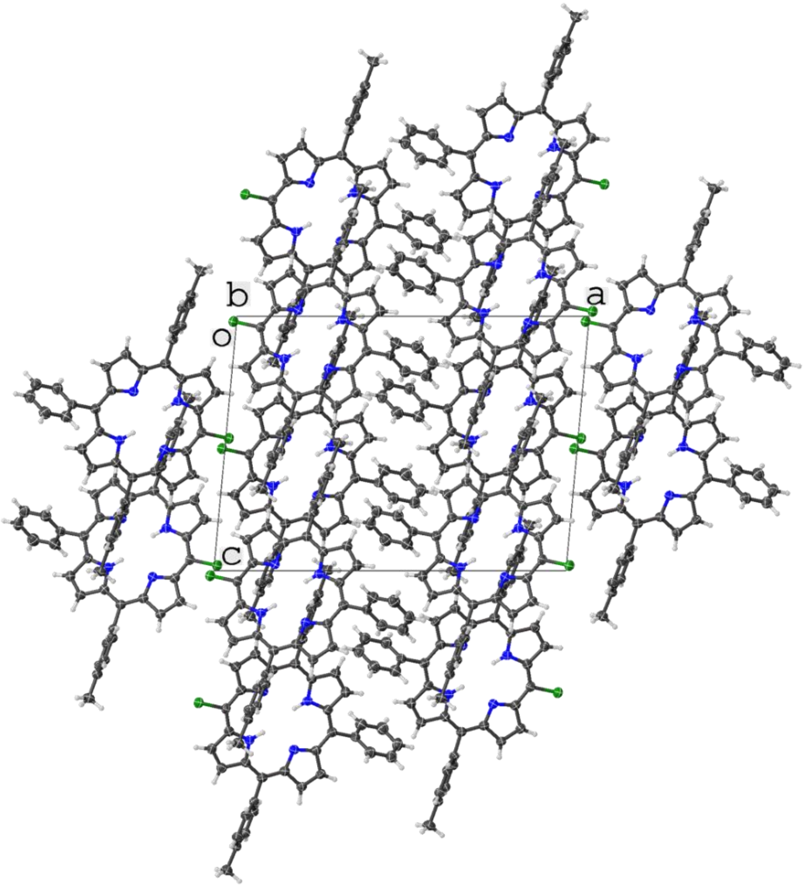

Figure S8: Packing diagram (thermal ellipsoid plot) of compound **4** looking down the *b*-axis (thermal displacement given as 50% probability). Minor disordered moieties have been omitted.

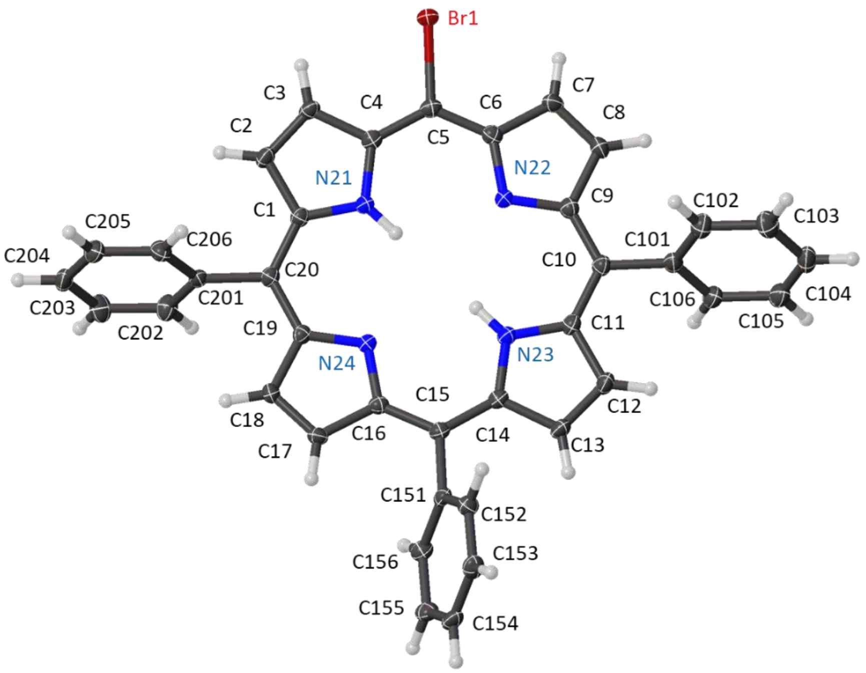

Figure S9: Thermal ellipsoid plot of compound **5** (thermal displacement given as 50% probability). Minor disordered moieties have been omitted.

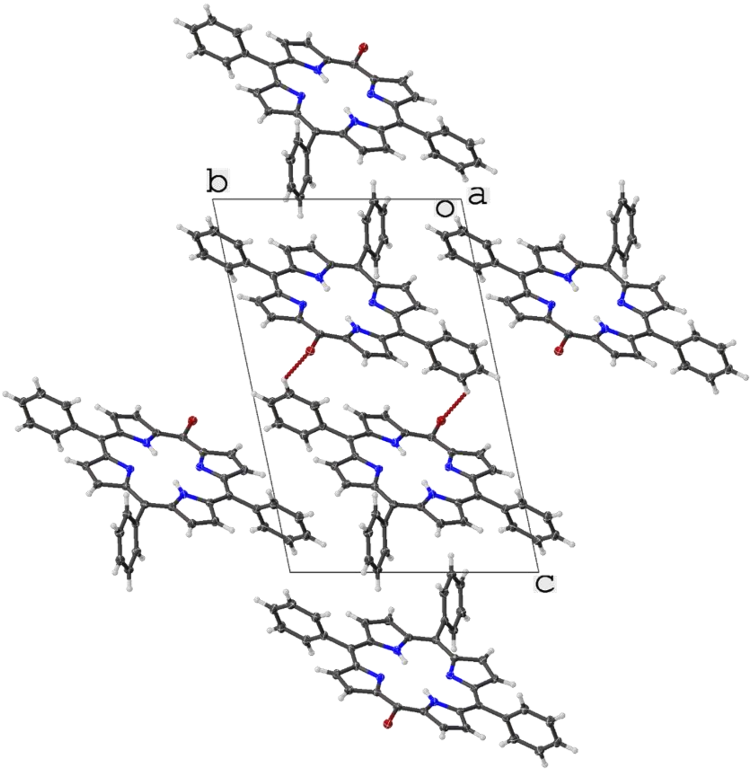

Figure S10: Packing diagram (thermal ellipsoid plot) of compound **5** looking down the *a*-axis (thermal displacement given as 50% probability). Minor disordered moieties have been omitted.

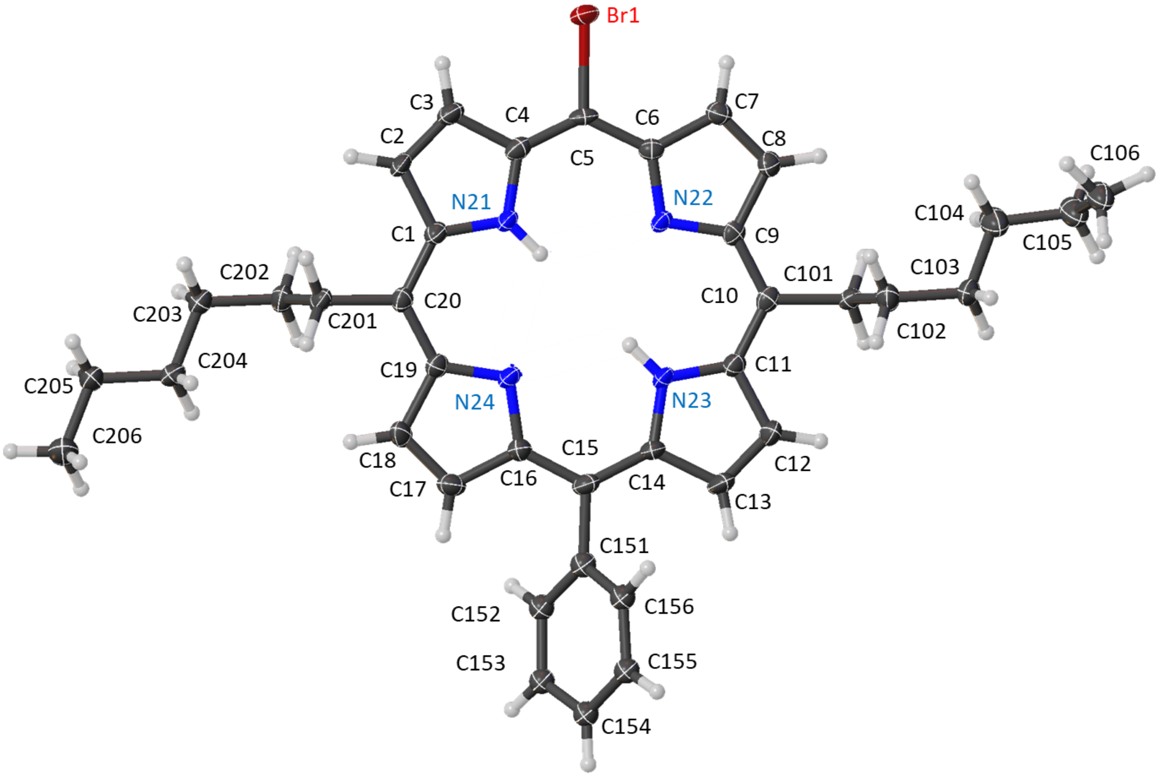

Figure S11: Thermal ellipsoid plot of compound **6** (thermal displacement given as 50% probability). Minor disordered moieties have been omitted.

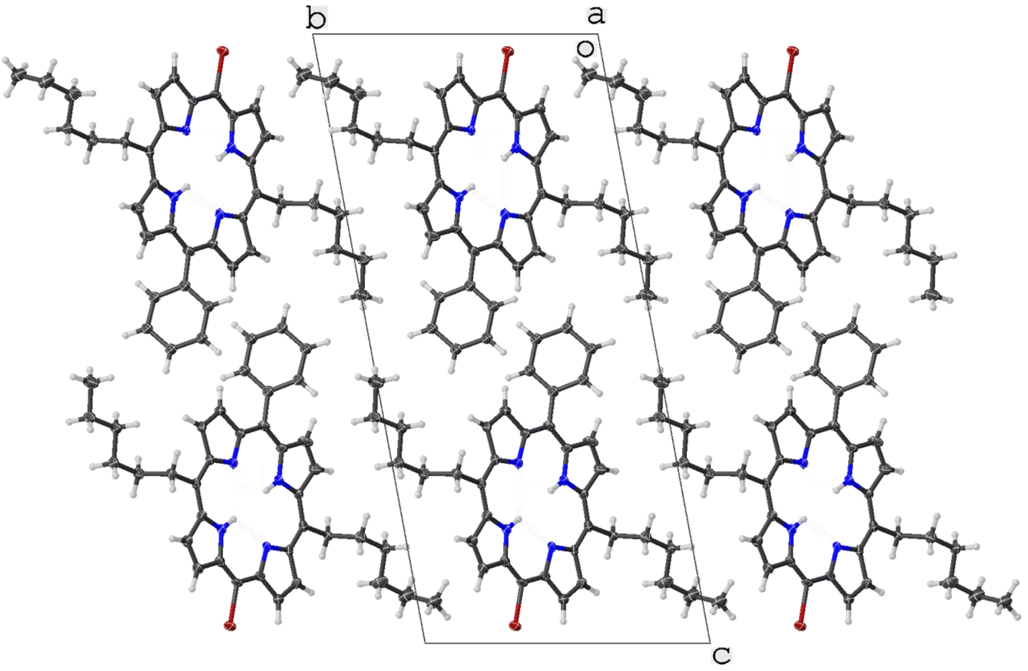

Figure S12: Packing diagram (thermal ellipsoid plot) of compound **6** looking down the *a*-axis (thermal displacement given as 50% probability). Minor disordered moieties have been omitted.

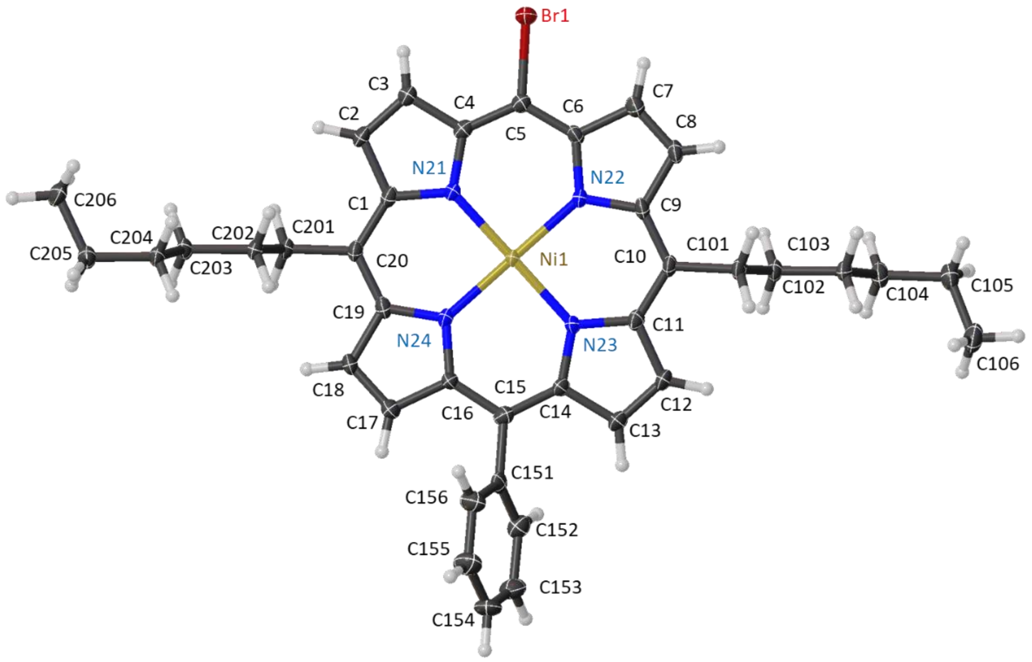

Figure S13: Thermal ellipsoid plot of compound **7** (thermal displacement given as 50% probability). Minor disordered moieties have been omitted.

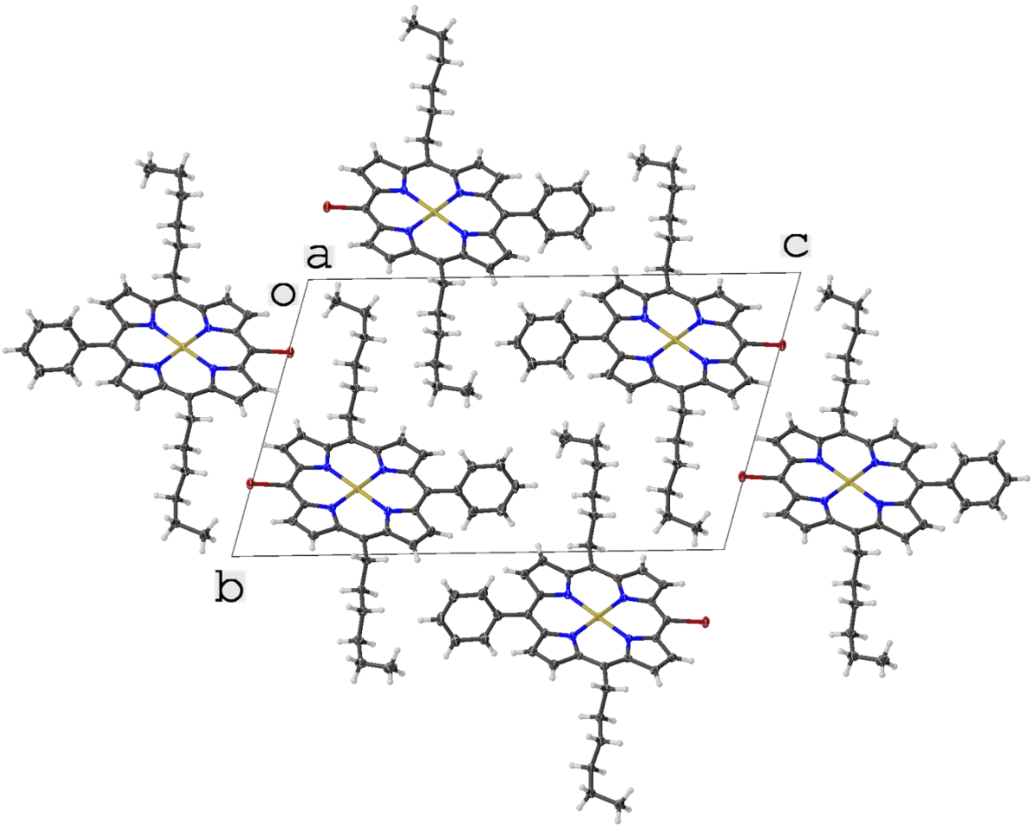

Figure S14: Packing diagram (thermal ellipsoid plot) of compound **7** looking down the *a*-axis (thermal displacement given as 50% probability). Minor disordered moieties have been omitted.

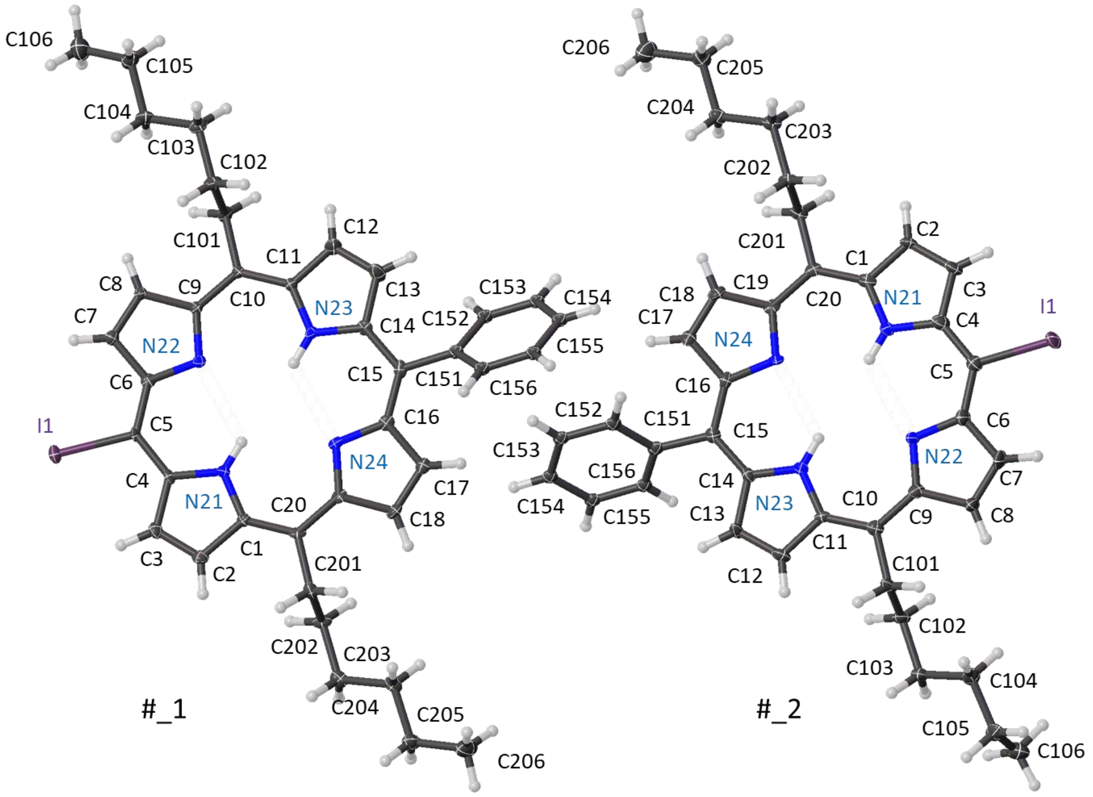

Figure S15: Thermal ellipsoid plot of compound **8** (thermal displacement given as 50% probability). Minor disordered moieties have been omitted. #_1 and #_2 indicate the residue of each molecule in the asymmetric unit.

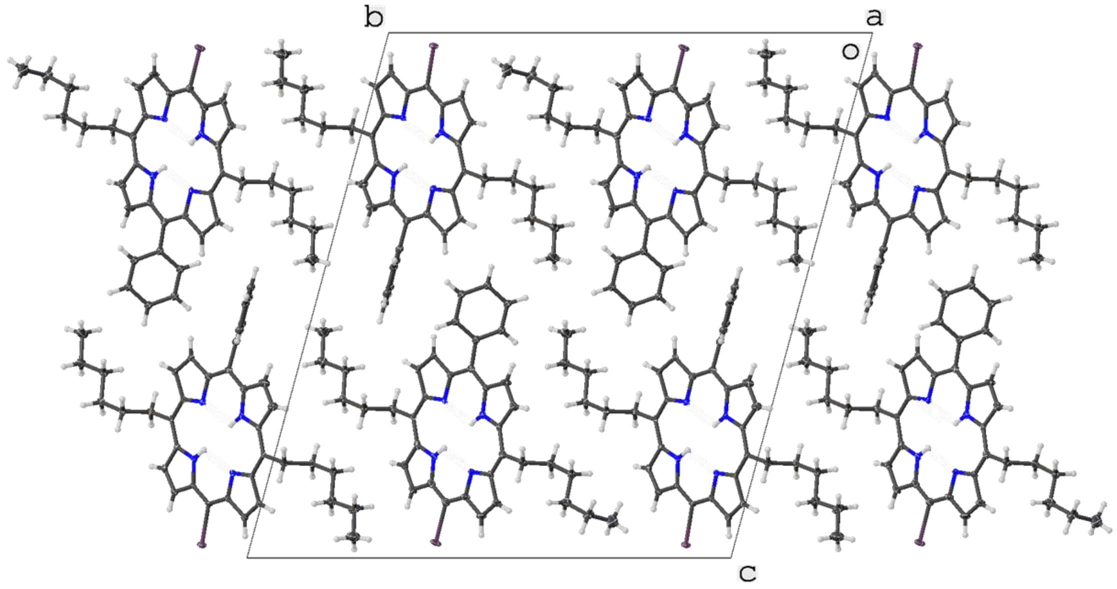

Figure S16: Packing diagram (thermal ellipsoid plot) of compound **8** looking down the *a*-axis (thermal displacement given as 50% probability). Minor disordered moieties have been omitted.

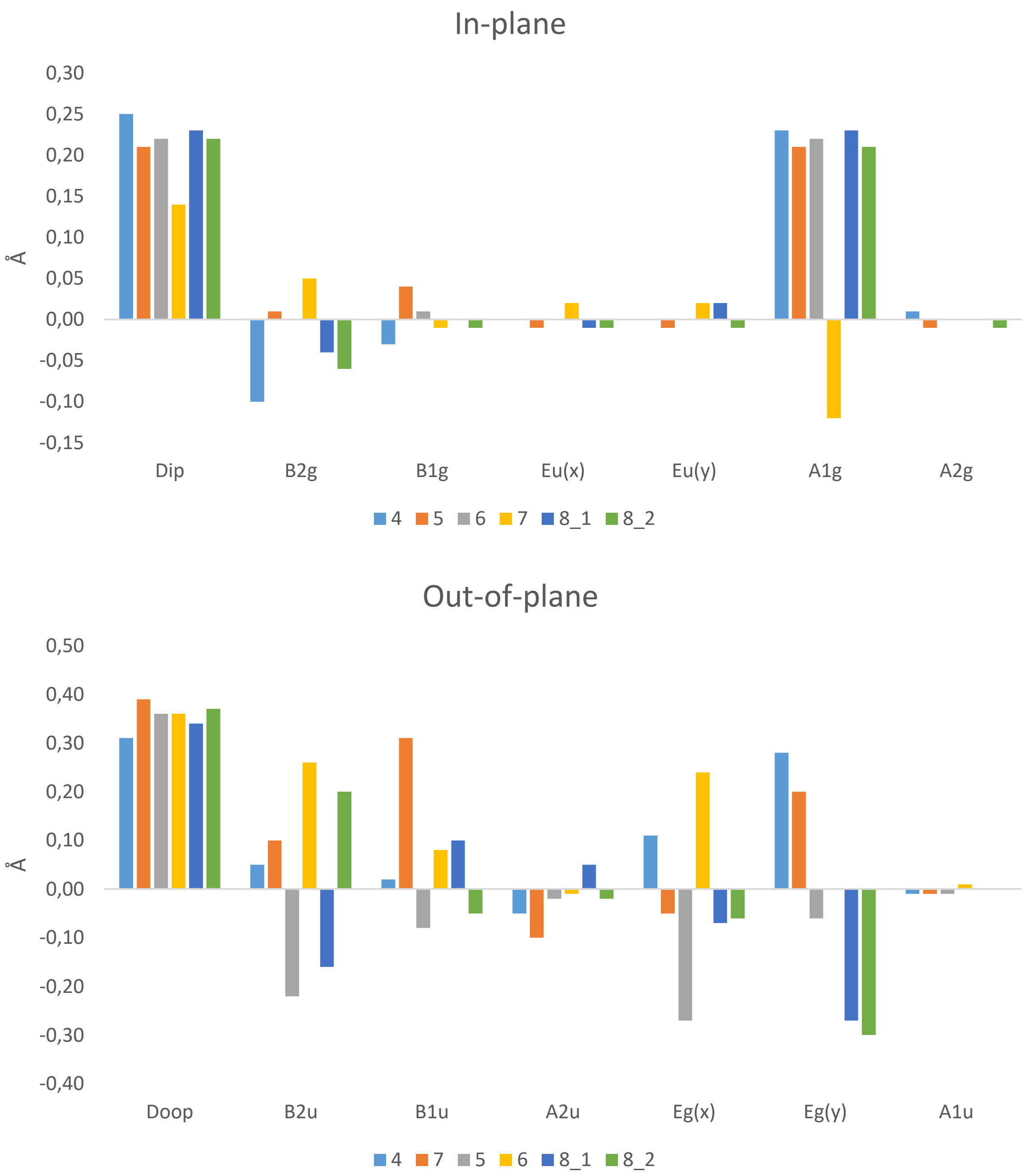

Figure S17: NSD charts for compounds **4-8**.

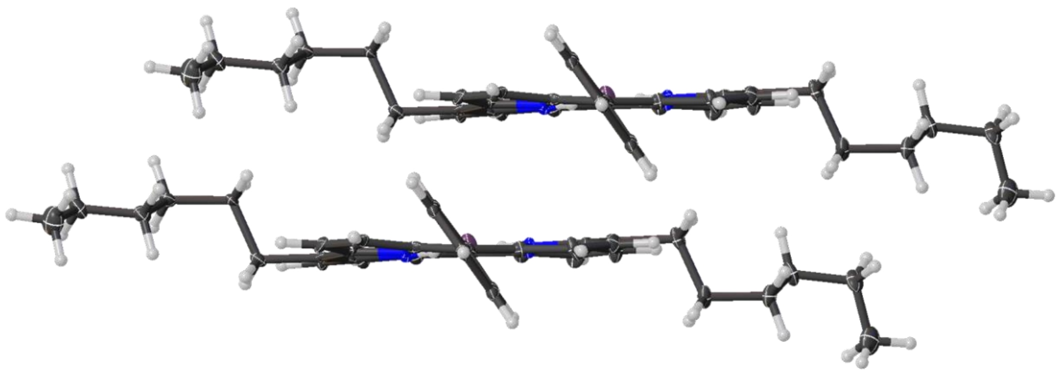

Figure S18: Expanded view (thermal ellipsoid plot) of compound **8** showing the stacking between porphyrin macrocycles.

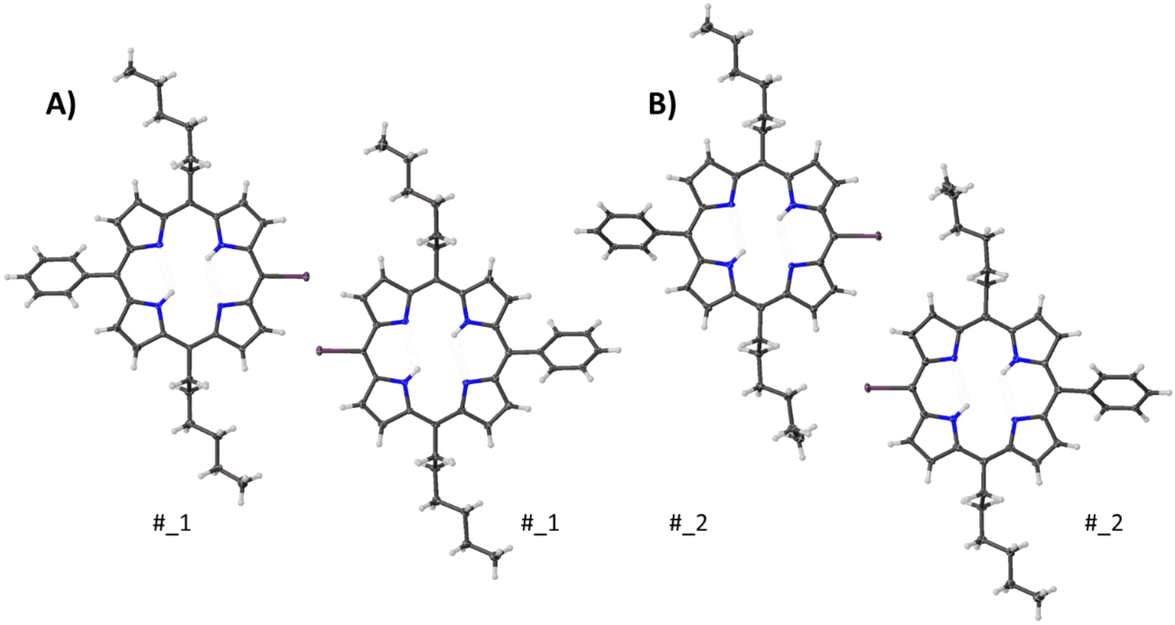

Figure S19: Expanded view (thermal ellipsoid plot) of compound **8** showing the close packing between (**A**) pyrrole and iodine (**B**) hexyl and iodine. #_X indicated the residue number.

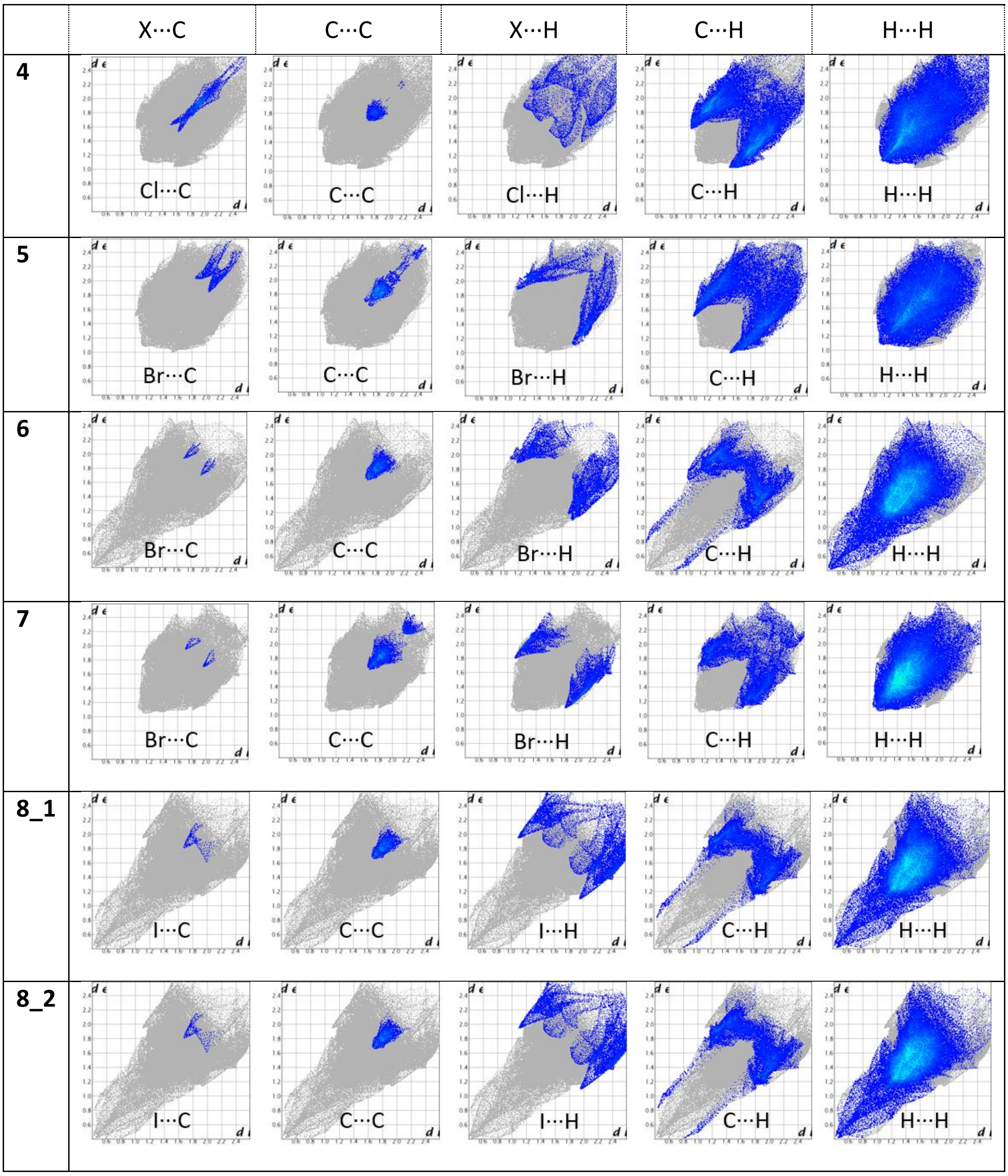

Figure S20: Hirshfeld surfaces of compounds **4**-**8**.

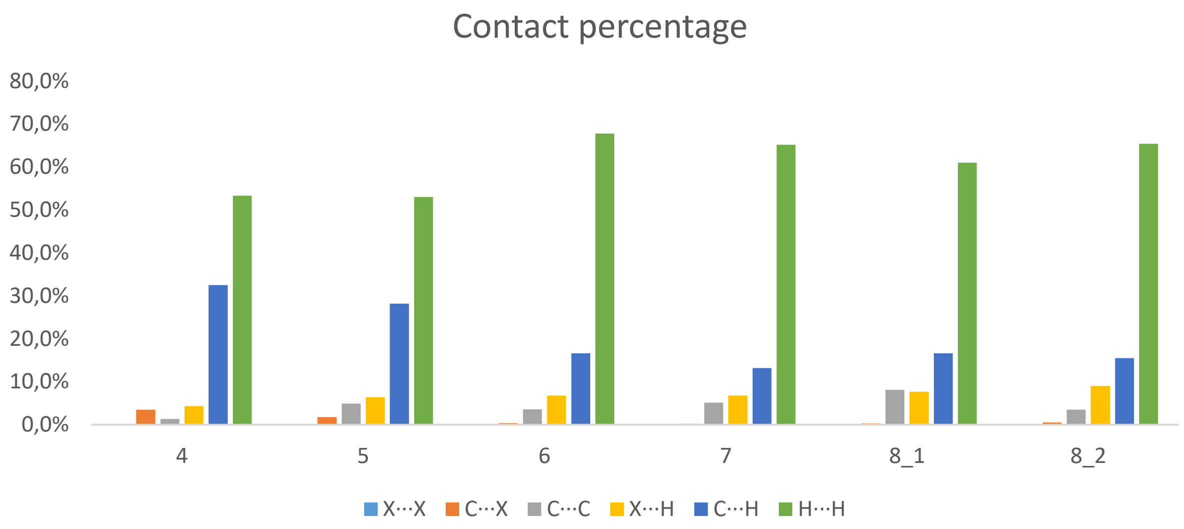

Figure S21: Contact percentages of compounds **4**-**8**.

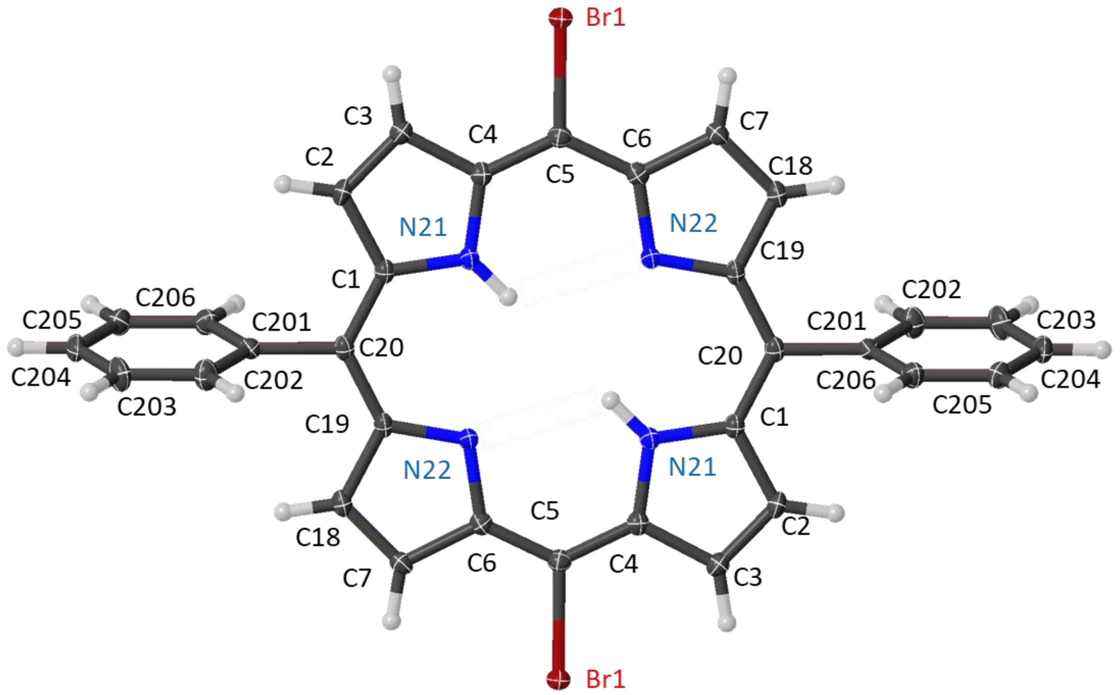

Figure S22: Thermal ellipsoid plot of compound **9** (thermal displacement given as 50% probability). Minor disordered moieties have been omitted.

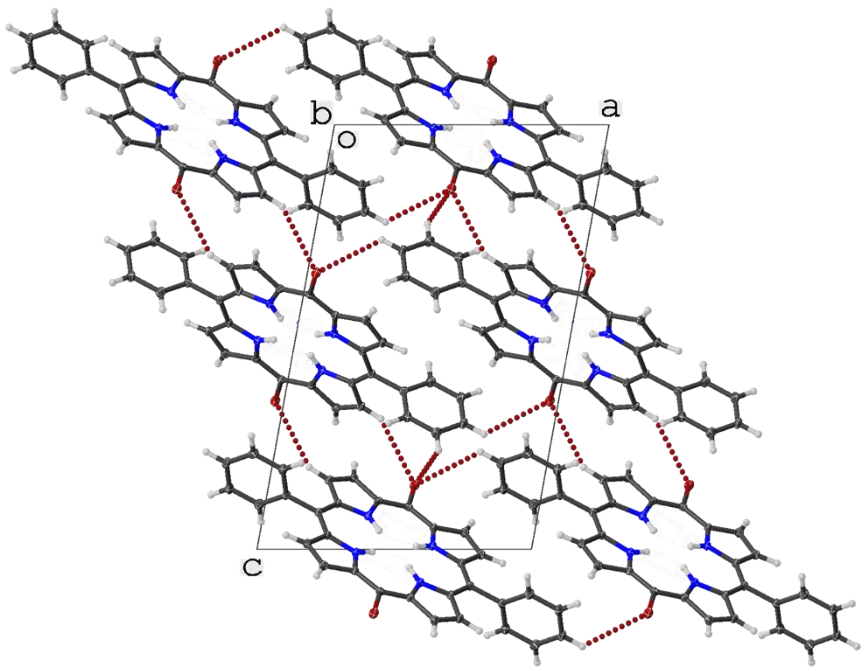

Figure S23: Packing diagram (thermal ellipsoid plot) of compound **9** looking down the *b*-axis (thermal displacement given as 50% probability). Minor disordered moieties have been omitted.

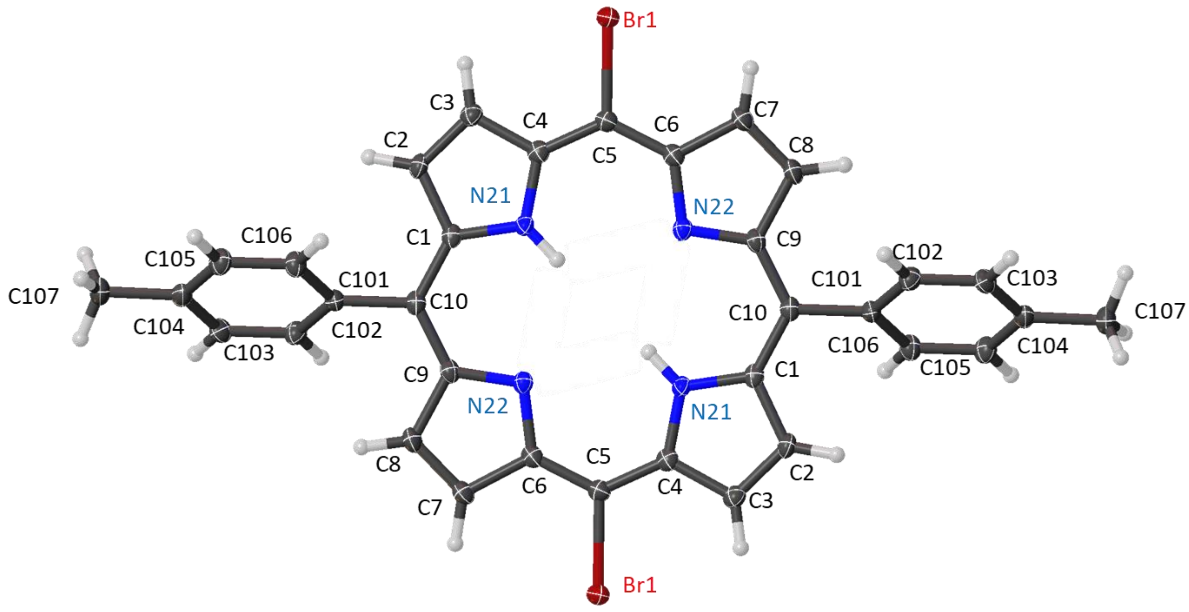

Figure S24: Thermal ellipsoid plot of compound **10** (thermal displacement given as 50% probability). Minor disordered moieties have been omitted.

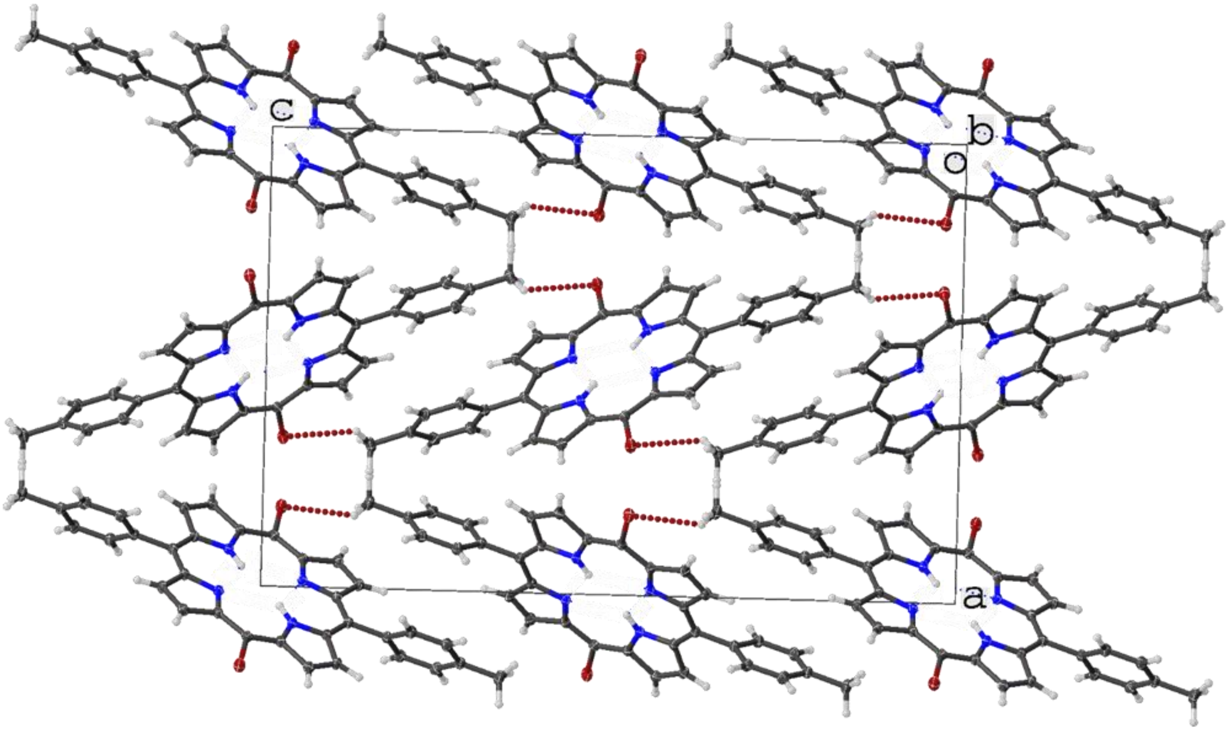

Figure S25: Packing diagram (thermal ellipsoid plot) of compound **10** looking down the *b*-axis (thermal displacement given as 50% probability). Minor disordered moieties have been omitted.

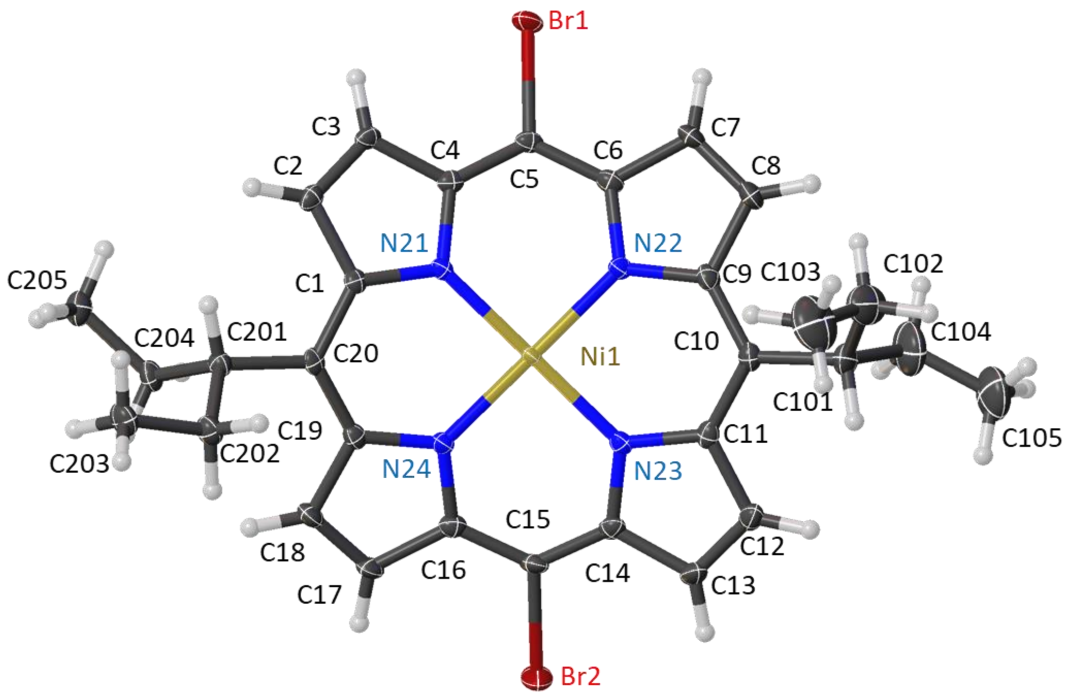

Figure S26: Thermal ellipsoid plot of compound **11** (thermal displacement given as 50% probability). Minor disordered moieties have been omitted.

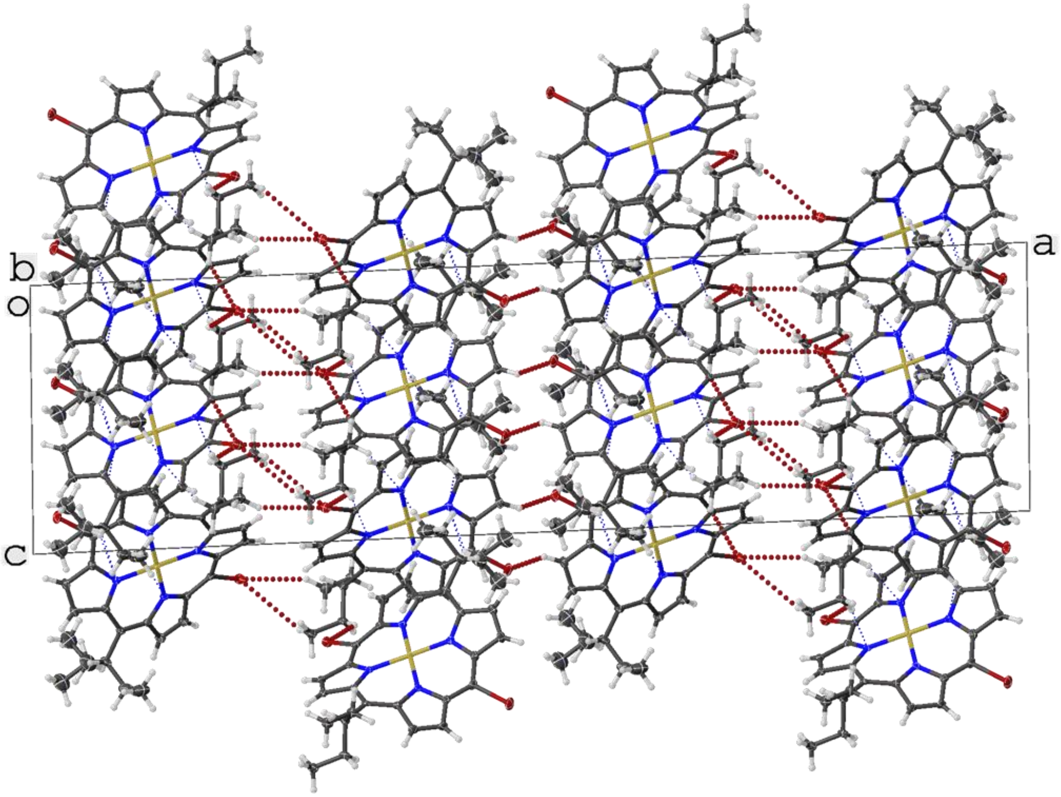

Figure S27: Packing diagram (thermal ellipsoid plot) of compound **11** looking down the *b*-axis (thermal displacement given as 50% probability). Minor disordered moieties have been omitted.

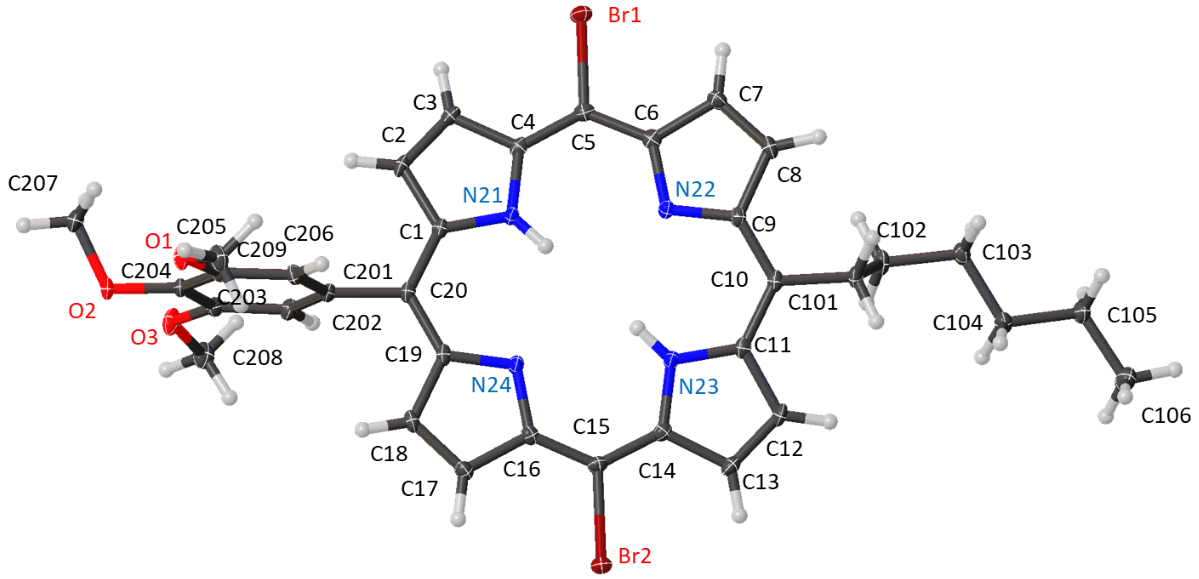

Figure S28: Thermal ellipsoid plot of compound **13** (thermal displacement given as 50% probability). Minor disordered moieties have been omitted.

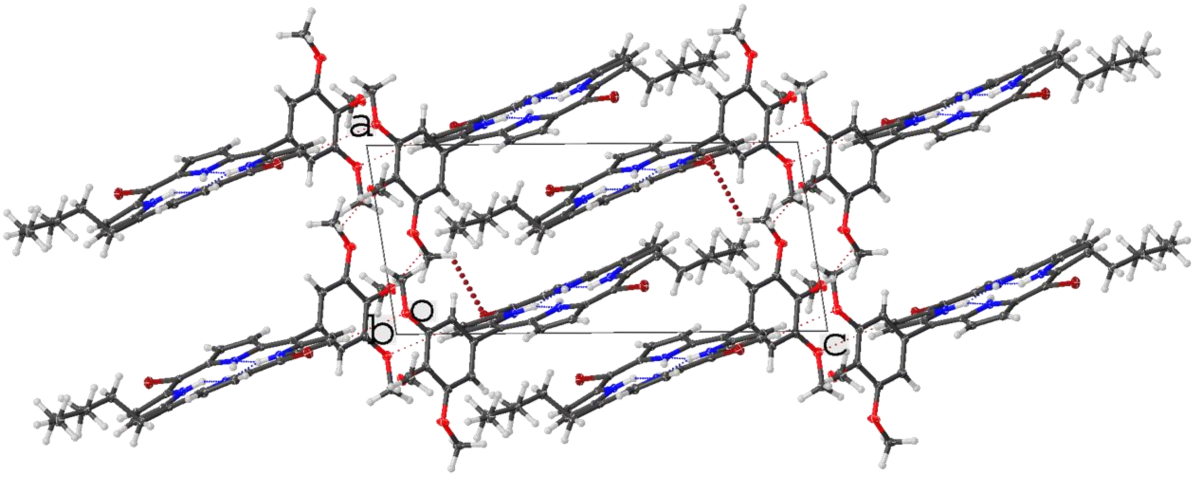

Figure S29: Packing diagram (thermal ellipsoid plot) of compound **13** looking down the *b*-axis (thermal displacement given as 50% probability). Minor disordered moieties have been omitted.

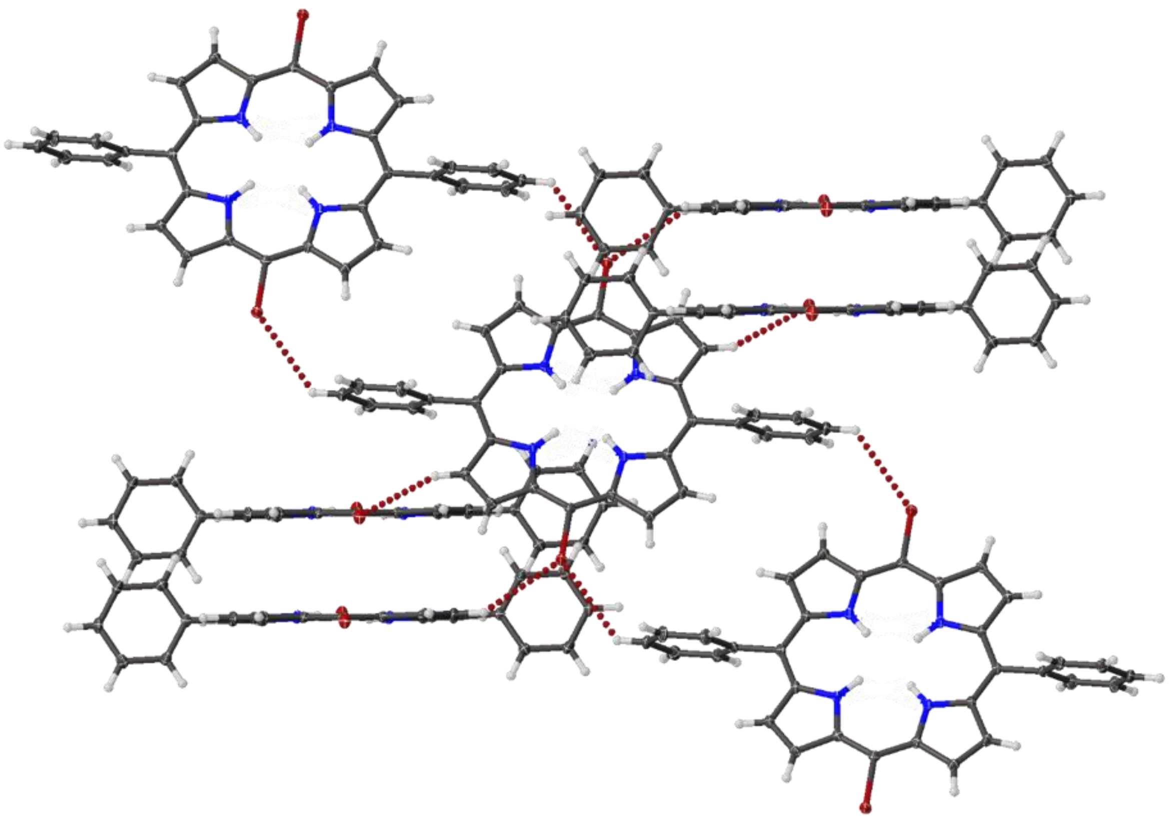

Figure S30: Expanded view (thermal ellipsoid plot) of compound **9** showing the combination of interactions seen in Figure S37.

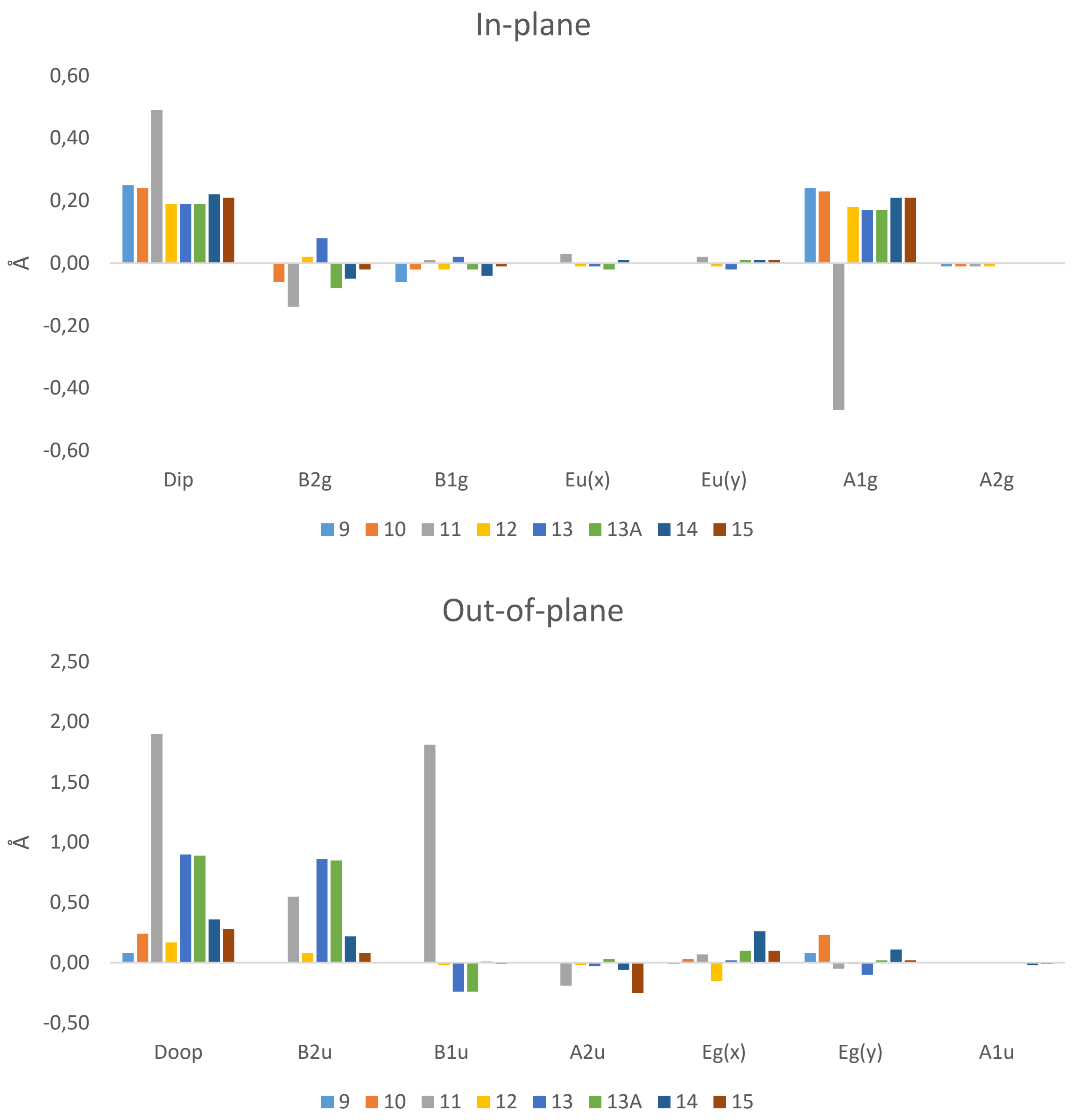

Figure S31: NSD charts for compounds **9**-**15**.

A)

B)

C)

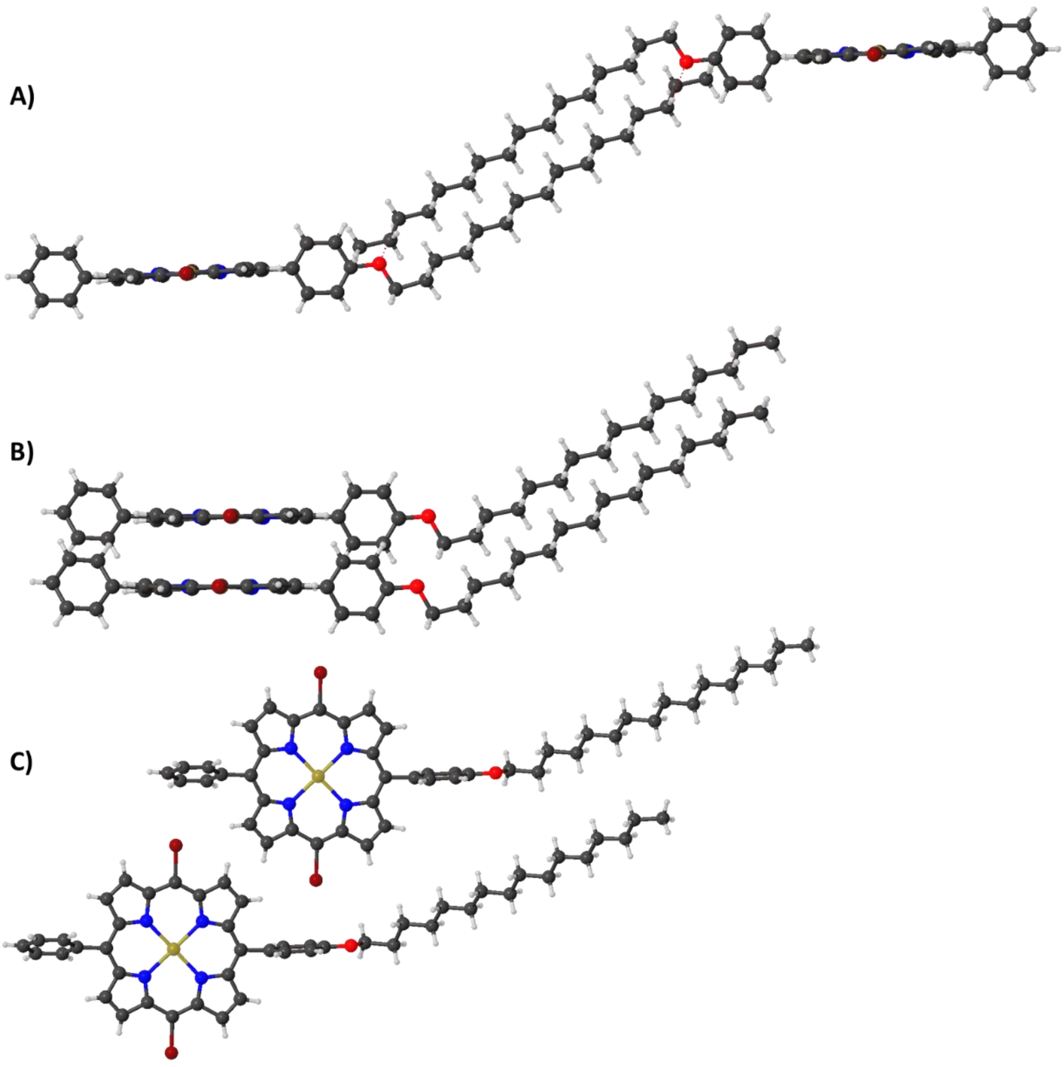

Figure S32: Expanded view (ball and stick) of compound 12 showing the (A) alkyl chain interactions (B) porphyrin stacking (C) close packing side-on alignment.

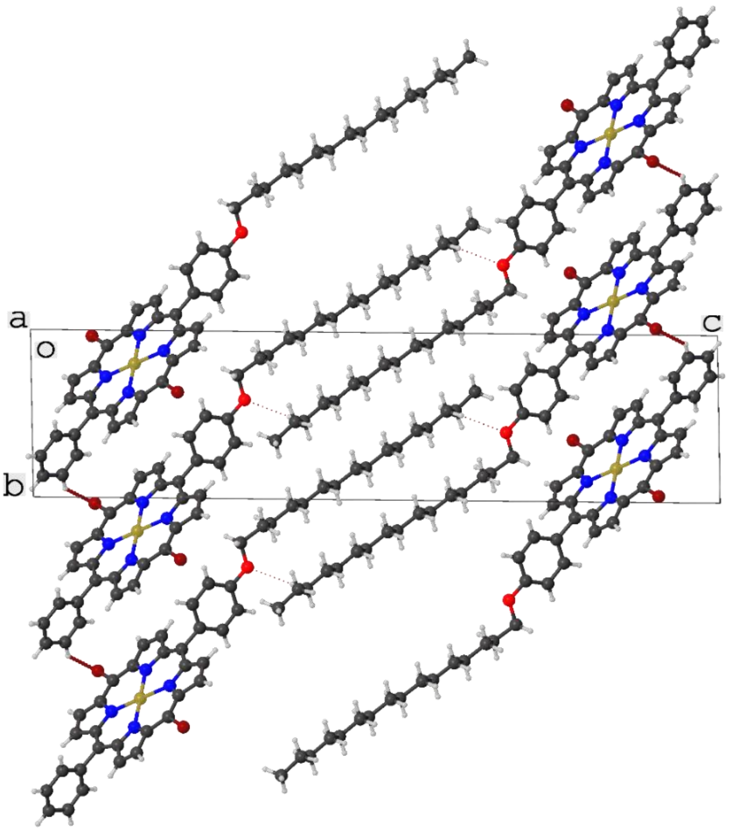

Figure S33: Packing diagram (ball and stick) of compound **12** looking down the *a*-axis.

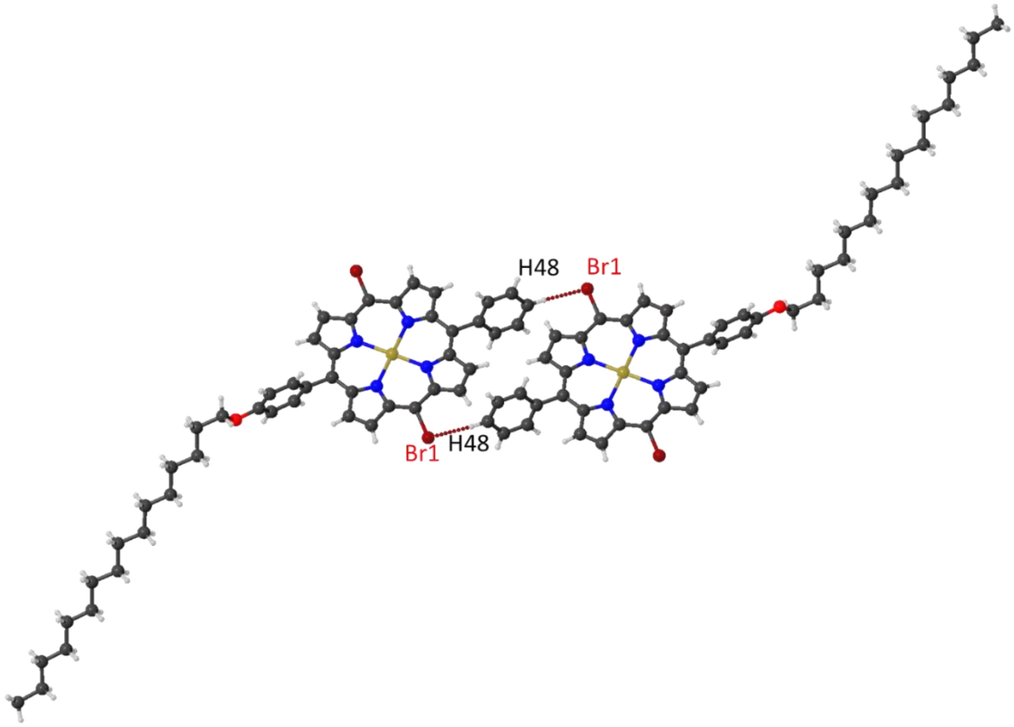

Figure S34: Expanded view (ball and stick) of compound **12** showing the head-to-head Br···H contact.

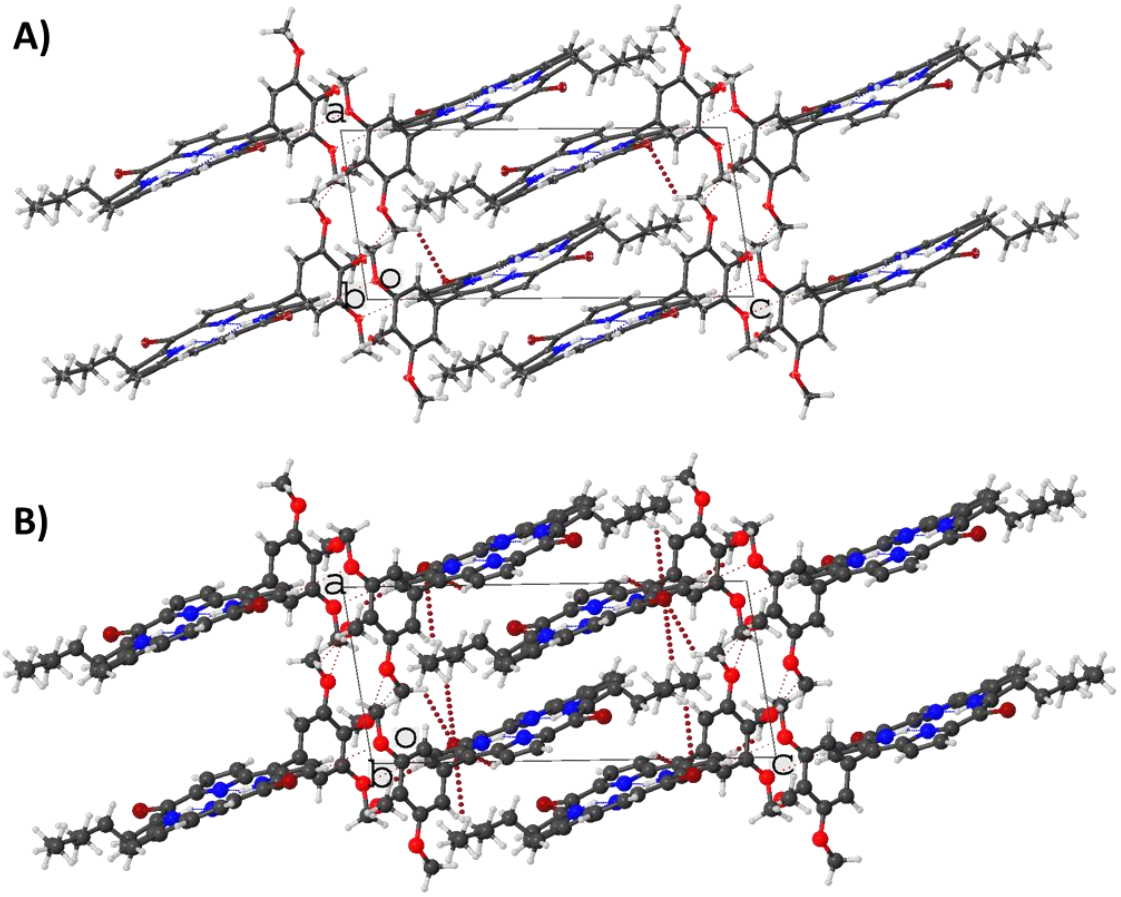

Figure S35: Packing diagrams for compound **13** (thermal ellipsoid plot) (**A**) and **13A** (ball and stick) (**B**) looking down the *b*-axis.

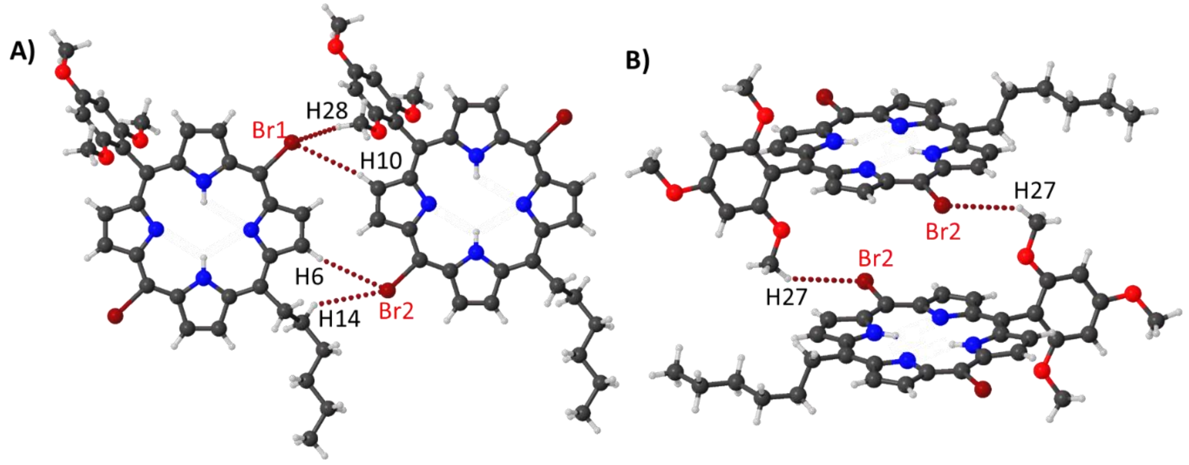

Figure S36: Expanded view (ball and stick) of compound **14** showing (**A**) Br···H interactions with pyrrole units, methoxy group, and the hexyl chains (**B**) Br···H interactions with methoxy group.

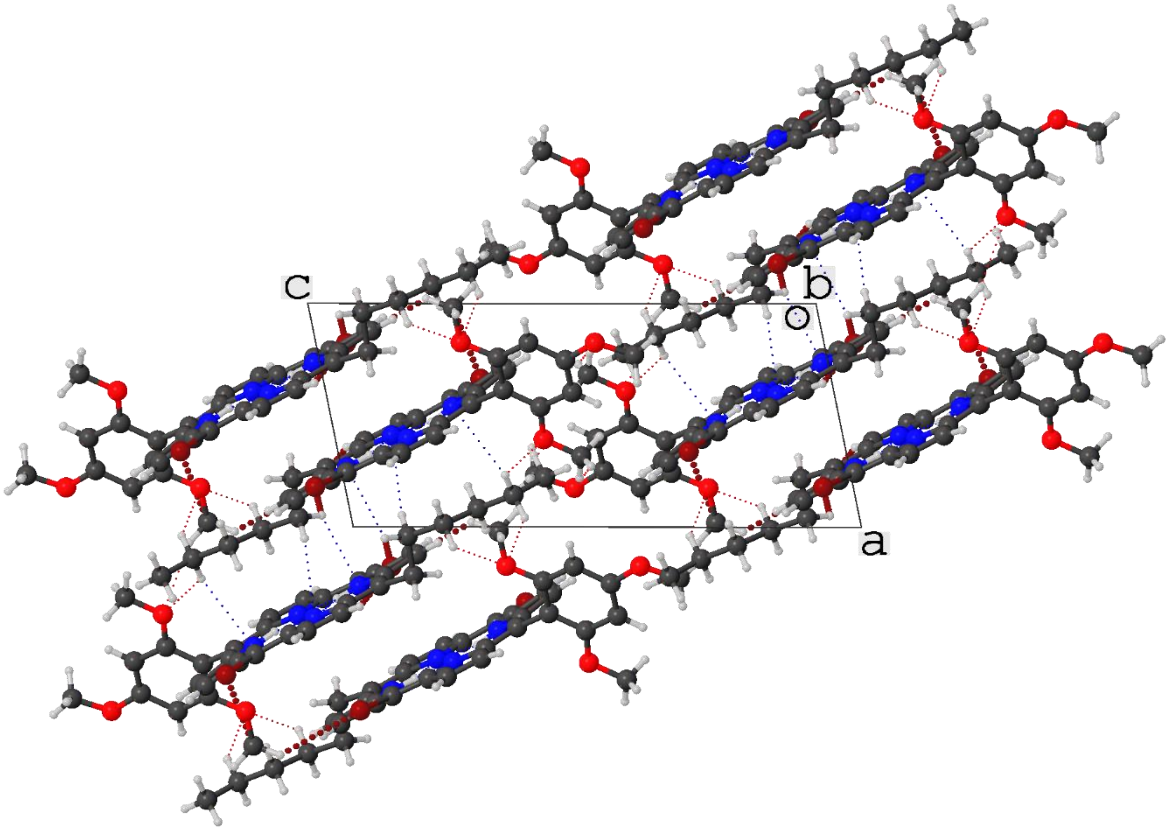

Figure S37: Packing diagrams (ball and stick) of compound **14** looking down the *b*-axis.

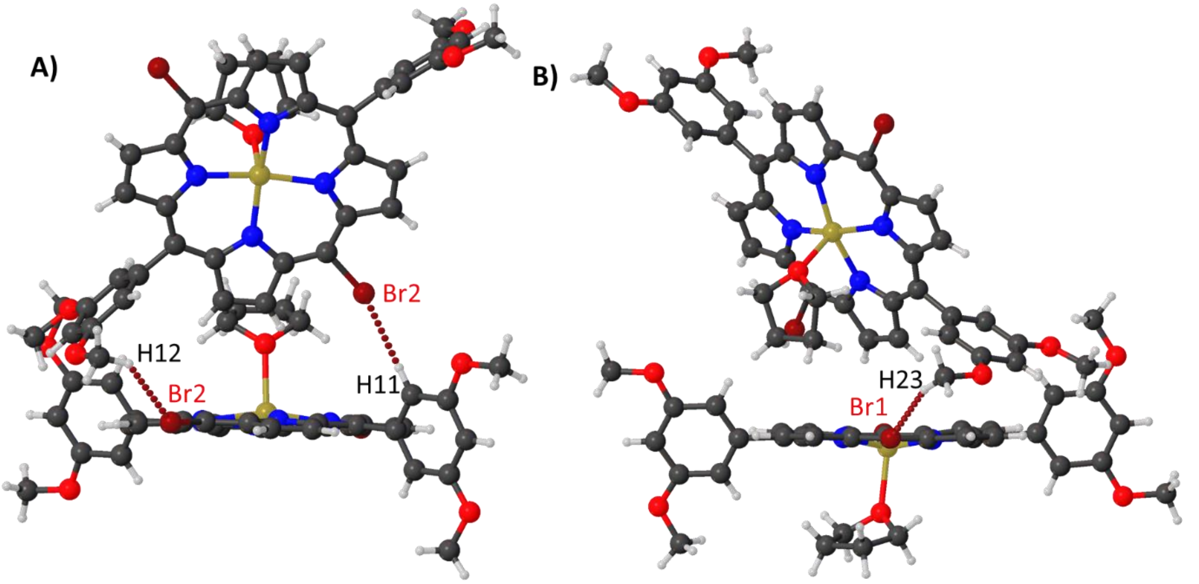

Figure S38: Expanded view (ball and stick) of compound **15** showing the above (**A**) and below (**B**) plane interactions between porphyrins.

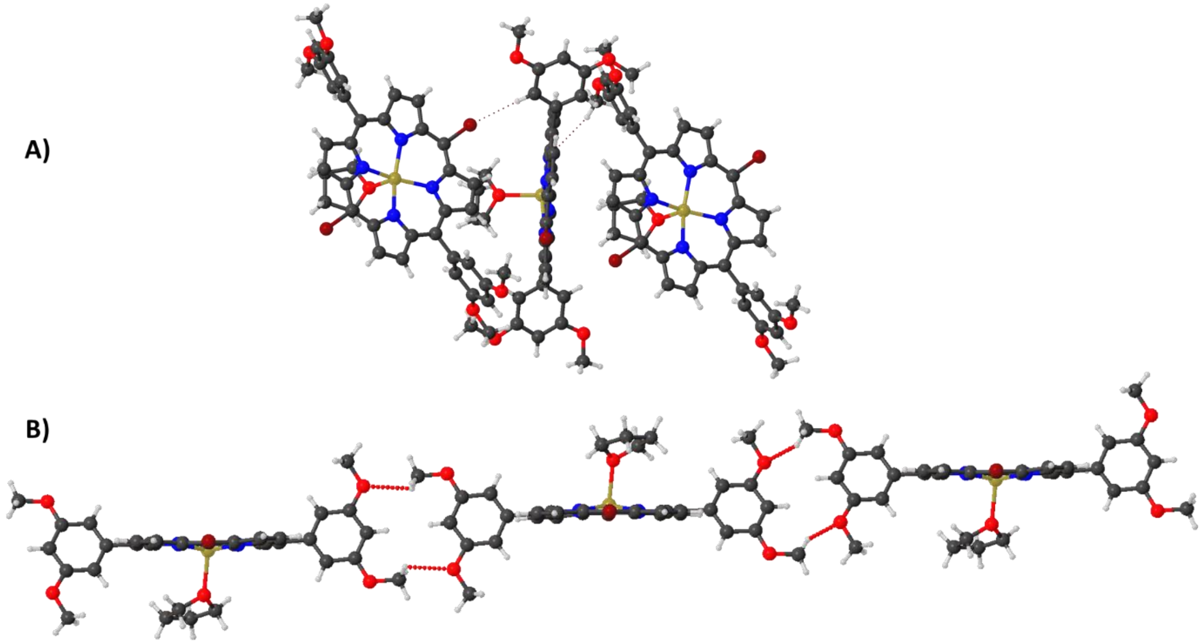

Figure S39: Expanded view (ball and stick) of compound **15** showing face-to-edge (**A**) and hydrogen bonded network (**B**).

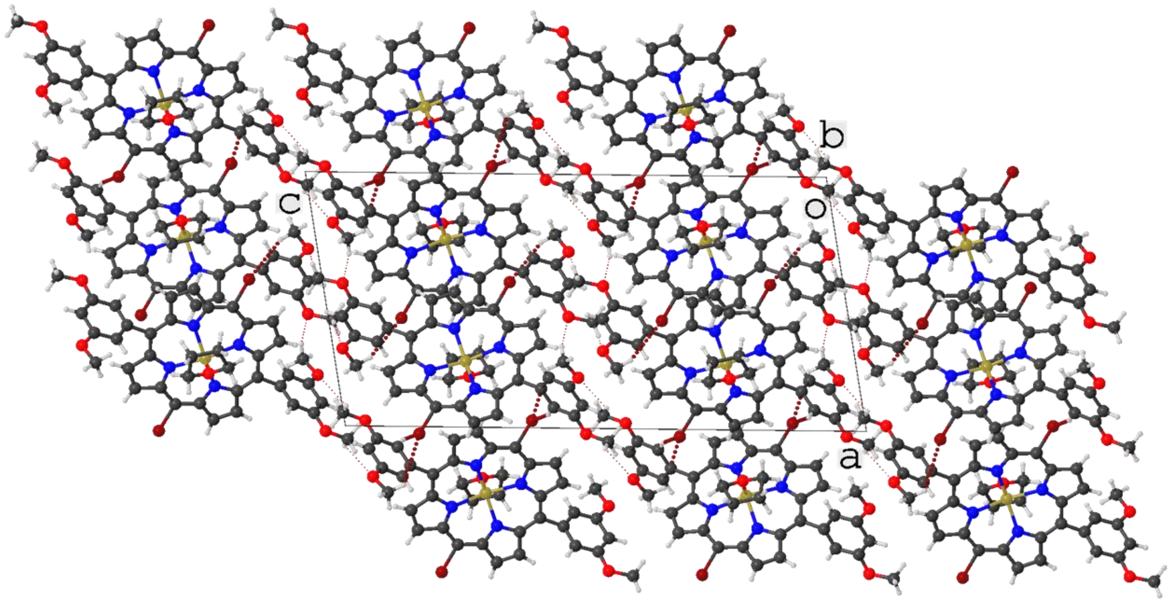

Figure S40: Packing diagrams (ball and stick) for compound **15** looking down the *b*-axis.

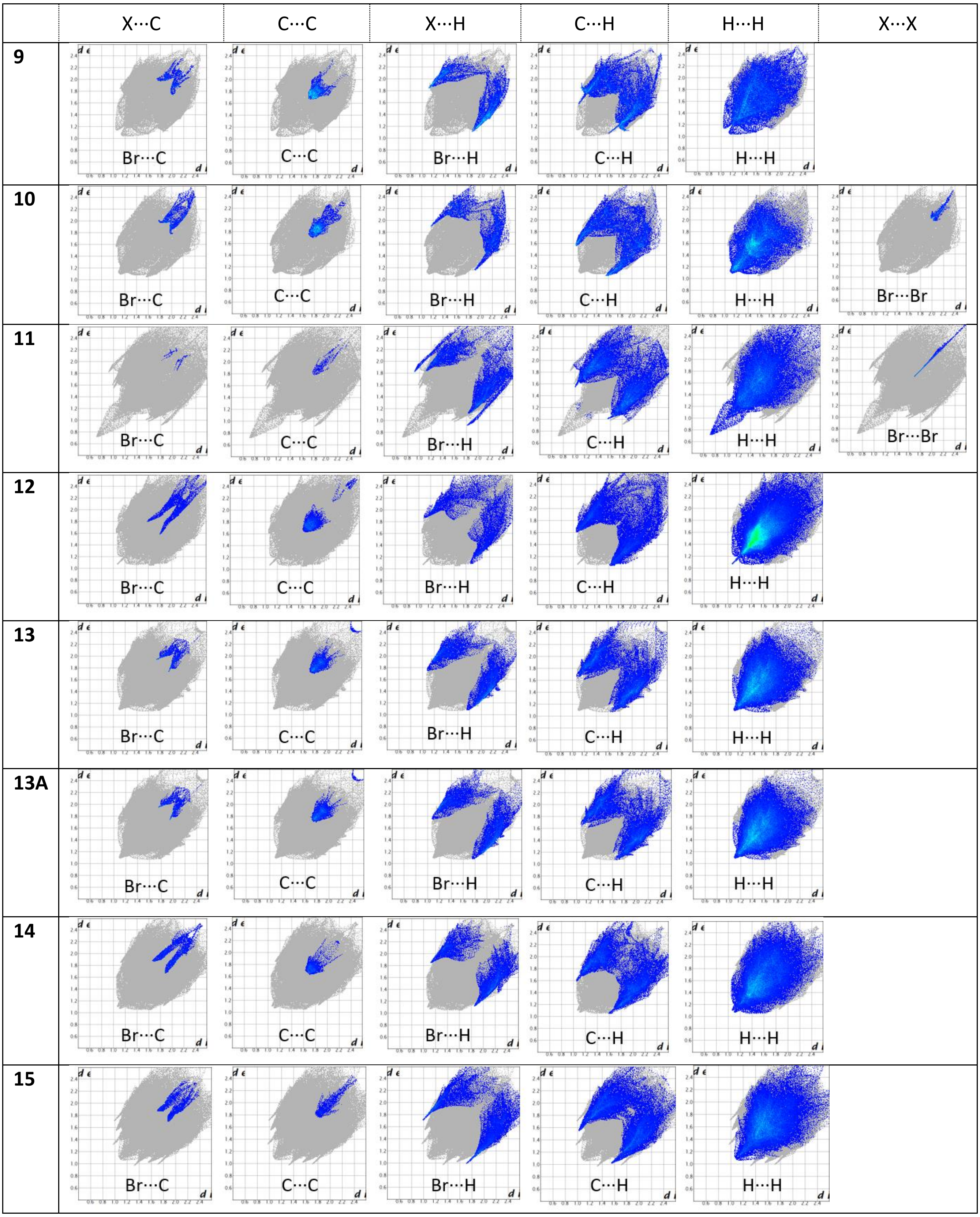

Figure S41: Hirshfeld surfaces of compounds 9-15.

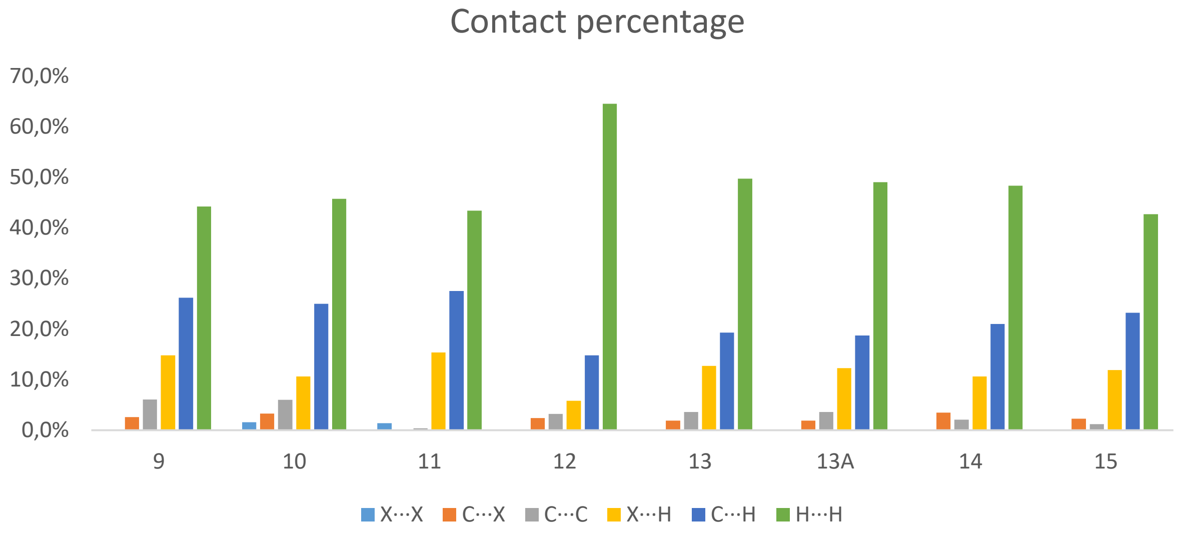

Figure S42: Contact percentages of compounds **9**-**15**.

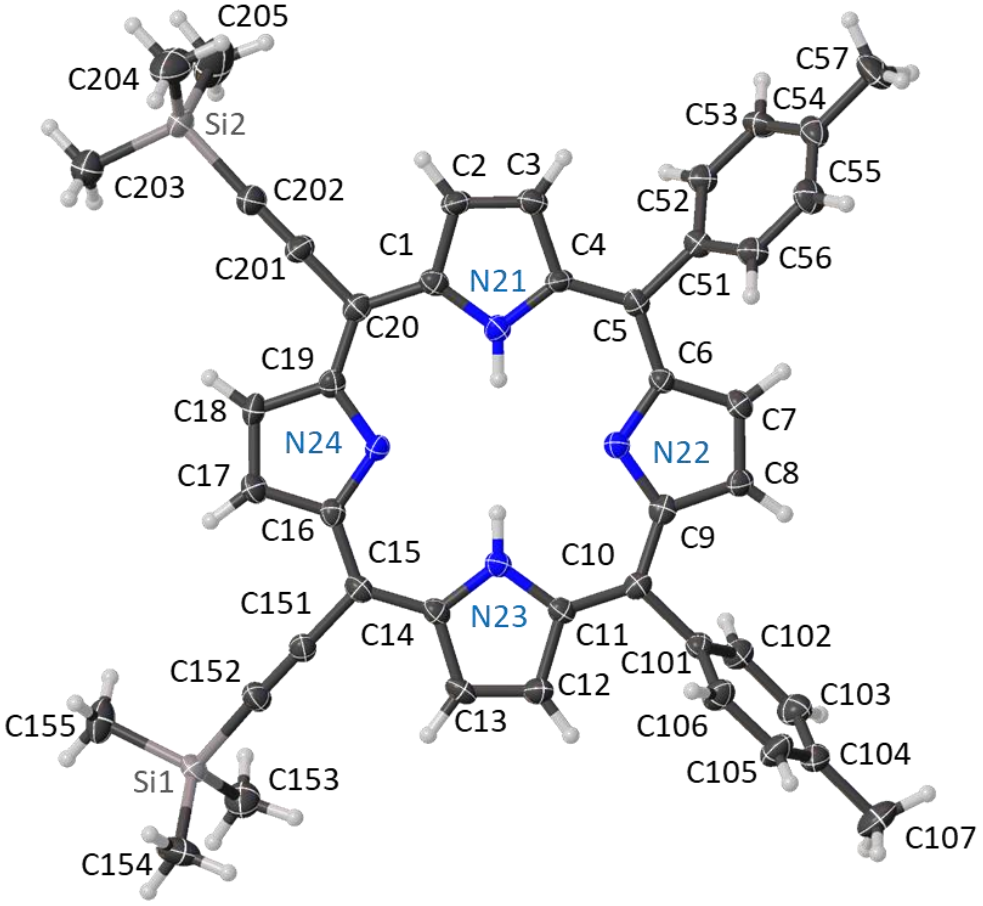

Figure S43: Thermal ellipsoid plot of compound **16A** (thermal displacement given as 50% probability). Minor disordered moieties have been omitted.

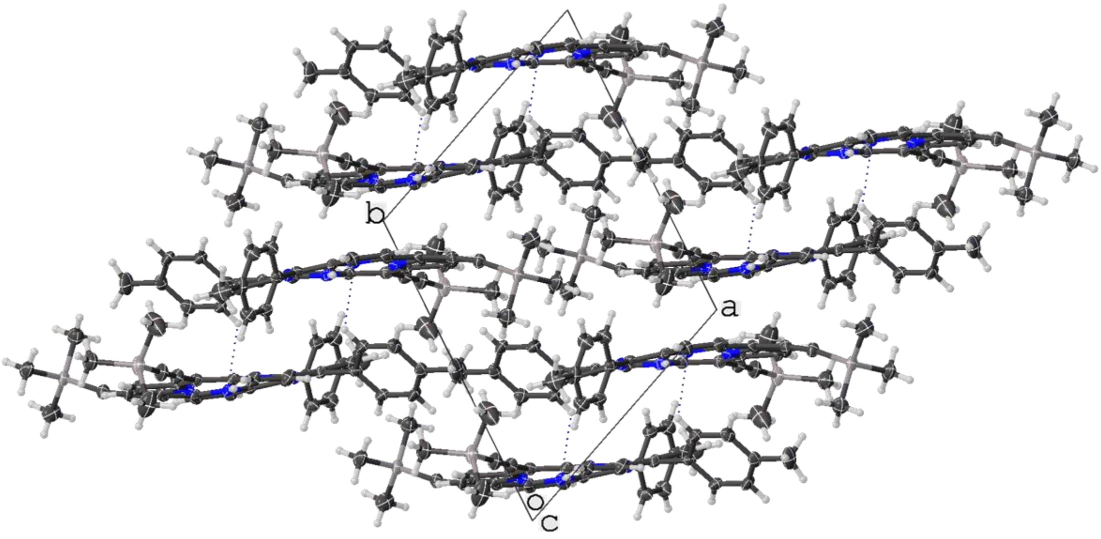

Figure S44: Packing diagram (thermal ellipsoid plot) of compound **16A** looking down the *c*-axis (thermal displacement given as 50% probability). Minor disordered moieties have been omitted.

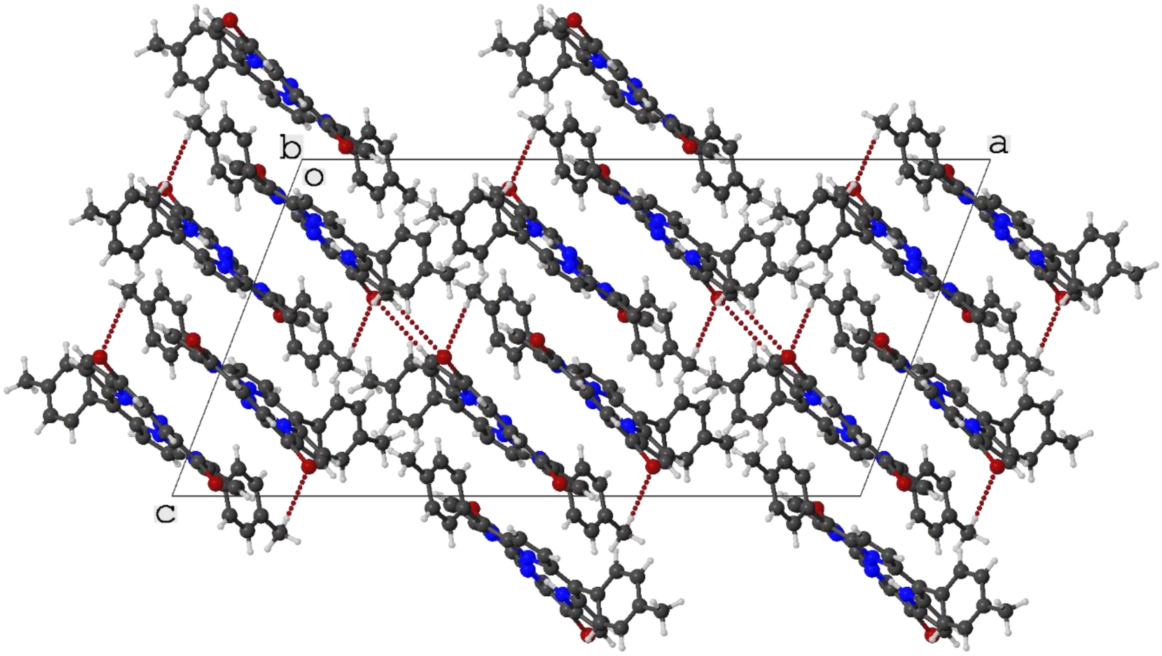

Figure S45: Packing diagram (ball and stick) of compound **16** looking down the *b*-axis.

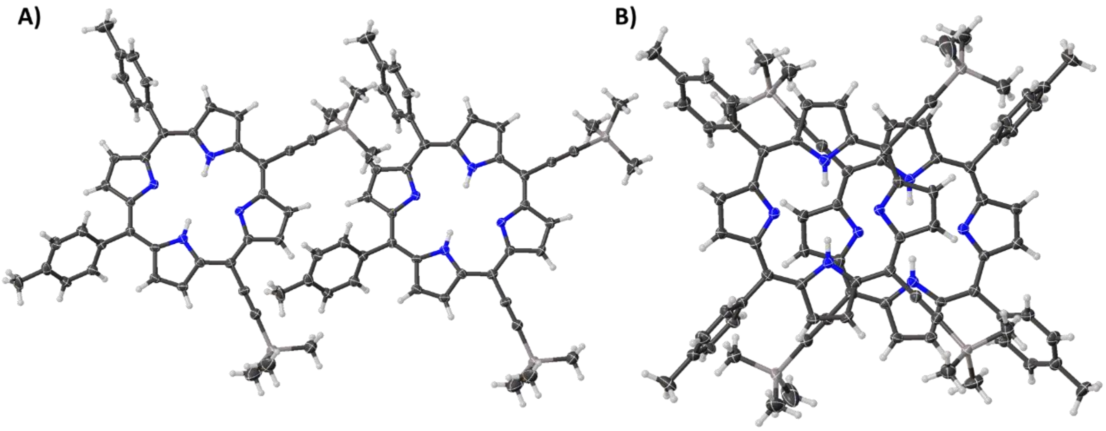

Figure S46: Expanded view (thermal ellipsoid plot) of compound **16A** showing (**A**) linear alignment and (**B**) stacking.

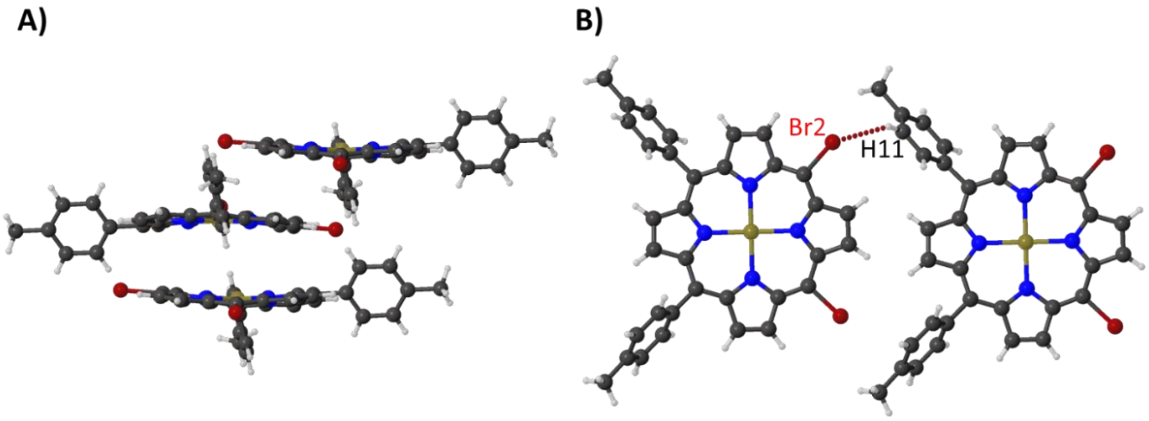

Figure S47: Expanded view (ball and stick) of compound **17** showing the (**A**) stacking and (**B**) head-to-tail alignment.

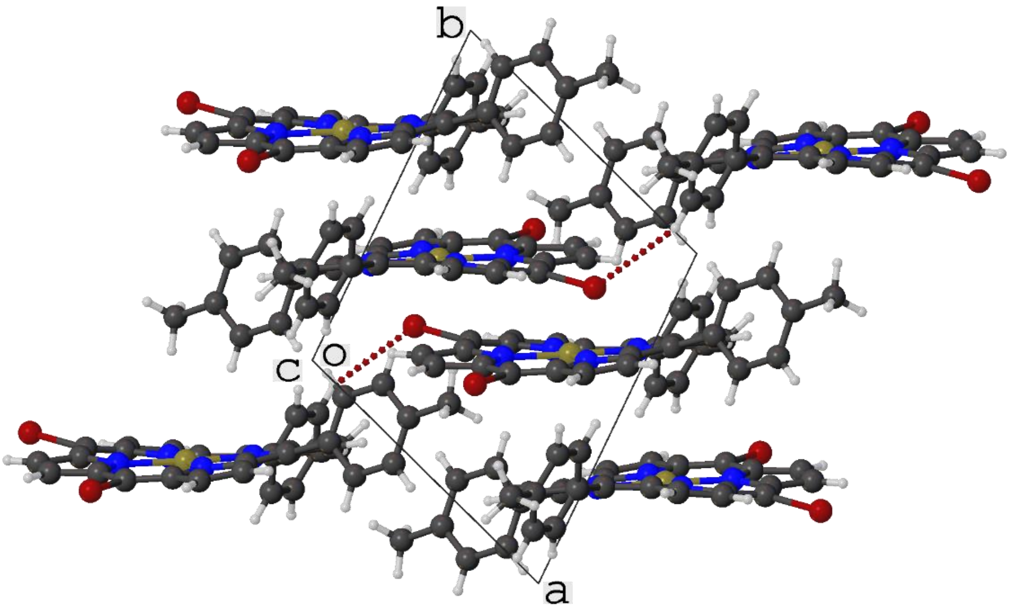

Figure S48: Packing diagram (ball and stick) of compound **17** looking down the *c*-axis.

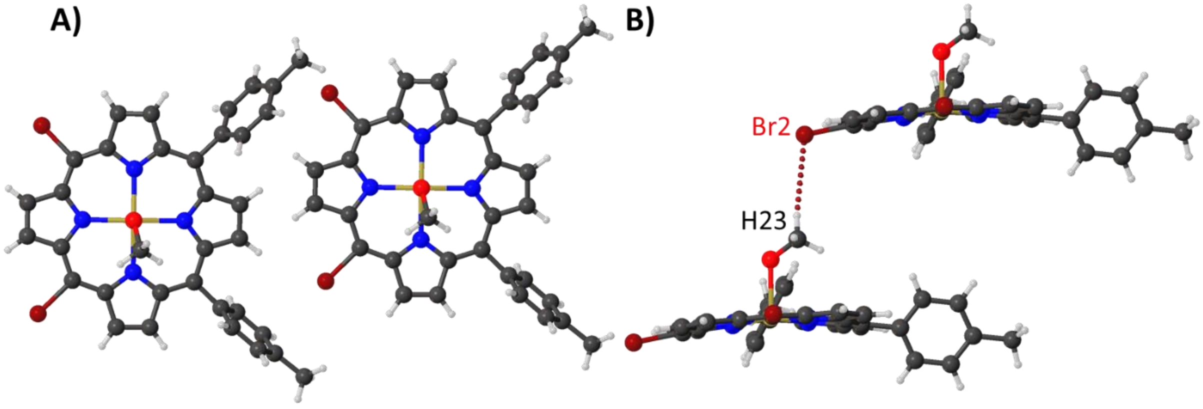

Figure S49: Expanded view (ball and stick) of compound **18** showing the (**A**) head-to-tail alignment and (**B**) interaction between the axial ligand and the bromine atoms.

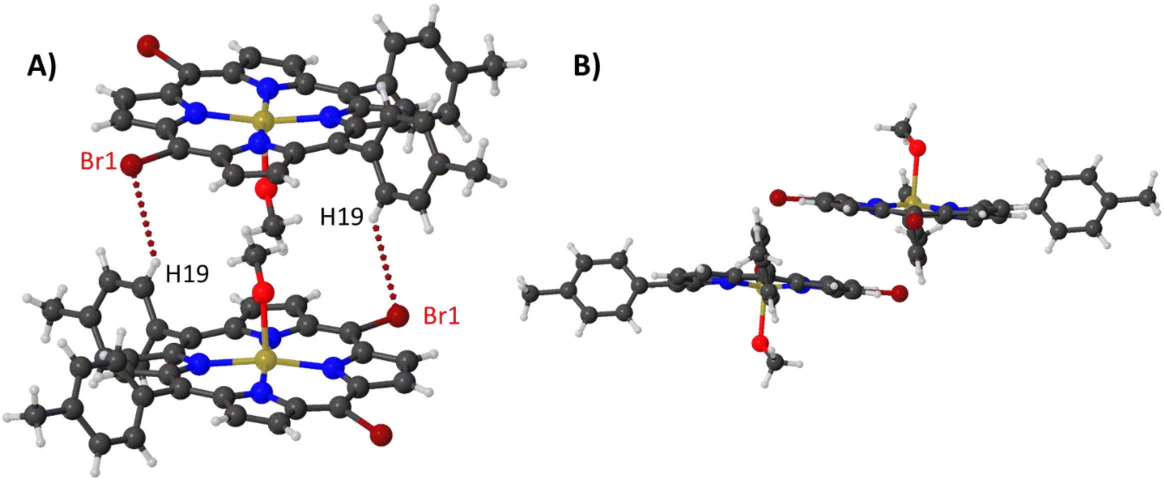

Figure S50: Expanded view (ball and stick) of compound **18** showing the stacking between (**A**) solvent side of the porphyrin and (**B**) non-solvent side.

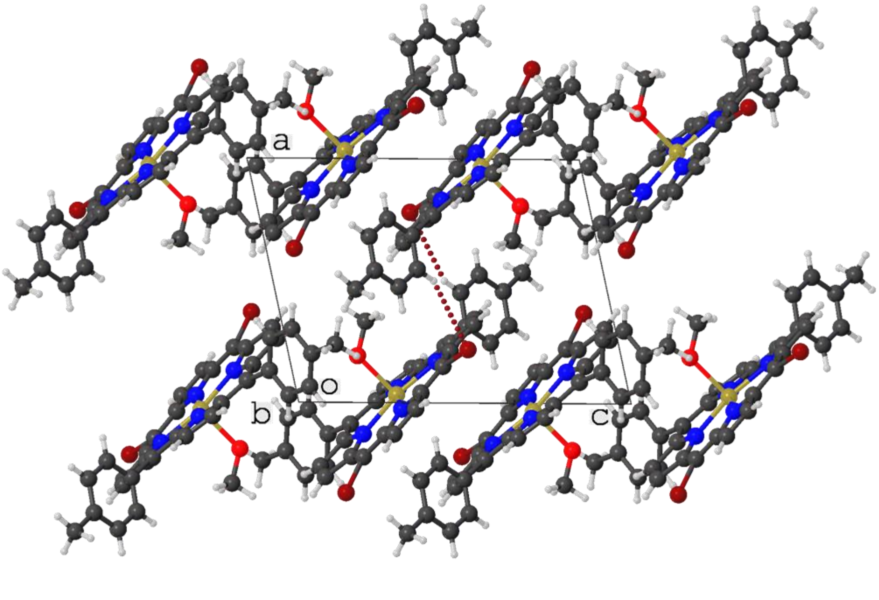

Figure S51: Packing diagram (ball and stick) of compound **18** looking down the *b*-axis.

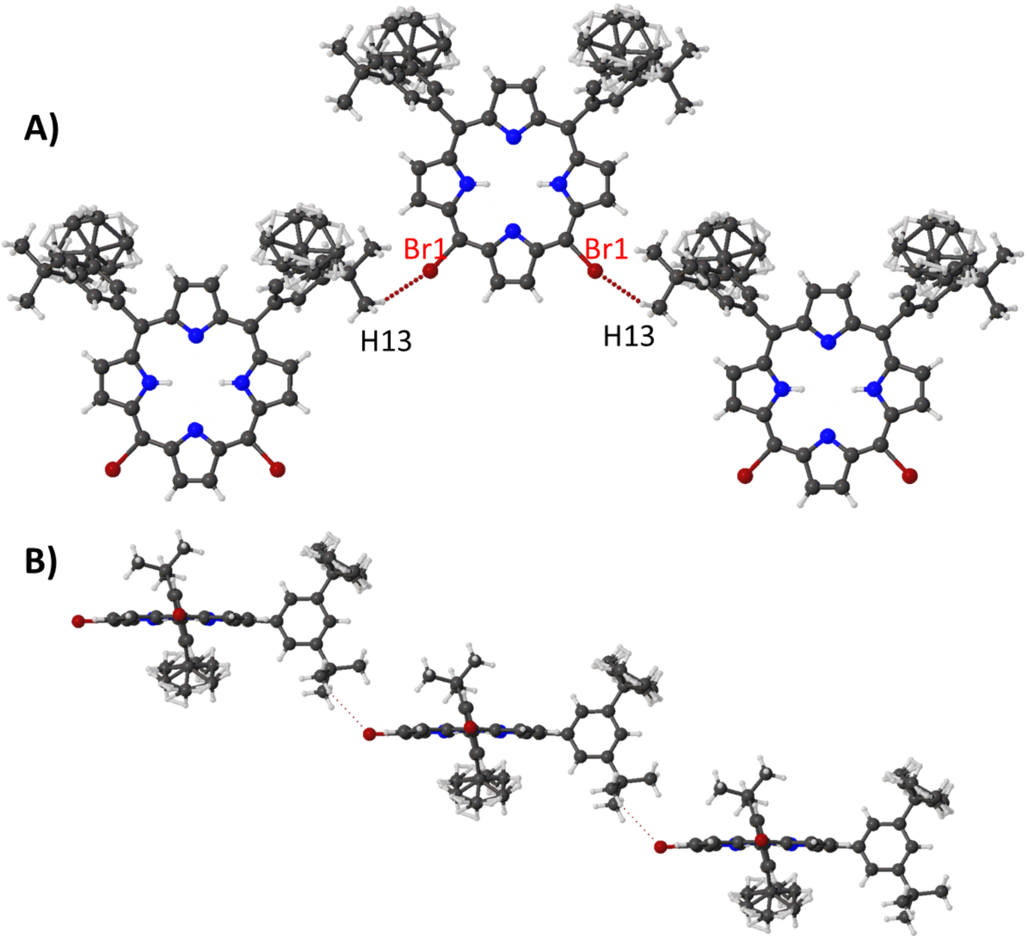

Figure S52: Expanded view (ball and stick) of compound **19** showing the stepwise alignment of porphyrins with Br···H interactions (**A**) top and (**B**) side.

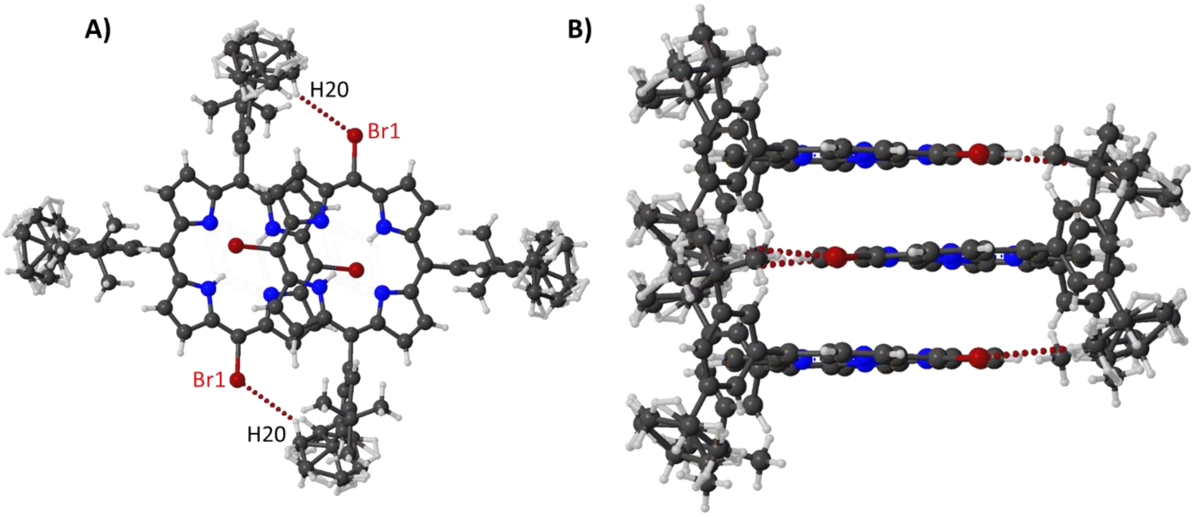

Figure S53: Expanded view (ball and stick) of compound **19** showing the (**A**) top view of stacking and (**B**) edge-on view of stacking.

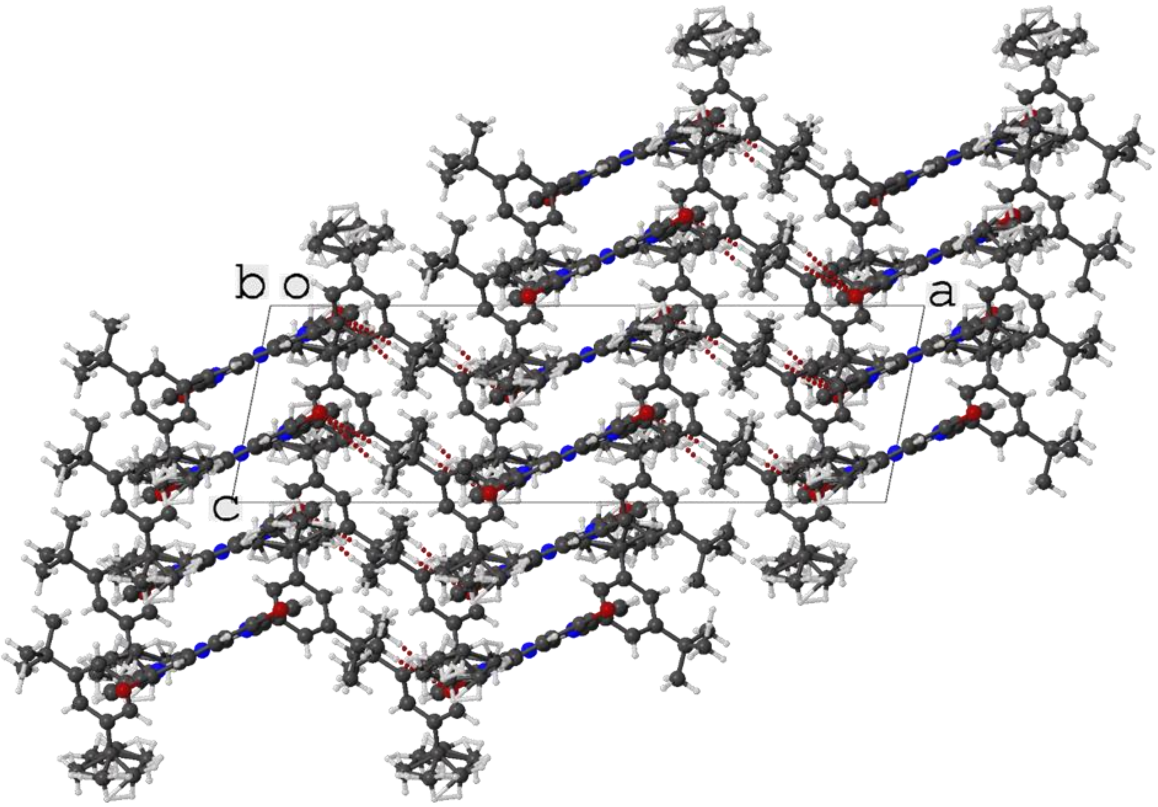

Figure S54: Packing diagram (ball and stick) of compound **19** looking down the *b*-axis.

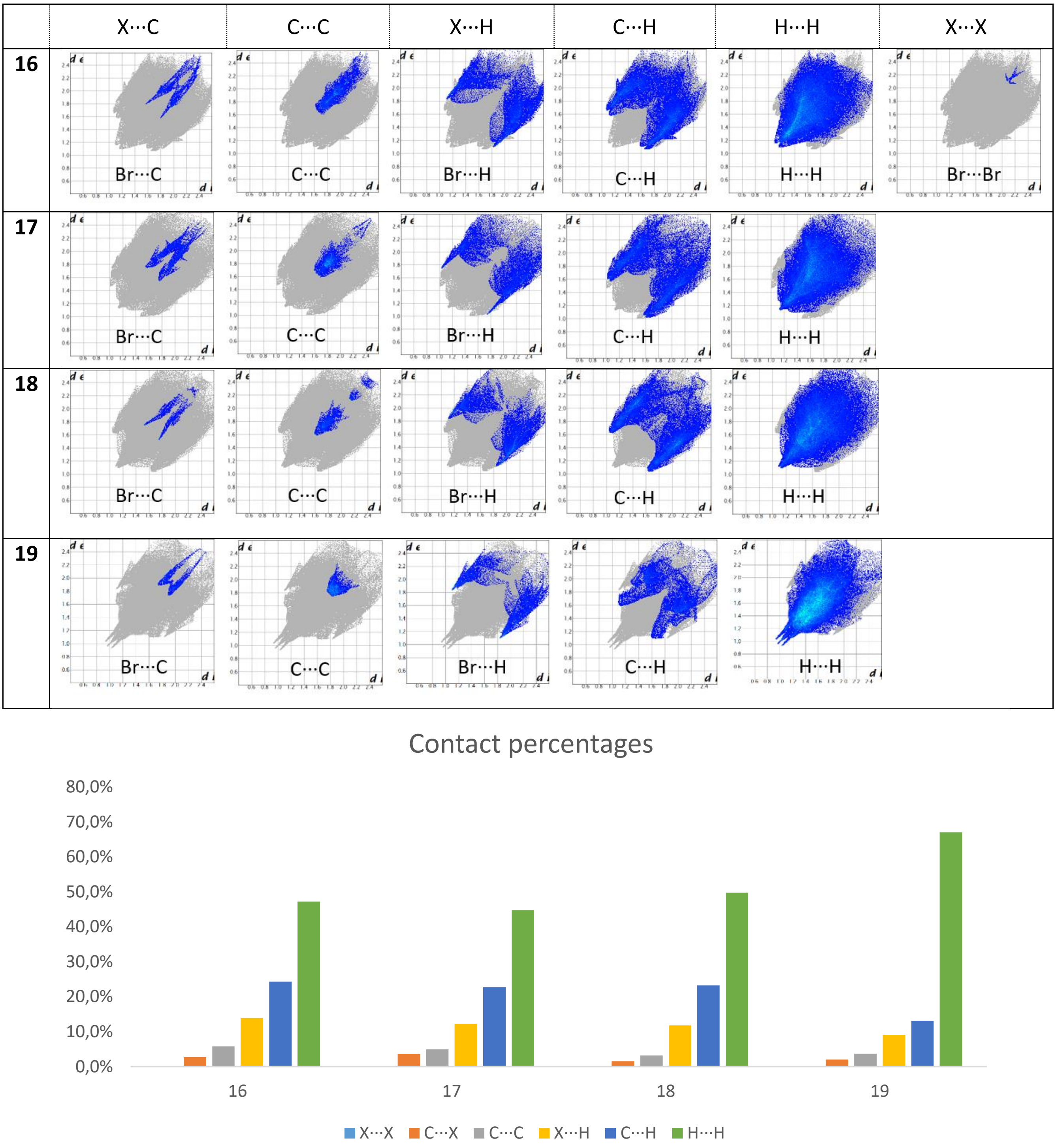

Figure S55: Hirshfeld surfaces and contact percentages of compounds **16**-**19**.

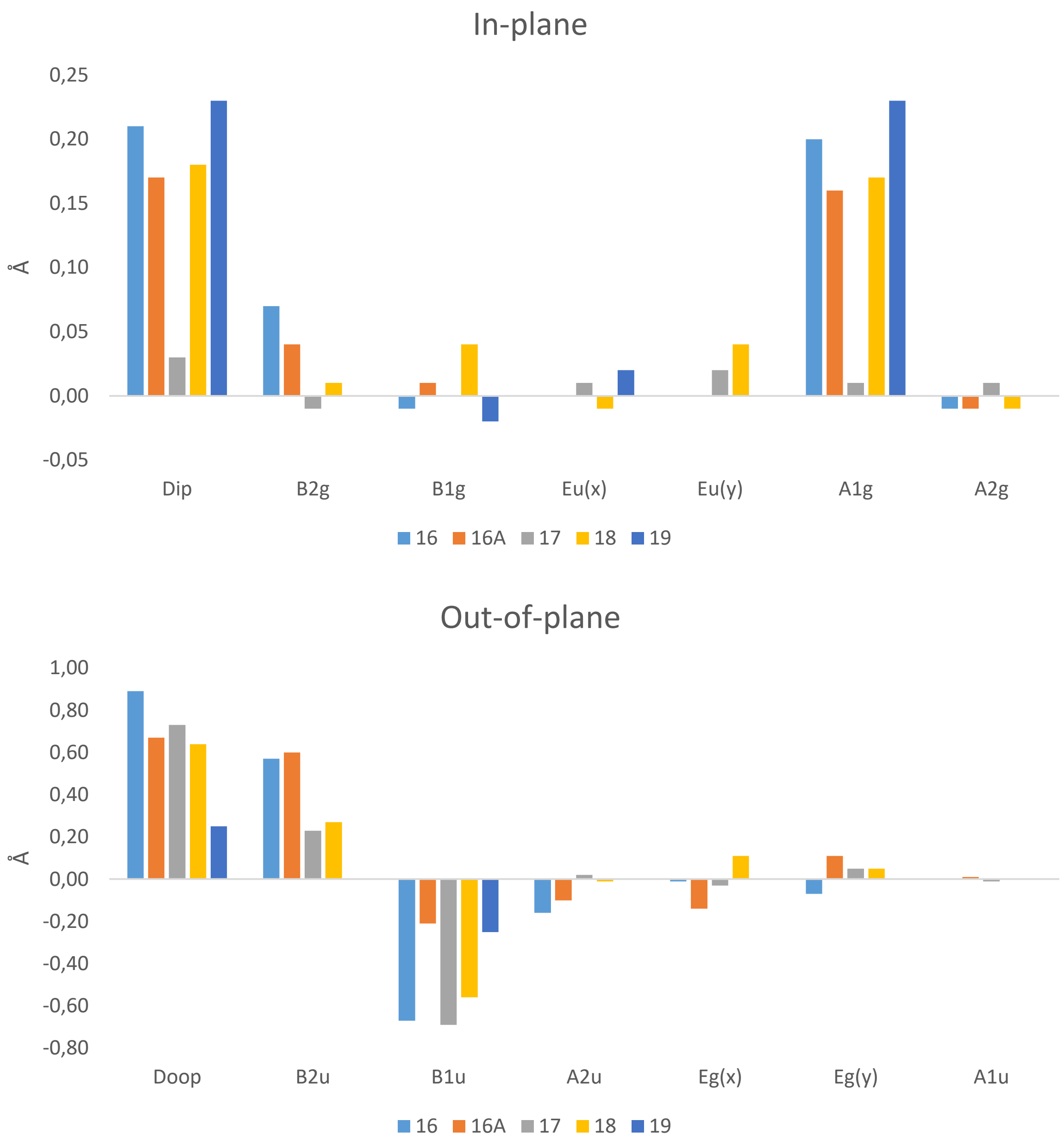

Figure S56: NSD charts for compounds **16**-**19**.

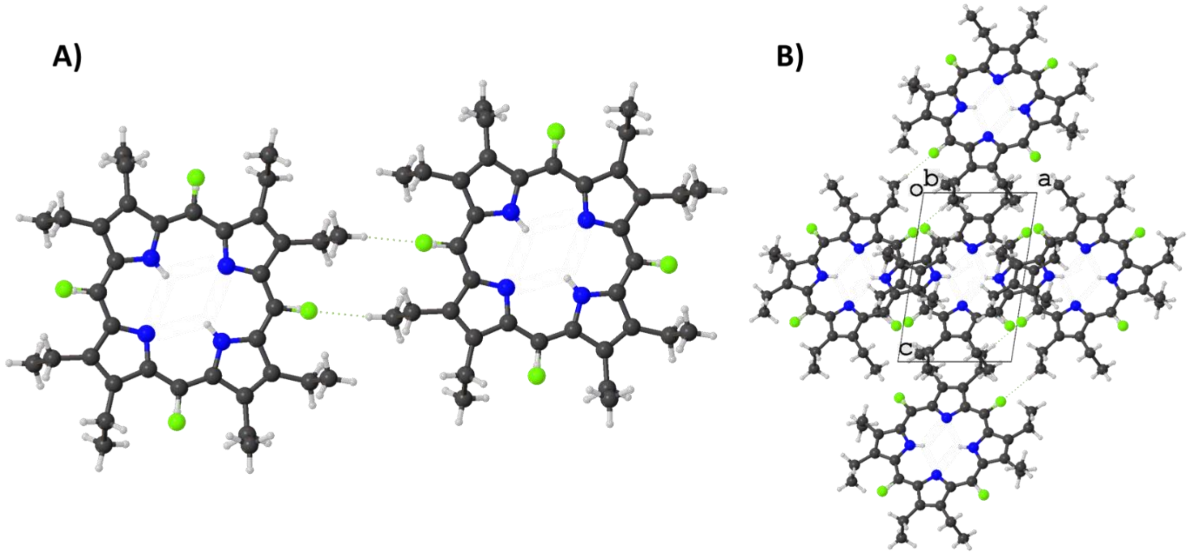

Figure S57: (**A**) Expanded view (ball and stick) of compound **20** showing the F···H interaction and (**B**) crystal packing (ball and stick) of compound **20**.

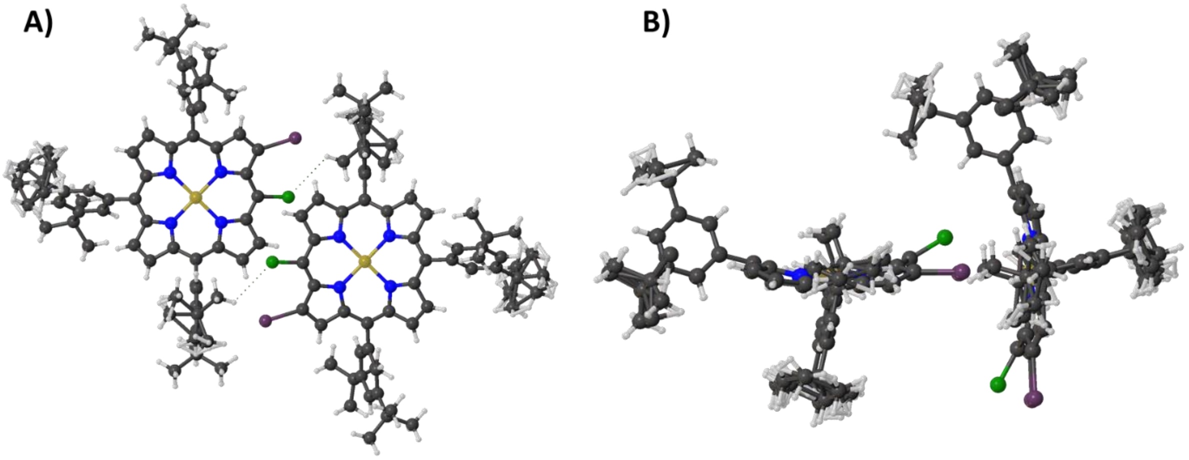

Figure S58: (**A**) Expanded view (ball and stick) of compound **21** showing the head-to-head interaction (**B**) Expanded view (ball and stick) of compound **22** showing the face-to-edge interaction.

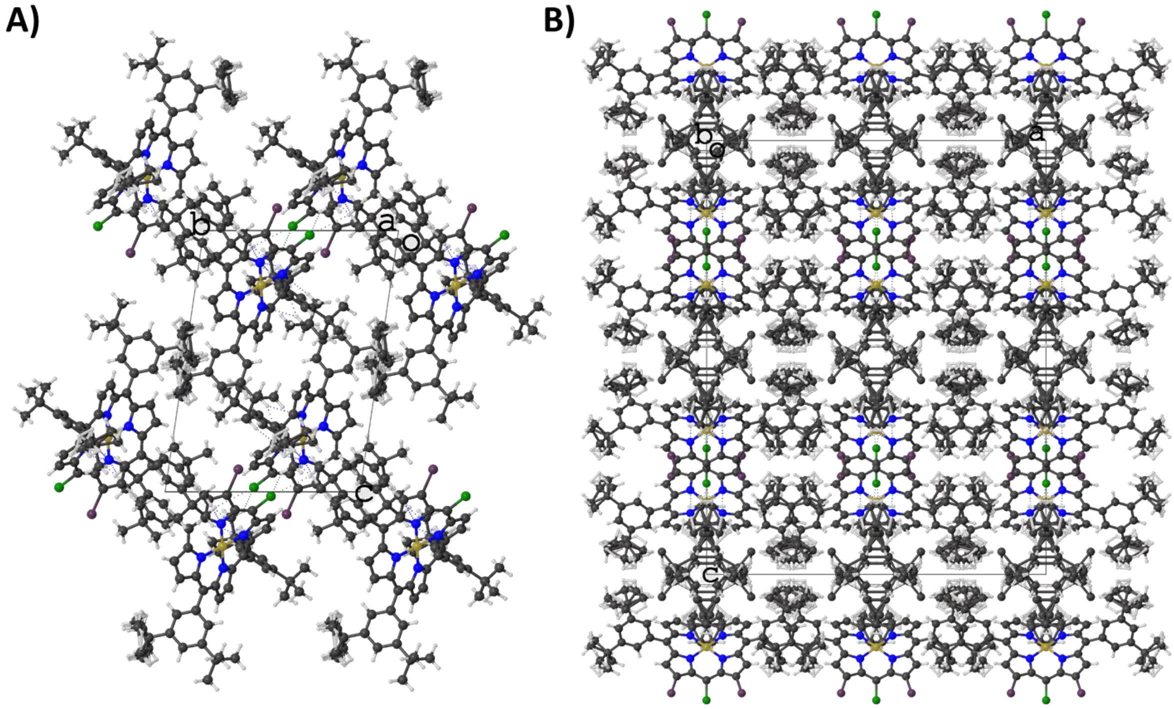

Figure S59: Packing diagram (ball and stick) of compound **21** looking down the *a*-axis (**A**) and **22** looking down the *b*-axis (**B**).

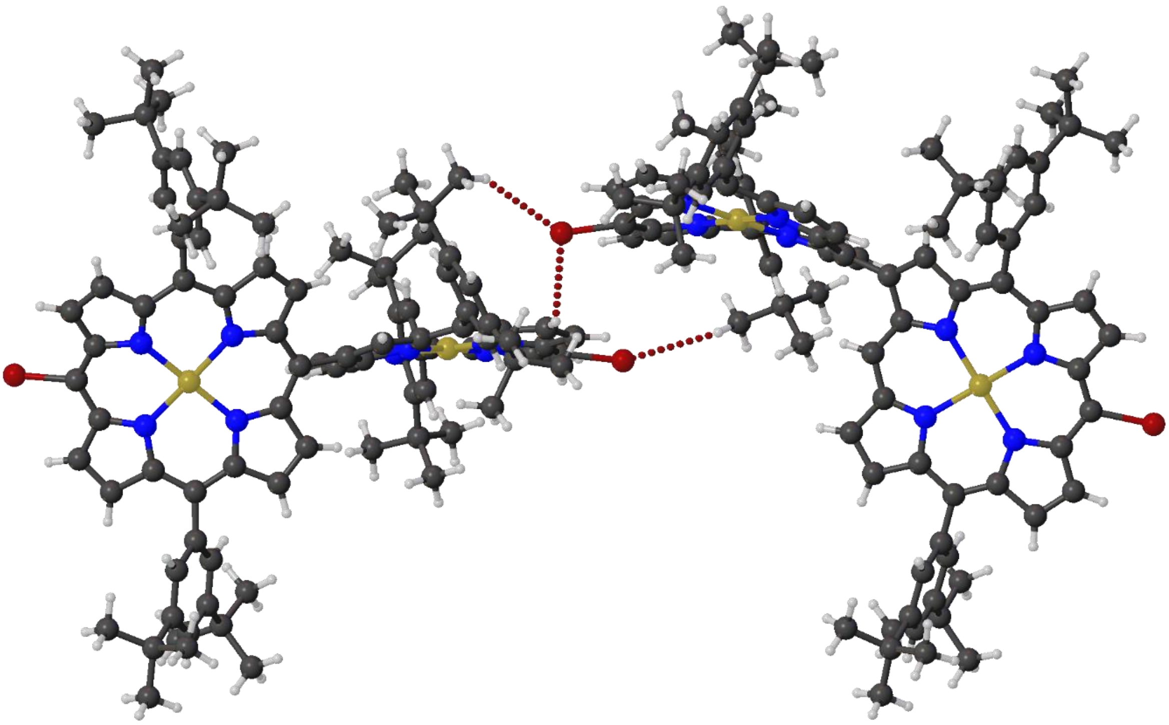

Figure S60: Expanded view (ball and stick) of compound **23** showing the head-to-tail interaction.

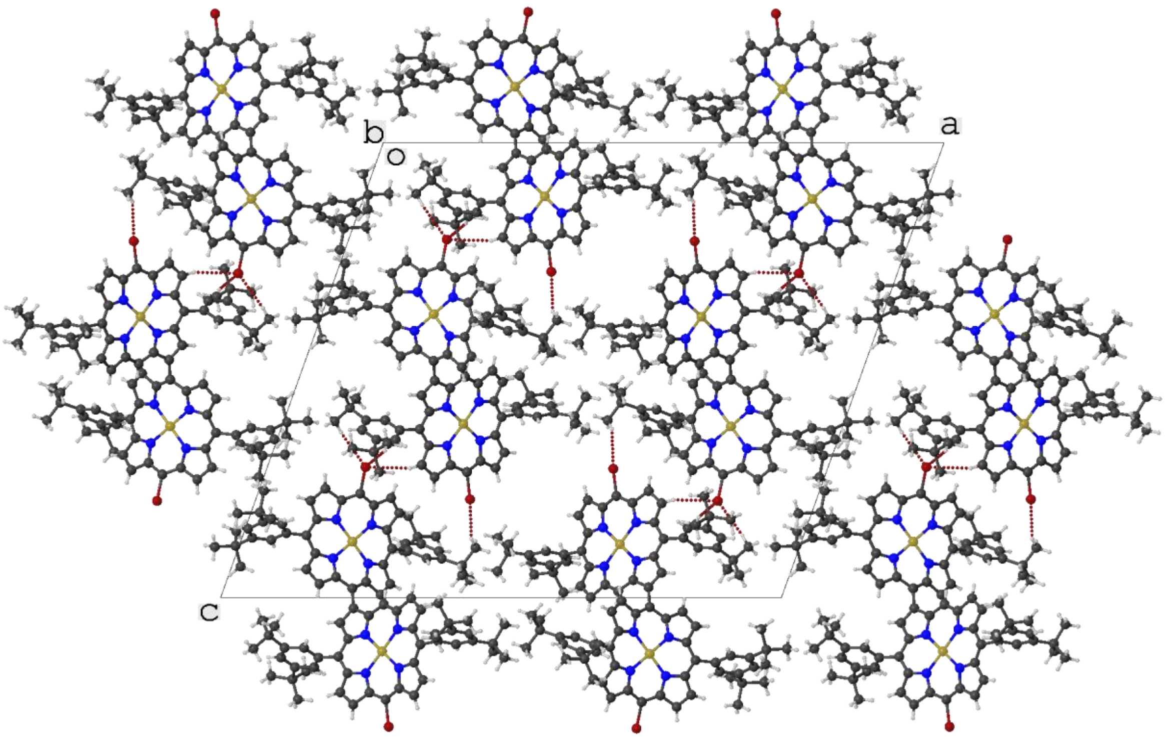

Figure S61: Packing diagram (ball and stick) of compound **23** looking down the *b*-axis.

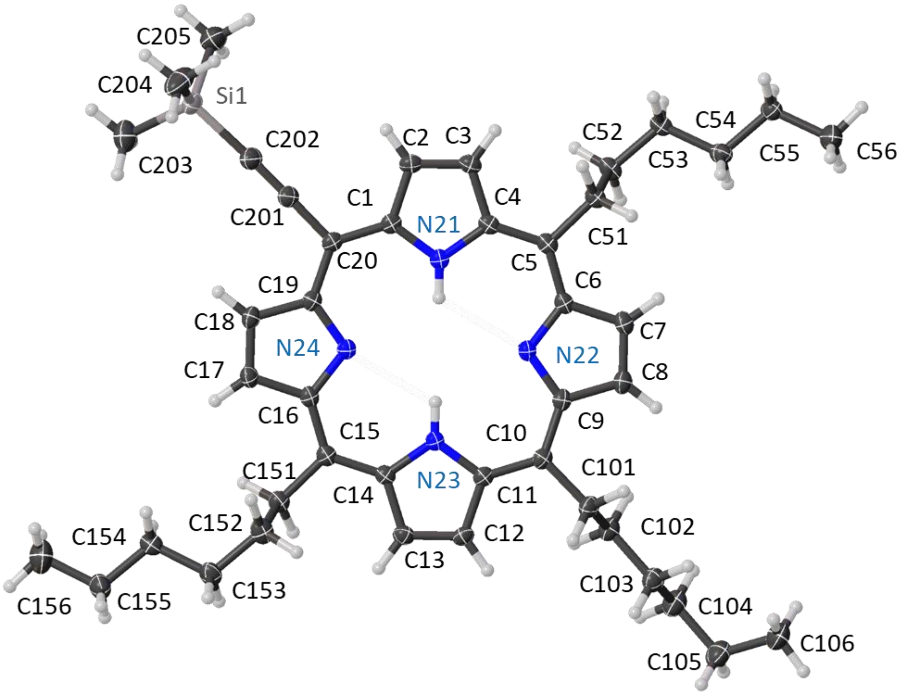

Figure S62: Thermal ellipsoid plot of compound **24** (thermal displacement given as 50% probability). Minor disordered moieties have been omitted.

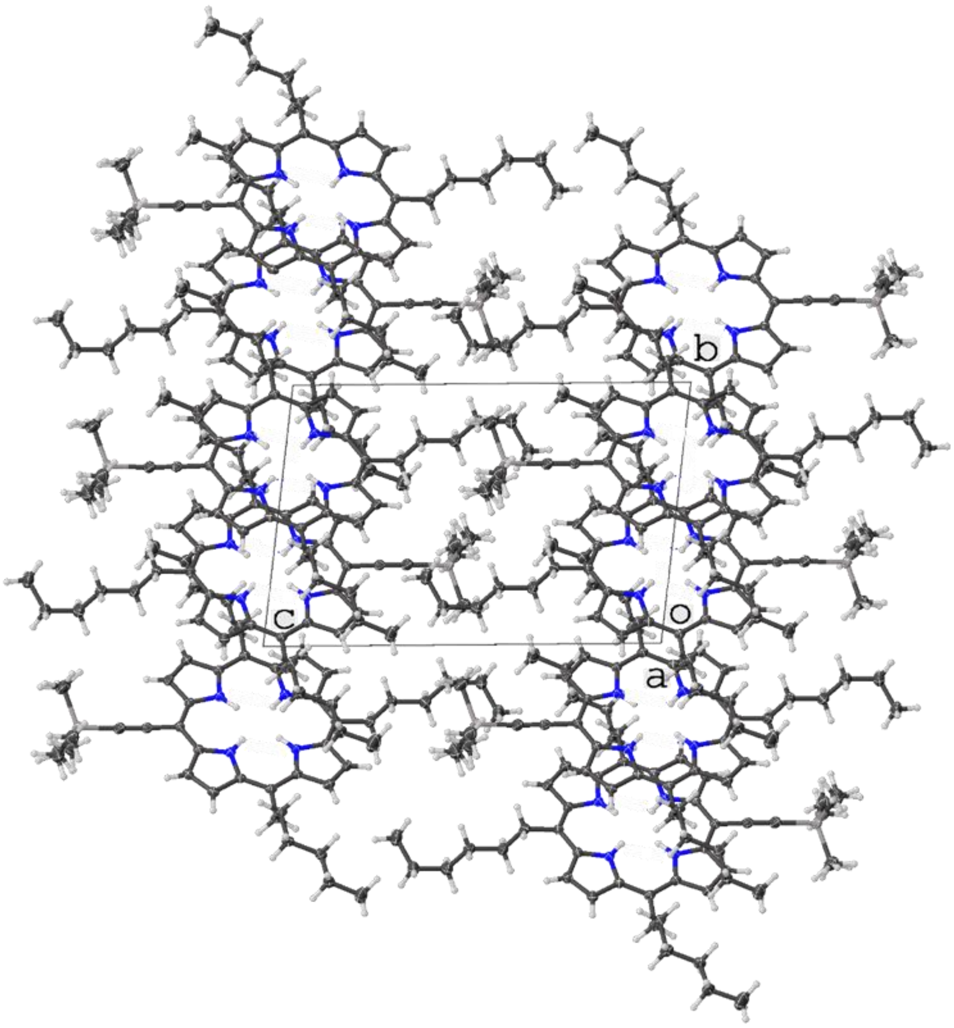

Figure S63: Packing diagram (thermal ellipsoid plot) of compound **24** looking down the *a*-axis (thermal displacement given as 50% probability). Minor disordered moieties have been omitted.

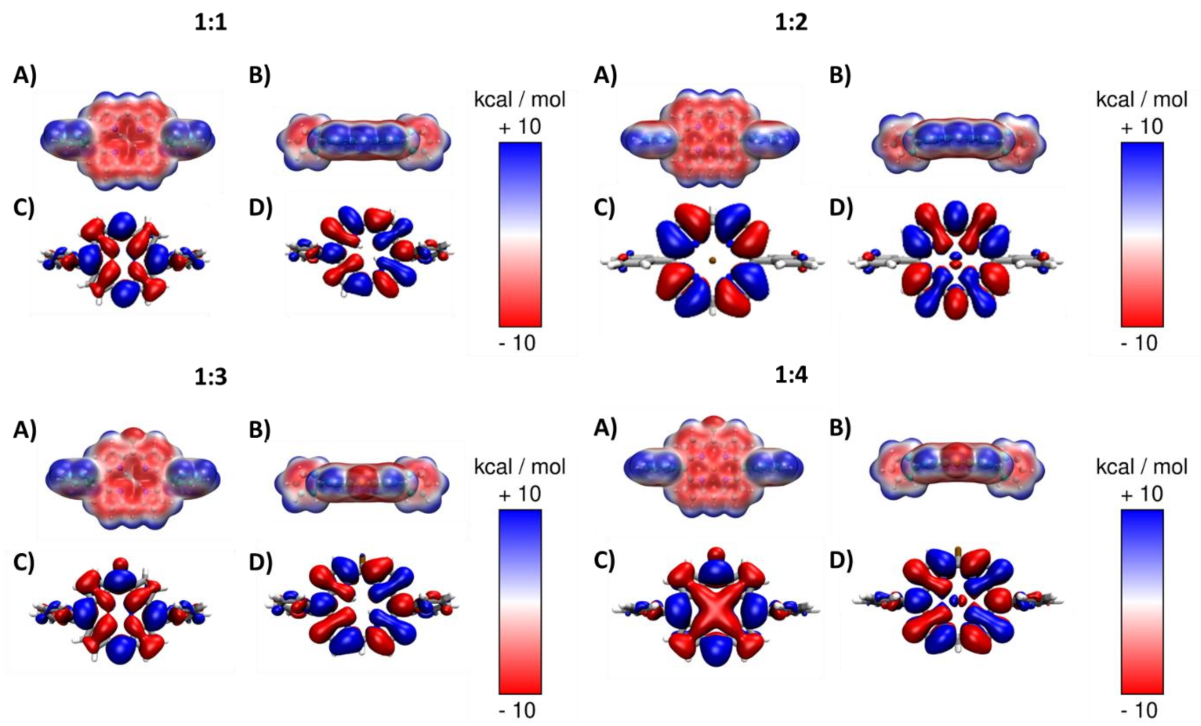

Figure S64: DFT generated images of compounds **1:1-1:4** (**A**) MEP map top view, (**B**) MEP map side view, (**C**) electron density distribution of HOMO, (**D**) electron density distribution of LUMO.

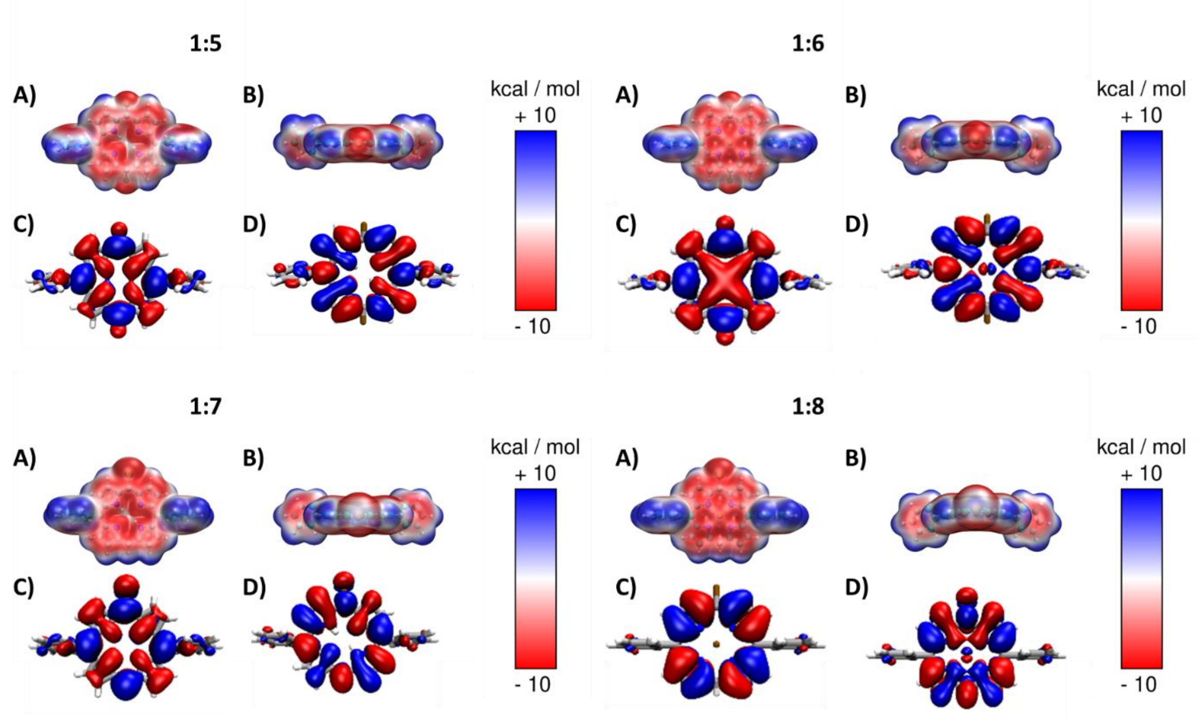

Figure S65: DFT generated images of compounds **1:5-1:8** (**A**) MEP map top view, (**B**) MEP map side view, (**C**) electron density distribution of HOMO, (**D**) electron density distribution of LUMO.

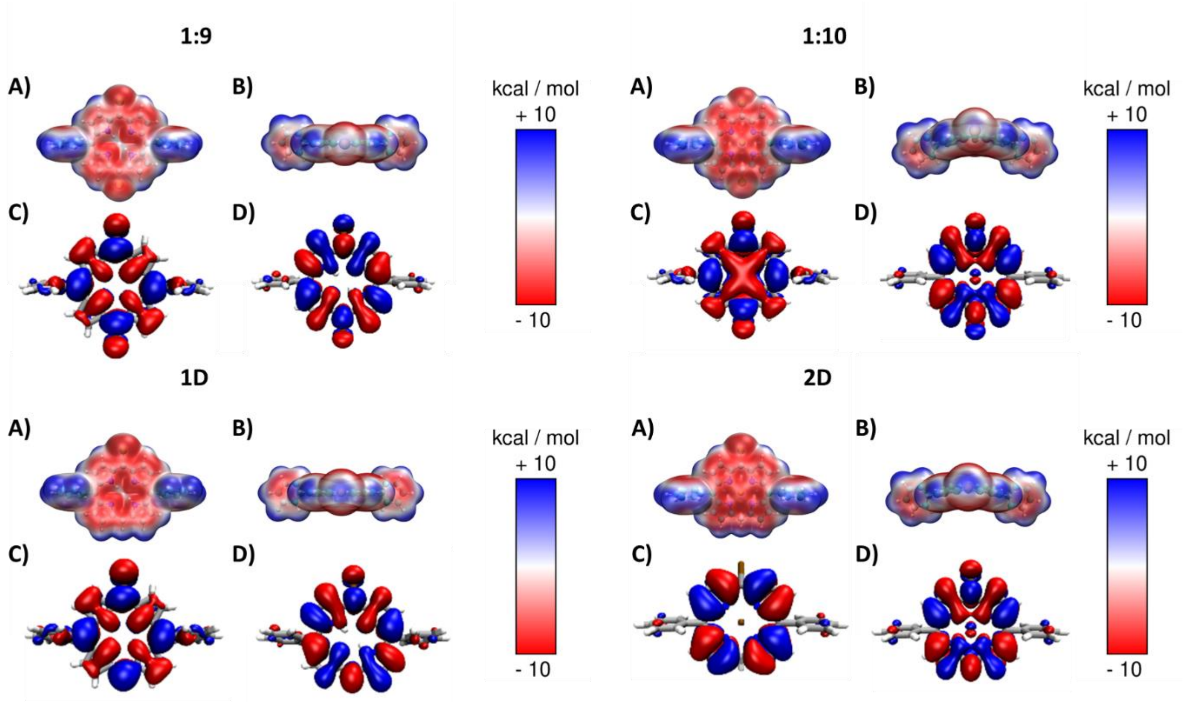

Figure S66: DFT generated images of compounds **1:9**, **1:10**, **1D**, and **2D** (**A**) MEP map top view, (**B**) MEP map side view, (**C**) electron density distribution of HOMO, (**D**) electron density distribution of LUMO.

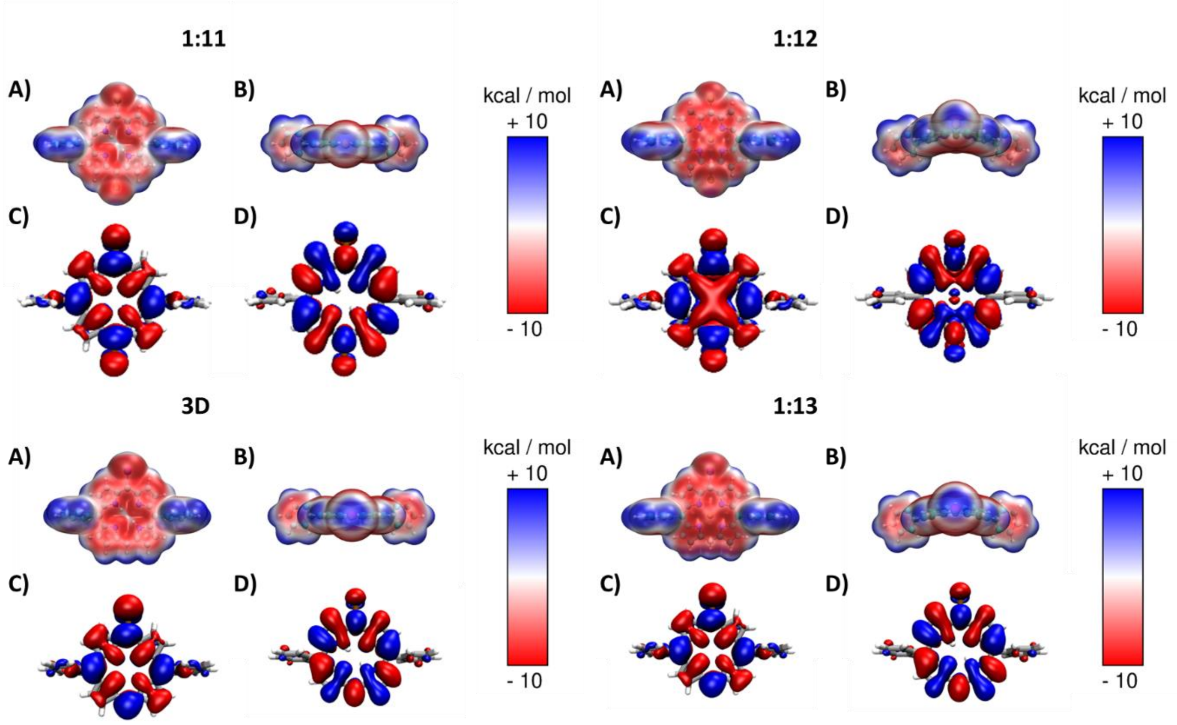

Figure S67: DFT generated images of compounds **1:11-1:13** and **3D** (**A**) MEP map top view, (**B**) MEP map side view, (**C**) electron density distribution of HOMO, (**D**) electron density distribution of LUMO.

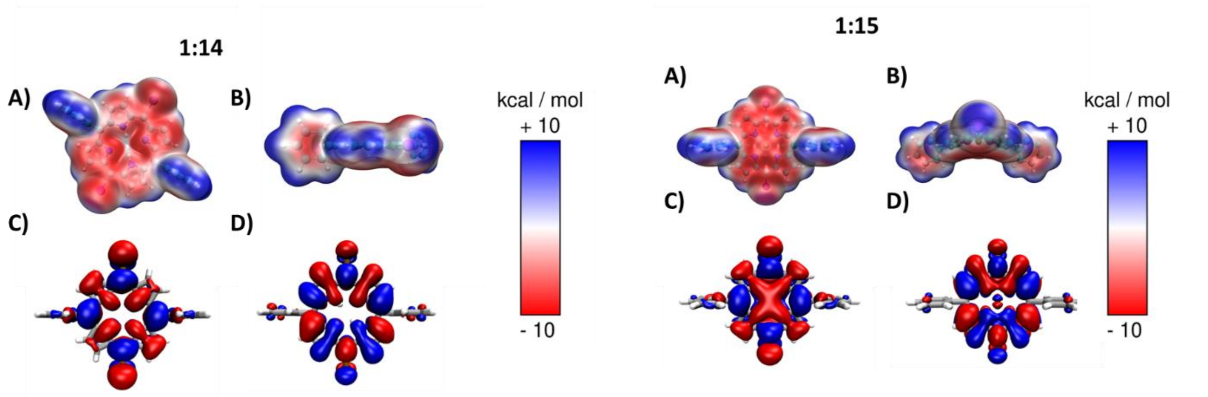

Figure S68: DFT generated images of compounds **1:14** and **1:15** (**A**) MEP map top view, (**B**) MEP map side view, (**C**) electron density distribution of HOMO, (**D**) electron density distribution of LUMO.

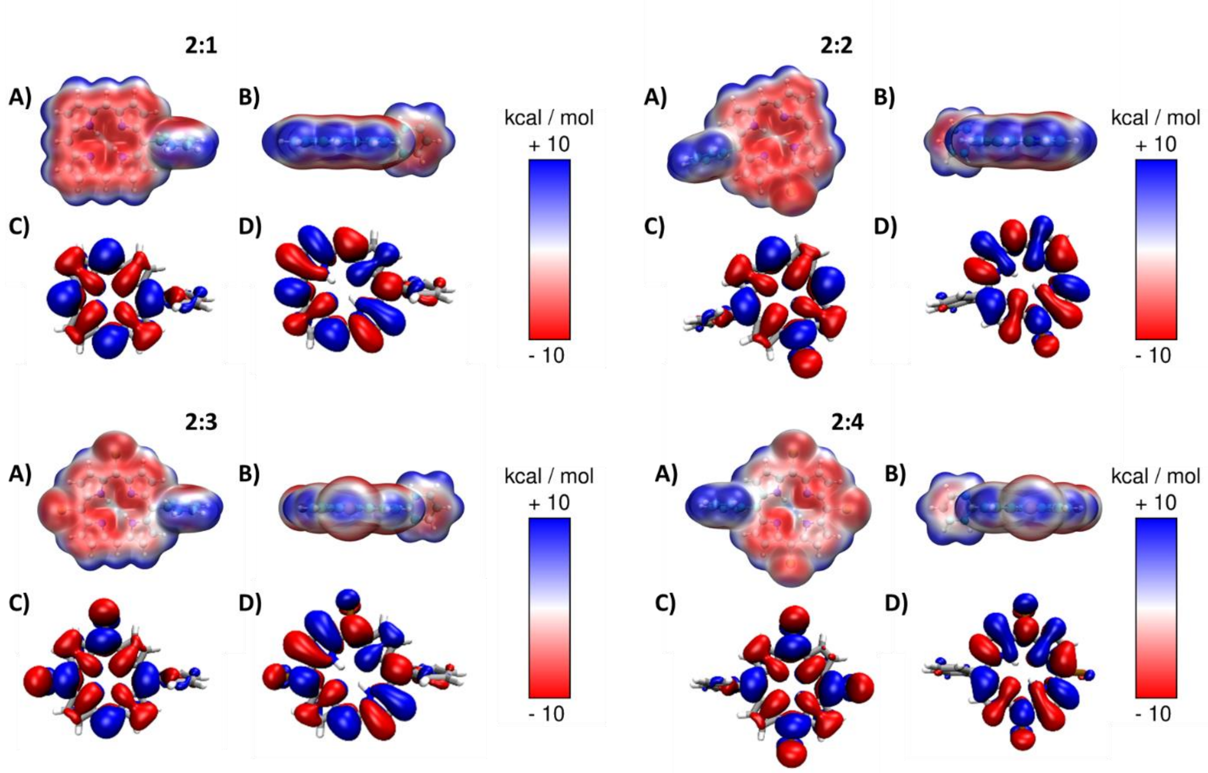

Figure S69: DFT generated images of compounds **2:1-2:4** (**A**) MEP map top view, (**B**) MEP map side view, (**C**) electron density distribution of HOMO, (**D**) electron density distribution of LUMO.

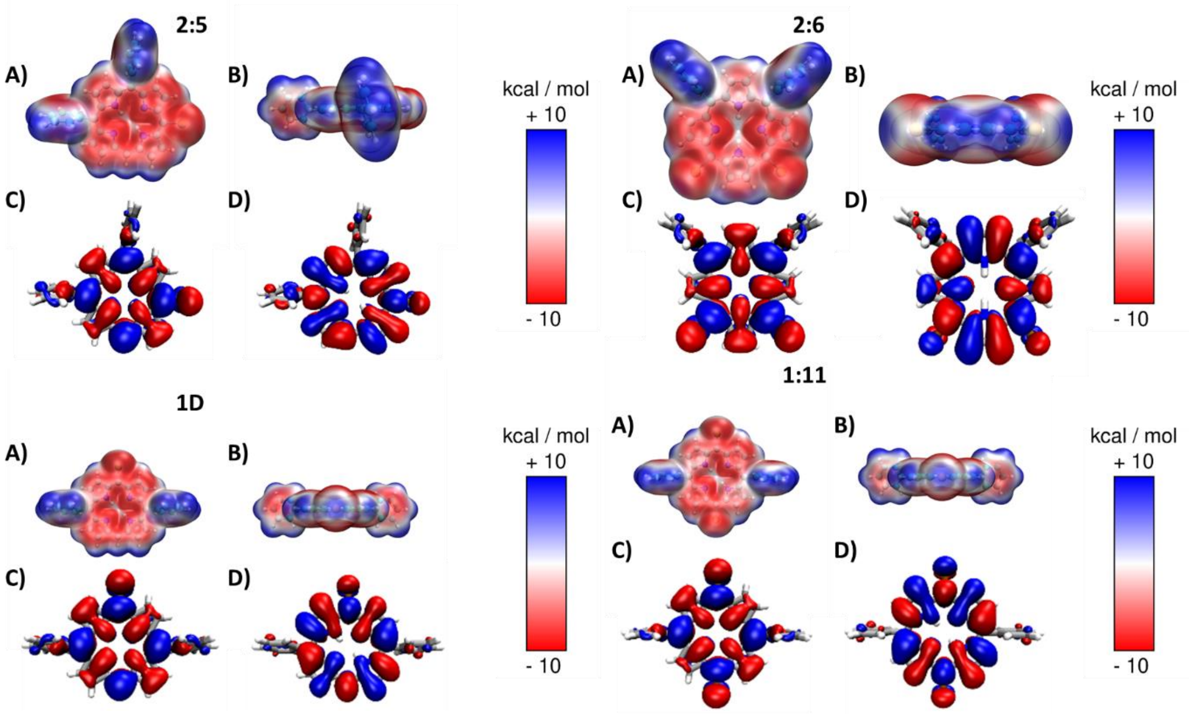

Figure S70: DFT generated images of compounds **2:5**, **2:6**, **1D**, and **1:11** (**A**) MEP map top view, (**B**) MEP map side view, (**C**) electron density distribution of HOMO, (**D**) electron density distribution of LUMO.

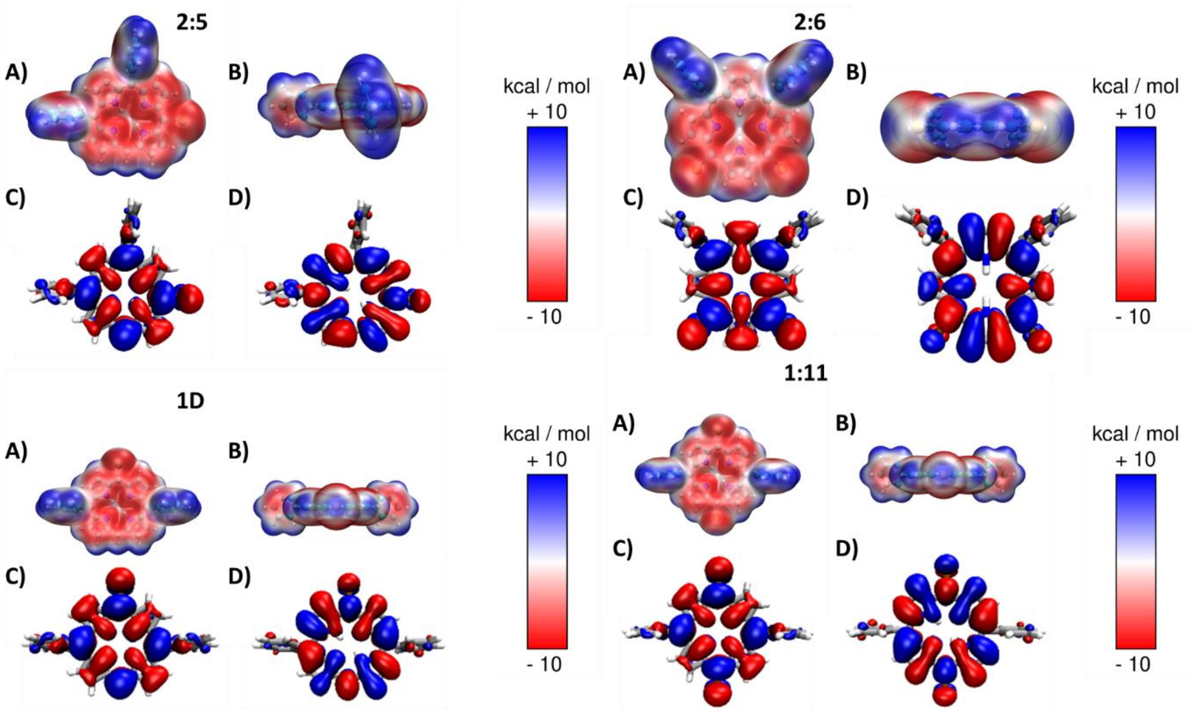

Figure S71: DFT generated images of compounds **3:1-3:3** and **2:6** (**A**) MEP map top view, (**B**) MEP map side view, (**C**) electron density distribution of HOMO, (**D**) electron density distribution of LUMO.

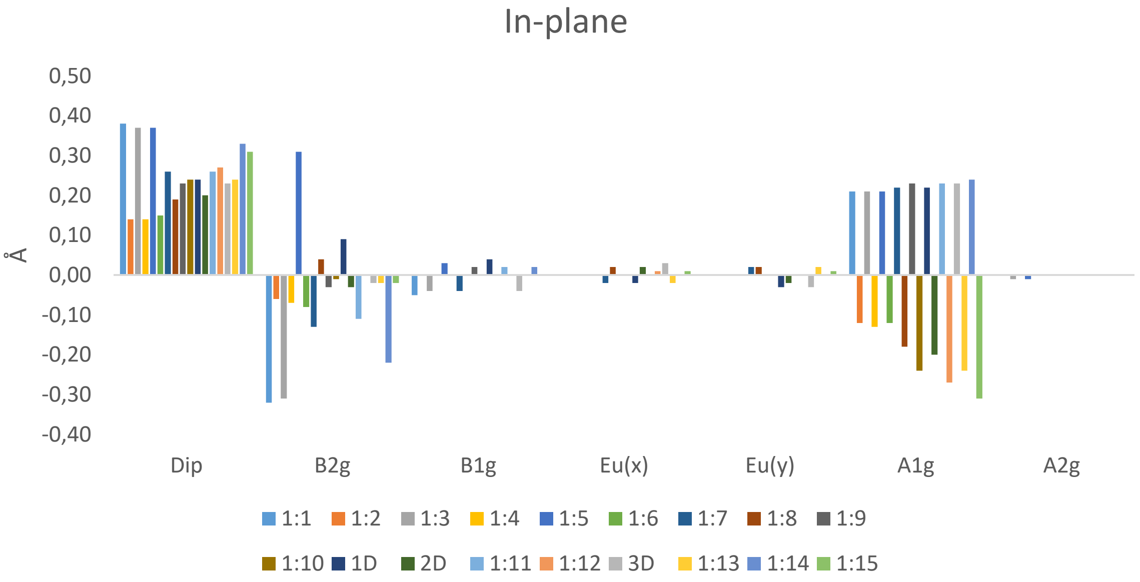

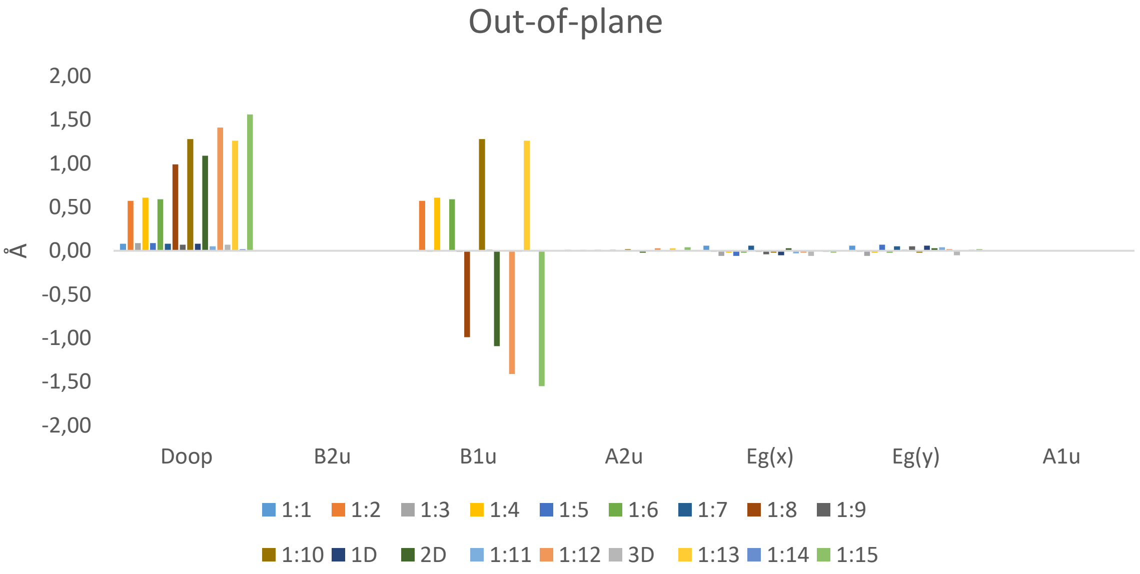

Figure S72: NSD charts for compounds **1:1**-**1:15**.

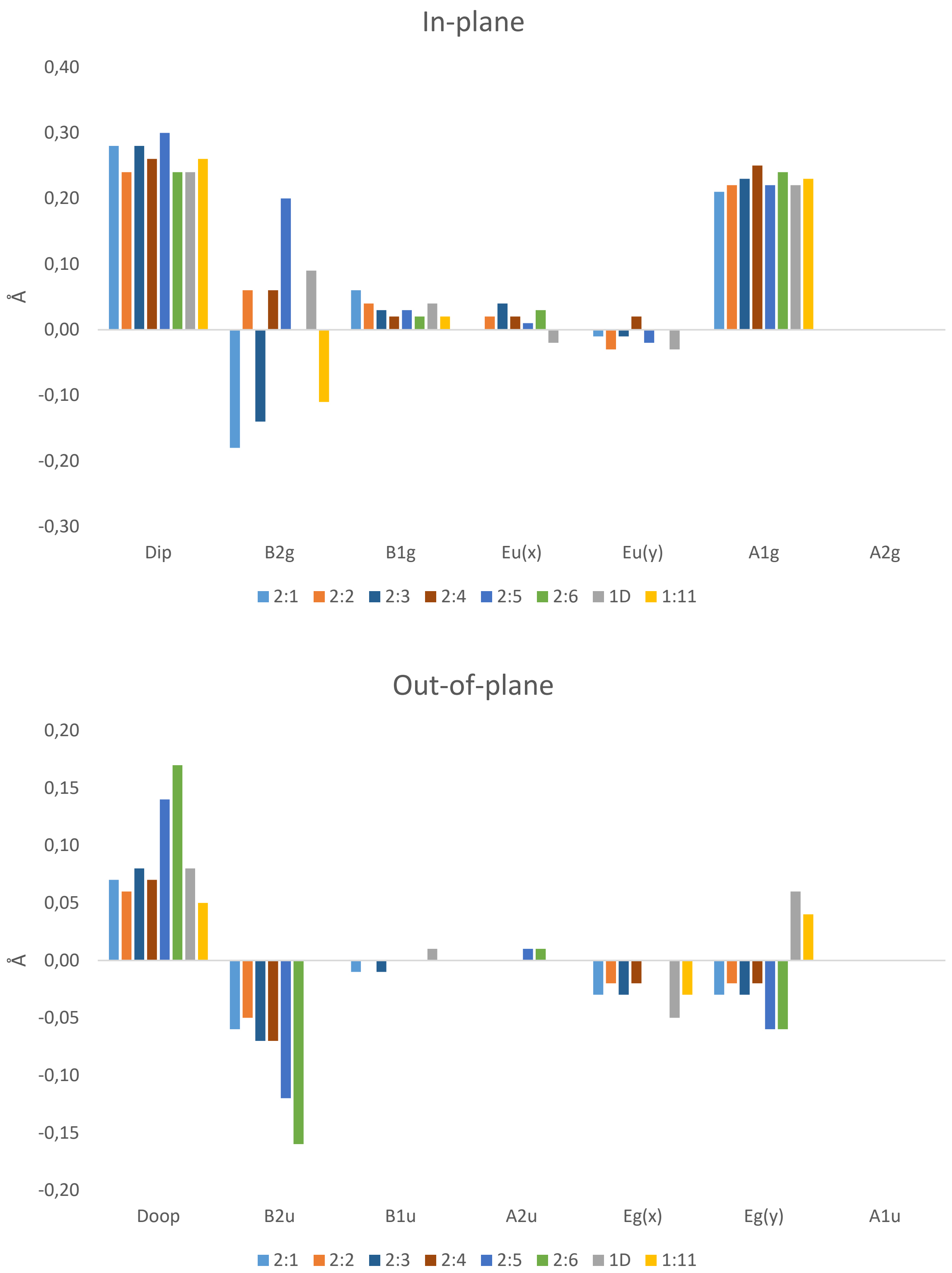

Figure S73: NSD charts for compounds **2:1-2:6**, **1D**, and **1:11**.

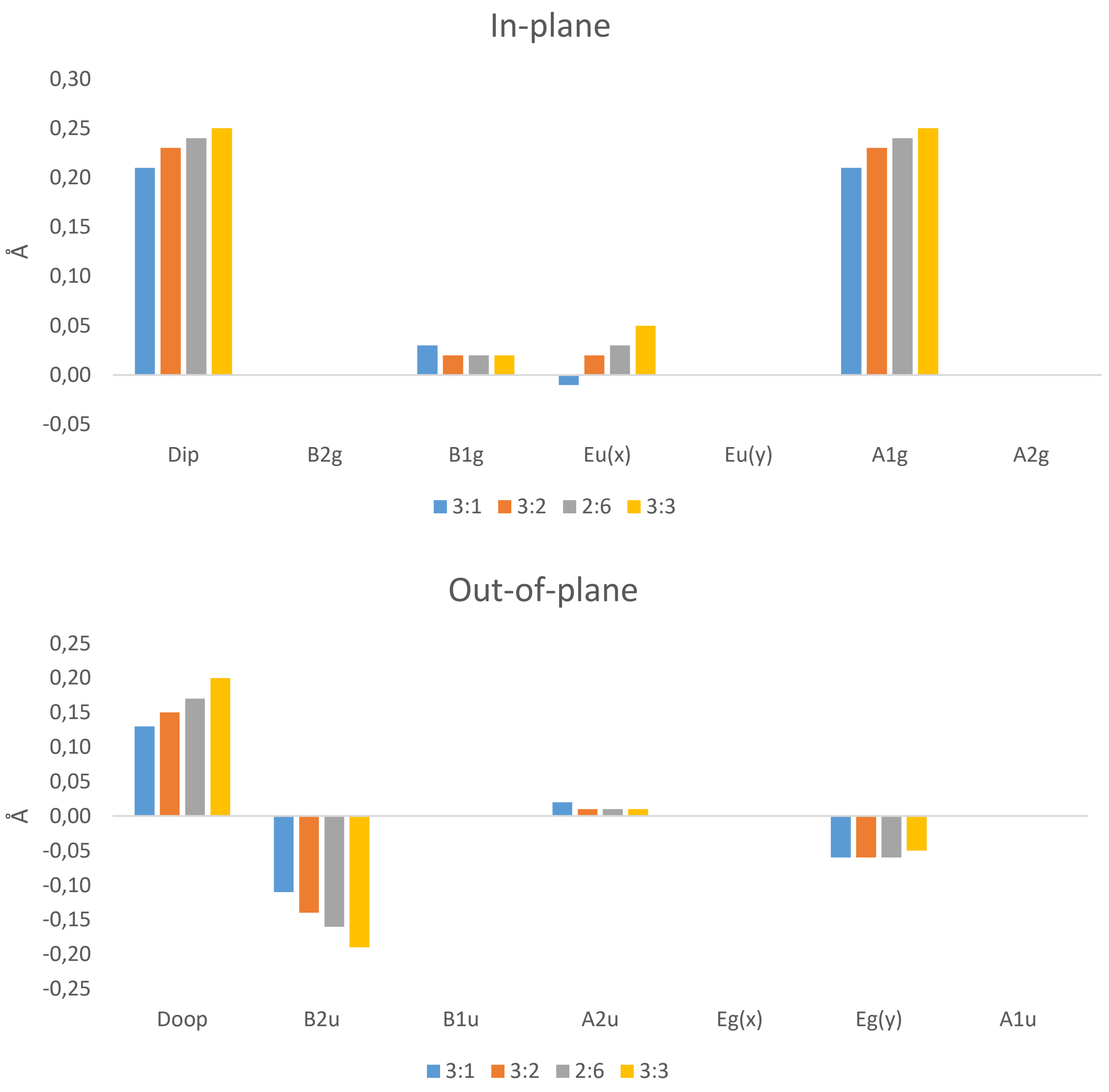

Figure S74: NSD charts for compounds **3:1**-**3:3** and **2:6**.

NSD tables and plots for crystal structures

basis	Δ_{ip}	δ_{ip}	B_{2g}	B_{1g}	$E_u(x)$	$E_u(y)$	A_{1g}	A_{2g}
min.	0.24	0.00	-0.12	0.02	0.03	-0.03	0.20	0.00
ext.	0.24	0.00	-0.12	0.02	0.03	-0.03	0.20	0.00
			0.00	-0.02	-0.01	0.02	0.00	0.00
total	0.25	0.00	-0.12	0.02	0.03	-0.03	0.20	0.00
			0.00	-0.02	-0.01	0.02	0.00	0.00
			-0.02	-0.03	0.00	0.00	0.06	0.00
			0.01	0.00	-0.01	0.01	-0.01	0.00
			0.00	0.00	0.00	0.00	-0.01	0.00
			-0.01	-0.01	0.00	0.00	0.02	
					0.00	0.00		
					-0.01	0.00		
					-0.01	0.00		
					0.00	0.00		
					0.00	0.00		
comp.	0.25	0.00	0.13	0.04	0.03	0.03	0.21	0.00

basis	Δ_{oop}	δ_{oop}	B_{2u}	B_{1u}	A_{2u}	$E_g(x)$	$E_g(y)$	A_{1u}
min.	0.15	0.00	-0.08	0.10	0.05	-0.01	0.06	0.02
ext.	0.17	0.00	-0.07	0.10	0.06	-0.02	0.06	0.02
			0.03	0.01	0.03	-0.02	-0.03	0.01
total	0.17	0.00	-0.07	0.10	0.06	-0.02	0.06	0.02
			0.03	0.01	0.03	-0.02	-0.03	0.01
			0.00	0.01	-0.01	0.00	0.00	
						0.00	0.00	
						-0.01	0.00	
comp.	0.17	0.00	0.08	0.10	0.07	0.03	0.07	0.02

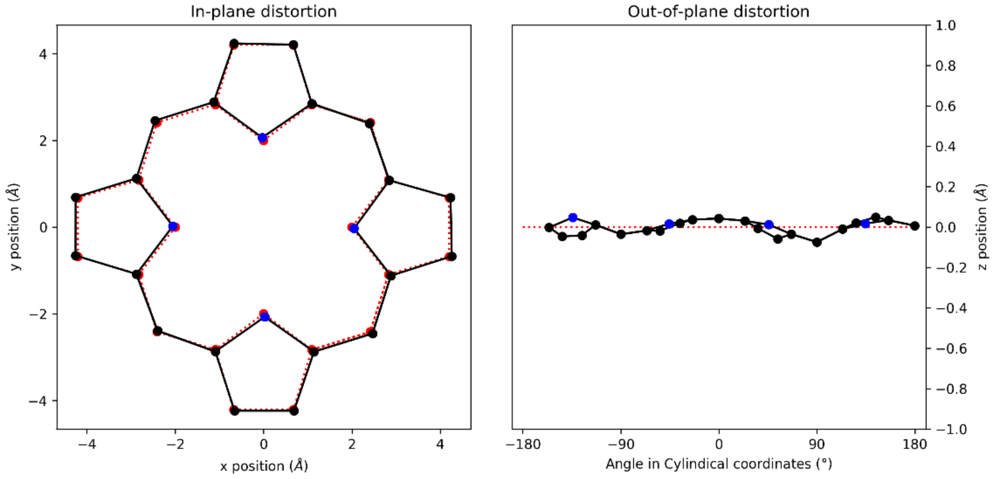

Figure S75: NSD result generated from **1** (in Å) (**A**) in-plane and (**B**) out-of-plane skeletal plots of the porphyrin core. Porphyrin is represented in black (C) and blue (N), with the reference structure (CuTPP) in red dotted lines.

basis	Δ_{ip}	δ_{ip}	B_{2g}	B_{1g}	$E_u(x)$	$E_u(y)$	A_{1g}	A_{2g}
min.	0.25	0.00	-0.06	0.02	-0.02	0.01	-0.24	-0.01
ext.	0.25	0.00	-0.06	0.02	-0.02	0.01	-0.24	-0.01
			0.03	0.01	0.02	-0.01	0.02	0.00
total	0.27	0.00	-0.06	0.02	-0.02	0.01	-0.24	-0.01
			0.03	0.01	0.02	-0.01	0.02	0.00
			-0.01	-0.01	0.01	-0.02	-0.01	0.00
			0.02	0.00	0.01	-0.02	-0.06	0.01
			0.00	0.02	-0.01	0.01	-0.03	0.02
			-0.02	0.03	-0.01	0.01	0.04	
					0.00	0.00		
					0.00	-0.01		
					0.01	0.00		
					0.01	0.02		
					0.00	-0.01		
comp.	0.27	0.00	0.07	0.04	0.04	0.04	0.25	0.02

basis	Δ_{oop}	δ_{oop}	B_{2u}	B_{1u}	A_{2u}	$E_g(x)$	$E_g(y)$	A_{1u}
min.	1.01	0.00	0.10	1.00	-0.06	0.03	0.06	0.00
ext.	1.01	0.00	0.10	1.00	-0.06	0.03	0.06	0.00
			-0.03	0.00	0.03	-0.05	-0.05	-0.01
total	1.01	0.00	0.10	1.00	-0.06	0.03	0.06	0.00
			-0.03	0.00	0.03	-0.05	-0.05	-0.01
			0.00	0.01	-0.01	0.01	-0.01	
						0.01	-0.01	
						0.00	0.00	
comp.	1.01	0.00	0.10	1.00	0.06	0.06	0.08	0.01

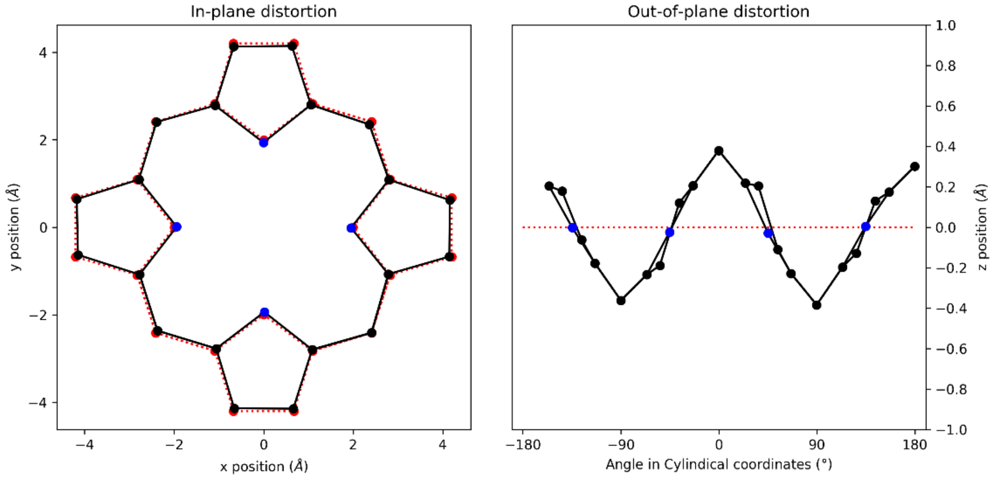

Figure S76: NSD result generated from **2** (in Å) (**A**) in-plane and (**B**) out-of-plane skeletal plots of the porphyrin core. Porphyrin is represented in black (C) and blue (N), with the reference structure (CuTPP) in red dotted lines.

basis	Δ_{ip}	δ_{ip}	B_{2g}	B_{1g}	$E_u(x)$	$E_u(y)$	A_{1g}	A_{2g}
min.	0.13	0.00	-0.06	0.02	0.02	-0.02	-0.11	-0.01
ext.	0.14	0.00	-0.06	0.02	0.02	-0.02	-0.12	-0.01
			-0.03	0.00	0.00	0.01	-0.04	0.00
total	0.15	0.00	-0.06	0.02	0.02	-0.02	-0.12	-0.01
			-0.03	0.00	0.00	0.01	-0.04	0.00
			-0.01	0.00	0.00	0.00	-0.03	0.00
			0.00	0.00	0.00	0.00	-0.01	0.00
			0.01	0.00	0.00	0.01	-0.01	0.00
			0.00	0.00	0.00	-0.01	0.00	
					0.00	0.00		
					0.00	0.00		
					0.01	0.00		
					-0.01	0.00		
					0.00	0.00		
comp.	0.15	0.00	0.06	0.02	0.02	0.02	0.13	0.01

basis	Δ_{oop}	δ_{oop}	B_{2u}	B_{1u}	A_{2u}	$E_g(x)$	$E_g(y)$	A_{1u}
min.	0.22	0.00	-0.06	0.20	0.04	0.01	0.07	0.01
ext.	0.23	0.00	-0.06	0.20	0.05	0.01	0.07	0.01
			0.01	0.00	0.03	-0.01	-0.03	0.00
total	0.23	0.00	-0.06	0.20	0.05	0.01	0.07	0.01
			0.01	0.00	0.03	-0.01	-0.03	0.00
			0.00	0.00	-0.01	0.00	0.01	
						-0.01	-0.01	
						0.00	0.00	
comp.	0.23	0.00	0.06	0.20	0.06	0.01	0.08	0.01

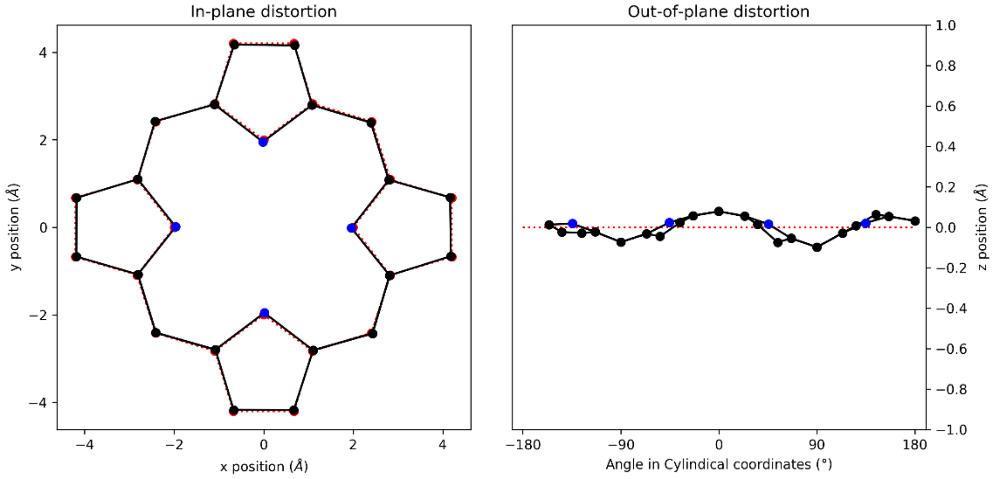

Figure S77: NSD result generated from **2A** (in Å) (**A**) in-plane and (**B**) out-of-plane skeletal plots of the porphyrin core. Porphyrin is represented in black (C) and blue (N), with the reference structure (CuTPP) in red dotted lines.

basis	Δ_{ip}	δ_{ip}	B_{2g}	B_{1g}	$E_u(x)$	$E_u(y)$	A_{1g}	A_{2g}
min.	0.29	0.00	-0.20	-0.03	-0.05	0.02	0.20	0.00
ext.	0.30	0.00	-0.20	-0.04	-0.05	0.02	0.20	0.00
			-0.04	-0.07	0.02	-0.04	-0.02	0.01
total	0.32	0.00	-0.20	-0.04	-0.05	0.02	0.20	0.00
			-0.04	-0.07	0.02	-0.04	-0.02	0.01
			-0.01	-0.06	0.00	0.00	0.05	0.00
			0.01	0.00	0.00	0.00	0.00	0.00
			0.00	0.01	0.00	0.01	0.01	0.00
			-0.01	-0.01	0.00	0.00	0.01	
					0.00	0.00		
					0.00	0.00		
					0.00	-0.01		
					0.00	0.00		
					0.00	-0.01		
comp.	0.32	0.00	0.20	0.10	0.05	0.05	0.21	0.01

basis	Δ_{oop}	δ_{oop}	B_{2u}	B_{1u}	A_{2u}	$E_g(x)$	$E_g(y)$	A_{1u}
min.	0.47	0.00	0.42	0.13	-0.06	-0.15	0.09	-0.02
ext.	0.48	0.00	0.41	0.13	-0.06	-0.15	0.09	-0.02
			-0.11	-0.01	0.00	-0.01	0.00	-0.01
total	0.48	0.00	0.41	0.13	-0.06	-0.15	0.09	-0.02
			-0.11	-0.01	0.00	-0.01	0.00	-0.01
			0.00	0.00	0.00	0.00	-0.01	
						0.00	0.00	
						-0.01	0.00	
comp.	0.48	0.00	0.43	0.13	0.06	0.15	0.09	0.02

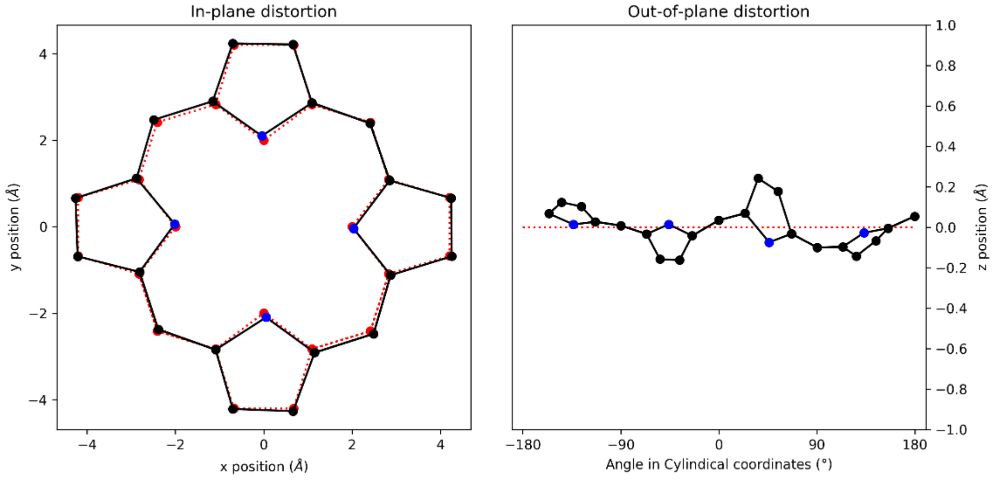

Figure S78: NSD result generated from **3** (in Å) (**A**) in-plane and (**B**) out-of-plane skeletal plots of the porphyrin core. Porphyrin is represented in black (C) and blue (N), with the reference structure (CuTPP) in red dotted lines.

basis	Δ_{ip}	δ_{ip}	B_{2g}	B_{1g}	$E_u(x)$	$E_u(y)$	A_{1g}	A_{2g}
min.	0.25	0.00	-0.10	-0.03	0.00	0.00	0.23	0.01
ext.	0.25	0.00	-0.10	-0.03	0.00	0.00	0.23	0.01
			-0.02	0.01	0.01	-0.02	-0.02	0.00
total	0.27	0.00	-0.10	-0.02	0.00	0.00	0.23	0.01
			-0.02	0.01	0.01	-0.01	-0.02	0.00
			-0.01	0.01	0.00	0.01	0.05	0.00
			0.00	0.00	-0.02	0.01	-0.01	0.00
			0.00	0.00	0.00	-0.01	0.02	0.00
			-0.01	0.00	-0.01	0.01	0.01	
					0.00	0.00		
					0.00	0.00		
					-0.01	0.01		
					0.01	-0.01		
					0.00	0.00		
comp.	0.27	0.00	0.10	0.03	0.03	0.03	0.24	0.01

basis	Δ_{oop}	δ_{oop}	B_{2u}	B_{1u}	A_{2u}	$E_g(x)$	$E_g(y)$	A_{1u}
min.	0.31	0.00	0.05	0.02	-0.05	0.11	0.28	-0.01
ext.	0.33	0.00	0.05	0.02	-0.05	0.11	0.28	-0.01
			-0.03	0.00	0.00	0.00	0.10	0.01
total	0.33	0.00	0.05	0.02	-0.05	0.11	0.28	-0.01
			-0.03	0.00	0.00	0.00	0.10	0.01
			0.00	0.00	0.01	-0.01	-0.02	
						-0.01	-0.01	
						0.00	0.01	
comp.	0.33	0.00	0.05	0.02	0.05	0.11	0.30	0.01

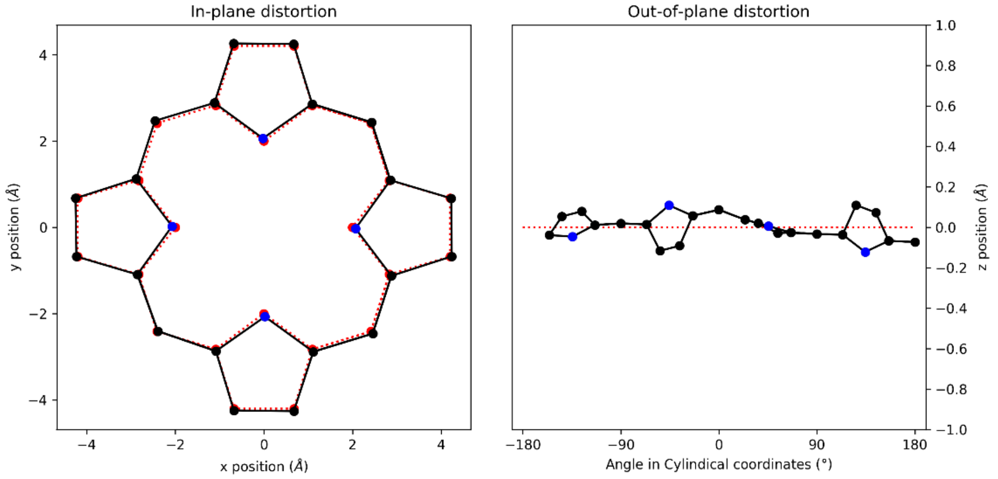

Figure S79: NSD result generated from **4** (in Å) (**A**) in-plane and (**B**) out-of-plane skeletal plots of the porphyrin core. Porphyrin is represented in black (C) and blue (N), with the reference structure (CuTPP) in red dotted lines.

basis	Δ_{ip}	δ_{ip}	B_{2g}	B_{1g}	$E_u(x)$	$E_u(y)$	A_{1g}	A_{2g}
min.	0.21	0.00	0.01	0.04	-0.01	-0.01	0.21	-0.01
ext.	0.22	0.00	0.01	0.04	-0.01	-0.01	0.21	-0.01
			0.01	0.03	0.01	0.01	-0.02	0.00
total	0.23	0.00	0.01	0.04	-0.01	-0.01	0.21	-0.01
			0.01	0.03	0.01	0.01	-0.02	0.00
			-0.01	0.02	0.01	0.00	0.05	0.00
			0.01	0.00	0.01	-0.02	-0.01	0.00
			0.00	-0.01	0.00	0.00	0.01	0.00
			-0.01	0.00	0.01	0.00	0.01	
					0.00	0.00		
					0.00	0.00		
					0.00	-0.01		
					0.00	0.00		
					0.00	0.00		
comp.	0.23	0.00	0.03	0.06	0.02	0.02	0.22	0.01

basis	Δ_{oop}	δ_{oop}	B_{2u}	B_{1u}	A_{2u}	$E_g(x)$	$E_g(y)$	A_{1u}
min.	0.39	0.00	0.10	0.31	-0.10	-0.05	0.20	-0.01
ext.	0.41	0.00	0.10	0.31	-0.10	-0.05	0.20	-0.01
			0.03	0.00	-0.04	0.01	0.09	-0.01
total	0.41	0.00	0.10	0.31	-0.10	-0.05	0.20	-0.01
			0.03	0.00	-0.04	0.01	0.09	-0.01
			0.00	0.00	0.01	0.02	0.01	
						0.00	-0.01	
						-0.01	0.01	
comp.	0.41	0.00	0.11	0.31	0.11	0.05	0.22	0.01

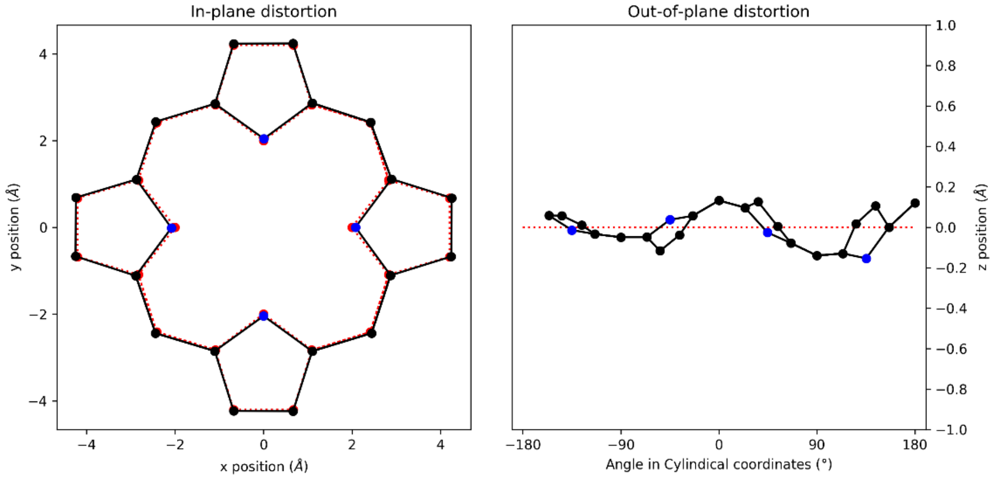

Figure S80: NSD result generated from **5** (in Å) (**A**) in-plane and (**B**) out-of-plane skeletal plots of the porphyrin core. Porphyrin is represented in black (C) and blue (N), with the reference structure (CuTPP) in red dotted lines.

basis	Δ_{ip}	δ_{ip}	B_{2g}	B_{1g}	$E_u(x)$	$E_u(y)$	A_{1g}	A_{2g}
min.	0.22	0.00	0.00	0.01	0.00	0.00	0.22	0.00
ext.	0.23	0.00	0.00	0.01	0.01	0.00	0.22	0.00
			-0.01	0.01	0.03	0.02	-0.03	0.00
total	0.25	0.00	0.00	0.01	0.00	0.00	0.23	0.00
			-0.01	0.01	0.03	0.02	-0.03	0.00
			0.01	0.02	-0.01	0.01	0.06	0.00
			-0.01	0.00	-0.01	-0.02	-0.02	-0.01
			-0.01	0.00	0.00	0.00	0.01	0.00
			0.00	0.01	0.00	-0.02	0.03	
					0.00	0.00		
					0.00	-0.01		
					-0.01	0.01		
					0.00	0.00		
					0.01	-0.01		
comp.	0.25	0.00	0.03	0.03	0.03	0.04	0.24	0.01

basis	Δ_{oop}	δ_{oop}	B_{2u}	B_{1u}	A_{2u}	$E_g(x)$	$E_g(y)$	A_{1u}
min.	0.36	0.00	-0.22	-0.08	-0.02	-0.27	-0.06	-0.01
ext.	0.36	0.00	-0.22	-0.08	-0.02	-0.27	-0.06	-0.01
			0.01	0.01	0.00	0.03	0.01	0.00
total	0.36	0.00	-0.22	-0.08	-0.02	-0.27	-0.06	-0.01
			0.02	0.01	0.00	0.03	0.01	0.00
			0.00	0.00	0.00	0.01	0.01	
						0.00	0.00	
						-0.01	0.01	
comp.	0.36	0.00	0.22	0.08	0.02	0.27	0.06	0.01

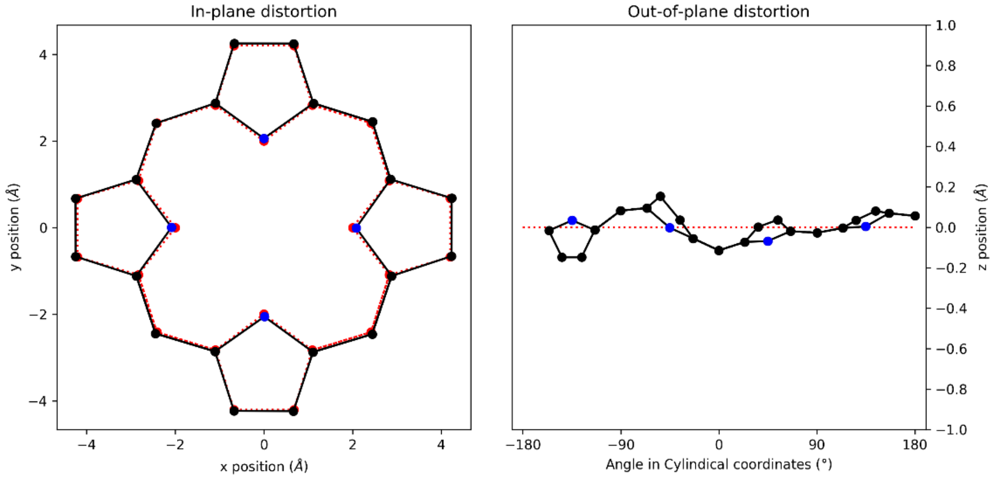

Figure S81: NSD result generated from **6** (in Å) (**A**) in-plane and (**B**) out-of-plane skeletal plots of the porphyrin core. Porphyrin is represented in black (C) and blue (N), with the reference structure (CuTPP) in red dotted lines.

basis	Δ_{ip}	δ_{ip}	B_{2g}	B_{1g}	$E_u(x)$	$E_u(y)$	A_{1g}	A_{2g}
min.	0.14	0.00	0.05	-0.01	0.02	0.02	-0.12	0.00
ext.	0.15	0.00	0.05	-0.01	0.02	0.02	-0.12	0.00
			0.00	0.00	0.03	0.01	-0.05	0.00
total	0.16	0.00	0.05	-0.01	0.02	0.02	-0.13	0.00
			0.00	0.00	0.03	0.01	-0.05	0.00
			0.01	0.00	-0.01	-0.01	-0.04	0.00
			-0.01	0.00	-0.02	-0.02	-0.02	0.00
			0.00	0.00	0.00	0.00	-0.01	0.00
			0.00	0.00	-0.01	-0.01	0.01	
					0.00	0.00		
					0.00	0.00		
					-0.01	0.00		
					0.01	0.01		
					0.01	0.00		
comp.	0.16	0.00	0.05	0.01	0.04	0.03	0.14	0.01

basis	Δ_{oop}	δ_{oop}	B_{2u}	B_{1u}	A_{2u}	$E_g(x)$	$E_g(y)$	A_{1u}
min.	0.36	0.00	0.26	0.08	-0.01	0.24	0.00	0.01
ext.	0.37	0.00	0.26	0.08	0.00	0.23	0.00	0.01
			0.00	0.00	0.03	-0.09	-0.02	0.00
total	0.37	0.00	0.26	0.08	0.00	0.23	0.00	0.01
			0.00	0.00	0.03	-0.09	-0.02	0.00
			0.00	0.00	0.00	-0.01	0.00	
						0.00	0.00	
						0.01	0.00	
comp.	0.37	0.00	0.26	0.08	0.03	0.25	0.02	0.01

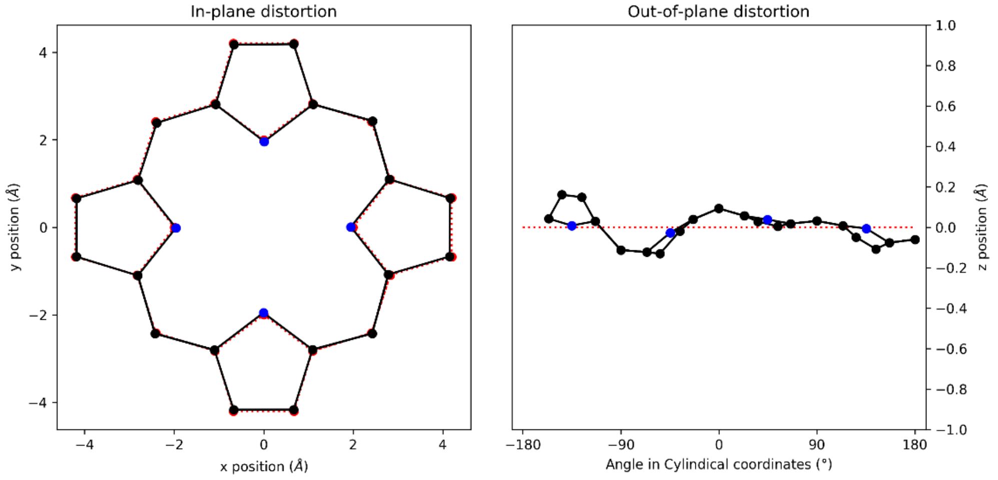

Figure S82: NSD result generated from **7** (in Å) (**A**) in-plane and (**B**) out-of-plane skeletal plots of the porphyrin core. Porphyrin is represented in black (C) and blue (N), with the reference structure (CuTPP) in red dotted lines.

basis	Δ_{ip}	δ_{ip}	B_{2g}	B_{1g}	$E_u(x)$	$E_u(y)$	A_{1g}	A_{2g}
min.	0.23	0.00	-0.04	0.00	-0.01	0.02	0.23	0.00
ext.	0.24	0.00	-0.04	0.00	-0.01	0.02	0.23	0.00
			-0.03	-0.03	0.02	0.01	-0.04	-0.01
total	0.26	0.00	-0.04	0.00	-0.01	0.02	0.23	0.00
			-0.03	-0.03	0.02	0.01	-0.04	-0.01
			0.02	-0.02	0.00	0.00	0.06	0.00
			-0.02	0.00	-0.02	-0.01	-0.02	0.00
			-0.01	0.00	0.00	0.00	0.01	0.00
			0.01	0.00	-0.01	-0.01	0.02	
					-0.01	0.01		
					0.00	0.00		
					-0.01	-0.01		
					0.01	0.00		
					0.00	0.00		
comp.	0.26	0.00	0.06	0.04	0.03	0.03	0.24	0.01

basis	Δ_{oop}	δ_{oop}	B_{2u}	B_{1u}	A_{2u}	$E_g(x)$	$E_g(y)$	A_{1u}
min.	0.34	0.00	-0.16	0.10	0.05	-0.07	-0.27	0.00
ext.	0.34	0.00	-0.16	0.10	0.05	-0.07	-0.27	0.00
			0.01	-0.01	0.00	0.01	0.03	0.00
total	0.34	0.00	-0.16	0.10	0.05	-0.07	-0.27	0.00
			0.01	-0.01	0.00	0.01	0.03	0.00
			0.01	0.00	0.00	0.01	0.02	
						0.00	0.00	
						0.01	0.00	
comp.	0.34	0.00	0.16	0.10	0.05	0.07	0.28	0.00

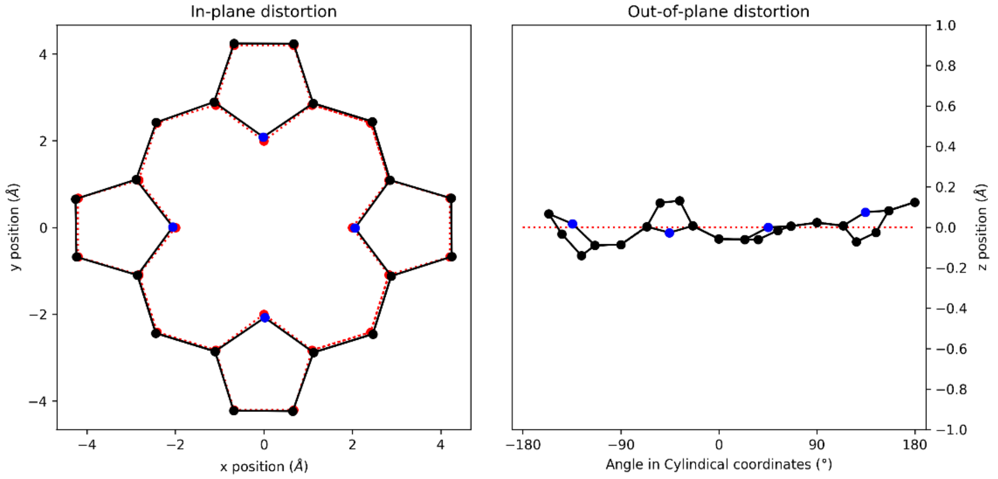

Figure S83: NSD result generated from 8_1 (in Å) (A) in-plane and (B) out-of-plane skeletal plots of the porphyrin core. Porphyrin is represented in black (C) and blue (N), with the reference structure (CuTPP) in red dotted lines.

basis	Δ_{ip}	δ_{ip}	B_{2g}	B_{1g}	$E_u(x)$	$E_u(y)$	A_{1g}	A_{2g}
min.	0.22	0.00	-0.06	-0.01	-0.01	-0.01	0.21	-0.01
ext.	0.23	0.00	-0.06	-0.01	-0.01	-0.01	0.21	-0.01
			-0.03	-0.02	-0.03	-0.02	-0.04	0.00
total	0.25	0.00	-0.06	-0.01	-0.01	-0.01	0.22	-0.01
			-0.03	-0.02	-0.03	-0.02	-0.04	0.00
			0.01	-0.02	0.00	0.01	0.06	0.00
			-0.02	0.00	0.02	0.01	-0.01	-0.01
			0.00	0.01	0.00	0.00	0.00	0.01
			0.01	0.00	0.00	0.00	0.01	
					0.00	0.00		
					0.00	0.00		
					0.01	0.01		
					0.00	0.00		
					0.00	0.01		
comp.	0.25	0.00	0.07	0.03	0.04	0.03	0.23	0.02

basis	Δ_{oop}	δ_{oop}	B_{2u}	B_{1u}	A_{2u}	$E_g(x)$	$E_g(y)$	A_{1u}
min.	0.37	0.00	0.20	-0.05	-0.02	-0.06	-0.30	0.00
ext.	0.37	0.00	0.20	-0.05	-0.02	-0.06	-0.30	0.00
			-0.01	0.00	0.00	0.03	0.04	0.01
total	0.37	0.00	0.20	-0.05	-0.02	-0.06	-0.30	0.00
			-0.01	0.00	0.00	0.03	0.04	0.01
			0.00	-0.01	0.00	-0.01	0.02	
						0.00	0.00	
						0.00	-0.01	
comp.	0.37	0.00	0.20	0.05	0.02	0.06	0.30	0.01

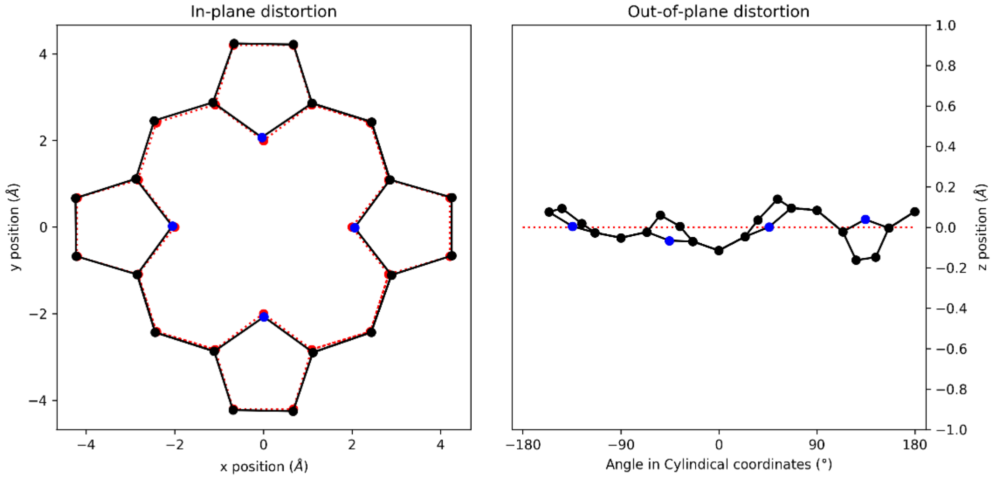

Figure S84: NSD result generated from **8_2** (in Å) (**A**) in-plane and (**B**) out-of-plane skeletal plots of the porphyrin core. Porphyrin is represented in black (C) and blue (N), with the reference structure (CuTPP) in red dotted lines.

basis	Δ_{ip}	δ_{ip}	B_{2g}	B_{1g}	$E_u(x)$	$E_u(y)$	A_{1g}	A_{2g}
min.	0.25	0.00	0.00	-0.06	0.00	0.00	0.24	-0.01
ext.	0.25	0.00	0.00	-0.06	0.00	0.00	0.24	-0.01
			-0.02	0.03	0.00	0.00	0.00	0.00
total	0.26	0.00	0.00	-0.06	0.00	0.00	0.24	-0.01
			-0.02	0.03	0.00	0.00	0.00	0.00
			0.03	0.03	0.00	0.00	0.05	0.00
			-0.02	0.00	0.00	0.00	0.00	0.00
			0.00	-0.01	0.00	0.00	-0.01	0.00
			0.01	0.00	0.00	0.00	0.01	
					0.00	0.00		
					0.00	0.00		
					0.00	0.00		
					0.00	0.00		
					0.00	0.00		
comp.	0.26	0.00	0.04	0.08	0.00	0.00	0.25	0.01

basis	Δ_{oop}	δ_{oop}	B_{2u}	B_{1u}	A_{2u}	$E_g(x)$	$E_g(y)$	A_{1u}
min.	0.08	0.00	0.00	0.00	0.00	-0.01	0.08	0.00
ext.	0.11	0.00	0.00	0.00	0.00	-0.01	0.08	0.00
			0.00	0.00	0.00	0.06	0.02	0.00
total	0.11	0.00	0.00	0.00	0.00	-0.01	0.08	0.00
			0.00	0.00	0.00	0.06	0.02	0.00
			0.00	0.00	0.00	0.01	-0.01	
						0.00	0.00	
						0.01	0.00	
comp.	0.11	0.00	0.00	0.00	0.00	0.07	0.08	0.00

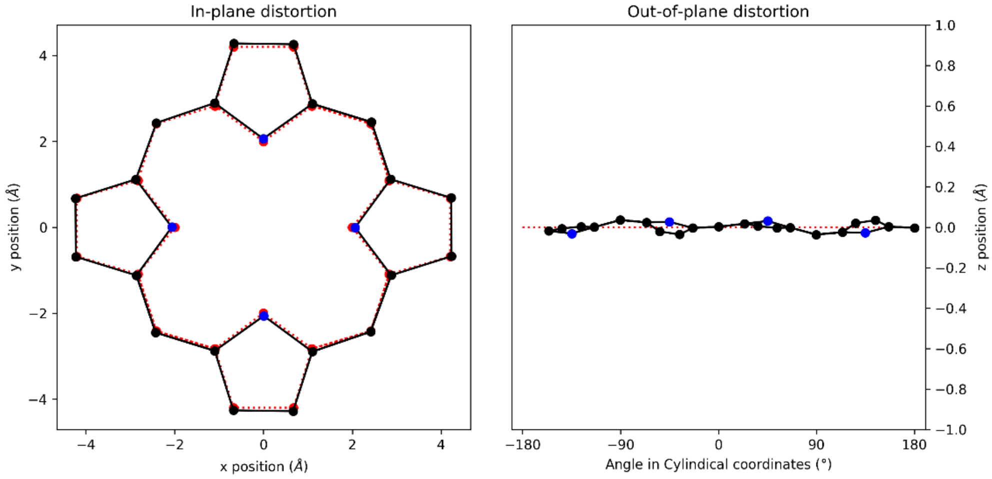

Figure S85: NSD result generated from **9** (in Å) (**A**) in-plane and (**B**) out-of-plane skeletal plots of the porphyrin core. Porphyrin is represented in black (C) and blue (N), with the reference structure (CuTPP) in red dotted lines.

basis	Δ_{ip}	δ_{ip}	B_{2g}	B_{1g}	$E_u(x)$	$E_u(y)$	A_{1g}	A_{2g}
min.	0.24	0.00	-0.06	-0.02	0.00	0.00	0.23	-0.01
ext.	0.24	0.00	-0.06	-0.02	0.00	0.00	0.23	-0.01
			-0.01	0.04	0.00	0.00	0.00	-0.01
total	0.26	0.00	-0.06	-0.02	0.00	0.00	0.23	-0.01
			-0.01	0.04	0.00	0.00	0.00	-0.01
			-0.03	0.04	0.00	0.00	0.05	0.00
			0.01	0.00	0.00	0.00	-0.01	0.00
			0.00	-0.01	0.00	0.00	0.00	0.00
			-0.01	0.01	0.00	0.00	0.01	
					0.00	0.00		
					0.00	0.00		
					0.00	0.00		
					0.00	0.00		
					0.00	0.00		
comp.	0.26	0.00	0.07	0.06	0.00	0.00	0.24	0.01

basis	Δ_{oop}	δ_{oop}	B_{2u}	B_{1u}	A_{2u}	$E_g(x)$	$E_g(y)$	A_{1u}
min.	0.24	0.00	0.00	0.00	0.00	0.03	0.23	0.00
ext.	0.24	0.00	0.00	0.00	0.00	0.03	0.23	0.00
			0.00	0.00	0.00	0.04	0.00	0.00
total	0.24	0.00	0.00	0.00	0.00	0.03	0.23	0.00
			0.00	0.00	0.00	0.04	0.00	0.00
			0.00	0.00	0.00	0.02	0.01	
						0.00	-0.02	
						-0.01	0.01	
comp.	0.24	0.00	0.00	0.00	0.00	0.06	0.24	0.00

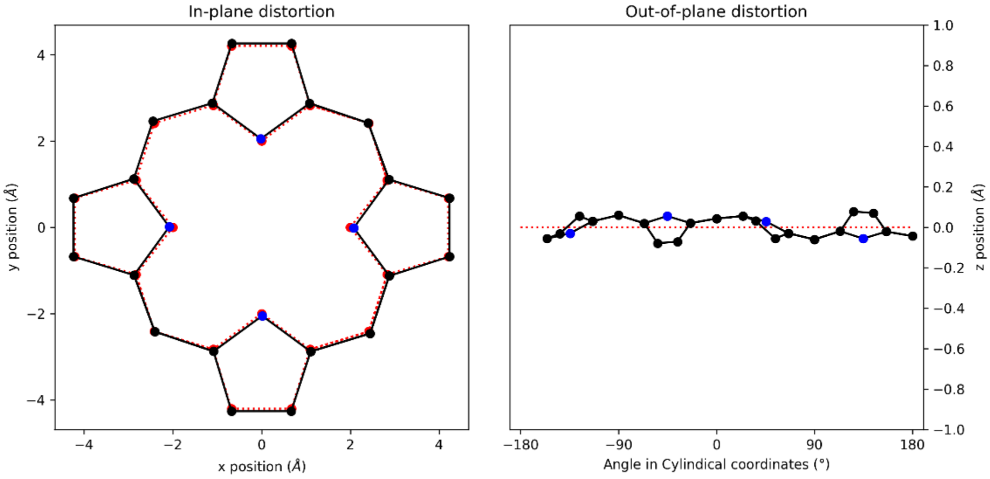

Figure S86: NSD result generated from **10** (in Å) (**A**) in-plane and (**B**) out-of-plane skeletal plots of the porphyrin core. Porphyrin is represented in black (C) and blue (N), with the reference structure (CuTPP) in red dotted lines.

basis	Δ_{ip}	δ_{ip}	B_{2g}	B_{1g}	$E_u(x)$	$E_u(y)$	A_{1g}	A_{2g}
min.	0.49	0.01	-0.14	0.01	0.03	0.02	-0.47	-0.01
ext.	0.53	0.01	-0.14	0.01	0.03	0.02	-0.47	-0.01
			0.05	0.01	-0.01	-0.01	0.16	0.07
total	0.54	0.00	-0.14	0.01	0.03	0.02	-0.47	-0.01
			0.05	0.01	-0.01	-0.01	0.16	0.07
			-0.01	-0.01	0.00	0.01	0.02	-0.01
			0.04	0.01	0.02	0.01	-0.11	0.00
			0.00	0.00	0.00	0.00	-0.03	0.02
			0.00	0.01	0.00	0.00	0.07	
					0.01	0.00		
					0.00	0.00		
					-0.02	0.00		
					0.00	-0.01		
					0.00	0.00		
comp.	0.54	0.00	0.15	0.02	0.04	0.03	0.51	0.08

basis	Δ_{oop}	δ_{oop}	B_{2u}	B_{1u}	A_{2u}	$E_g(x)$	$E_g(y)$	A_{1u}
min.	1.90	0.00	0.55	1.81	-0.19	0.07	-0.05	0.00
ext.	1.90	0.00	0.55	1.81	-0.19	0.07	-0.05	0.00
			0.00	0.02	0.06	-0.03	0.00	0.00
total	1.90	0.00	0.54	1.81	-0.19	0.07	-0.05	0.00
			0.00	0.02	0.06	-0.03	0.00	0.00
			-0.01	0.02	-0.01	0.00	0.01	
						0.00	0.00	
						0.00	0.00	
comp.	1.90	0.00	0.55	1.81	0.20	0.07	0.05	0.00

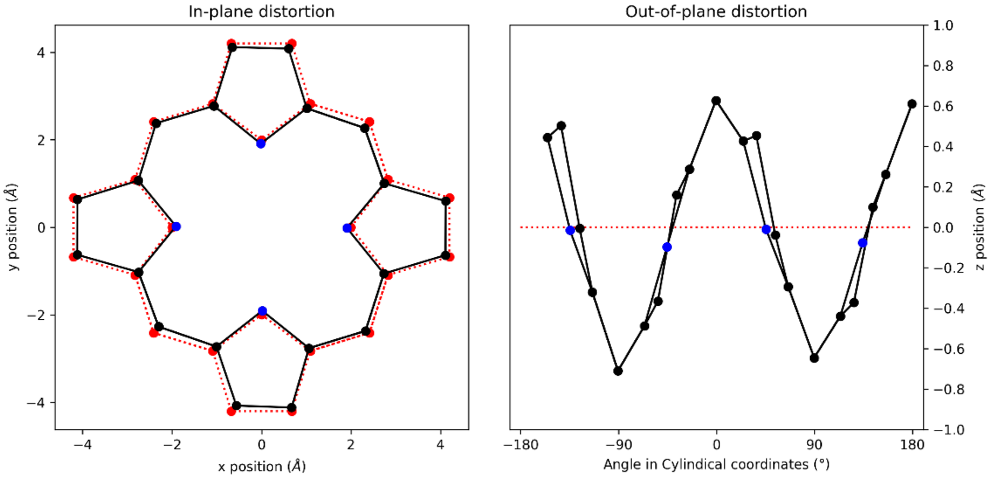

Figure S87: NSD result generated from **11** (in Å) (**A**) in-plane and (**B**) out-of-plane skeletal plots of the porphyrin core. Porphyrin is represented in black (C) and blue (N), with the reference structure (CuTPP) in red dotted lines.

basis	Δ_{ip}	δ_{ip}	B_{2g}	B_{1g}	$E_u(x)$	$E_u(y)$	A_{1g}	A_{2g}
min.	0.19	0.01	0.02	-0.02	-0.01	-0.01	0.18	-0.01
ext.	0.21	0.01	0.02	-0.02	-0.01	-0.01	0.18	-0.01
			-0.07	-0.05	-0.01	0.00	0.01	0.01
total	0.26	0.00	0.02	-0.02	-0.01	-0.01	0.18	-0.01
			-0.07	-0.05	-0.01	0.00	0.01	0.01
			0.05	0.03	0.01	-0.01	0.03	0.00
			0.02	0.02	-0.01	-0.01	-0.09	0.00
			0.00	0.05	0.01	-0.01	0.01	-0.01
			-0.02	0.02	-0.01	-0.01	-0.02	
					0.02	-0.01		
					0.02	-0.02		
					-0.05	-0.03		
					0.02	-0.01		
					0.02	-0.01		
comp.	0.26	0.00	0.09	0.08	0.07	0.05	0.21	0.01

basis	Δ_{oop}	δ_{oop}	B_{2u}	B_{1u}	A_{2u}	$E_g(x)$	$E_g(y)$	A_{1u}
min.	0.17	0.00	0.08	-0.02	-0.02	-0.15	-0.01	0.00
ext.	0.19	0.00	0.08	-0.02	-0.02	-0.15	-0.01	0.00
			-0.04	0.02	0.01	0.05	-0.03	0.02
total	0.20	0.00	0.08	-0.02	-0.02	-0.15	-0.01	0.00
			-0.04	0.02	0.01	0.05	-0.03	0.02
			-0.01	0.04	0.00	-0.03	0.00	
						0.02	0.01	
						-0.03	0.00	
comp.	0.20	0.00	0.09	0.04	0.03	0.17	0.04	0.02

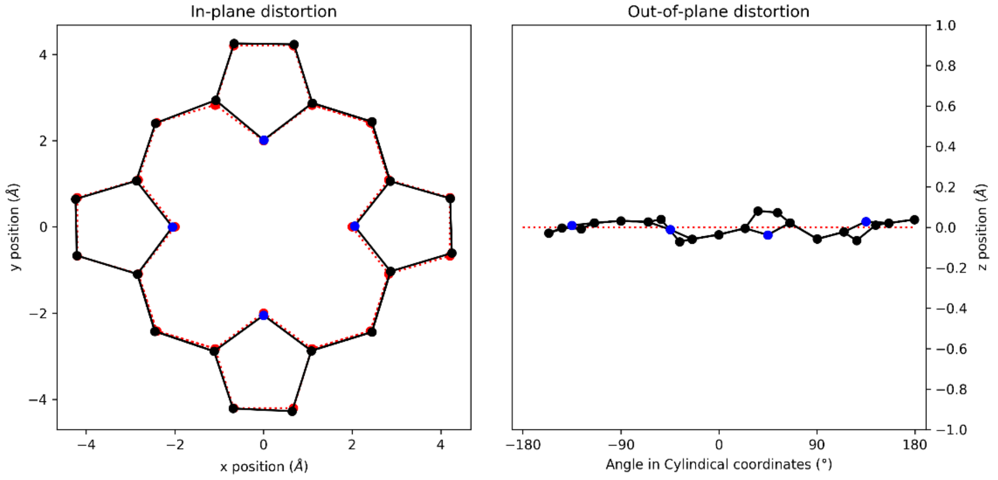

Figure S88: NSD result generated from **12** (in Å) (**A**) in-plane and (**B**) out-of-plane skeletal plots of the porphyrin core. Porphyrin is represented in black (C) and blue (N), with the reference structure (CuTPP) in red dotted lines.

basis	Δ_{ip}	δ_{ip}	B_{2g}	B_{1g}	$E_u(x)$	$E_u(y)$	A_{1g}	A_{2g}
min.	0.19	0.00	0.08	0.02	-0.01	-0.02	0.17	0.00
ext.	0.20	0.00	0.08	0.02	-0.01	-0.02	0.17	0.00
			-0.01	0.02	-0.02	0.01	-0.01	-0.02
total	0.21	0.00	0.08	0.02	-0.01	-0.02	0.18	0.00
			-0.01	0.02	-0.02	0.01	-0.01	-0.02
			0.03	0.01	0.00	0.00	0.06	0.00
			-0.02	0.00	0.01	-0.01	-0.01	0.00
			0.00	0.00	0.01	0.00	0.00	0.00
			0.01	0.00	0.00	0.00	0.01	
					0.00	0.00		
					0.00	0.00		
					0.00	0.00		
					0.00	0.00		
					0.00	0.00		
comp.	0.21	0.00	0.09	0.03	0.02	0.02	0.19	0.02

basis	Δ_{oop}	δ_{oop}	B_{2u}	B_{1u}	A_{2u}	$E_g(x)$	$E_g(y)$	A_{1u}
min.	0.90	0.00	0.86	-0.24	-0.03	0.02	-0.10	0.00
ext.	0.90	0.00	0.86	-0.24	-0.03	0.02	-0.10	0.00
			-0.03	0.00	-0.01	0.00	0.01	0.00
total	0.90	0.00	0.86	-0.24	-0.03	0.02	-0.10	0.00
			-0.03	0.00	-0.01	0.00	0.01	0.00
			-0.01	0.00	0.00	0.01	-0.01	
						0.00	0.00	
						0.00	0.00	
comp.	0.90	0.00	0.86	0.24	0.03	0.02	0.10	0.00

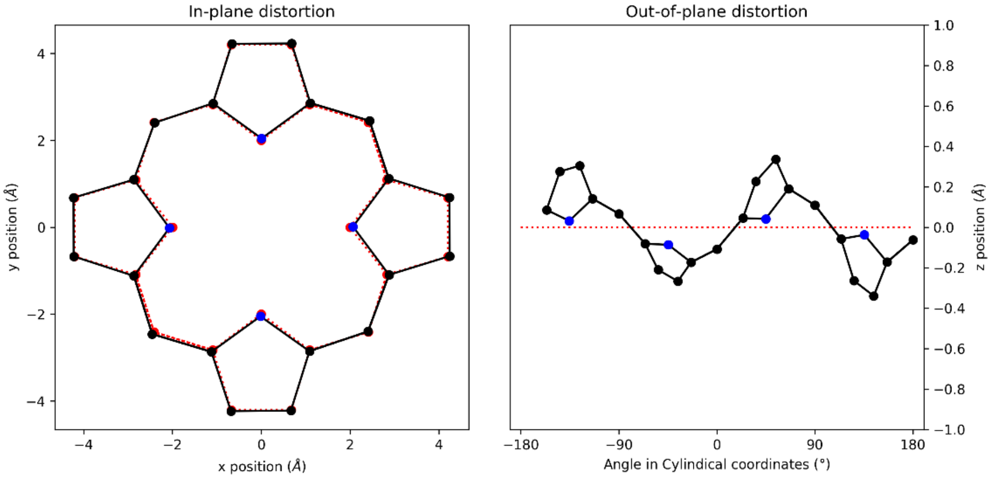

Figure S89: NSD result generated from **13** (in Å) (**A**) in-plane and (**B**) out-of-plane skeletal plots of the porphyrin core. Porphyrin is represented in black (C) and blue (N), with the reference structure (CuTPP) in red dotted lines.

basis	Δ_{ip}	δ_{ip}	B_{2g}	B_{1g}	$E_u(x)$	$E_u(y)$	A_{1g}	A_{2g}
min.	0.19	0.00	-0.08	-0.02	-0.02	0.01	0.17	0.00
ext.	0.20	0.00	-0.08	-0.02	-0.02	0.01	0.17	0.00
			0.01	-0.02	0.01	0.02	-0.01	-0.02
total	0.21	0.00	-0.08	-0.02	-0.02	0.01	0.18	0.00
			0.01	-0.02	0.01	0.02	-0.01	-0.02
			-0.03	-0.02	0.00	0.00	0.06	0.00
			0.02	0.00	0.00	-0.01	0.00	0.00
			0.00	0.00	0.00	-0.01	0.00	0.00
			-0.01	0.00	0.00	0.00	0.01	
					0.00	0.00		
					0.00	0.00		
					0.00	0.00		
					0.00	0.00		
					0.00	0.00		
comp.	0.21	0.00	0.09	0.03	0.02	0.02	0.19	0.02

basis	Δ_{oop}	δ_{oop}	B_{2u}	B_{1u}	A_{2u}	$E_g(x)$	$E_g(y)$	A_{1u}
min.	0.89	0.00	0.85	-0.24	0.03	0.10	0.02	0.00
ext.	0.89	0.00	0.85	-0.24	0.03	0.10	0.02	0.00
			-0.03	0.00	0.01	-0.01	0.00	0.00
total	0.89	0.00	0.85	-0.24	0.03	0.10	0.02	0.00
			-0.03	0.00	0.01	-0.01	0.00	0.00
			-0.01	0.00	0.00	0.01	0.01	
						0.00	0.00	
						0.00	0.00	
comp.	0.89	0.00	0.85	0.24	0.03	0.10	0.02	0.00

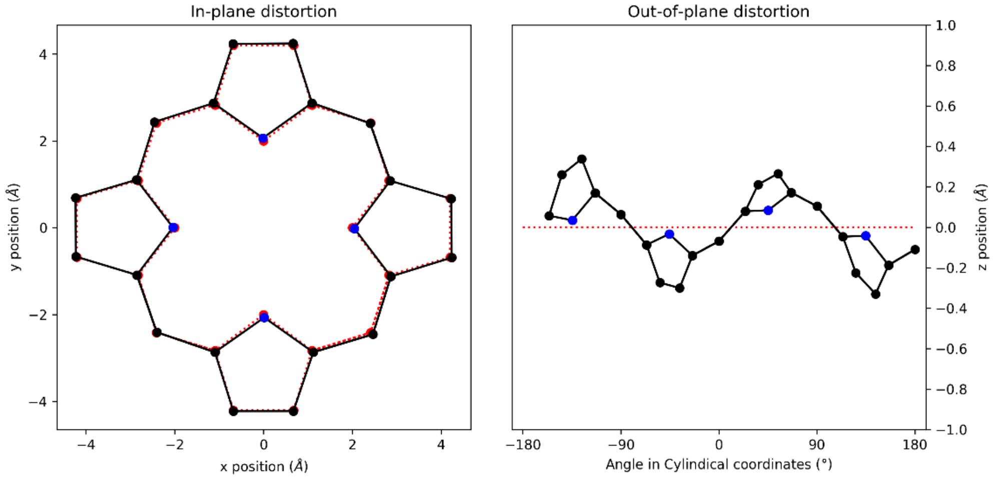

Figure S90: NSD result generated from **13A** (in Å) (**A**) in-plane and (**B**) out-of-plane skeletal plots of the porphyrin core. Porphyrin is represented in black (C) and blue (N), with the reference structure (CuTPP) in red dotted lines.

basis	Δ_{ip}	δ_{ip}	B_{2g}	B_{1g}	$E_u(x)$	$E_u(y)$	A_{1g}	A_{2g}
min.	0.22	0.00	-0.05	-0.04	0.01	0.01	0.21	0.00
ext.	0.22	0.00	-0.05	-0.04	0.01	0.01	0.21	0.00
			0.01	-0.02	-0.01	0.00	-0.01	0.00
total	0.23	0.00	-0.05	-0.04	0.01	0.01	0.21	0.00
			0.01	-0.02	-0.01	0.00	-0.01	0.00
			-0.03	-0.02	0.00	0.00	0.06	0.00
			0.02	-0.01	0.00	0.00	-0.01	0.00
			0.01	0.00	0.00	-0.01	0.00	-0.01
			-0.01	0.00	0.00	0.00	0.02	
					0.00	0.00		
					0.00	0.01		
					0.00	0.00		
					0.00	0.00		
					0.00	0.00		
comp.	0.23	0.00	0.06	0.05	0.01	0.02	0.22	0.01

basis	Δ_{oop}	δ_{oop}	B_{2u}	B_{1u}	A_{2u}	$E_g(x)$	$E_g(y)$	A_{1u}
min.	0.36	0.00	0.22	0.01	-0.06	0.26	0.11	-0.02
ext.	0.37	0.00	0.22	0.01	-0.06	0.25	0.11	-0.02
			-0.04	0.00	-0.05	-0.05	0.03	0.00
total	0.37	0.00	0.22	0.01	-0.06	0.25	0.11	-0.02
			-0.04	0.00	-0.05	-0.05	0.03	0.00
			0.00	-0.01	0.00	-0.03	-0.01	
						0.00	0.00	
						0.01	0.00	
comp.	0.37	0.00	0.22	0.01	0.08	0.26	0.11	0.02

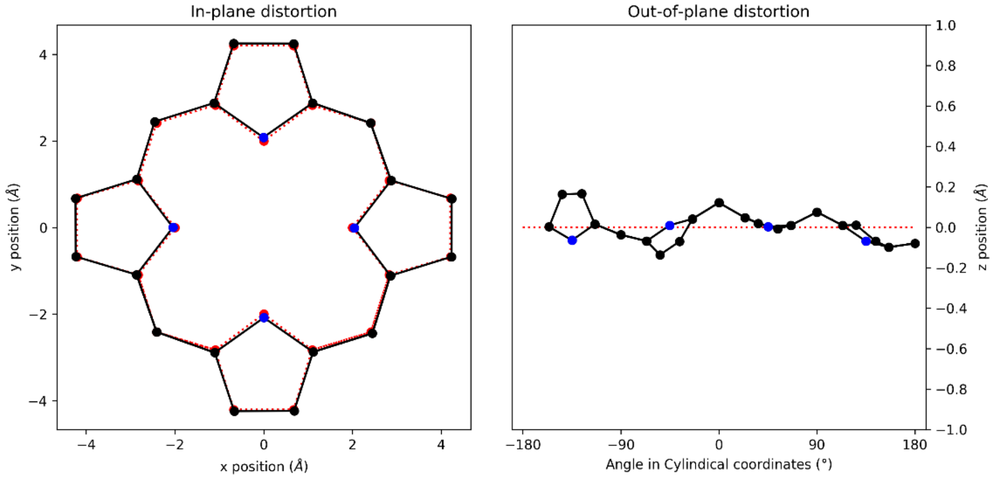

Figure S91: NSD result generated from **14** (in Å) (**A**) in-plane and (**B**) out-of-plane skeletal plots of the porphyrin core. Porphyrin is represented in black (C) and blue (N), with the reference structure (CuTPP) in red dotted lines.

basis	Δ_{ip}	δ_{ip}	B_{2g}	B_{1g}	$E_u(x)$	$E_u(y)$	A_{1g}	A_{2g}
min.	0.21	0.00	-0.02	-0.01	0.00	0.01	0.21	0.00
ext.	0.21	0.00	-0.02	-0.01	0.00	0.01	0.21	0.00
			0.02	0.00	0.00	0.01	0.02	0.00
total	0.22	0.00	-0.02	-0.01	0.00	0.01	0.21	0.00
			0.02	0.00	0.00	0.01	0.02	0.00
			-0.03	0.00	0.00	0.00	0.02	0.00
			0.02	0.00	0.00	0.00	-0.01	0.00
			0.01	0.00	0.00	-0.01	-0.01	-0.01
			0.00	-0.01	0.00	0.00	0.01	
					0.00	0.00		
					0.00	0.00		
					0.00	0.00		
					0.00	0.00		
					0.00	0.00		
comp.	0.22	0.00	0.04	0.01	0.01	0.01	0.21	0.01

basis	Δ_{oop}	δ_{oop}	B_{2u}	B_{1u}	A_{2u}	$E_g(x)$	$E_g(y)$	A_{1u}
min.	0.28	0.00	0.08	-0.01	-0.25	0.10	0.02	-0.01
ext.	0.29	0.00	0.08	-0.01	-0.25	0.11	0.02	-0.01
			0.00	0.00	0.03	0.07	0.00	0.00
total	0.29	0.00	0.08	-0.01	-0.25	0.11	0.02	-0.01
			0.00	0.00	0.03	0.07	0.00	0.00
			0.00	0.00	-0.01	0.00	-0.01	
						0.00	-0.01	
						0.00	0.00	
comp.	0.29	0.00	0.08	0.01	0.25	0.13	0.02	0.01

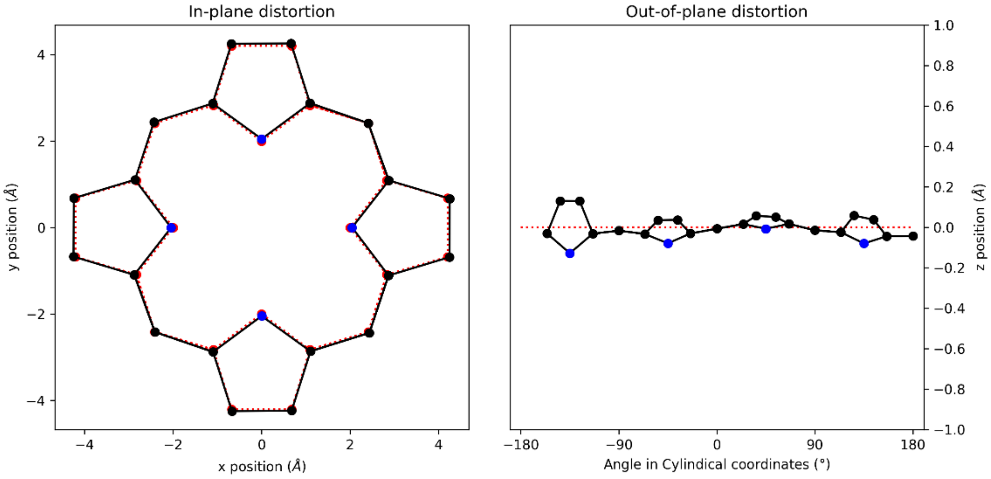

Figure S92: NSD result generated from **15** (in Å) (**A**) in-plane and (**B**) out-of-plane skeletal plots of the porphyrin core. Porphyrin is represented in black (C) and blue (N), with the reference structure (CuTPP) in red dotted lines.

basis	Δ_{ip}	δ_{ip}	B_{2g}	B_{1g}	$E_u(x)$	$E_u(y)$	A_{1g}	A_{2g}
min.	0.21	0.00	0.07	-0.01	0.00	0.00	0.20	-0.01
ext.	0.22	0.00	0.07	-0.01	0.00	0.00	0.20	-0.01
			0.00	-0.04	-0.01	0.02	0.00	-0.03
total	0.25	0.00	0.07	-0.01	0.00	0.00	0.20	-0.02
			0.00	-0.04	-0.01	0.02	0.00	-0.03
			-0.01	-0.04	0.00	0.00	0.07	-0.01
			-0.01	0.02	0.00	-0.03	-0.04	0.00
			0.01	0.00	0.02	0.02	0.00	-0.02
			0.01	-0.01	-0.01	0.02	0.02	
					0.00	-0.01		
					0.01	0.00		
					0.00	0.00		
					0.00	0.00		
					0.00	0.00		
comp.	0.25	0.00	0.07	0.07	0.03	0.04	0.22	0.04

basis	Δ_{oop}	δ_{oop}	B_{2u}	B_{1u}	A_{2u}	$E_g(x)$	$E_g(y)$	A_{1u}
min.	0.89	0.00	0.57	-0.67	-0.16	-0.01	-0.07	0.00
ext.	0.90	0.00	0.57	-0.67	-0.16	-0.01	-0.07	0.00
			-0.07	-0.02	-0.01	0.00	-0.05	0.00
total	0.90	0.00	0.57	-0.67	-0.16	-0.01	-0.07	0.00
			-0.07	-0.02	-0.01	0.00	-0.05	0.00
			-0.02	0.01	0.00	0.01	0.00	
						0.00	0.00	
						-0.02	0.00	
comp.	0.90	0.00	0.57	0.67	0.16	0.02	0.08	0.00

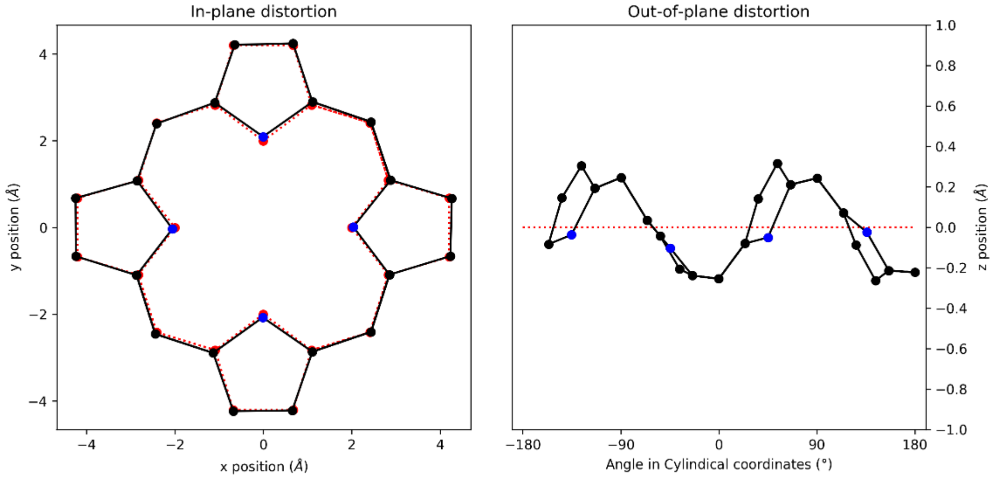

Figure S93: NSD result generated from **16** (in Å) (**A**) in-plane and (**B**) out-of-plane skeletal plots of the porphyrin core. Porphyrin is represented in black (C) and blue (N), with the reference structure (CuTPP) in red dotted lines.

basis	Δ_{ip}	δ_{ip}	B_{2g}	B_{1g}	$E_u(x)$	$E_u(y)$	A_{1g}	A_{2g}
min.	0.17	0.00	0.04	0.01	0.00	0.00	0.16	-0.01
ext.	0.19	0.00	0.04	0.00	0.00	0.00	0.16	-0.01
			0.00	-0.06	0.00	-0.04	-0.03	-0.01
total	0.21	0.00	0.04	0.00	0.00	0.00	0.17	-0.01
			0.00	-0.06	0.00	-0.04	-0.03	-0.01
			0.01	-0.05	0.00	0.00	0.07	-0.01
			0.00	0.00	-0.01	0.01	-0.01	0.00
			0.01	0.01	0.00	0.01	0.01	0.01
			0.01	-0.01	0.00	0.00	0.01	
					0.01	0.00		
					0.00	-0.01		
					0.00	0.00		
					0.00	0.00		
					0.00	-0.01		
comp.	0.21	0.00	0.04	0.08	0.02	0.04	0.19	0.02

basis	Δ_{oop}	δ_{oop}	B_{2u}	B_{1u}	A_{2u}	$E_g(x)$	$E_g(y)$	A_{1u}
min.	0.67	0.00	0.60	-0.21	-0.10	-0.14	0.11	0.01
ext.	0.68	0.00	0.60	-0.21	-0.10	-0.14	0.11	0.01
			-0.07	-0.01	-0.02	0.04	0.07	0.01
total	0.68	0.00	0.60	-0.21	-0.10	-0.14	0.11	0.01
			-0.07	-0.01	-0.02	0.04	0.07	0.01
			-0.01	0.00	0.00	0.02	0.01	
						0.01	-0.01	
						-0.02	0.00	
comp.	0.68	0.00	0.61	0.21	0.10	0.15	0.13	0.02

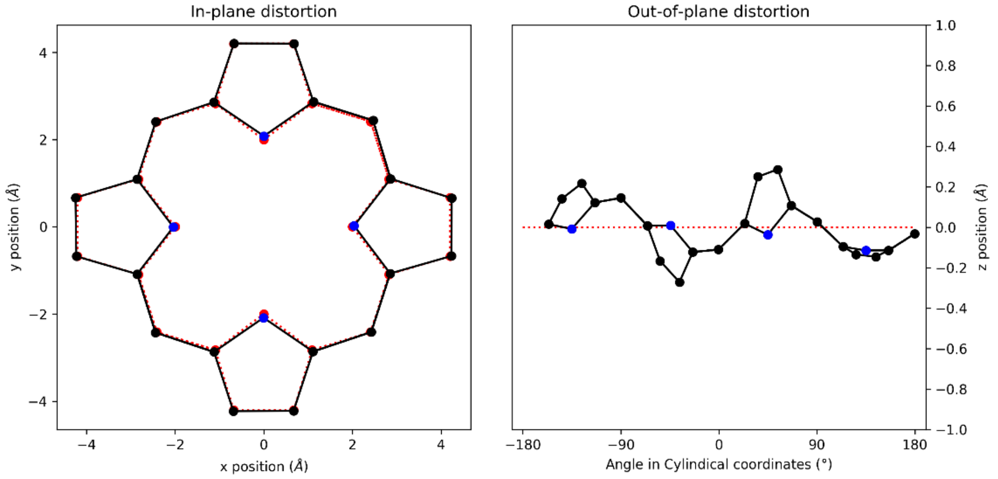

Figure S94: NSD result generated from **16A** (in Å) (**A**) in-plane and (**B**) out-of-plane skeletal plots of the porphyrin core. Porphyrin is represented in black (C) and blue (N), with the reference structure (CuTPP) in red dotted lines.

basis	Δ_{ip}	δ_{ip}	B_{2g}	B_{1g}	$E_u(x)$	$E_u(y)$	A_{1g}	A_{2g}
min.	0.03	0.00	-0.01	0.00	0.01	0.02	0.01	0.01
ext.	0.05	0.00	-0.01	0.00	0.01	0.02	0.01	0.01
			-0.02	0.00	0.01	0.00	0.02	-0.01
total	0.09	0.00	-0.01	0.00	0.01	0.02	0.01	0.01
			-0.02	0.00	0.01	0.00	0.02	-0.01
			-0.01	0.01	-0.01	0.00	0.03	0.00
			0.00	0.01	-0.02	0.01	-0.03	0.00
			-0.01	0.00	0.01	0.00	-0.02	-0.01
			-0.01	-0.02	-0.02	-0.01	0.02	
					-0.02	-0.01		
					0.00	0.00		
					-0.03	0.01		
					0.03	0.00		
					0.00	-0.01		
comp.	0.09	0.00	0.03	0.02	0.06	0.03	0.06	0.02

basis	Δ_{oop}	δ_{oop}	B_{2u}	B_{1u}	A_{2u}	$E_g(x)$	$E_g(y)$	A_{1u}
min.	0.73	0.00	0.23	-0.69	0.02	-0.03	0.05	-0.01
ext.	0.74	0.00	0.23	-0.69	0.02	-0.03	0.05	-0.01
			0.04	-0.02	0.06	0.01	0.00	0.01
total	0.74	0.00	0.23	-0.69	0.02	-0.03	0.05	-0.01
			0.04	-0.02	0.06	0.01	0.00	0.01
			-0.01	-0.01	-0.01	0.00	-0.01	
						0.02	0.01	
						0.00	0.00	
comp.	0.74	0.00	0.23	0.69	0.06	0.04	0.05	0.01

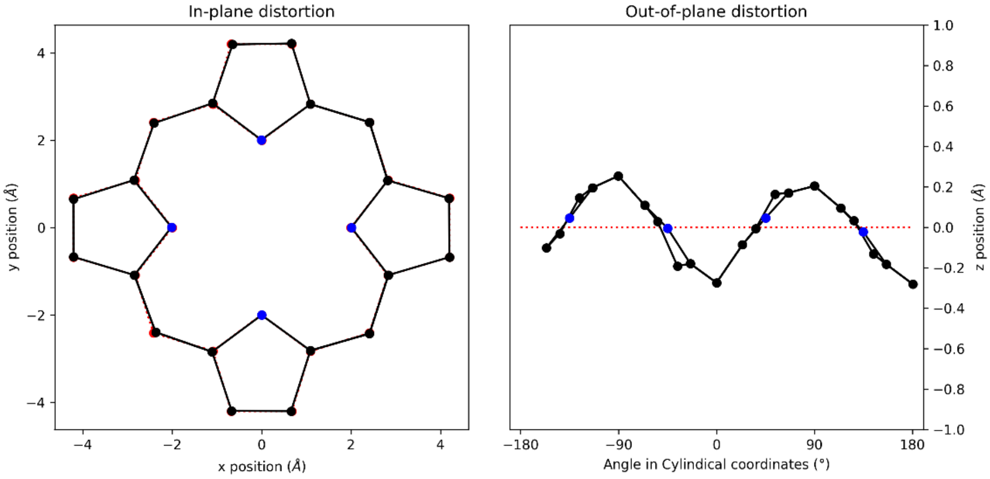

Figure S95: NSD result generated from **17** (in Å) (**A**) in-plane and (**B**) out-of-plane skeletal plots of the porphyrin core. Porphyrin is represented in black (C) and blue (N), with the reference structure (CuTPP) in red dotted lines.

basis	Δ_{ip}	δ_{ip}	B_{2g}	B_{1g}	$E_u(x)$	$E_u(y)$	A_{1g}	A_{2g}
min.	0.18	0.00	0.01	0.04	-0.01	0.04	0.17	-0.01
ext.	0.18	0.00	0.01	0.04	-0.01	0.04	0.17	-0.01
			0.01	0.01	-0.01	0.02	0.03	-0.02
total	0.19	0.00	0.01	0.04	-0.01	0.04	0.17	-0.01
			0.01	0.01	-0.01	0.02	0.03	-0.02
			0.00	0.00	-0.01	-0.01	0.03	0.00
			-0.01	0.00	-0.01	-0.02	-0.04	0.00
			0.01	0.01	0.01	0.01	-0.01	0.00
			0.00	0.00	0.00	-0.01	0.01	
					-0.01	0.00		
					-0.01	-0.01		
					0.00	-0.01		
					0.00	0.01		
					0.00	0.00		
comp.	0.19	0.00	0.02	0.04	0.02	0.05	0.18	0.02

basis	Δ_{oop}	δ_{oop}	B_{2u}	B_{1u}	A_{2u}	$E_g(x)$	$E_g(y)$	A_{1u}
min.	0.64	0.00	0.27	-0.56	-0.01	0.11	0.05	0.00
ext.	0.64	0.00	0.27	-0.56	-0.02	0.11	0.05	0.00
			-0.03	-0.01	-0.04	-0.06	-0.05	0.01
total	0.64	0.00	0.27	-0.56	-0.02	0.11	0.05	0.00
			-0.03	-0.01	-0.04	-0.06	-0.05	0.01
			0.00	-0.01	0.01	0.00	0.01	
						0.00	0.00	
						0.00	0.01	
comp.	0.64	0.00	0.27	0.56	0.04	0.12	0.07	0.01

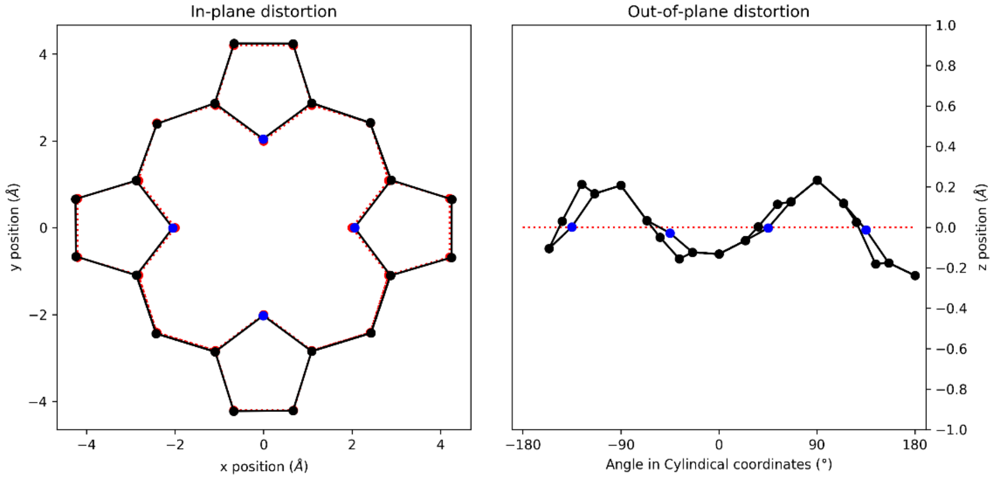

Figure S96: NSD result generated from **18** (in Å) (**A**) in-plane and (**B**) out-of-plane skeletal plots of the porphyrin core. Porphyrin is represented in black (C) and blue (N), with the reference structure (CuTPP) in red dotted lines.

basis	Δ_{ip}	δ_{ip}	B_{2g}	B_{1g}	$E_u(x)$	$E_u(y)$	A_{1g}	A_{2g}
min.	0.23	0.00	0.00	-0.02	0.02	0.00	0.23	0.00
ext.	0.23	0.00	0.00	-0.02	0.02	0.00	0.23	0.00
			0.00	-0.04	0.00	0.00	0.01	0.00
total	0.25	0.00	0.00	-0.02	0.02	0.00	0.23	0.00
			0.00	-0.04	0.00	0.00	0.01	0.00
			0.00	-0.03	-0.02	0.00	0.05	0.00
			0.00	0.00	-0.03	0.00	-0.01	0.00
			0.00	0.01	0.01	0.00	0.01	0.00
			0.00	-0.01	0.00	0.00	0.01	
					0.00	0.00		
					0.00	0.00		
					-0.01	0.00		
					0.01	0.00		
					0.00	0.00		
comp.	0.25	0.00	0.00	0.05	0.04	0.00	0.24	0.00

basis	Δ_{oop}	δ_{oop}	B_{2u}	B_{1u}	A_{2u}	$E_g(x)$	$E_g(y)$	A_{1u}
min.	0.25	0.00	0.00	-0.25	0.00	0.00	0.00	0.00
ext.	0.25	0.00	0.00	-0.25	0.00	0.00	0.00	0.00
			0.00	0.00	0.00	-0.02	0.00	0.00
total	0.25	0.00	0.00	-0.25	0.00	0.00	0.00	0.00
			0.00	0.00	0.00	-0.02	0.00	0.00
			0.00	-0.01	0.00	0.01	0.00	
						0.01	0.00	
						0.00	0.00	
comp.	0.25	0.00	0.00	0.25	0.00	0.02	0.00	0.01

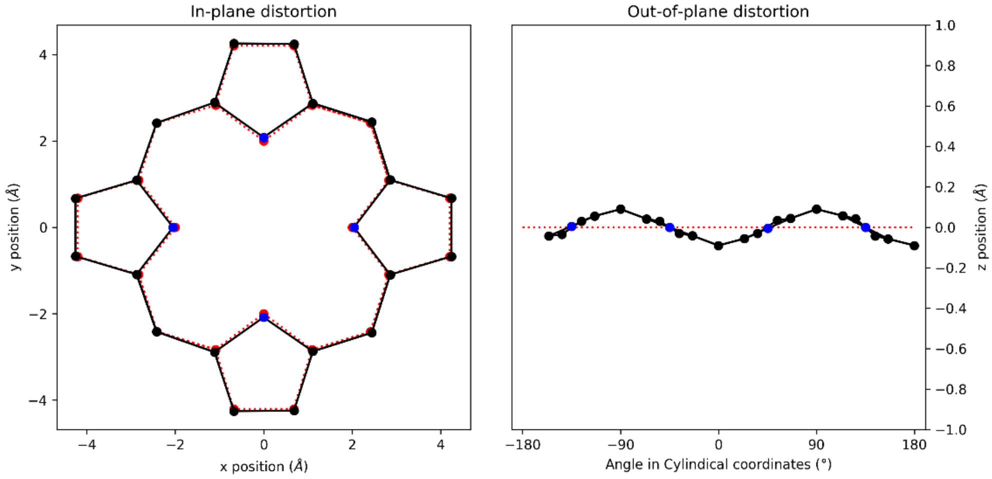

Figure S97: NSD result generated from **19** (in Å) (**A**) in-plane and (**B**) out-of-plane skeletal plots of the porphyrin core. Porphyrin is represented in black (C) and blue (N), with the reference structure (CuTPP) in red dotted lines.

basis	Δ_{ip}	δ_{ip}	B_{2g}	B_{1g}	$E_u(x)$	$E_u(y)$	A_{1g}	A_{2g}
min.	0.26	0.00	-0.01	0.06	0.00	0.00	0.26	0.00
ext.	0.28	0.00	-0.01	0.06	0.00	0.00	0.26	0.00
			-0.01	0.06	0.00	0.00	0.06	0.00
total	0.29	0.00	-0.01	0.07	0.00	0.00	0.26	0.00
			-0.01	0.06	0.00	0.00	0.06	0.00
			0.01	0.05	0.00	0.00	0.04	0.00
			0.00	0.00	0.00	0.00	0.02	0.00
			0.00	-0.01	0.00	0.00	0.00	0.00
			0.00	0.01	0.00	0.00	0.01	
					0.00	0.00		
					0.00	0.00		
					0.00	0.00		
					0.00	0.00		
					0.00	0.00		
comp.	0.29	0.00	0.02	0.10	0.00	0.00	0.27	0.00

basis	Δ_{oop}	δ_{oop}	B_{2u}	B_{1u}	A_{2u}	$E_g(x)$	$E_g(y)$	A_{1u}
min.	0.12	0.00	0.00	0.00	0.00	0.08	0.09	0.00
ext.	0.14	0.00	0.00	0.00	0.00	0.09	0.09	0.00
			0.00	0.00	0.00	0.05	-0.02	0.00
total	0.14	0.00	0.00	0.00	0.00	0.09	0.09	0.00
			0.00	0.00	0.00	0.05	-0.02	0.00
			0.00	0.00	0.00	-0.01	0.01	
						0.00	0.00	
						0.00	0.00	
comp.	0.14	0.00	0.00	0.00	0.00	0.10	0.09	0.00

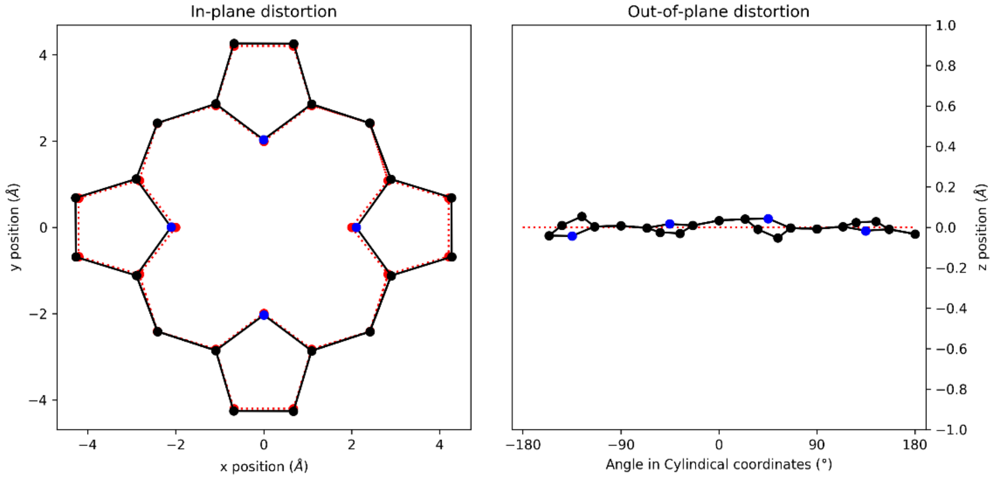

Figure S98: NSD result generated from **20** (in Å) (**A**) in-plane and (**B**) out-of-plane skeletal plots of the porphyrin core. Porphyrin is represented in black (C) and blue (N), with the reference structure (CuTPP) in red dotted lines.

basis	Δ_{ip}	δ_{ip}	B_{2g}	B_{1g}	$E_u(x)$	$E_u(y)$	A_{1g}	A_{2g}
min.	0.47	0.01	0.02	-0.01	0.00	-0.03	-0.46	0.04
ext.	0.50	0.01	0.02	-0.01	0.00	-0.03	-0.46	0.04
			0.01	-0.01	-0.02	-0.01	0.17	-0.06
total	0.52	0.00	0.02	-0.01	0.00	-0.03	-0.46	0.03
			0.01	-0.01	-0.02	-0.01	0.17	-0.06
			-0.02	0.00	-0.03	0.00	0.01	0.00
			0.00	0.00	-0.04	0.02	-0.13	-0.01
			0.00	0.00	0.01	-0.01	-0.01	-0.02
			0.00	0.00	0.00	0.00	0.08	
					0.00	-0.01		
					0.00	0.00		
					0.00	0.01		
					0.01	0.00		
					0.00	0.00		
comp.	0.52	0.00	0.03	0.02	0.05	0.04	0.51	0.07

basis	Δ_{oop}	δ_{oop}	B_{2u}	B_{1u}	A_{2u}	$E_g(x)$	$E_g(y)$	A_{1u}
min.	1.89	0.00	0.34	-1.85	-0.09	0.09	0.00	-0.01
ext.	1.89	0.00	0.34	-1.85	-0.09	0.09	0.00	-0.01
			0.00	-0.02	0.01	-0.02	-0.02	0.00
total	1.89	0.00	0.34	-1.85	-0.09	0.09	0.00	-0.01
			0.00	-0.02	0.01	-0.02	-0.02	0.00
			0.00	-0.02	0.00	-0.02	-0.02	
						0.00	0.00	
						0.00	0.01	
comp.	1.89	0.00	0.34	1.85	0.09	0.10	0.03	0.01

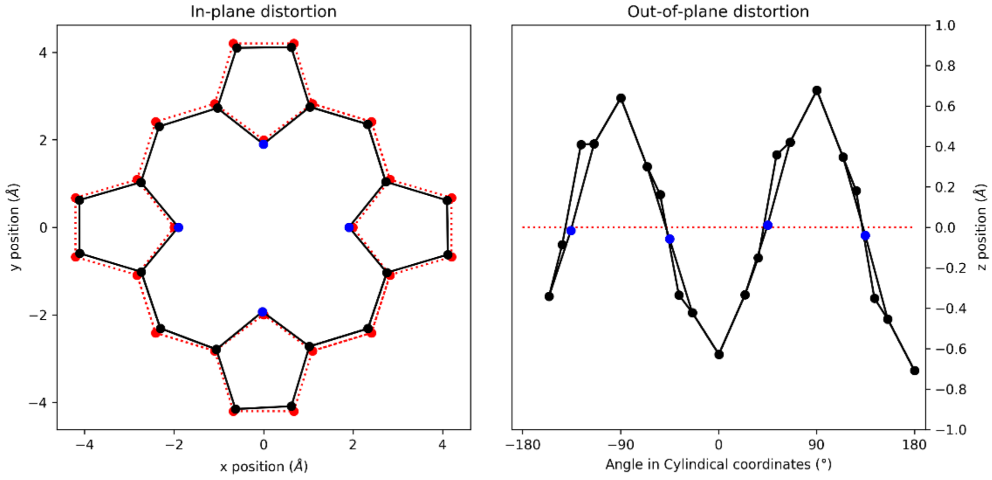

Figure S99: NSD result generated from **21** (in Å) (**A**) in-plane and (**B**) out-of-plane skeletal plots of the porphyrin core. Porphyrin is represented in black (C) and blue (N), with the reference structure (CuTPP) in red dotted lines.

basis	Δ_{ip}	δ_{ip}	B_{2g}	B_{1g}	$E_u(x)$	$E_u(y)$	A_{1g}	A_{2g}
min.	0.42	0.01	-0.04	0.00	-0.01	-0.01	-0.41	0.00
ext.	0.44	0.00	-0.04	0.00	-0.01	-0.01	-0.41	0.00
			0.01	0.00	-0.02	-0.02	0.13	0.00
total	0.46	0.00	-0.04	0.00	-0.01	-0.01	-0.41	0.00
			0.01	0.00	-0.02	-0.02	0.13	0.00
			0.03	0.00	0.01	0.01	0.01	0.00
			-0.01	0.00	0.03	0.03	-0.10	0.00
			0.00	0.00	-0.02	-0.02	-0.01	0.00
			0.02	0.00	0.01	0.01	0.06	
					0.00	0.00		
					0.01	0.01		
					0.00	0.00		
					0.00	0.00		
					0.00	0.00		
comp.	0.46	0.00	0.06	0.00	0.05	0.05	0.45	0.00

basis	Δ_{oop}	δ_{oop}	B_{2u}	B_{1u}	A_{2u}	$E_g(x)$	$E_g(y)$	A_{1u}
min.	1.74	0.00	0.00	1.72	-0.09	0.14	-0.14	0.00
ext.	1.74	0.00	0.00	1.72	-0.09	0.14	-0.14	0.00
			0.00	0.02	0.05	-0.01	0.01	0.00
total	1.74	0.00	0.00	1.72	-0.09	0.14	-0.14	0.00
			0.00	0.02	0.05	-0.01	0.01	0.00
			0.00	0.03	0.00	-0.05	0.05	
						-0.01	0.01	
						0.00	0.00	
comp.	1.74	0.00	0.00	1.72	0.10	0.15	0.15	0.00

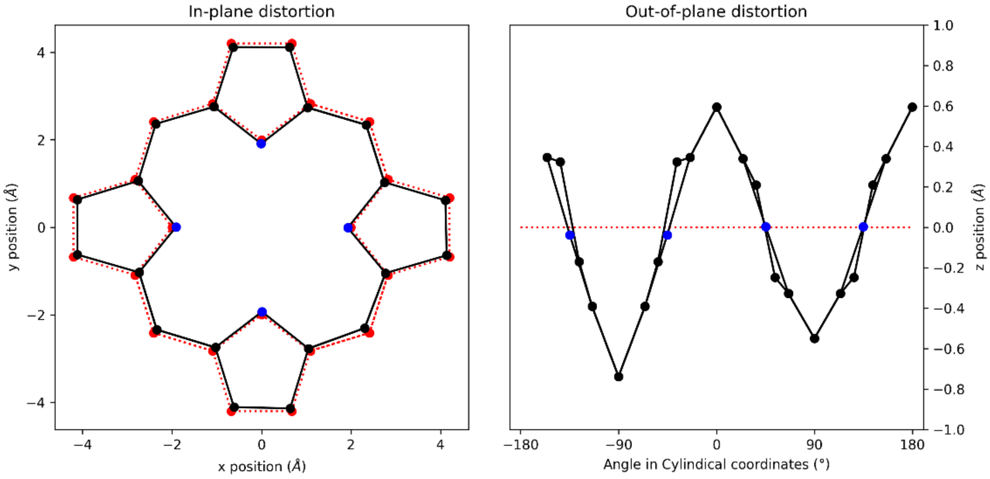

Figure S100: NSD result generated from **22** (in Å) (**A**) in-plane and (**B**) out-of-plane skeletal plots of the porphyrin core. Porphyrin is represented in black (C) and blue (N), with the reference structure (CuTPP) in red dotted lines.

basis	Δ_{ip}	δ_{ip}	B_{2g}	B_{1g}	$E_u(x)$	$E_u(y)$	A_{1g}	A_{2g}
min.	0.23	0.00	-0.05	0.01	-0.01	0.02	-0.22	0.00
ext.	0.23	0.00	-0.05	0.01	-0.01	0.02	-0.22	0.00
			0.03	0.01	-0.02	0.00	0.04	-0.01
total	0.26	0.00	-0.05	0.01	-0.01	0.02	-0.22	0.00
			0.03	0.01	-0.02	0.00	0.04	-0.01
			-0.02	-0.01	0.01	0.00	-0.04	0.00
			0.00	0.02	0.00	-0.02	-0.03	-0.03
			0.01	0.01	-0.01	-0.02	-0.03	0.01
			0.00	0.01	0.01	0.01	0.02	
					0.04	0.00		
					0.01	-0.03		
					0.01	-0.01		
					0.00	0.01		
					-0.02	0.00		
comp.	0.26	0.00	0.06	0.03	0.05	0.05	0.24	0.03

basis	Δ_{oop}	δ_{oop}	B_{2u}	B_{1u}	A_{2u}	$E_g(x)$	$E_g(y)$	A_{1u}
min.	0.96	0.00	0.23	0.93	-0.02	0.07	-0.01	-0.02
ext.	0.97	0.00	0.23	0.93	-0.02	0.07	-0.01	-0.02
			0.04	0.02	0.01	-0.04	0.05	0.02
total	0.97	0.00	0.23	0.93	-0.02	0.07	-0.01	-0.02
			0.04	0.02	0.01	-0.04	0.05	0.02
			0.00	0.02	0.01	-0.03	0.02	
						0.01	-0.01	
						0.03	0.00	
comp.	0.97	0.00	0.24	0.93	0.02	0.09	0.05	0.03

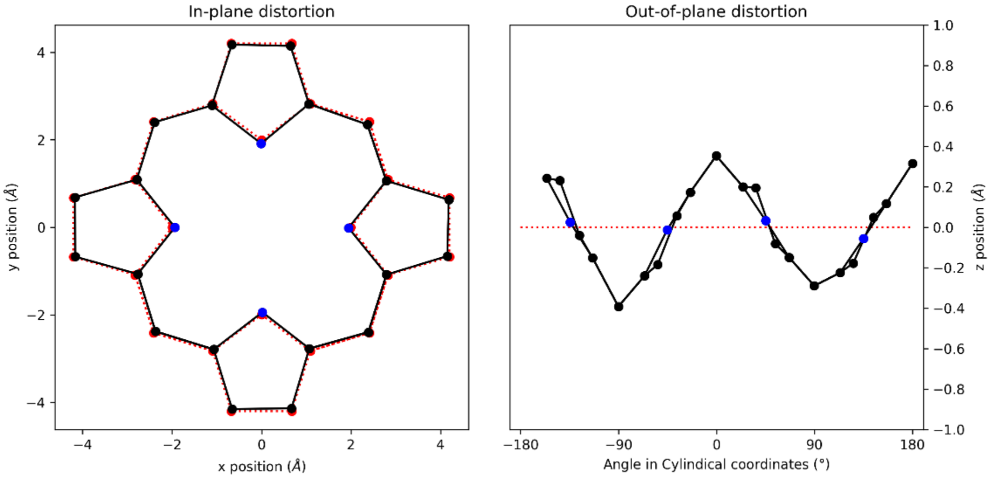

Figure S101: NSD result generated from **23** (in Å) (**A**) in-plane and (**B**) out-of-plane skeletal plots of the porphyrin core. Porphyrin is represented in black (C) and blue (N), with the reference structure (CuTPP) in red dotted lines.

basis	Δ_{ip}	δ_{ip}	B_{2g}	B_{1g}	$E_u(x)$	$E_u(y)$	A_{1g}	A_{2g}
min.	0.21	0.00	-0.07	0.01	0.00	0.00	0.20	0.00
ext.	0.22	0.00	-0.07	0.01	0.00	0.00	0.20	0.00
			-0.01	-0.02	-0.02	0.01	-0.04	0.00
total	0.23	0.00	-0.07	0.01	0.00	0.00	0.20	0.00
			-0.01	-0.02	-0.02	0.01	-0.04	0.00
			-0.01	-0.02	0.00	0.00	0.05	0.00
			0.00	0.00	0.00	0.00	-0.01	0.00
			0.00	0.00	0.00	0.00	0.02	0.00
			0.00	0.00	0.00	0.00	0.01	
					0.00	0.00		
					0.00	0.00		
					0.00	0.00		
					0.00	0.00		
					0.00	0.01		
comp.	0.23	0.00	0.07	0.03	0.02	0.02	0.21	0.00

basis	Δ_{oop}	δ_{oop}	B_{2u}	B_{1u}	A_{2u}	$E_g(x)$	$E_g(y)$	A_{1u}
min.	0.29	0.00	-0.10	-0.16	-0.01	0.01	0.21	0.00
ext.	0.30	0.00	-0.10	-0.16	-0.01	0.01	0.21	0.00
			-0.04	-0.01	0.01	0.04	-0.07	0.00
total	0.30	0.00	-0.10	-0.16	-0.01	0.01	0.21	0.00
			-0.04	-0.01	0.01	0.04	-0.07	0.00
			0.00	-0.01	0.00	0.01	-0.02	
						0.00	-0.01	
						0.01	0.01	
comp.	0.30	0.00	0.11	0.16	0.01	0.04	0.22	0.00

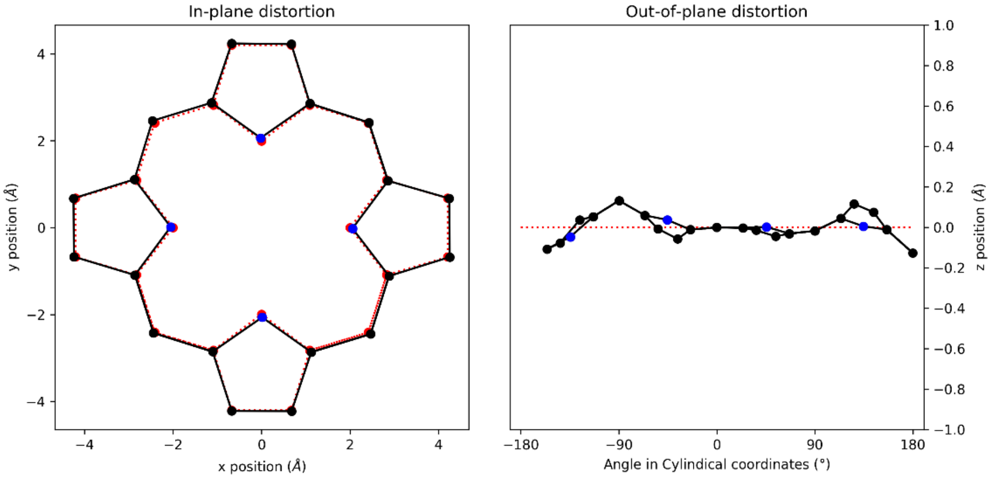

Figure S102: NSD result generated from **24** (in Å) (**A**) in-plane and (**B**) out-of-plane skeletal plots of the porphyrin core. Porphyrin is represented in black (C) and blue (N), with the reference structure (CuTPP) in red dotted lines.

NSD tables and plots for DFT structures series 1

basis	Δ_{ip}	δ_{ip}	B_{2g}	B_{1g}	$E_u(x)$	$E_u(y)$	A_{1g}	A_{2g}
min.	0.38	0.00	-0.32	-0.05	0.00	0.00	0.21	0.00
ext.	0.40	0.00	-0.32	-0.05	0.00	0.00	0.21	0.00
			-0.05	-0.09	0.00	0.00	0.00	0.00
total	0.41	0.00	-0.32	-0.05	0.00	0.00	0.21	0.00
			-0.05	-0.09	0.00	0.00	0.00	0.00
			0.00	-0.06	0.00	0.00	0.05	0.00
			0.00	0.00	0.00	0.00	0.01	0.00
			0.00	0.01	0.00	0.00	0.02	0.00
			-0.01	-0.01	0.00	0.00	0.01	
					0.00	0.00		
					0.00	0.00		
					0.00	0.00		
					0.00	0.00		
					0.00	0.00		
comp.	0.41	0.00	0.32	0.12	0.00	0.00	0.22	0.00

basis	Δ_{oop}	δ_{oop}	B_{2u}	B_{1u}	A_{2u}	$E_g(x)$	$E_g(y)$	A_{1u}
min.	0.08	0.00	0.00	0.00	0.00	0.06	0.06	0.00
ext.	0.10	0.00	0.00	0.00	0.00	0.06	0.06	0.00
			0.00	0.00	0.00	0.05	0.03	0.00
total	0.10	0.00	0.00	0.00	0.00	0.06	0.06	0.00
			0.00	0.00	0.00	0.05	0.03	0.00
			0.00	0.00	0.00	0.00	0.00	
						0.00	0.00	
						0.00	0.00	
comp.	0.10	0.00	0.00	0.00	0.00	0.08	0.07	0.00

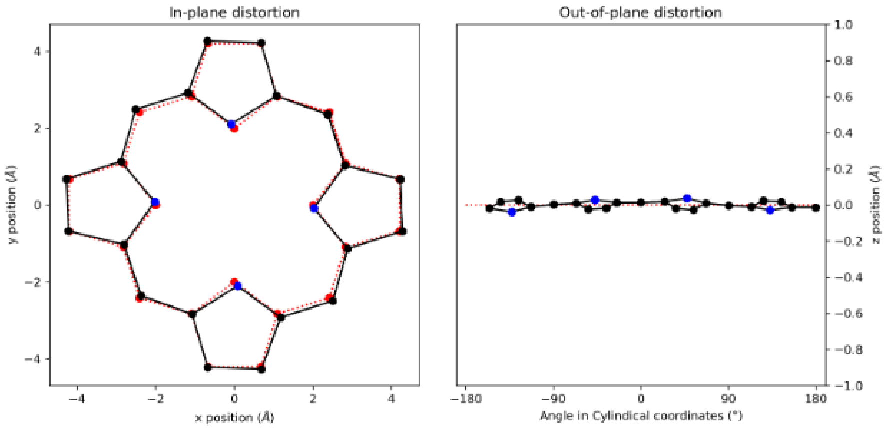

Figure S103: NSD result generated from **1:1** (in Å) (**A**) in-plane and (**B**) out-of-plane skeletal plots of the porphyrin core. Porphyrin is represented in black (C) and blue (N), with the reference structure (CuTPP) in red dotted lines.

basis	Δ_{ip}	δ_{ip}	B_{2g}	B_{1g}	$E_u(x)$	$E_u(y)$	A_{1g}	A_{2g}
min.	0.14	0.00	-0.06	0.00	0.00	0.00	-0.12	0.00
ext.	0.14	0.00	-0.07	0.00	0.00	0.00	-0.12	0.00
			-0.02	0.00	0.00	0.00	0.00	0.00
total	0.15	0.00	-0.06	0.00	0.00	0.00	-0.12	0.00
			-0.02	0.00	0.00	0.00	0.00	0.00
			-0.01	0.00	0.00	0.00	-0.03	0.00
			0.01	0.00	0.00	0.00	-0.01	0.00
			0.00	0.00	0.00	0.00	0.00	0.00
			0.00	0.00	0.00	0.00	0.00	
					0.00	0.00		
					0.00	0.00		
					0.00	0.00		
					0.00	0.00		
					0.00	0.00		
comp.	0.15	0.00	0.07	0.00	0.00	0.00	0.13	0.00

basis	Δ_{oop}	δ_{oop}	B_{2u}	B_{1u}	A_{2u}	$E_g(x)$	$E_g(y)$	A_{1u}
min.	0.57	0.00	0.00	0.57	0.01	-0.01	-0.01	0.00
ext.	0.57	0.00	0.00	0.57	0.01	-0.01	-0.01	0.00
			0.00	0.00	0.00	-0.01	-0.01	0.00
total	0.57	0.00	0.00	0.57	0.01	-0.01	-0.01	0.00
			0.00	0.00	0.00	-0.01	-0.01	0.00
			0.00	0.01	0.00	0.00	0.00	
						0.00	0.00	
						0.00	0.00	
comp.	0.57	0.00	0.00	0.57	0.01	0.02	0.02	0.00

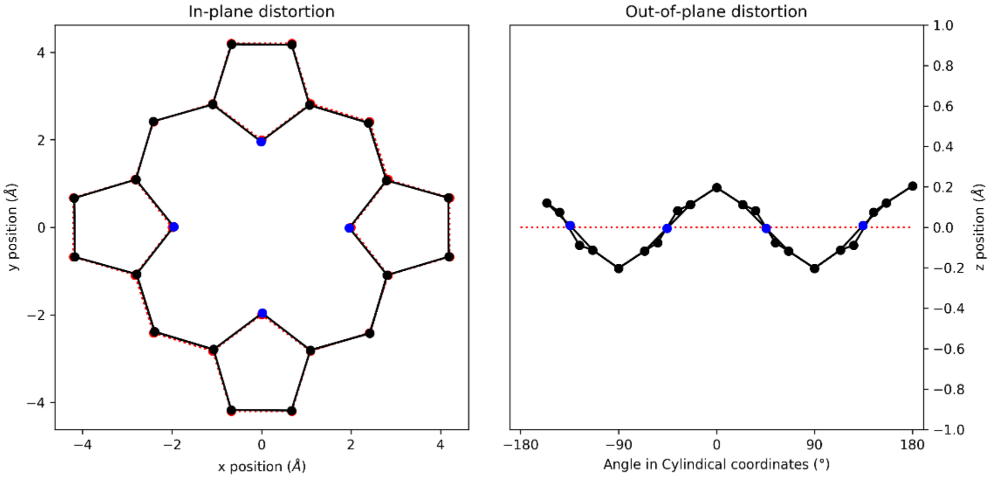

Figure S104: NSD result generated from **1:2** (in Å) (**A**) in-plane and (**B**) out-of-plane skeletal plots of the porphyrin core. Porphyrin is represented in black (C) and blue (N), with the reference structure (CuTPP) in red dotted lines.

basis	Δ_{ip}	δ_{ip}	B_{2g}	B_{1g}	$E_u(x)$	$E_u(y)$	A_{1g}	A_{2g}
min.	0.37	0.00	-0.31	-0.04	0.00	0.00	0.21	-0.01
ext.	0.39	0.00	-0.31	-0.04	0.00	0.00	0.21	-0.01
			-0.05	-0.09	0.01	0.01	0.01	0.00
total	0.40	0.00	-0.31	-0.04	0.00	0.00	0.21	-0.01
			-0.05	-0.09	0.01	0.01	0.01	0.00
			-0.01	-0.06	0.00	0.00	0.05	0.00
			0.00	0.00	-0.01	0.02	0.01	0.00
			0.00	0.01	0.00	-0.01	0.02	0.00
			-0.01	-0.01	0.00	0.00	0.01	
					0.00	0.00		
					0.00	0.00		
					0.00	0.01		
					0.00	0.00		
					-0.01	0.02		
comp.	0.40	0.00	0.31	0.11	0.02	0.03	0.22	0.01

basis	Δ_{oop}	δ_{oop}	B_{2u}	B_{1u}	A_{2u}	$E_g(x)$	$E_g(y)$	A_{1u}
min.	0.09	0.00	0.00	-0.01	0.00	-0.06	-0.06	0.00
ext.	0.11	0.00	0.00	-0.01	0.00	-0.07	-0.06	0.00
			0.00	0.00	0.00	-0.05	-0.03	0.00
total	0.11	0.00	0.00	-0.01	0.00	-0.07	-0.06	0.00
			0.00	0.00	0.00	-0.05	-0.03	0.00
			0.00	0.00	0.00	0.00	0.00	
						0.00	0.00	
						0.00	0.00	
comp.	0.11	0.00	0.00	0.01	0.00	0.08	0.07	0.00

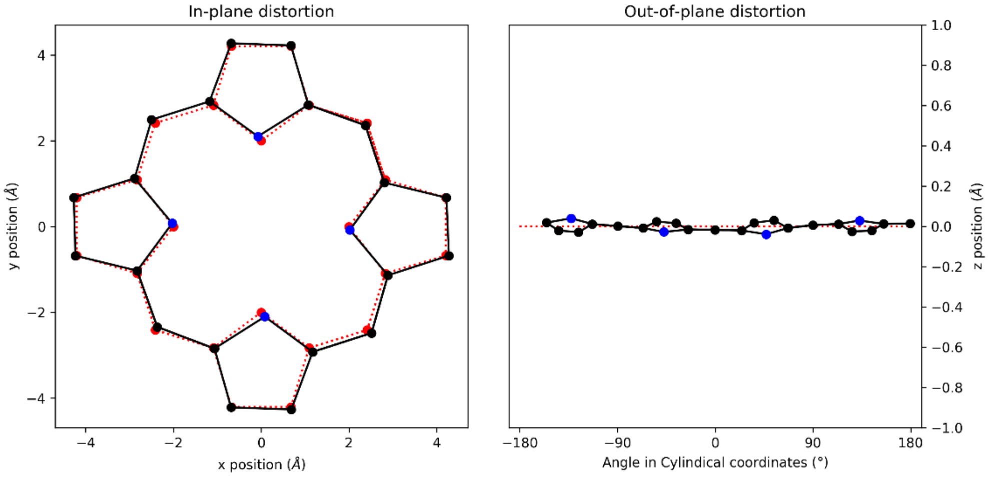

Figure S105: NSD result generated from **1:3** (in Å) (**A**) in-plane and (**B**) out-of-plane skeletal plots of the porphyrin core. Porphyrin is represented in black (C) and blue (N), with the reference structure (CuTPP) in red dotted lines.

basis	Δ_{ip}	δ_{ip}	B_{2g}	B_{1g}	$E_u(x)$	$E_u(y)$	A_{1g}	A_{2g}
min.	0.14	0.00	-0.07	0.00	0.00	0.00	-0.13	0.00
ext.	0.15	0.00	-0.07	0.00	0.00	0.00	-0.13	0.00
			-0.02	0.00	0.00	0.00	0.01	0.00
total	0.15	0.00	-0.07	0.00	0.00	0.00	-0.13	0.00
			-0.02	0.00	0.00	0.00	0.01	0.00
			-0.02	0.00	0.00	0.00	-0.03	0.00
			0.01	0.00	0.01	-0.01	-0.01	0.00
			0.00	0.00	0.00	0.00	0.00	0.00
			-0.01	0.00	0.00	0.00	0.00	
					0.00	0.00		
					0.00	0.00		
					0.01	0.00		
					0.00	0.00		
					0.01	-0.01		
comp.	0.15	0.00	0.08	0.00	0.02	0.02	0.13	0.00

basis	Δ_{oop}	δ_{oop}	B_{2u}	B_{1u}	A_{2u}	$E_g(x)$	$E_g(y)$	A_{1u}
min.	0.61	0.00	0.00	0.61	0.01	-0.02	-0.02	0.00
ext.	0.61	0.00	0.00	0.61	0.01	-0.02	-0.02	0.00
			0.00	0.00	0.00	-0.01	-0.01	0.00
total	0.61	0.00	0.00	0.61	0.01	-0.02	-0.02	0.00
			0.00	0.00	0.00	-0.01	-0.01	0.00
			0.00	0.01	0.00	0.00	0.00	
						0.00	0.00	
						0.00	0.00	
comp.	0.61	0.00	0.00	0.61	0.01	0.02	0.02	0.00

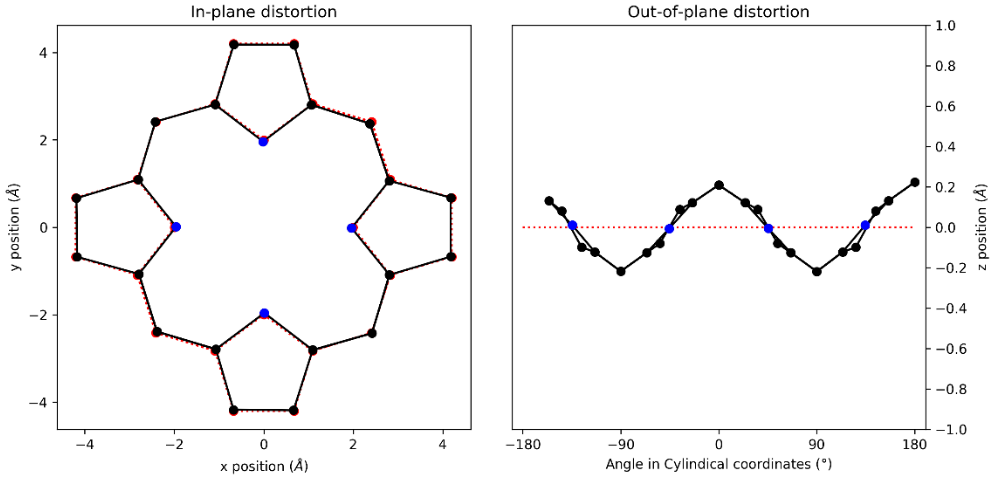

Figure S106: NSD result generated from **1:4** (in Å) (**A**) in-plane and (**B**) out-of-plane skeletal plots of the porphyrin core. Porphyrin is represented in black (C) and blue (N), with the reference structure (CuTPP) in red dotted lines.

basis	Δ_{ip}	δ_{ip}	B_{2g}	B_{1g}	$E_u(x)$	$E_u(y)$	A_{1g}	A_{2g}
min.	0.37	0.00	0.31	0.03	0.00	0.00	0.21	-0.01
ext.	0.39	0.00	0.31	0.03	0.00	0.00	0.21	-0.01
			0.05	0.08	0.00	0.00	0.02	0.01
total	0.40	0.00	0.31	0.03	0.00	0.00	0.21	-0.01
			0.05	0.09	0.00	0.00	0.02	0.01
			0.03	0.06	0.00	0.00	0.05	0.00
			-0.01	0.00	0.00	0.00	0.00	0.00
			0.00	-0.01	0.00	0.00	0.02	0.00
			0.01	0.01	0.00	0.00	0.00	
					0.00	0.00		
					0.00	0.00		
					0.00	0.00		
					0.00	0.00		
					0.00	0.00		
comp.	0.40	0.00	0.32	0.11	0.00	0.00	0.22	0.01

basis	Δ_{oop}	δ_{oop}	B_{2u}	B_{1u}	A_{2u}	$E_g(x)$	$E_g(y)$	A_{1u}
min.	0.09	0.00	0.00	0.00	0.00	-0.06	0.07	0.00
ext.	0.11	0.00	0.00	0.00	0.00	-0.06	0.07	0.00
			0.00	0.00	0.00	-0.03	0.05	0.00
total	0.11	0.00	0.00	0.00	0.00	-0.06	0.07	0.00
			0.00	0.00	0.00	-0.03	0.05	0.00
			0.00	0.00	0.00	0.00	0.00	
						0.00	0.00	
						0.00	0.00	
comp.	0.11	0.00	0.00	0.00	0.00	0.07	0.09	0.00

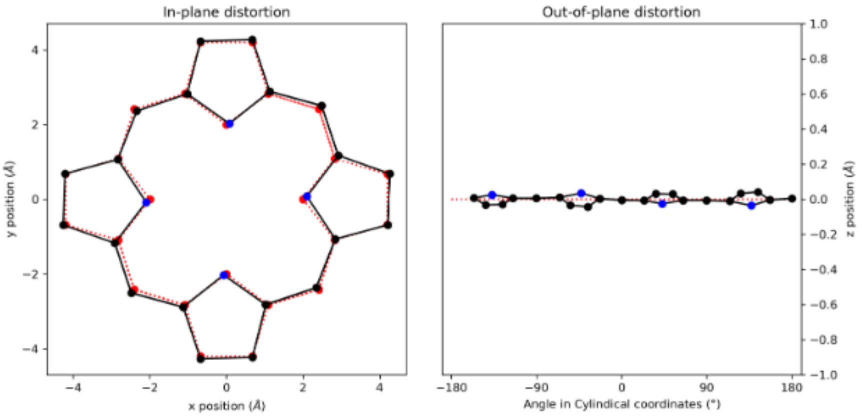

Figure S107: NSD result generated from **1:5** (in Å) (**A**) in-plane and (**B**) out-of-plane skeletal plots of the porphyrin core. Porphyrin is represented in black (C) and blue (N), with the reference structure (CuTPP) in red dotted lines.

basis	Δ_{ip}	δ_{ip}	B_{2g}	B_{1g}	$E_u(x)$	$E_u(y)$	A_{1g}	A_{2g}
min.	0.15	0.00	-0.08	0.00	0.00	0.00	-0.12	0.00
ext.	0.15	0.00	-0.08	0.00	0.00	0.00	-0.12	0.00
			-0.02	0.00	0.00	0.00	0.02	0.00
total	0.16	0.00	-0.08	0.00	0.00	0.00	-0.13	0.00
			-0.02	0.00	0.00	0.00	0.02	0.00
			-0.03	0.00	0.00	0.00	-0.03	0.00
			0.02	0.00	0.00	0.00	-0.01	0.00
			0.01	0.00	0.00	0.00	-0.01	0.00
			-0.01	0.00	0.00	0.00	0.00	
					0.00	0.00		
					0.00	0.00		
					0.00	0.00		
					0.00	0.00		
					0.00	0.00		
comp.	0.16	0.00	0.09	0.00	0.00	0.00	0.13	0.00

basis	Δ_{oop}	δ_{oop}	B_{2u}	B_{1u}	A_{2u}	$E_g(x)$	$E_g(y)$	A_{1u}
min.	0.59	0.00	0.00	0.59	0.01	-0.02	-0.02	0.00
ext.	0.59	0.00	0.00	0.59	0.01	-0.02	-0.02	0.00
			0.00	0.00	0.00	-0.01	-0.01	0.00
total	0.59	0.00	0.00	0.59	0.01	-0.02	-0.02	0.00
			0.00	0.00	0.00	-0.01	-0.01	0.00
			0.00	0.01	0.00	0.00	0.00	
						0.00	0.00	
						0.00	0.00	
comp.	0.59	0.00	0.00	0.59	0.01	0.02	0.02	0.00

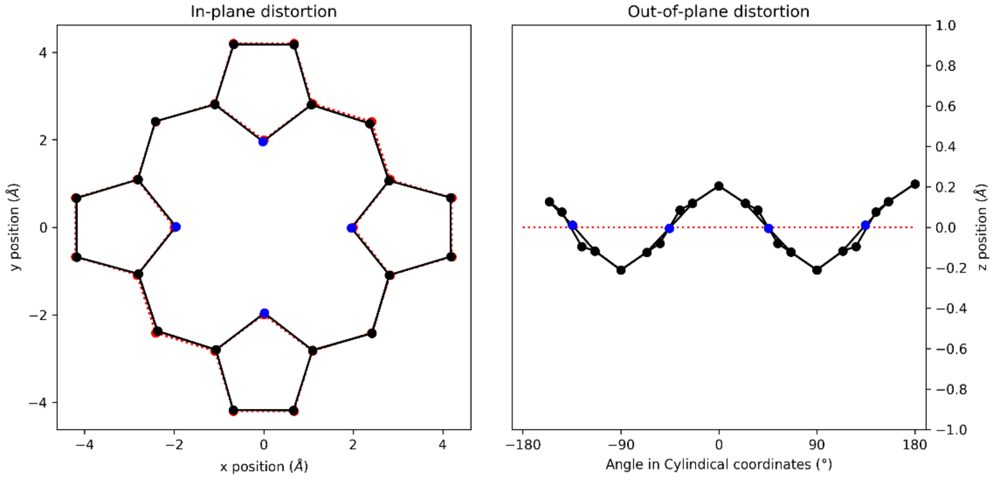

Figure S108: NSD result generated from **1:6** (in Å) (**A**) in-plane and (**B**) out-of-plane skeletal plots of the porphyrin core. Porphyrin is represented in black (C) and blue (N), with the reference structure (CuTPP) in red dotted lines.

basis	Δ_{ip}	δ_{ip}	B_{2g}	B_{1g}	$E_u(x)$	$E_u(y)$	A_{1g}	A_{2g}
min.	0.26	0.00	-0.13	-0.04	-0.02	0.02	0.22	0.00
ext.	0.28	0.00	-0.13	-0.04	-0.02	0.02	0.22	0.00
			-0.01	-0.09	0.01	-0.02	0.00	0.00
total	0.29	0.00	-0.13	-0.04	-0.02	0.02	0.22	0.00
			-0.01	-0.09	0.01	-0.02	0.00	0.00
			-0.01	-0.06	0.00	0.00	0.04	0.00
			0.01	0.00	0.01	-0.01	0.00	0.00
			0.00	0.01	0.00	0.00	0.02	0.00
			-0.01	-0.01	0.00	0.00	0.01	
					0.00	0.00		
					0.00	0.00		
					0.00	0.00		
					0.00	0.00		
					0.00	0.00		
comp.	0.29	0.00	0.14	0.12	0.02	0.03	0.23	0.00

basis	Δ_{oop}	δ_{oop}	B_{2u}	B_{1u}	A_{2u}	$E_g(x)$	$E_g(y)$	A_{1u}
min.	0.08	0.00	0.00	-0.01	0.00	0.06	0.05	0.00
ext.	0.10	0.00	0.00	-0.01	0.00	0.06	0.06	0.00
			0.00	0.00	0.00	0.04	0.02	0.00
total	0.10	0.00	0.00	-0.01	0.00	0.06	0.06	0.00
			0.00	0.00	0.00	0.04	0.02	0.00
			0.00	0.00	0.00	0.00	0.00	
						0.00	0.00	
						0.00	0.00	
comp.	0.10	0.00	0.00	0.01	0.00	0.08	0.06	0.00

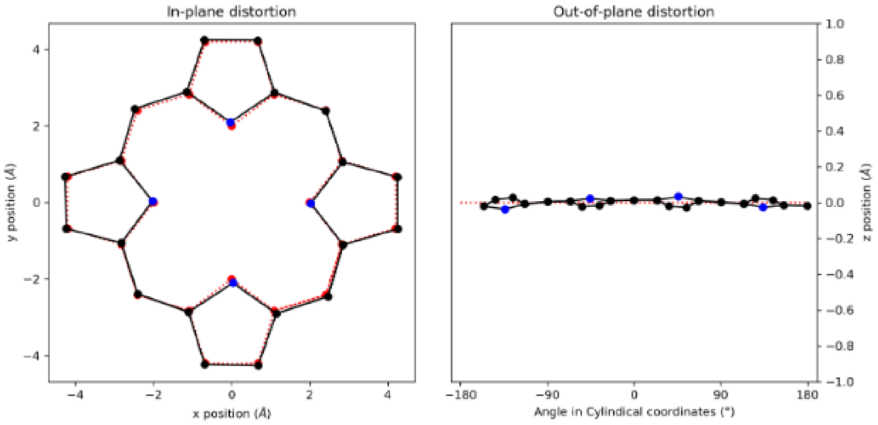

Figure S109: NSD result generated from **1:7** (in Å) (**A**) in-plane and (**B**) out-of-plane skeletal plots of the porphyrin core. Porphyrin is represented in black (C) and blue (N), with the reference structure (CuTPP) in red dotted lines.

basis	Δ_{ip}	δ_{ip}	B_{2g}	B_{1g}	$E_u(x)$	$E_u(y)$	A_{1g}	A_{2g}
min.	0.19	0.00	0.04	0.00	0.02	0.02	-0.18	0.00
ext.	0.19	0.00	0.04	0.00	0.02	0.02	-0.18	0.00
			0.00	0.00	-0.01	-0.01	0.04	0.00
total	0.20	0.00	0.04	0.00	0.02	0.02	-0.18	0.00
			0.00	0.00	-0.01	-0.01	0.04	0.00
			0.01	0.00	-0.01	0.00	-0.02	0.00
			-0.01	0.00	-0.01	-0.01	-0.03	0.00
			0.00	0.00	0.00	0.00	0.00	0.00
			0.00	0.00	0.00	0.00	0.01	
					0.00	0.00		
					0.00	0.00		
					0.00	0.00		
					0.00	0.00		
					0.00	0.00		
comp.	0.20	0.00	0.04	0.00	0.03	0.03	0.19	0.00

basis	Δ_{oop}	δ_{oop}	B_{2u}	B_{1u}	A_{2u}	$E_g(x)$	$E_g(y)$	A_{1u}
min.	0.99	0.00	0.00	-0.99	0.01	-0.01	0.01	0.00
ext.	0.99	0.00	0.00	-0.99	0.01	-0.01	0.01	0.00
			0.00	0.00	0.00	-0.02	0.02	0.00
total	0.99	0.00	0.00	-0.99	0.01	-0.01	0.01	0.00
			0.00	0.00	0.00	-0.02	0.02	0.00
			0.00	-0.02	0.00	0.00	0.00	
						0.00	0.00	
						0.00	0.00	
comp.	0.99	0.00	0.00	0.99	0.01	0.02	0.02	0.00

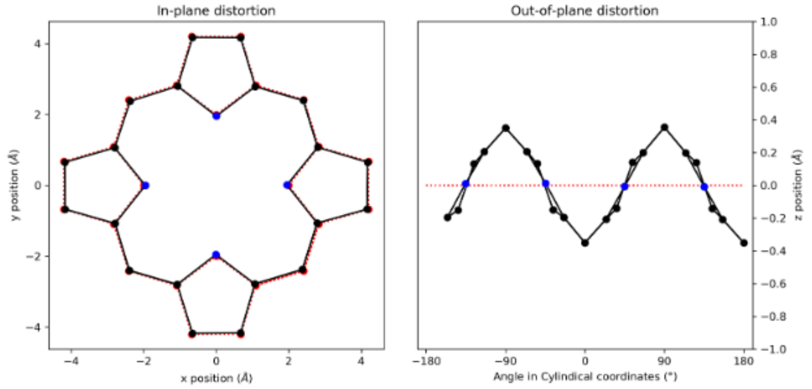

Figure S110: NSD result generated from **1:8** (in Å) (**A**) in-plane and (**B**) out-of-plane skeletal plots of the porphyrin core. Porphyrin is represented in black (C) and blue (N), with the reference structure (CuTPP) in red dotted lines.

**basis**	Δ_{ip}	δ_{ip}	B_{2g}	B_{1g}	$E_u(x)$	$E_u(y)$	A_{1g}	A_{2g}
min.	0.23	0.00	-0.03	0.02	0.00	0.00	0.23	0.00
ext.	0.25	0.00	-0.03	0.02	0.00	0.00	0.23	0.00
			-0.02	0.09	0.00	0.00	0.01	0.00
total	0.26	0.00	-0.04	0.03	0.00	0.00	0.23	0.00
			-0.02	0.09	0.00	0.00	0.01	0.00
			0.02	0.06	0.00	0.00	0.04	0.00
			-0.01	0.00	0.00	0.00	0.00	0.00
			0.00	-0.01	0.00	0.00	0.02	0.00
			0.00	0.01	0.00	0.00	0.01	
					0.00	0.00		
					0.00	0.00		
					0.00	0.00		
					0.00	0.00		
					0.00	0.00		
comp.	0.26	0.00	0.05	0.11	0.00	0.00	0.24	0.00

**basis**	Δ_{oop}	δ_{oop}	B_{2u}	B_{1u}	A_{2u}	$E_g(x)$	$E_g(y)$	A_{1u}
min.	0.07	0.00	0.00	0.00	0.00	-0.04	0.05	0.00
ext.	0.08	0.00	0.00	0.00	0.00	-0.04	0.05	0.00
			0.00	0.00	0.00	-0.02	0.03	0.00
total	0.08	0.00	0.00	0.00	0.00	-0.04	0.05	0.00
			0.00	0.00	0.00	-0.02	0.03	0.00
			0.00	0.00	0.00	0.00	0.00	
						0.00	0.00	
						0.00	0.00	
comp.	0.08	0.00	0.00	0.00	0.00	0.05	0.06	0.00

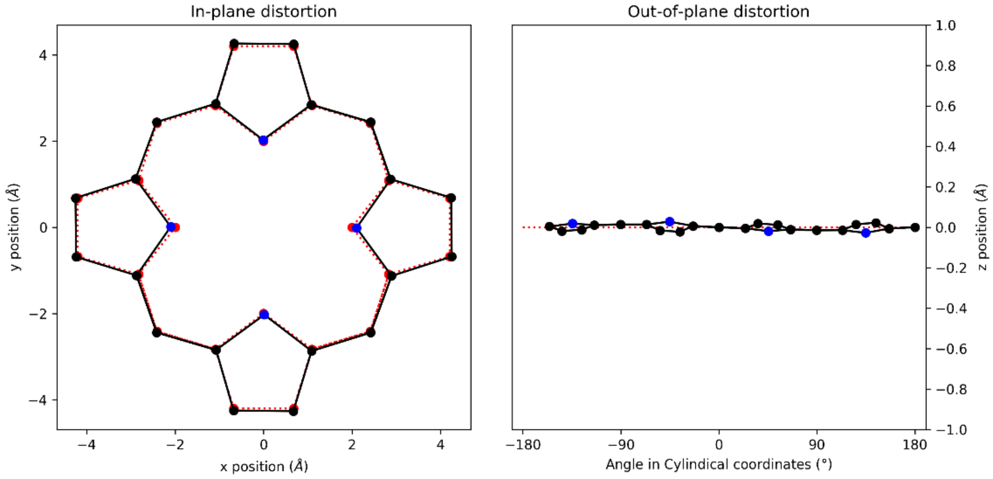

Figure S111: NSD result generated from **1:9** (in Å) (**A**) in-plane and (**B**) out-of-plane skeletal plots of the porphyrin core. Porphyrin is represented in black (C) and blue (N), with the reference structure (CuTPP) in red dotted lines.

basis	Δ_{ip}	δ_{ip}	B_{2g}	B_{1g}	$E_u(x)$	$E_u(y)$	A_{1g}	A_{2g}
min.	0.24	0.00	-0.01	0.00	0.00	0.00	-0.24	0.00
ext.	0.26	0.00	-0.01	0.00	0.00	0.00	-0.24	0.00
			0.01	0.00	0.00	0.00	0.09	0.00
total	0.27	0.00	-0.01	0.00	0.00	0.00	-0.24	0.00
			0.01	0.00	0.00	0.00	0.09	0.00
			-0.02	0.00	0.00	0.00	-0.02	0.00
			0.01	0.00	0.00	0.00	-0.05	0.00
			0.00	0.00	0.00	0.00	0.00	0.00
			0.00	0.00	0.00	0.00	0.02	
					0.00	0.00		
					0.00	0.00		
					0.00	0.00		
					0.00	0.00		
					0.00	0.00		
comp.	0.27	0.00	0.03	0.00	0.01	0.01	0.26	0.00

basis	Δ_{oop}	δ_{oop}	B_{2u}	B_{1u}	A_{2u}	$E_g(x)$	$E_g(y)$	A_{1u}
min.	1.28	0.00	0.00	1.28	0.02	-0.02	-0.02	0.00
ext.	1.28	0.00	0.00	1.28	0.02	-0.02	-0.02	0.00
			0.00	0.00	-0.01	-0.01	-0.01	0.00
total	1.28	0.00	0.00	1.28	0.02	-0.02	-0.02	0.00
			0.00	0.00	-0.01	-0.01	-0.01	0.00
			0.00	0.02	0.00	0.00	0.00	
						0.00	0.00	
						0.00	0.00	
comp.	1.28	0.00	0.00	1.28	0.03	0.02	0.02	0.00

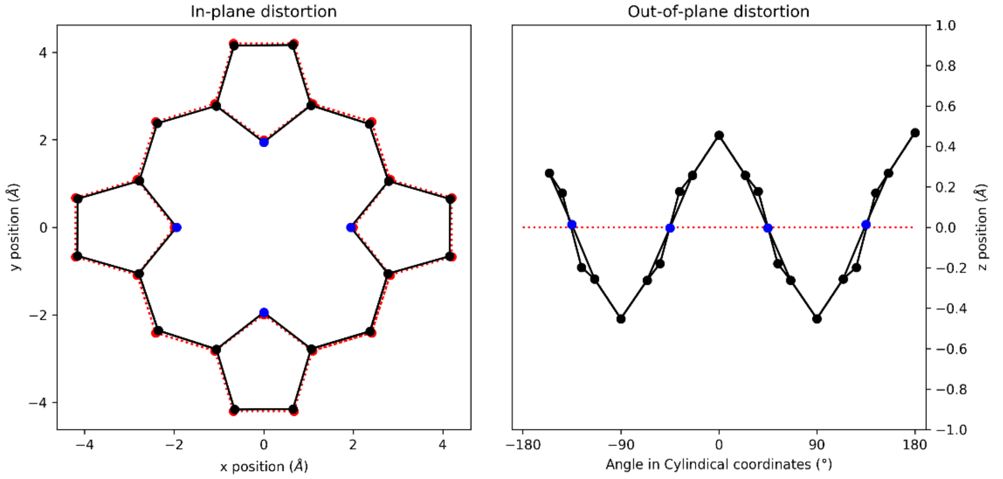

Figure S112: NSD result generated from **1:10** (in Å) (**A**) in-plane and (**B**) out-of-plane skeletal plots of the porphyrin core. Porphyrin is represented in black (C) and blue (N), with the reference structure (CuTPP) in red dotted lines.

basis	Δ_{ip}	δ_{ip}	B_{2g}	B_{1g}	$E_u(x)$	$E_u(y)$	A_{1g}	A_{2g}
min.	0.24	0.00	0.09	0.04	-0.02	-0.03	0.22	0.00
ext.	0.26	0.00	0.09	0.04	-0.02	-0.03	0.22	0.00
			0.00	0.09	0.03	0.01	0.00	0.00
total	0.28	0.00	0.09	0.04	-0.02	-0.03	0.23	0.00
			0.00	0.09	0.03	0.01	0.00	0.00
			0.01	0.06	0.01	0.00	0.04	0.00
			-0.01	0.00	0.01	0.01	0.00	0.00
			0.00	-0.01	0.00	0.00	0.02	0.00
			0.01	0.01	0.00	0.00	0.01	
					0.00	0.00		
					0.00	0.00		
					0.00	0.00		
					0.00	0.00		
					0.00	0.00		
comp.	0.28	0.00	0.09	0.11	0.04	0.03	0.23	0.00

basis	Δ_{oop}	δ_{oop}	B_{2u}	B_{1u}	A_{2u}	$E_g(x)$	$E_g(y)$	A_{1u}
min.	0.08	0.00	0.00	0.01	0.00	-0.05	0.06	0.00
ext.	0.09	0.00	0.00	0.01	0.00	-0.05	0.06	0.00
			0.00	0.00	0.00	-0.02	0.04	0.00
total	0.09	0.00	0.00	0.01	0.00	-0.05	0.06	0.00
			0.00	0.00	0.00	-0.02	0.04	0.00
			0.00	0.00	0.00	0.00	0.00	
						0.00	0.00	
						0.00	0.00	
comp.	0.09	0.00	0.00	0.01	0.00	0.06	0.07	0.00

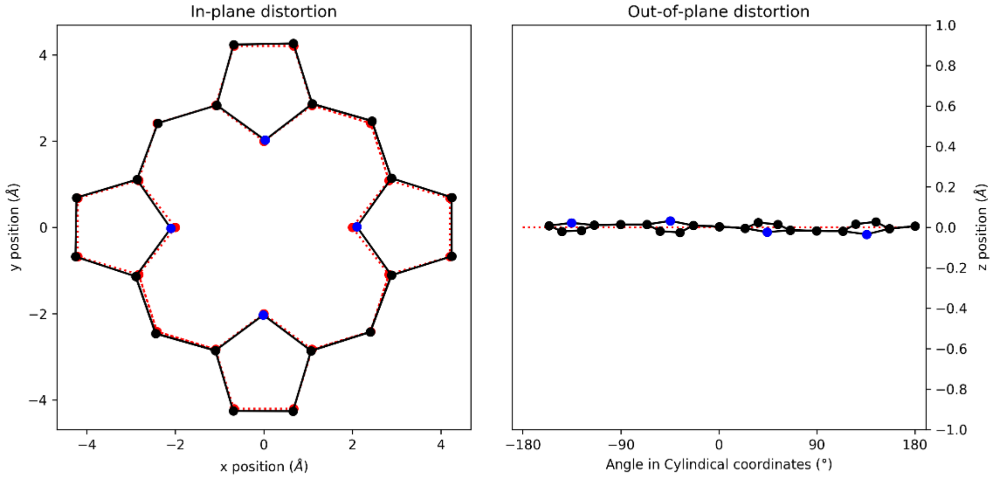

Figure S113: NSD result generated from **1D** (in Å) (**A**) in-plane and (**B**) out-of-plane skeletal plots of the porphyrin core. Porphyrin is represented in black (C) and blue (N), with the reference structure (CuTPP) in red dotted lines.

basis	Δ_{ip}	δ_{ip}	B_{2g}	B_{1g}	$E_u(x)$	$E_u(y)$	A_{1g}	A_{2g}
min.	0.20	0.00	-0.03	0.00	0.02	-0.02	-0.20	0.00
ext.	0.21	0.00	-0.03	0.00	0.02	-0.02	-0.20	0.00
			0.00	0.00	-0.01	0.01	0.06	0.00
total	0.22	0.00	-0.03	0.00	0.02	-0.02	-0.20	0.00
			0.00	0.00	-0.01	0.01	0.06	0.00
			-0.02	0.00	0.00	0.00	-0.02	0.00
			0.01	0.00	-0.01	0.01	-0.04	0.00
			0.00	0.00	0.00	0.00	0.00	0.00
			-0.01	0.00	0.00	0.00	0.02	
					0.00	0.00		
					0.00	0.00		
					0.00	0.00		
					0.00	0.00		
					0.00	0.00		
comp.	0.22	0.00	0.03	0.00	0.02	0.02	0.21	0.00

basis	Δ_{oop}	δ_{oop}	B_{2u}	B_{1u}	A_{2u}	$E_g(x)$	$E_g(y)$	A_{1u}
min.	1.09	0.00	0.00	-1.09	-0.02	0.03	0.03	0.00
ext.	1.09	0.00	0.00	-1.09	-0.02	0.03	0.03	0.00
			0.00	0.00	0.01	0.00	0.00	0.00
total	1.09	0.00	0.00	-1.09	-0.02	0.03	0.03	0.00
			0.00	0.00	0.01	0.00	0.00	0.00
			0.00	-0.02	0.00	0.00	0.00	
						0.00	0.00	
						0.00	0.00	
comp.	1.09	0.00	0.00	1.09	0.02	0.03	0.03	0.00

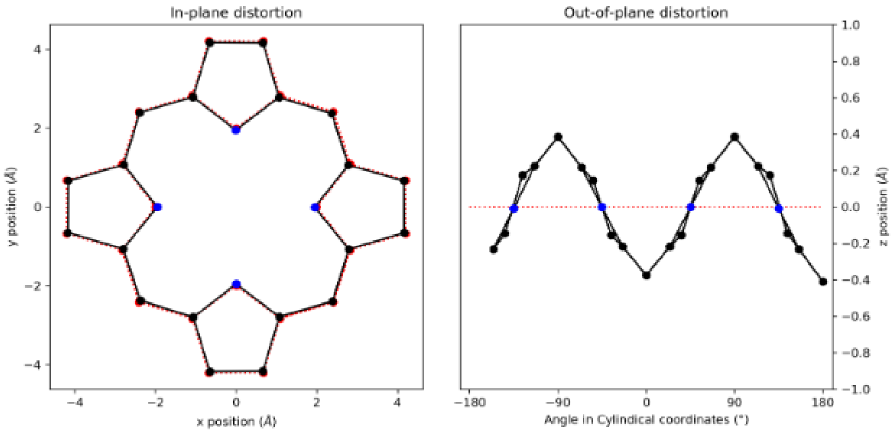

Figure S114: NSD result generated from **2D** (in Å) (**A**) in-plane and (**B**) out-of-plane skeletal plots of the porphyrin core. Porphyrin is represented in black (C) and blue (N), with the reference structure (CuTPP) in red dotted lines.

basis	Δ_{ip}	δ_{ip}	B_{2g}	B_{1g}	$E_u(x)$	$E_u(y)$	A_{1g}	A_{2g}
min.	0.26	0.00	-0.11	0.02	0.00	0.00	0.23	0.00
ext.	0.28	0.00	-0.11	0.02	0.00	0.00	0.23	0.00
			-0.04	0.08	0.00	0.00	0.01	0.00
total	0.29	0.00	-0.11	0.03	0.00	0.00	0.24	0.00
			-0.04	0.09	0.00	0.00	0.01	0.00
			0.02	0.06	0.00	0.00	0.04	0.00
			-0.02	0.00	0.00	0.00	0.00	0.00
			-0.01	-0.01	0.00	0.00	0.02	0.00
			0.00	0.01	0.00	0.00	0.01	
					0.00	0.00		
					0.00	0.00		
					0.00	0.00		
					0.00	0.00		
					0.00	0.00		
comp.	0.29	0.00	0.13	0.11	0.00	0.00	0.24	0.00

basis	Δ_{oop}	δ_{oop}	B_{2u}	B_{1u}	A_{2u}	$E_g(x)$	$E_g(y)$	A_{1u}
min.	0.05	0.00	0.00	0.00	0.00	-0.03	0.04	0.00
ext.	0.06	0.00	0.00	0.00	0.00	-0.03	0.04	0.00
			0.00	0.00	0.00	-0.01	0.02	0.00
total	0.06	0.00	0.00	0.00	0.00	-0.03	0.04	0.00
			0.00	0.00	0.00	-0.01	0.02	0.00
			0.00	0.00	0.00	0.00	0.00	
						0.00	0.00	
						0.00	0.00	
comp.	0.06	0.00	0.00	0.00	0.00	0.04	0.04	0.00

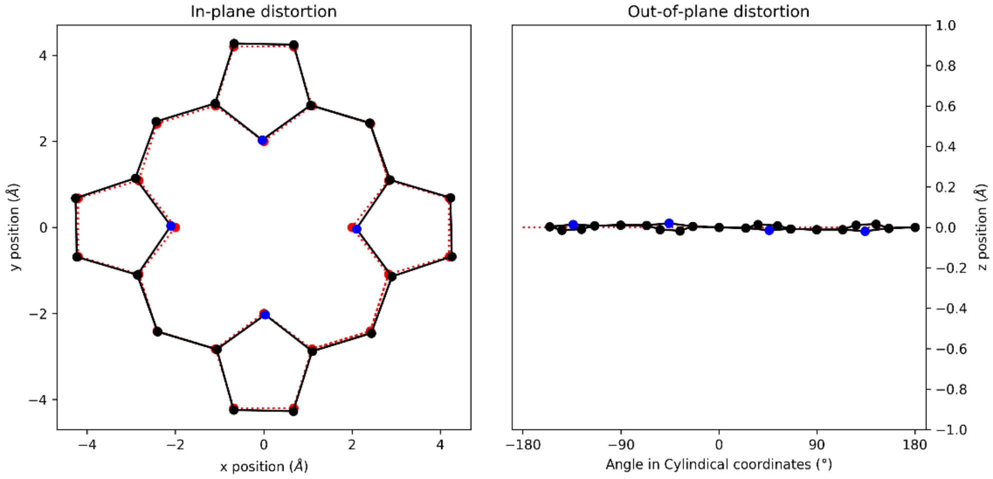

Figure S115: NSD result generated from **1:11** (in Å) (**A**) in-plane and (**B**) out-of-plane skeletal plots of the porphyrin core. Porphyrin is represented in black (C) and blue (N), with the reference structure (CuTPP) in red dotted lines.

basis	Δ_{ip}	δ_{ip}	B_{2g}	B_{1g}	$E_u(x)$	$E_u(y)$	A_{1g}	A_{2g}
min.	0.27	0.00	0.00	0.00	0.01	0.00	-0.27	0.00
ext.	0.29	0.00	0.00	0.00	0.01	0.00	-0.27	0.00
			-0.02	0.00	-0.01	-0.01	0.11	0.00
total	0.30	0.00	0.00	0.00	0.01	0.00	-0.27	0.00
			-0.02	0.00	-0.01	-0.01	0.11	0.00
			0.02	0.00	0.00	0.00	-0.01	0.00
			-0.01	0.00	0.00	0.00	-0.07	0.00
			0.00	0.00	0.00	0.00	0.00	0.00
			0.01	0.00	0.00	0.00	0.03	
					0.00	0.00		
					0.00	0.00		
					0.00	0.00		
					0.00	0.00		
					0.00	0.00		
comp.	0.30	0.00	0.04	0.00	0.01	0.01	0.30	0.00

basis	Δ_{oop}	δ_{oop}	B_{2u}	B_{1u}	A_{2u}	$E_g(x)$	$E_g(y)$	A_{1u}
min.	1.41	0.00	0.00	-1.41	0.03	-0.02	0.02	0.00
ext.	1.41	0.00	0.00	-1.41	0.03	-0.02	0.02	0.00
			0.00	0.00	-0.02	-0.01	0.01	0.00
total	1.41	0.00	0.00	-1.41	0.03	-0.02	0.02	0.00
			0.00	0.00	-0.02	-0.01	0.01	0.00
			0.00	-0.02	0.00	0.00	0.00	
						0.00	0.00	
						0.00	0.00	
comp.	1.41	0.00	0.00	1.41	0.03	0.02	0.02	0.00

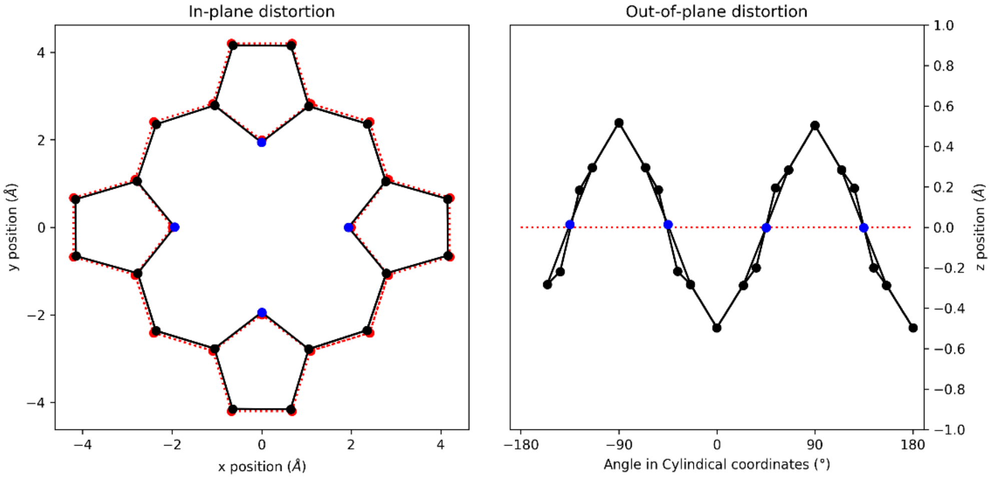

Figure S116: NSD result generated from **1:12** (in Å) (**A**) in-plane and (**B**) out-of-plane skeletal plots of the porphyrin core. Porphyrin is represented in black (C) and blue (N), with the reference structure (CuTPP) in red dotted lines.

**basis**	**Δ_{ip}**	**δ_{ip}**	**B_{2g}**	**B_{1g}**	**$E_u(x)$**	**$E_u(y)$**	**A_{1g}**	**A_{2g}**
min.	0.23	0.00	-0.02	-0.04	0.03	-0.03	0.23	0.00
ext.	0.25	0.00	-0.02	-0.04	0.03	-0.03	0.23	0.00
			0.01	-0.09	-0.02	0.03	0.00	0.00
total	0.27	0.00	-0.02	-0.04	0.03	-0.03	0.23	0.00
			0.01	-0.09	-0.02	0.03	0.00	0.00
			-0.01	-0.06	0.00	0.01	0.04	0.00
			0.01	0.00	0.00	0.00	0.00	0.00
			0.00	0.01	0.00	0.00	0.02	0.00
			-0.01	-0.01	0.00	0.00	0.01	
					0.00	0.00		
					0.00	0.00		
					0.00	0.00		
					0.00	0.00		
					0.00	0.00		
comp.	0.27	0.00	0.03	0.12	0.04	0.04	0.23	0.00

**basis**	**Δ_{oop}**	**δ_{oop}**	**B_{2u}**	**B_{1u}**	**A_{2u}**	**$E_g(x)$**	**$E_g(y)$**	**A_{1u}**
min.	0.07	0.00	0.00	-0.01	0.00	-0.06	-0.05	0.00
ext.	0.09	0.00	0.00	-0.01	0.00	-0.06	-0.05	0.00
			0.00	0.00	0.00	-0.04	-0.02	0.00
total	0.09	0.00	0.00	-0.01	0.00	-0.06	-0.05	0.00
			0.00	0.00	0.00	-0.04	-0.02	0.00
			0.00	0.00	0.00	0.00	0.00	
						0.00	0.00	
						0.00	0.00	
comp.	0.09	0.00	0.00	0.01	0.00	0.07	0.05	0.00

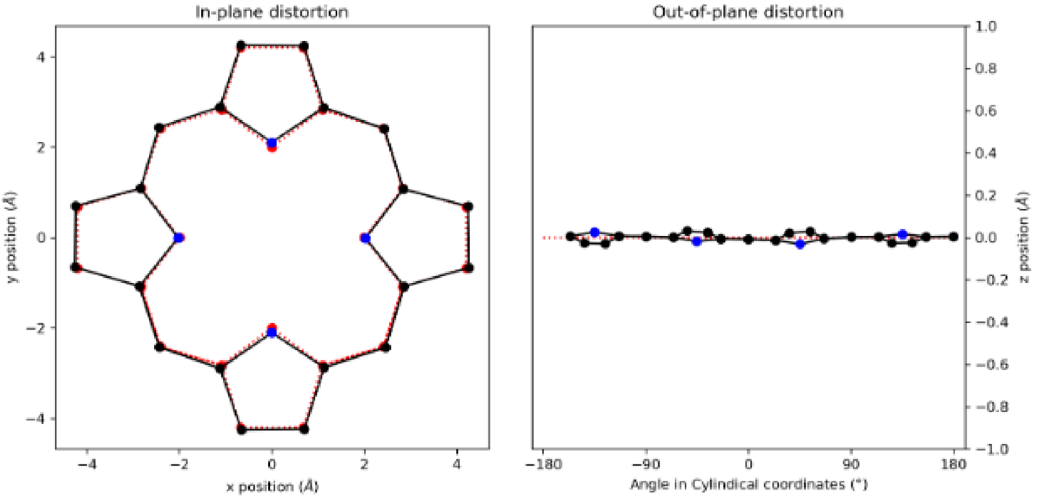

Figure S117: NSD result generated from **3D** (in Å) (**A**) in-plane and (**B**) out-of-plane skeletal plots of the porphyrin core. Porphyrin is represented in black (C) and blue (N), with the reference structure (CuTPP) in red dotted lines.

basis	Δ_{ip}	δ_{ip}	B_{2g}	B_{1g}	$E_u(x)$	$E_u(y)$	A_{1g}	A_{2g}
min.	0.24	0.00	-0.02	0.00	-0.02	0.02	-0.24	0.00
ext.	0.26	0.00	-0.02	0.00	-0.02	0.02	-0.24	0.00
			0.01	0.00	0.02	-0.02	0.08	0.00
total	0.26	0.00	-0.02	0.00	-0.02	0.02	-0.24	0.00
			0.01	0.00	0.02	-0.02	0.08	0.00
			-0.01	0.00	0.01	-0.01	-0.02	0.00
			0.01	0.00	0.01	-0.01	-0.05	0.00
			0.00	0.00	0.00	0.00	0.00	0.00
			-0.01	0.00	0.00	0.00	0.02	
					0.00	0.00		
					0.00	0.00		
					0.00	0.00		
					0.00	0.00		
					0.00	0.00		
comp.	0.26	0.00	0.02	0.00	0.04	0.03	0.26	0.00

basis	Δ_{oop}	δ_{oop}	B_{2u}	B_{1u}	A_{2u}	$E_g(x)$	$E_g(y)$	A_{1u}
min.	1.26	0.00	0.00	1.26	0.03	0.00	0.00	0.00
ext.	1.27	0.00	0.00	1.26	0.03	0.00	0.00	0.00
			0.00	0.00	-0.01	-0.03	-0.03	0.00
total	1.27	0.00	0.00	1.26	0.03	0.00	0.00	0.00
			0.00	0.00	-0.01	-0.03	-0.03	0.00
			0.00	0.02	0.00	0.00	-0.01	
						0.00	0.00	
						0.00	0.00	
comp.	1.27	0.00	0.00	1.26	0.03	0.03	0.03	0.00

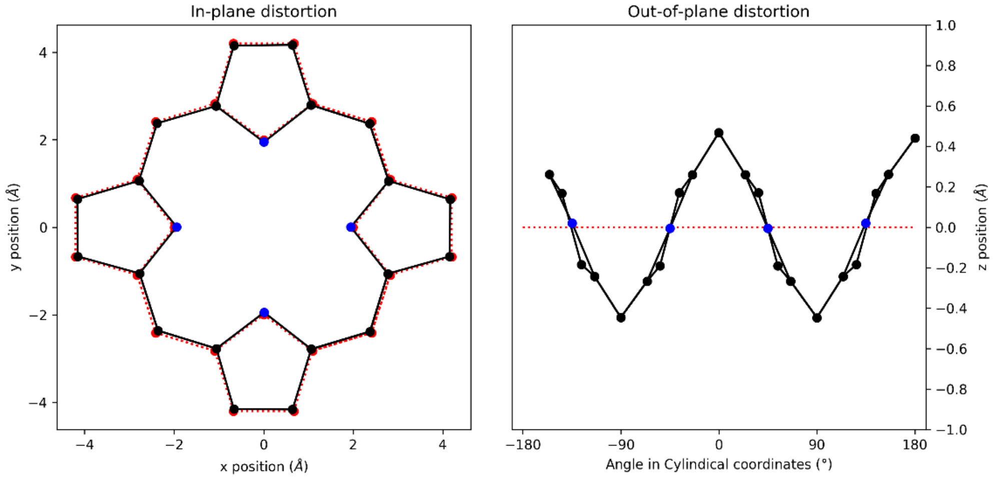

Figure S118: NSD result generated from **1:13** (in Å) (**A**) in-plane and (**B**) out-of-plane skeletal plots of the porphyrin core. Porphyrin is represented in black (C) and blue (N), with the reference structure (CuTPP) in red dotted lines.

basis	Δ_{ip}	δ_{ip}	B_{2g}	B_{1g}	$E_u(x)$	$E_u(y)$	A_{1g}	A_{2g}
min.	0.33	0.00	-0.22	0.02	0.00	0.00	0.24	0.00
ext.	0.35	0.00	-0.22	0.02	0.00	0.00	0.24	0.00
			-0.07	0.08	0.00	0.00	0.00	0.00
total	0.36	0.00	-0.22	0.02	0.00	0.00	0.25	0.00
			-0.07	0.09	0.00	0.00	0.00	0.00
			0.02	0.06	0.00	0.00	0.04	0.00
			-0.02	0.00	0.00	0.00	0.00	0.00
			-0.01	-0.01	0.00	0.00	0.02	0.00
			0.00	0.01	0.00	0.00	0.01	
					0.00	0.00		
					0.00	0.00		
					0.00	0.00		
					0.00	0.00		
					0.00	0.00		
comp.	0.36	0.00	0.24	0.11	0.00	0.00	0.25	0.01

basis	Δ_{oop}	δ_{oop}	B_{2u}	B_{1u}	A_{2u}	$E_g(x)$	$E_g(y)$	A_{1u}
min.	0.02	0.00	0.00	0.00	0.00	-0.01	0.01	0.00
ext.	0.02	0.00	0.00	0.00	0.00	-0.01	0.01	0.00
			0.00	0.00	0.00	0.00	0.01	0.00
total	0.02	0.00	0.00	0.00	0.00	-0.01	0.01	0.00
			0.00	0.00	0.00	0.00	0.01	0.00
			0.00	0.00	0.00	0.00	0.00	
						0.00	0.00	
						0.00	0.00	
comp.	0.02	0.00	0.00	0.00	0.00	0.01	0.01	0.00

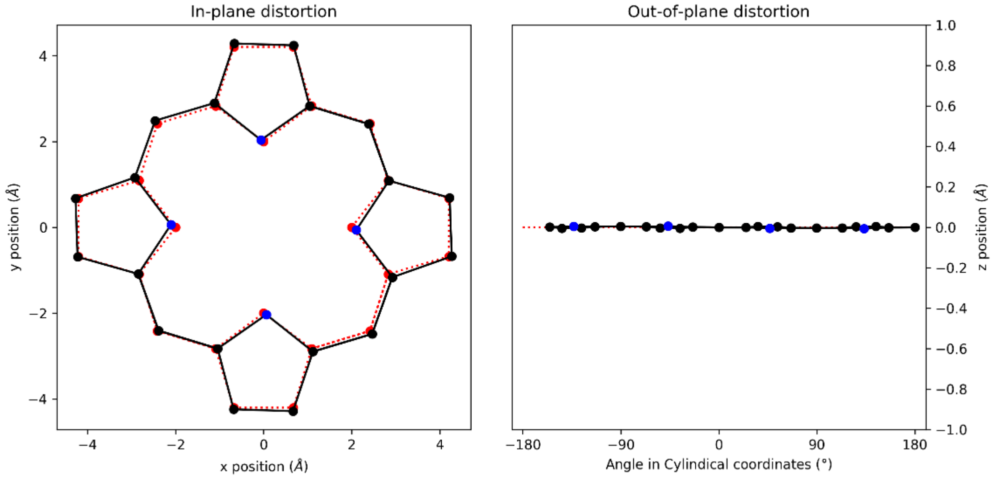

Figure S119: NSD result generated from **1:14** (in Å) (**A**) in-plane and (**B**) out-of-plane skeletal plots of the porphyrin core. Porphyrin is represented in black (C) and blue (N), with the reference structure (CuTPP) in red dotted lines.

basis	Δ_{ip}	δ_{ip}	B_{2g}	B_{1g}	$E_u(x)$	$E_u(y)$	A_{1g}	A_{2g}
min.	0.31	0.01	-0.02	0.00	0.01	0.01	-0.31	0.00
ext.	0.34	0.00	-0.02	0.00	0.01	0.01	-0.31	0.00
			-0.03	0.00	-0.01	-0.01	0.13	0.00
total	0.35	0.00	-0.02	0.00	0.01	0.01	-0.31	0.00
			-0.03	0.00	-0.01	-0.01	0.13	0.00
			0.02	0.00	0.00	0.00	-0.01	0.00
			-0.01	0.00	0.00	0.00	-0.08	0.00
			0.00	0.00	0.00	0.00	0.00	0.00
			0.01	0.00	0.00	0.00	0.04	
					0.00	0.00		
					0.00	0.00		
					0.00	0.00		
					0.00	0.00		
					0.00	0.00		
comp.	0.35	0.00	0.05	0.00	0.01	0.01	0.35	0.00

basis	Δ_{oop}	δ_{oop}	B_{2u}	B_{1u}	A_{2u}	$E_g(x)$	$E_g(y)$	A_{1u}
min.	1.56	0.00	0.00	-1.55	0.04	-0.02	0.02	0.00
ext.	1.56	0.00	0.00	-1.55	0.04	-0.02	0.02	0.00
			0.00	0.00	-0.03	-0.01	0.01	0.00
total	1.56	0.00	0.00	-1.55	0.04	-0.02	0.02	0.00
			0.00	0.00	-0.03	-0.01	0.01	0.00
			0.00	-0.03	0.00	0.00	0.00	
						0.00	0.00	
						0.00	0.00	
comp.	1.56	0.00	0.00	1.56	0.05	0.02	0.02	0.00

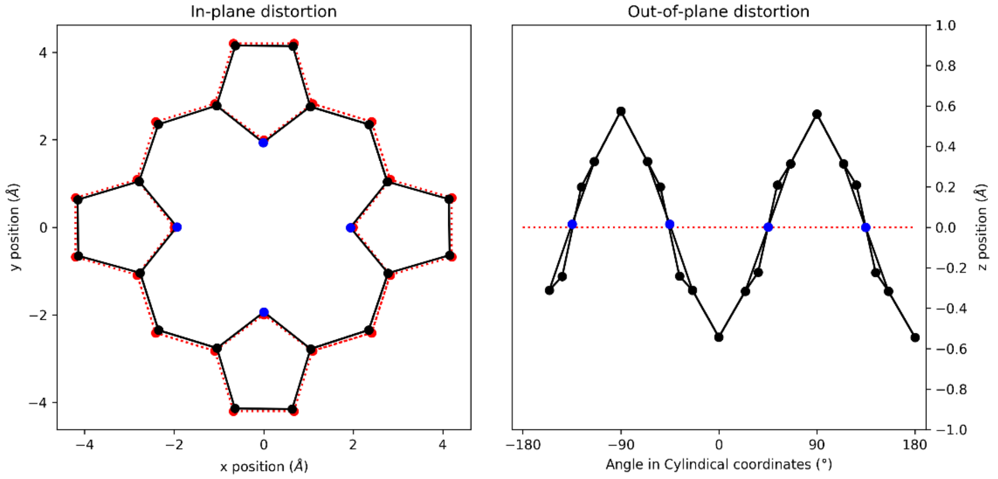

Figure S120: NSD result generated from **1:15** (in Å) (**A**) in-plane and (**B**) out-of-plane skeletal plots of the porphyrin core. Porphyrin is represented in black (C) and blue (N), with the reference structure (CuTPP) in red dotted lines.

NSD tables and plots for DFT structures series 2

basis	Δ_{ip}	δ_{ip}	B_{2g}	B_{1g}	$E_u(x)$	$E_u(y)$	A_{1g}	A_{2g}
min.	0.28	0.00	-0.18	0.06	0.00	-0.01	0.21	0.00
ext.	0.30	0.00	-0.18	0.06	0.00	-0.01	0.21	0.00
			-0.03	0.09	0.02	0.01	0.01	0.00
total	0.31	0.00	-0.18	0.06	0.00	-0.01	0.21	0.00
			-0.03	0.09	0.02	0.01	0.01	0.00
			0.00	0.06	0.00	0.00	0.05	0.00
			0.00	0.00	0.00	0.00	0.01	0.00
			0.00	-0.01	0.00	0.00	0.02	0.00
			0.00	0.01	0.00	0.00	0.01	
					0.00	0.00		
					0.00	0.00		
					0.00	0.00		
					0.00	0.00		
					0.00	0.00		
comp.	0.31	0.00	0.18	0.13	0.02	0.02	0.22	0.00

basis	Δ_{oop}	δ_{oop}	B_{2u}	B_{1u}	A_{2u}	$E_g(x)$	$E_g(y)$	A_{1u}
min.	0.07	0.00	-0.06	-0.01	0.00	-0.03	-0.03	0.00
ext.	0.08	0.00	-0.05	-0.01	0.00	-0.03	-0.03	0.00
			0.02	0.00	0.00	-0.01	-0.02	0.00
total	0.08	0.00	-0.05	-0.01	0.00	-0.03	-0.03	0.00
			0.02	0.00	0.00	-0.01	-0.02	0.00
			0.00	0.00	0.00	0.00	0.00	
						0.00	0.00	
						0.00	0.00	
comp.	0.08	0.00	0.06	0.01	0.00	0.03	0.04	0.00

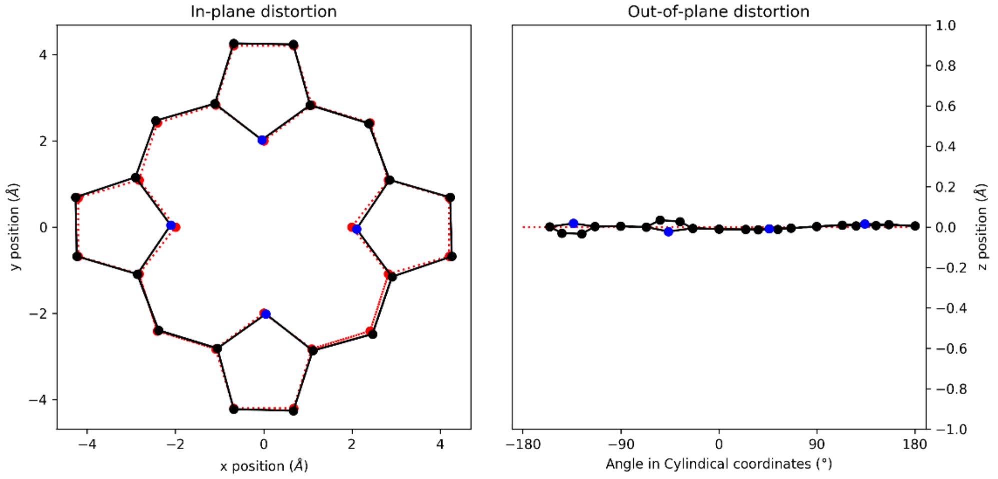

Figure S121: NSD result generated from **2:1** (in Å) (**A**) in-plane and (**B**) out-of-plane skeletal plots of the porphyrin core. Porphyrin is represented in black (C) and blue (N), with the reference structure (CuTPP) in red dotted lines.

basis	Δ_{ip}	δ_{ip}	B_{2g}	B_{1g}	$E_u(x)$	$E_u(y)$	A_{1g}	A_{2g}
min.	0.24	0.00	0.06	0.04	0.02	-0.03	0.22	0.00
ext.	0.25	0.00	0.07	0.04	0.02	-0.03	0.22	0.00
			0.02	0.09	0.00	0.03	0.01	0.00
total	0.27	0.00	0.07	0.05	0.01	-0.03	0.22	0.00
			0.02	0.09	0.00	0.03	0.01	0.00
			-0.01	0.06	-0.01	0.00	0.05	0.00
			0.01	0.00	-0.01	0.00	0.01	0.00
			0.00	-0.01	0.00	0.00	0.02	0.00
			0.00	0.01	0.00	0.00	0.01	
					0.00	0.00		
					0.00	0.00		
					0.00	0.00		
					0.00	0.00		
					0.00	0.00		
comp.	0.27	0.00	0.07	0.12	0.02	0.04	0.23	0.00

basis	Δ_{oop}	δ_{oop}	B_{2u}	B_{1u}	A_{2u}	$E_g(x)$	$E_g(y)$	A_{1u}
min.	0.06	0.00	-0.05	0.00	0.00	-0.02	-0.02	0.00
ext.	0.07	0.00	-0.05	0.00	0.00	-0.03	-0.02	0.00
			0.02	0.00	0.00	-0.01	-0.02	0.00
total	0.07	0.00	-0.05	0.00	0.00	-0.03	-0.02	0.00
			0.02	0.00	0.00	-0.01	-0.02	0.00
			0.00	0.00	0.00	0.00	0.00	
						0.00	0.00	
						0.00	0.00	
comp.	0.07	0.00	0.06	0.00	0.00	0.03	0.03	0.00

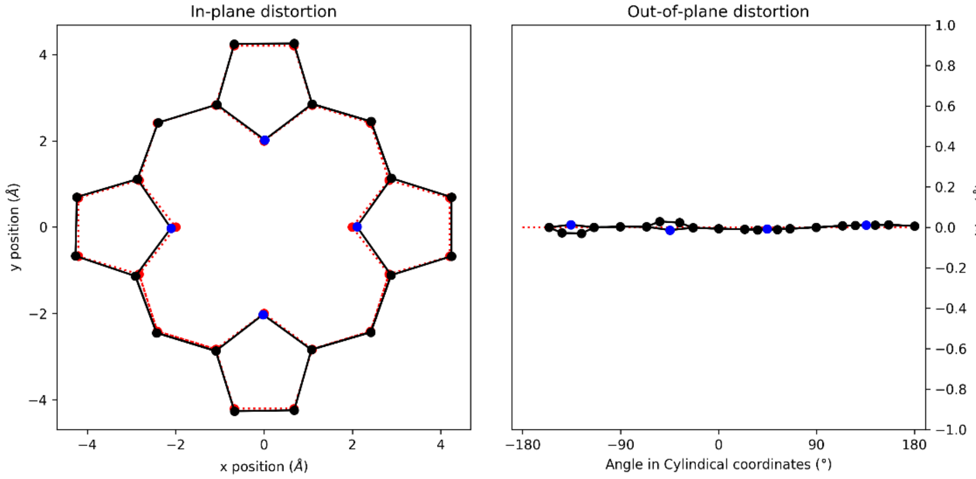

Figure S122: NSD result generated from **2:2** (in Å) (**A**) in-plane and (**B**) out-of-plane skeletal plots of the porphyrin core. Porphyrin is represented in black (C) and blue (N), with the reference structure (CuTPP) in red dotted lines.

basis	Δ_{ip}	δ_{ip}	B_{2g}	B_{1g}	$E_u(x)$	$E_u(y)$	A_{1g}	A_{2g}
min.	0.28	0.00	-0.14	0.03	0.04	-0.01	0.23	0.00
ext.	0.29	0.00	-0.14	0.03	0.04	-0.01	0.23	0.00
			-0.02	0.09	-0.03	0.02	0.02	0.00
total	0.31	0.00	-0.14	0.04	0.04	-0.01	0.24	0.00
			-0.02	0.09	-0.03	0.02	0.02	0.00
			0.00	0.06	-0.01	0.00	0.05	0.00
			0.00	0.00	-0.02	0.00	0.00	0.00
			0.00	-0.01	0.00	0.00	0.02	0.00
			0.00	0.01	0.00	0.00	0.01	
					0.00	0.00		
					0.00	0.00		
					0.00	0.00		
					0.00	0.00		
					0.00	0.00		
comp.	0.31	0.00	0.14	0.11	0.05	0.02	0.24	0.00

basis	Δ_{oop}	δ_{oop}	B_{2u}	B_{1u}	A_{2u}	$E_g(x)$	$E_g(y)$	A_{1u}
min.	0.08	0.00	-0.07	-0.01	0.00	-0.03	-0.03	0.00
ext.	0.09	0.00	-0.07	-0.01	0.00	-0.03	-0.03	0.00
			0.02	0.00	0.00	-0.01	-0.02	0.00
total	0.09	0.00	-0.07	-0.01	0.00	-0.03	-0.03	0.00
			0.02	0.00	0.00	-0.01	-0.02	0.00
			0.00	0.00	0.00	0.00	0.00	
						0.00	0.00	
						0.00	0.00	
comp.	0.09	0.00	0.08	0.01	0.00	0.03	0.03	0.00

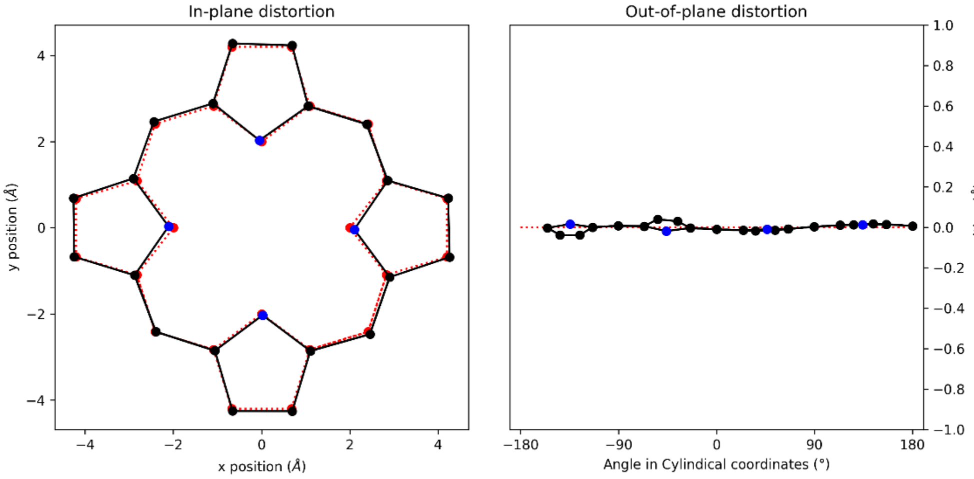

Figure S123: NSD result generated from **2:3** (in Å) (**A**) in-plane and (**B**) out-of-plane skeletal plots of the porphyrin core. Porphyrin is represented in black (C) and blue (N), with the reference structure (CuTPP) in red dotted lines.

basis	Δ_{ip}	δ_{ip}	B_{2g}	B_{1g}	$E_u(x)$	$E_u(y)$	A_{1g}	A_{2g}
min.	0.26	0.00	0.06	0.02	0.02	0.02	0.25	0.00
ext.	0.27	0.00	0.06	0.02	0.02	0.02	0.25	0.00
			0.02	0.09	0.00	0.00	0.02	0.00
total	0.29	0.00	0.06	0.02	0.02	0.02	0.25	0.00
			0.02	0.09	-0.01	0.00	0.02	0.00
			-0.01	0.06	-0.01	0.00	0.05	0.00
			0.01	0.00	-0.01	-0.01	0.00	0.00
			0.00	-0.01	0.00	0.00	0.01	0.00
			0.00	0.01	0.00	0.00	0.01	
					0.00	0.00		
					0.00	0.00		
					0.00	0.00		
					0.00	0.00		
					0.00	0.00		
comp.	0.29	0.00	0.07	0.11	0.02	0.02	0.26	0.00

basis	Δ_{oop}	δ_{oop}	B_{2u}	B_{1u}	A_{2u}	$E_g(x)$	$E_g(y)$	A_{1u}
min.	0.07	0.00	-0.07	0.00	0.00	-0.02	-0.02	0.00
ext.	0.07	0.00	-0.06	0.00	0.00	-0.02	-0.02	0.00
			0.01	0.00	0.00	-0.01	-0.01	0.00
total	0.07	0.00	-0.06	0.00	0.00	-0.02	-0.02	0.00
			0.01	0.00	0.00	-0.01	-0.01	0.00
			0.00	0.00	0.00	0.00	0.00	
						0.00	0.00	
						0.00	0.00	
comp.	0.07	0.00	0.07	0.00	0.00	0.02	0.03	0.00

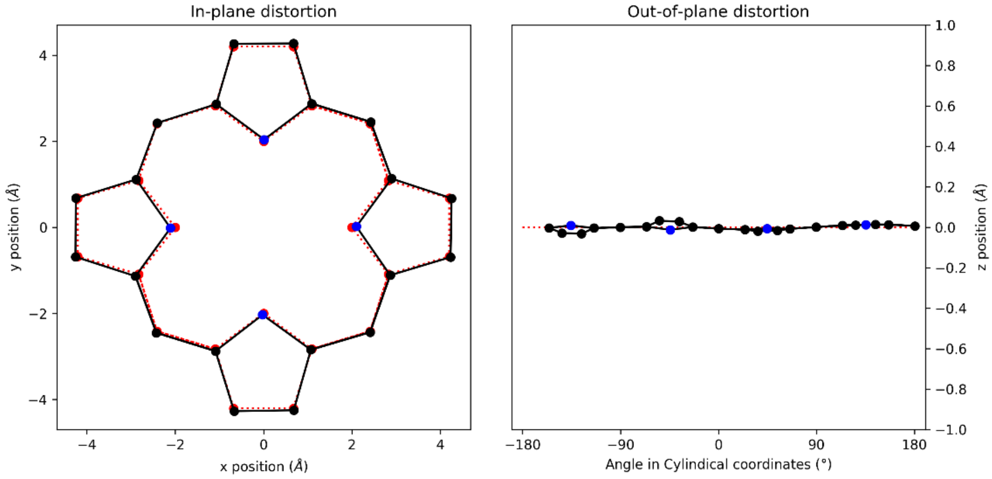

Figure S124: NSD result generated from **2:4** (in Å) (**A**) in-plane and (**B**) out-of-plane skeletal plots of the porphyrin core. Porphyrin is represented in black (C) and blue (N), with the reference structure (CuTPP) in red dotted lines.

basis	Δ_{ip}	δ_{ip}	B_{2g}	B_{1g}	$E_u(x)$	$E_u(y)$	A_{1g}	A_{2g}
min.	0.30	0.00	0.20	0.03	0.01	-0.02	0.22	0.00
ext.	0.32	0.00	0.20	0.03	0.01	-0.02	0.22	0.00
			0.04	0.09	0.02	0.01	0.00	0.00
total	0.33	0.00	0.21	0.04	0.01	-0.02	0.22	0.00
			0.04	0.09	0.02	0.01	0.00	0.00
			-0.01	0.06	-0.01	0.00	0.04	0.00
			0.01	0.00	-0.01	0.01	0.00	0.00
			0.00	-0.01	0.00	0.00	0.02	0.00
			0.00	0.01	-0.01	0.00	0.01	
					0.00	0.00		
					0.00	0.00		
					0.00	0.00		
					0.00	0.00		
					0.00	0.00		
comp.	0.33	0.00	0.21	0.11	0.03	0.03	0.23	0.00

basis	Δ_{oop}	δ_{oop}	B_{2u}	B_{1u}	A_{2u}	$E_g(x)$	$E_g(y)$	A_{1u}
min.	0.14	0.00	-0.12	0.00	0.01	0.00	-0.06	0.00
ext.	0.15	0.00	-0.12	0.00	0.01	0.00	-0.06	0.00
			0.04	0.00	0.00	0.00	-0.04	0.00
total	0.15	0.00	-0.12	0.00	0.01	0.00	-0.06	0.00
			0.04	0.00	0.00	0.00	-0.04	0.00
			0.00	0.00	0.00	0.00	0.00	
						0.00	0.00	
						0.00	0.00	
comp.	0.15	0.00	0.13	0.00	0.01	0.01	0.07	0.00

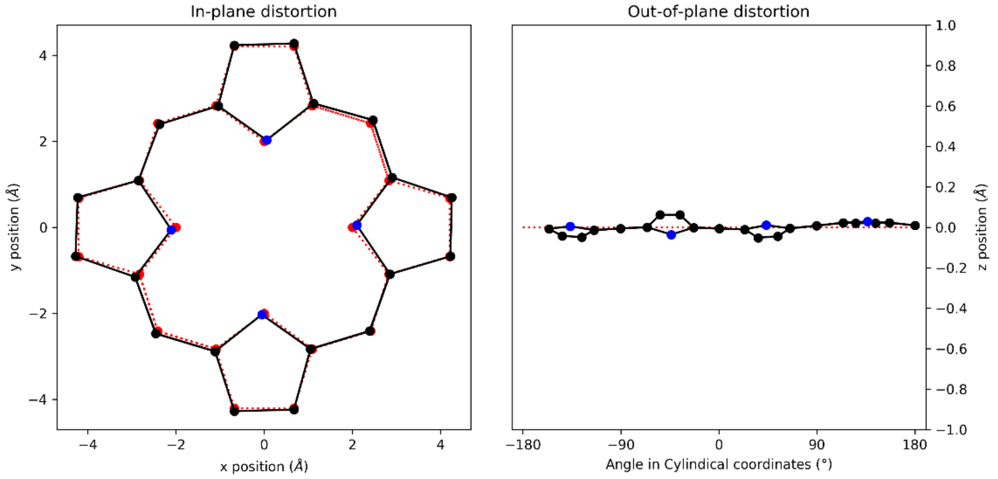

Figure S125: NSD result generated from **2:5** (in Å) (**A**) in-plane and (**B**) out-of-plane skeletal plots of the porphyrin core. Porphyrin is represented in black (C) and blue (N), with the reference structure (CuTPP) in red dotted lines.

basis	Δ_{ip}	δ_{ip}	B_{2g}	B_{1g}	$E_u(x)$	$E_u(y)$	A_{1g}	A_{2g}
min.	0.24	0.00	0.00	0.02	0.03	0.00	0.24	0.00
ext.	0.25	0.00	0.00	0.02	0.03	0.00	0.24	0.00
			0.00	0.08	-0.01	0.00	0.01	0.00
total	0.27	0.00	0.00	0.02	0.03	0.00	0.24	0.00
			0.00	0.09	-0.01	0.00	0.01	0.00
			0.00	0.06	-0.01	0.00	0.04	0.00
			0.00	0.00	-0.02	0.00	0.00	0.00
			0.00	-0.01	0.00	0.00	0.02	0.00
			0.00	0.01	-0.01	0.00	0.01	
					0.00	0.00		
					-0.01	0.00		
					-0.01	0.00		
					0.00	0.00		
					0.01	0.00		
comp.	0.27	0.00	0.00	0.11	0.04	0.00	0.24	0.00

basis	Δ_{oop}	δ_{oop}	B_{2u}	B_{1u}	A_{2u}	$E_g(x)$	$E_g(y)$	A_{1u}
min.	0.17	0.00	-0.16	0.00	0.01	0.00	-0.06	0.00
ext.	0.18	0.00	-0.16	0.00	0.01	0.00	-0.06	0.00
			0.04	0.00	-0.01	0.00	-0.03	0.00
total	0.18	0.00	-0.16	0.00	0.01	0.00	-0.06	0.00
			0.04	0.00	-0.01	0.00	-0.03	0.00
			0.00	0.00	0.00	0.00	0.00	
						0.00	0.00	
						0.00	0.00	
comp.	0.18	0.00	0.17	0.00	0.01	0.00	0.07	0.00

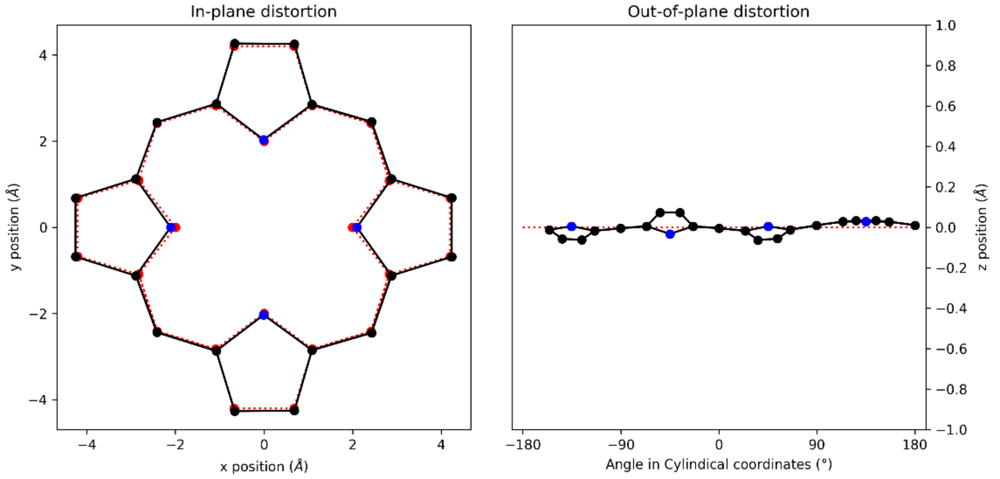

Figure S126: NSD result generated from **2:6** (in Å) (**A**) in-plane and (**B**) out-of-plane skeletal plots of the porphyrin core. Porphyrin is represented in black (C) and blue (N), with the reference structure (CuTPP) in red dotted lines.

NSD tables and plots for DFT structures series 3

basis	Δ_{ip}	δ_{ip}	B_{2g}	B_{1g}	$E_u(x)$	$E_u(y)$	A_{1g}	A_{2g}
min.	0.21	0.00	0.00	0.03	-0.01	0.00	0.21	0.00
ext.	0.23	0.00	0.00	0.04	-0.01	0.00	0.21	0.00
			0.00	0.09	0.03	0.00	0.02	0.00
total	0.26	0.00	0.00	0.04	-0.01	0.00	0.21	0.00
			0.00	0.09	0.03	0.00	0.02	0.00
			0.00	0.06	0.00	0.00	0.04	0.00
			0.00	0.00	-0.04	0.00	0.01	0.00
			0.00	-0.01	0.02	0.00	0.02	0.00
			0.00	0.01	-0.01	0.00	0.00	
					0.00	0.00		
					0.00	0.00		
					-0.02	0.00		
					0.01	0.00		
					-0.03	0.00		
comp.	0.26	0.00	0.00	0.11	0.06	0.00	0.22	0.00

basis	Δ_{oop}	δ_{oop}	B_{2u}	B_{1u}	A_{2u}	$E_g(x)$	$E_g(y)$	A_{1u}
min.	0.13	0.00	-0.11	0.00	0.02	0.00	-0.06	0.00
ext.	0.14	0.00	-0.11	0.00	0.02	0.00	-0.06	0.00
			0.04	0.00	-0.01	0.00	-0.04	0.00
total	0.14	0.00	-0.11	0.00	0.02	0.00	-0.06	0.00
			0.04	0.00	-0.01	0.00	-0.04	0.00
			0.00	0.00	0.00	0.00	0.00	
						0.00	0.00	
						0.00	0.00	
comp.	0.14	0.00	0.11	0.00	0.02	0.00	0.07	0.00

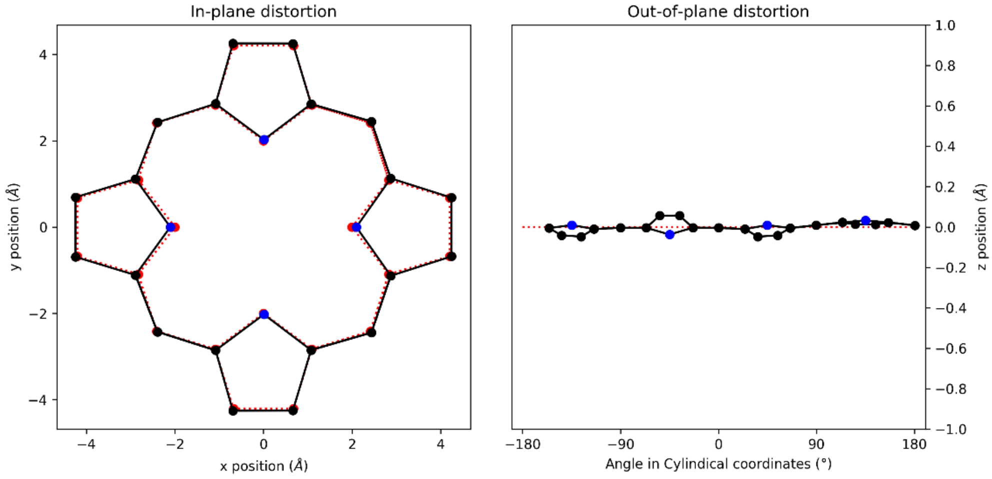

Figure S127: NSD result generated from **3:1** (in Å) (**A**) in-plane and (**B**) out-of-plane skeletal plots of the porphyrin core. Porphyrin is represented in black (C) and blue (N), with the reference structure (CuTPP) in red dotted lines.

basis	Δ_{ip}	δ_{ip}	B_{2g}	B_{1g}	$E_u(x)$	$E_u(y)$	A_{1g}	A_{2g}
min.	0.23	0.00	0.00	0.02	0.02	0.00	0.23	0.00
ext.	0.25	0.00	0.00	0.02	0.02	0.00	0.23	0.00
			0.00	0.09	0.00	0.00	0.01	0.00
total	0.26	0.00	0.00	0.03	0.02	0.00	0.23	0.00
			0.00	0.09	0.00	0.00	0.01	0.00
			0.00	0.06	-0.01	0.00	0.04	0.00
			0.00	0.00	-0.02	0.00	0.00	0.00
			0.00	-0.01	0.01	0.00	0.02	0.00
			0.00	0.01	-0.01	0.00	0.01	
					0.00	0.00		
					0.00	0.00		
					-0.01	0.00		
					0.00	0.00		
					0.00	0.00		
comp.	0.26	0.00	0.00	0.11	0.03	0.00	0.24	0.00

basis	Δ_{oop}	δ_{oop}	B_{2u}	B_{1u}	A_{2u}	$E_g(x)$	$E_g(y)$	A_{1u}
min.	0.15	0.00	-0.14	0.00	0.01	0.00	-0.06	0.00
ext.	0.16	0.00	-0.14	0.00	0.01	0.00	-0.06	0.00
			0.04	0.00	-0.01	0.00	-0.04	0.00
total	0.16	0.00	-0.14	0.00	0.01	0.00	-0.06	0.00
			0.04	0.00	-0.01	0.00	-0.04	0.00
			0.00	0.00	0.00	0.00	0.00	
						0.00	0.00	
						0.00	0.00	
comp.	0.16	0.00	0.15	0.00	0.01	0.00	0.07	0.00

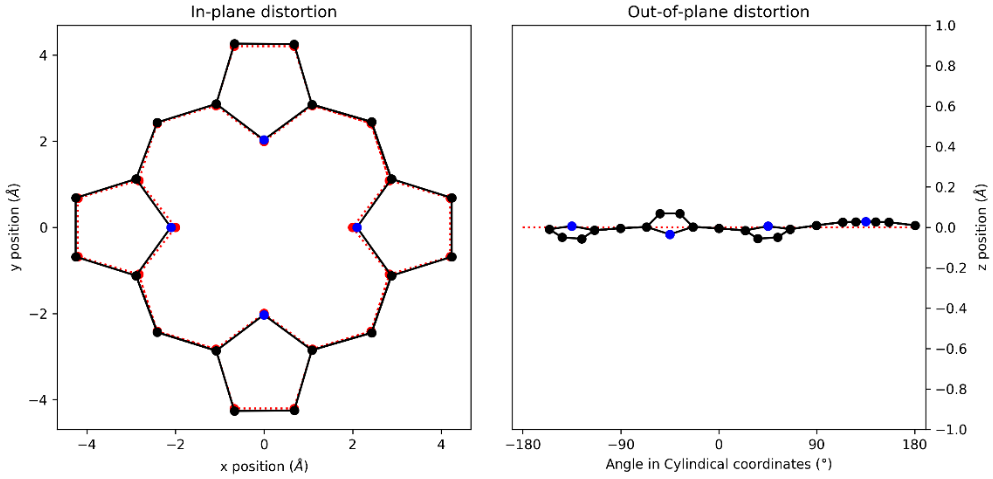

Figure S128: NSD result generated from **3:2** (in Å) (**A**) in-plane and (**B**) out-of-plane skeletal plots of the porphyrin core. Porphyrin is represented in black (C) and blue (N), with the reference structure (CuTPP) in red dotted lines.

basis	Δ_{ip}	δ_{ip}	B_{2g}	B_{1g}	$E_u(x)$	$E_u(y)$	A_{1g}	A_{2g}
min.	0.25	0.00	0.00	0.02	0.05	0.00	0.25	0.00
ext.	0.27	0.00	0.00	0.02	0.05	0.00	0.25	0.00
			0.00	0.09	-0.02	0.00	0.00	0.00
total	0.28	0.00	0.00	0.02	0.05	0.00	0.25	0.00
			0.00	0.09	-0.02	0.00	0.00	0.00
			0.00	0.06	-0.02	0.00	0.04	0.00
			0.00	0.00	-0.01	0.00	0.00	0.00
			0.00	-0.01	0.00	0.00	0.02	0.00
			0.00	0.01	0.00	0.00	0.01	
					0.00	0.00		
					-0.01	0.00		
					0.00	0.00		
					0.00	0.00		
					0.01	0.00		
comp.	0.28	0.00	0.00	0.11	0.06	0.00	0.25	0.00

basis	Δ_{oop}	δ_{oop}	B_{2u}	B_{1u}	A_{2u}	$E_g(x)$	$E_g(y)$	A_{1u}
min.	0.20	0.00	-0.19	0.00	0.01	0.00	-0.05	0.00
ext.	0.20	0.00	-0.19	0.00	0.01	0.00	-0.05	0.00
			0.04	0.00	-0.01	0.00	-0.03	0.00
total	0.20	0.00	-0.19	0.00	0.01	0.00	-0.05	0.00
			0.04	0.00	-0.01	0.00	-0.03	0.00
			0.00	0.00	0.00	0.00	0.00	
						0.00	0.00	
						0.00	0.00	
comp.	0.20	0.00	0.19	0.00	0.01	0.00	0.06	0.00

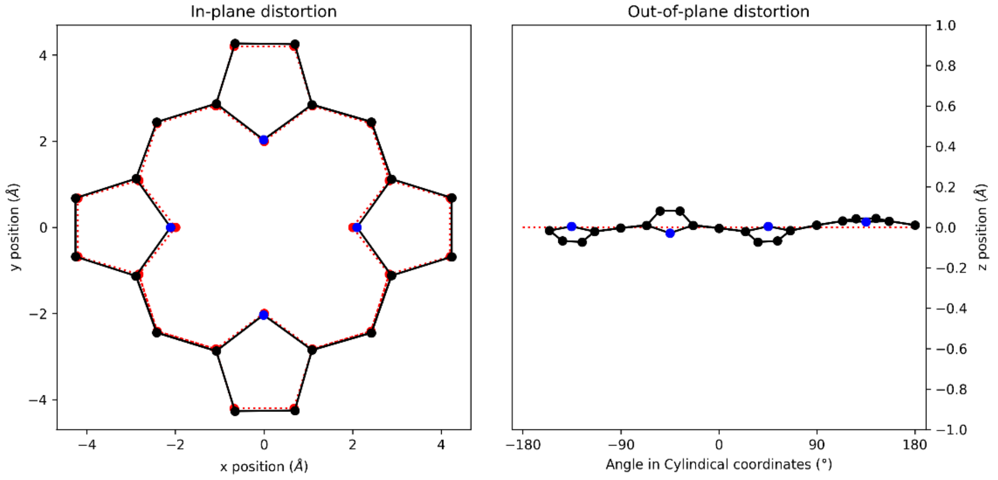

Figure S129: NSD result generated from **3:3** (in Å) (**A**) in-plane and (**B**) out-of-plane skeletal plots of the porphyrin core. Porphyrin is represented in black (C) and blue (N), with the reference structure (CuTPP) in red dotted lines.

NMR and UV spectrum.

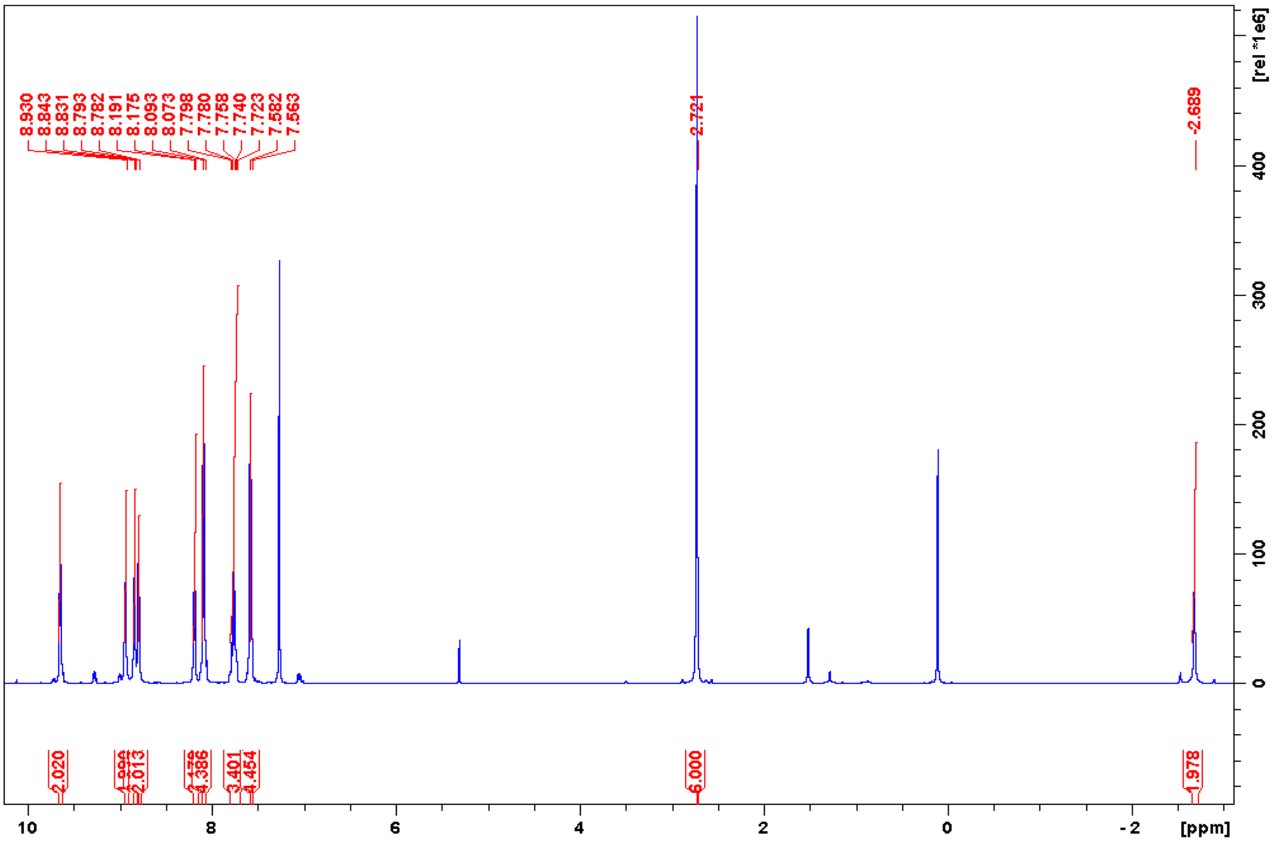

Figure S130: ^{1}H NMR spectrum of compound **4** in $CDCl_3$.

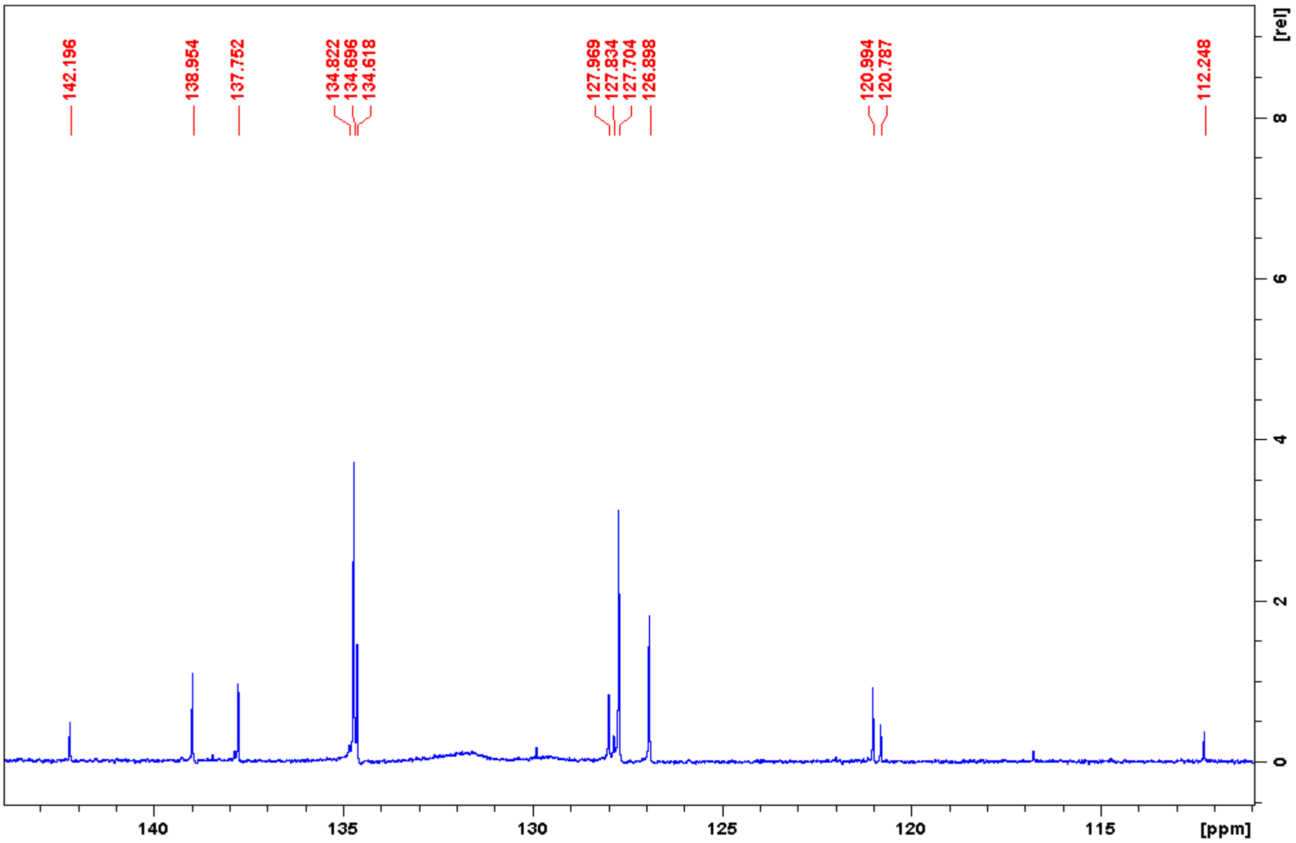

Figure S131: ^{13}C NMR spectrum of compound **4** in $CDCl_3$.

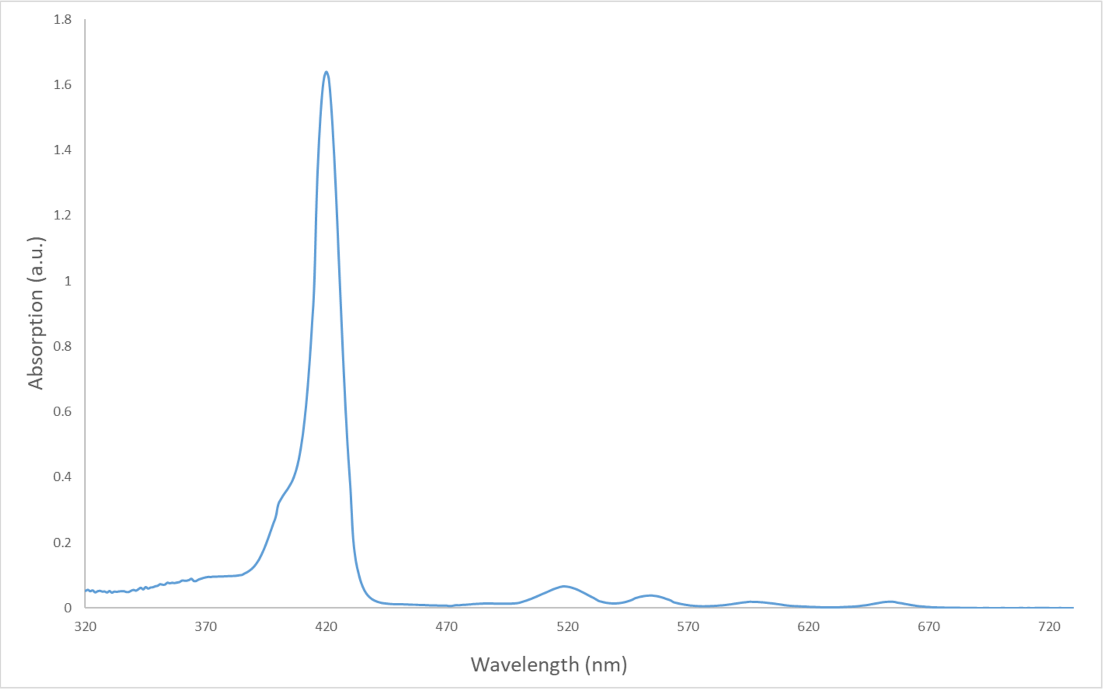

Figure S132: UV-visible spectrum of compound **4** in dichloromethane.
