# Supplementary material for: Structural effects of meso-halogenation on porphyrins
Source: Beilstein J Org Chem. 2021 May 14;17:1149–70. doi: 10.3762/bjoc.17.88 (PMC8144917; doi:10.3762/bjoc.17.88)
Supplement: File 2 — Supplementary tables. [file Beilstein_J_Org_Chem-17-1149-s002.pdf]

BEILSTEIN JOURNAL OF ORGANIC CHEMISTRY

Supporting Information

for

Structural effects of meso-halogenation on porphyrins

Keith J. Flanagan, Maximilian Paradiz Dominguez, Zoi Melissari, Hans-Georg Eckhardt, René M. Williams, Dáire Gibbons, Caroline Prior, Gemma M. Locke, Alina Meindl, Aoife A. Ryan and Mathias O. Senge

Beilstein J. Org. Chem. **2021,** *17,* 1149–1170. doi:10.3762/bjoc.17.88

Supplementary tables

License and Terms: This is a supporting information file under the terms of the Creative Commons Attribution License (https://creativecommons.org/licenses/by/4.0). Please note that the reuse, redistribution and reproduction in particular requires that the author(s) and source are credited and that individual graphics may be subject to special legal provisions.
The license is subject to the *Beilstein Journal of Organic Chemistry* terms and conditions: (https://www.beilstein-journals.org/bjoc/terms)

Table of Contents

5-Halo-substituted porphyrin S3

Bond lengths and angles S3

Table S1: Bond distances, angles, and atom displacement of compounds **1**–**3**.......... S3

5-Halo-15-phenyl-substituted porphyrin S4

Bond lengths and angles S4

Table S2: Bond distances, angles, and atom displacement of compounds **4**–**8**.......... S4

5,15-Dihalo-substituted porphyrin S5

Table S3: List of hydrogen···halogen and halogen···halogen interactions seen in compounds **9–15**. S5

Bond lengths and angles S6

Table S4: Bond distances, angles, and atom displacement of compounds **9**–**12**.......... S6

Table S5: Bond distances, angles, and atom displacement of compounds **13**–**15**.......... S7

5,10-Dihalo-substituted porphyrin S8

Table S6: Bond distances, angles, and atom displacement of compounds **16**–**19**.......... S8

Honourable mentions S9

Table S7: Bond distances, angles, and atom displacement of compounds **20**-**24**. S9

DFT Tables S10

Table S8: Molecular orbital energies (HOMO, LUMO) and singlet state properties (S_1, f_1) together with the porphyrin-phenyl dihedral.......... S10

Table S9: Molecular orbital energies (HOMO, LUMO) and singlet state properties (S_1, f_1) together with the porphyrin-phenyl dihedral.......... S10

Table S10: Molecular orbital energies (HOMO, LUMO) and singlet state properties (S_1, f_1) together with the porphyrin-phenyl dihedral.......... S10

Series 1 .... S11

Table S11: Bond distances, angles, and atom displacement of compounds **1:1**–**1:6**. S11

Table S12: Bond distances, angles, and atom displacement of compounds **1:7**–**1:10, 1D, 2D**.......... S12

Table S13: Bond distances, angles, and atom displacement of compounds **1:11**–**1:15, 3D**.......... S13

Table S14: Comparative tables between crystal structure and calculated structure of compounds **1**-**3** where D signifies the calculated structure. S14

Series 2 .... S15

Table S15: Bond distances, angles, and atom displacement of compounds **2:1**–**2:6, 1D,** and **1:11**. S15

Series 3 .... S16

Table S16: Bond distances, angles, and atom displacement of compounds **3:1**–**3:3, 2:4**. S16

Crystal data and refinement S17

Table S17: Details of XRD data refinement. S17
Table S18: Details of XRD data refinement. S18
Table S19: Details of XRD data refinement. S19
Table S20: Details of XRD data refinement. S20
Complete NSD tables S21
Table S21: NSD out-put of compounds **1**–**23**. S21
Table S22: NSD out-put series 1. .... S22
Table S23: NSD out-put series 2. .... S23
Table S24: NSD out-put series 3. .... S23

5-Halo-substituted porphyrin

Bond lengths and angles

Table S1: Bond distances, angles, and atom displacement of compounds 1–3.

**Bond Distances, Bond Angles, Atom Displacements**	**1**	**2**	**2A**	**3**
CCDC code	MS563	NESHUO	MS546	UDERUR
N–C_a (Å)	1.373(4)	1.384(11)	1.381(3)	1.370(5)
C_a–C_b (Å)	1.437(9)	1.430(17)	1.433(6)	1.441(14)
C_a–C_m (Å)	1.392(5)	1.377(18)	1.382(7)	1.399(8)
C_b–C_b (Å)	1.347(2)	1.326(18)	1.346(3)	1.357(9)
∠$C_aC_bC_b$ (°)	107.6(5)	107.6(9)	107.0(3)	107.3(7)
∠NC_aC_b (°)	108.6(9)	110.0(12)	110.8(4)	108.9(19)
∠NC_aC_m (°)	125.2(10)	125.2(13)	125.7(2)	125.8(7)
∠C_aNC_a (°)	107.6(9)	104.7(8)	104.4(15)	107(2)
∠$C_mC_aC_b$ (°)	126.2(10)	124.7(18)	123.4(4)	125.3(19)
∠$C_aC_mC_a$ (°)	127.0(2)	123(2)	122.8(8)	125(2)
Δ24 (Å)	0.0287	0.1769	0.0396	0.0781
ΔN (Å)	0.023(15)	0.015(12)	0.020(3)	0.030(2)
ΔC_a (Å)	0.022(13)	0.200(2)	0.040(17)	0.050(3)
ΔC_b (Å)	0.033(18)	0.140(4)	0.030(2)	0.150(5)
ΔC_m (Å)	0.040(2)	0.360(3)	0.070(2)	0.050(3)
∠ pyrrole tilt (°)	1.8(4)	11.2(11)	2.4(8)	4.0(2)
N···N dist (adj) (Å)	2.910(4)	2.748(15)	2.770(2)	2.920(7)
N···N dist (opp) (Å)	4.118(15)	3.886(12)	3.917(9)	4.120(7)

5-Halo-15-phenyl-substituted porphyrin

Bond lengths and angles

Table S2: Bond distances, angles, and atom displacement of compounds **4**–**8**

**Bond Distances, Bond Angles, Atom Displacements**	**4**	**5**	**6**	**7**	**8_1**	**8_2**
CCDC code	DGIB005a	MS567	KJF150xp	DGIB007b	TCD662	TCD662
$N–C_a$ (Å)	1.367(2)	1.371(4)	1.371(6)	1.382(4)	1.370(4)	1.373(4)
$C_a–C_b$ (Å)	1.444(4)	1.441(5)	1.441(7)	1.435(5)	1.439(9)	1.436(12)
$C_a–C_m$ (Å)	1.402(7)	1.400(5)	1.399(13)	1.385(6)	1.402(6)	1.401(7)
$C_b–C_b$ (Å)	1.352(4)	1.350(5)	1.3414(8)	1.335(4)	1.344(6)	1.349(8)
$\angle C_aC_bC_b$ (°)	107.3(3)	107.4(2)	107.8(5)	107.2(10)	107.8(3)	107.6(6)
$\angle NC_aC_b$ (°)	108.7(5)	108.7(5)	108.1(9)	110.6(3)	108.1(9)	108.4(8)
$\angle NC_aC_m$ (°)	126.0(8)	125.8(10)	126.1(12)	125.9(11)	126.3(15)	126.0(15)
$\angle C_aNC_a$ (°)	107.9(5)	107.7(6)	108.3(10)	104.3(15)	108.3(13)	107.9(11)
$\angle C_mC_aC_b$ (°)	125.2(7)	125.4(12)	125.8(8)	123.4(9)	125.6(15)	125.6(14)
$\angle C_aC_mC_a$ (°)	125.9(18)	126.0(13)	125.8(14)	122.2(20)	125.6(18)	125.7(17)
Δ24 (Å)	0.0564	0.0688	0.0573	0.0591	0.0553	0.0612
ΔN (Å)	0.070(5)	0.060(6)	0.030(3)	0.020(14)	0.030(3)	0.030(3)
ΔC_a (Å)	0.036(18)	0.060(4)	0.040(3)	0.050(3)	0.040(4)	0.050(3)
ΔC_b (Å)	0.070(3)	0.060(5)	0.080(6)	0.080(6)	0.080(5)	0.080(6)
ΔC_m (Å)	0.050(3)	0.110(4)	0.070(3)	0.070(3)	0.070(4)	0.080(2)
∠ pyrrole tilt (°)	4.2(18)	4.7(15)	3.8(13)	3.2(14)	3.8(12)	4.0(10)
N···N dist (adj) (Å)	2.930(3)	2.918(11)	2.926(9)	2.766(16)	2.930(2)	2.920(3)
N···N dist (opp) (Å)	4.141(7)	4.130(4)	4.140(2)	3.912(4)	4.140(3)	4.130(3)

5,15-Dihalo-substituted porphyrin

Table S3: List of hydrogen···halogen and halogen···halogen interactions seen in compounds **9–15**.

	D···H–A	Distance (Å)	Angle (°)
**9**	Br1···H18–C18	3.071(3)	148.1(1)
	Br1···H204–C204	3.126(3)	125.4(1)
	Br1···H205–C205	3.200(3)	149.3(1)
**10**	Br1···H10C–C107	3.156(5)	141.7(1)
**11**	Br2···Br2–C10	3.433(13)	152.4(11)
	Br1···H20E–C203	3.153(8)	155.4(2)
	Br1···H20F–C204	3.270(5)	127.9(2)
**12**	Br1···H48–C46	3.207(18)	130.7(8)
**13**	Br2···H10I–C105	3.079(3)	125.2(13)
	Br2···H2–C2	3.323(4)	139.7(9)
	Br2···H20A–C207	3.174(4)	139.7(9)
	Br1···H12–C12	3.210(4)	149.5(8)
**13A**	Br2···H6–C8	3.214(4)	149.6(8)
	Br1···H10–C18	3.328(4)	139.7(8)
	Br1···H31–C34	3.181(4)	130.2(11)
	Br1···H20–C25	3.070(4)	125.3(13)
**14**	Br1···H28–C33	3.065(5)	144.1(15)
	Br1···H10–C18	3.453(5)	150.3(17)
	Br2···H6–C8	3.306(5)	157.8(17)
	Br2···H14–C22	3.323(6)	123.6(15)
	Br2···H27–C33	3.129(6)	132.0(13)
**15**	Br1···H23–C35	2.979(3)	158.1(16)
	Br2···H11–C26	2.856(3)	161.5(15)
	Br2···H12–C27	3.064(4)	132.3(15)

Bond lengths and angles

Table S4: Bond distances, angles, and atom displacement of compounds **9**–**12**.

**Bond Distances, Bond Angles, Atom Displacements**	**9**	**10**	**11**	**12**
CCDC code	MS545	HE006	HE015	NOGWEN
N–C_a (Å)	1.375(2)	1.371(9)	1.384(5)	1.360(4)
C_a–C_b (Å)	1.441(9)	1.439(7)	1.433(8)	1.420(3)
C_a–C_m (Å)	1.396(3)	1.399(6)	1.384(7)	1.430(3)
C_b–C_b (Å)	1.352(7)	1.352(3)	1.344(5)	1.320(3)
∠$C_aC_bC_b$ (°)	107.5(6)	107.4(6)	107.4(5)	107.1(19)
∠NC_aC_b (°)	108.8(10)	108.9(13)	109.8(6)	110.5(14)
∠NC_aC_m (°)	125.1(14)	125.2(9)	124.3(11)	124.0(3)
∠C_aNC_a (°)	107.5(12)	107.5(15)	105.5(3)	104.0(2)
∠$C_mC_aC_b$ (°)	126.1(11)	125.9(9)	125.5(8)	125.1(19)
∠$C_aC_mC_a$ (°)	127.2(16)	126.9(19)	122.0(3)	125.9(20)
Δ24 (Å)	0.0174	0.0459	0.3228	0.034
ΔN (Å)	0.029(19)	0.043(14)	0.050(4)	0.022(12)
ΔC_a (Å)	0.012(10)	0.031(15)	0.370(8)	0.026(15)
ΔC_b (Å)	0.016(13)	0.059(18)	0.250(19)	0.040(3)
ΔC_m (Å)	0.019(17)	0.052(8)	0.650(4)	0.041(10)
∠ pyrrole tilt (°)	1.2(4)	3.2(4)	20.8(12)	2.3(8)
N···N dist (adj) (Å)	2.918(7)	2.920(18)	2.700(3)	2.890(2)
N···N dist (opp) (Å)	4.127(13)	4.13(3)	3.822(13)	4.090(2)

Table S5: Bond distances, angles, and atom displacement of compounds **13**–**15**.

**Bond Distances, Bond Angles, Atom Displacements**	**13**	**13A**	**14**	**15**
CCDC code	MS556	HUMWES	HUMWAO	LASMOK
N–C_a (Å)	1.372(3)	1.371(3)	1.37(5)	1.377(4)
C_a–C_b (Å)	1.440(4)	1.442(6)	1.438(6)	1.445(4)
C_a–C_m (Å)	1.398(5)	1.400(4)	1.398(7)	1.398(4)
C_b–C_b (Å)	1.351(17)	1.356(16)	1.347(5)	1.351(3)
$\angle C_aC_bC_b$ (°)	107.4(3)	107.2(3)	107.48(16)	107.1(3)
$\angle NC_aC_b$ (°)	108.8(5)	109.1(6)	108.7(6)	109.8(4)
$\angle NC_aC_m$ (°)	125.0(9)	125.1(9)	125.4(13)	124.5(12)
$\angle C_aNC_a$ (°)	107.5(5)	107.4(7)	107.7(7)	106.16(9)
$\angle C_mC_aC_b$ (°)	126.1(9)	125.8(9)	125.9(12)	125.7(8)
$\angle C_aC_mC_a$ (°)	126.0(2)	126.0(2)	126.0(2)	127.3(15)
Δ24 (Å)	0.155	0.154	0.0582	0.048
ΔN (Å)	0.050(2)	0.050(2)	0.040(3)	0.070(4)
ΔC_a (Å)	0.120(5)	0.120(5)	0.040(3)	0.028(9)
ΔC_b (Å)	0.280(4)	0.280(4)	0.080(6)	0.070(4)
ΔC_m (Å)	0.090(2)	0.090(2)	0.080(3)	0.020(14)
∠ pyrrole tilt (°)	6.8(12)	6.8(12)	3.7(19)	3.7(19)
N···N dist (adj) (Å)	2.910(2)	2.910(2)	2.918(8)	2.895(2)
N···N dist (opp) (Å)	4.110(2)	4.110(2)	4.130(4)	4.094(4)

5,10-Dihalo-substituted porphyrin

Table S6: Bond distances, angles, and atom displacement of compounds **16**–**19**.

**Bond Distances, Bond Angles, Atom Displacements**	**16**	**16A**	**17**	**18**	**19**
CCDC code	RAKGAN	MS529	ZOXQUA	ZOXCAS	BASDOR
$N–C_a$ (Å)	1.375(12)	1.366(6)	1.377(11)	1.374(2)	1.369(2)
$C_a–C_b$ (Å)	1.438(13)	1.438(12)	1.432(14)	1.439(5)	1.442(6)
$C_a–C_m$ (Å)	1.406(10)	1.400(12)	1.388(18)	1.402(7)	1.400(5)
$C_b–C_b$ (Å)	1.341(7)	1.347(11)	1.341(15)	1.341(7)	1.351(7)
$\angle C_aC_bC_b$ (°)	107.8(13)	107.4(7)	107.5(10)	107.4(2)	107.3(3)
$\angle NC_aC_b$ (°)	108.4(14)	108.6(16)	109.5(5)	109.4(3)	108.9(9)
$\angle NC_aC_m$ (°)	125.1(9)	125.9(3)	124.8(16)	124.4(9)	125.3(12)
$\angle C_aNC_a$ (°)	107.6(12)	107.9(19)	106.1(4)	106.4(4)	107.5(13)
$\angle C_mC_aC_b$ (°)	126.3(15)	125.4(14)	125.6(18)	126.1(12)	125.7(13)
$\angle C_aC_mC_a$ (°)	126.5(11)	125.6(10)	125.0(2)	127.0(18)	126.8(18)
Δ24 (Å)	0.159	0.113	0.125	0.106	0.043
ΔN (Å)	0.050(3)	0.040(4)	0.030(18)	0.011(11)	0.003(3)
ΔC_a (Å)	0.140(8)	0.080(5)	0.140(4)	0.110(4)	0.050(7)
ΔC_b (Å)	0.190(9)	0.200(6)	0.090(7)	0.100(8)	0.034(5)
ΔC_m (Å)	0.242(12)	0.080(5)	0.250(3)	0.200(4)	0.090(2)
∠ pyrrole tilt (°)	9.0(14)	5(3)	7.6(5)	6.5(16)	2.7(3)
N···N dist (adj) (Å)	2.920(2)	2.915(16)	2.841(6)	2.890(12)	2.919(2)
N···N dist (opp) (Å)	4.130(4)	4.120(4)	4.017(6)	4.090(2)	4.130(4)

Honourable mentions

Table S7: Bond distances, angles, and atom displacement of compounds **20**–**24**.

**Bond Distances, Bond Angles, Atom Displacements**	**20**	**21**	**22**	**23**	**24**
CCDC code	YISZAD	QUGMEM	QUGMIQ	MORBEC	HE014
N–C_a (Å)	1.368(10)	1.381(4)	1.380(8)	1.390(20)	1.369(5)
C_a–C_b (Å)	1.452(9)	1.440(9)	1.436(7)	1.440(2)	1.438(6)
C_a–C_m (Å)	1.390(5)	1.387(5)	1.388(6)	1.376(17)	1.404(4)
C_b–C_b (Å)	1.366(6)	1.333(9)	1.344(5)	1.340(2)	1.350(4)
∠$C_aC_bC_b$ (°)	106.8(6)	107.5(4)	107.3(11)	107.0(11)	107.5(4)
∠NC_aC_b (°)	109.3(17)	109.5(3)	109.7(4)	111.0(8)	108.6(8)
∠NC_aC_m (°)	124.4(3)	124.7(15)	125.0(14)	124.6(18)	126.4(4)
∠C_aNC_a (°)	107.0(2)	105.8(3)	105.8(3)	104.0(11)	107.9(8)
∠$C_mC_aC_b$ (°)	126.3(16)	125.3(18)	124.9(18)	124.3(14)	125.0(5)
∠$C_aC_mC_a$ (°)	128.8(14)	121.5(12)	121.3(12)	123.4(19)	124.9(8)
Δ24 (Å)	0.023	0.327	0.303	0.166	0.047
ΔN (Å)	0.030(14)	0.032(19)	0.021(18)	0.031(16)	0.023(20)
ΔC_a (Å)	0.014(16)	0.380(5)	0.350(3)	0.190(4)	0.040(3)
ΔC_b (Å)	0.029(16)	0.250(12)	0.240(6)	0.130(7)	0.050(3)
ΔC_m (Å)	0.020(14)	0.660(3)	0.620(7)	0.340(4)	0.070(6)
∠ pyrrole tilt (°)	1.8(6)	20.9(8)	19.3(11)	10.2(7)	2.8(12)
N···N dist (adj) (Å)	2.926(6)	2.704(18)	2.716(15)	2.737(15)	2.910(2)
N···N dist (opp) (Å)	4.140(7)	3.824(8)	3.840(20)	3.869(12)	4.118(13)

DFT Tables

Table S8: Molecular orbital energies (HOMO, LUMO) and singlet state properties (S_1, f_1) together with the porphyrin-phenyl dihedral.

Series 1	HOMO (eV)	LUMO (eV)	S_1 (nm)	f_1	Dihedral (°)
**1:1**	-6.68	-1.08	592	0.0023	69.2
**1:2**	-6.75	-0.90	520	0.0012	82.6
**1:3**	-6.68	-1.18	585	0.0202	69.1
**1:4**	-6.85	-1.00	522	0.0050	81.9
**1:5**	-6.64	-1.32	612	0.0465	68.9
**1:6**	-6.82	-1.14	532	0.0304	81.6
**1:7**	-6.76	-1.24	603	0.0023	72.8
**1:8**	-6.91	-1.08	529	0.0003	81.6
**1:9**	-6.83	-1.40	614	0.0059	78.0
**1:10**	-6.96	-1.24	539	0.0033	81.4
**1D**	-6.77	-1.24	603	0.0018	74.1
**2D**	-6.90	-1.09	530	0.0009	82.8
**1:11**	-6.84	-1.08	614	0.0071	81.4
**1:12**	-6.96	-1.27	541	0.0061	81.5
**3D**	-6.77	-1.24	602	0.0020	75.8
**1:13**	-6.89	-1.09	532	0.0004	80.2
**1:14**	-6.83	-1.18	614	0.0116	87.6
**1:15**	-6.94	-1.28	545	0.0115	81.5

S_1=first singlet state; f_1=oscillator strength

Table S9: Molecular orbital energies (HOMO, LUMO) and singlet state properties (S_1, f_1) together with the porphyrin-phenyl dihedral.

Series 2	HOMO (eV)	LUMO (eV)	S_1 (nm)	f_1	Dihedral (°)
**2:1**	-6.76	-1.08	585	0.0006	71.4
**2:2**	-6.84	-1.25	595	0.0007	78.3
**1D**	-6.77	-1.24	603	0.0018	74.1
**1:11**	-6.84	-1.08	614	0.0071	81.4
**2:3**	-6.90	-1.41	607	0.0024	73.9
**2:4**	-6.97	-1.57	620	0.0065	80.1
**2:5**	-6.77	-1.35	601	0.0037	79.3
**2:6**	-6.83	-1.38	612	0.0051	75.6

S_1=first singlet state; f_1=oscillator strength

Table S10: Molecular orbital energies (HOMO, LUMO) and singlet state properties (S_1, f_1) together with the porphyrin-phenyl dihedral..

Series 3	HOMO (eV)	LUMO (eV)	S_1 (nm)	f_1	Dihedral (°)
**3:1**	-6.82	-1.37	566	0.0171	74.2
**3:2**	-6.69	-1.17	614	0.0060	75.4
**2:6**	-6.83	-1.38	612	0.0051	75.6
**3:3**	-6.83	-1.37	608	0.0048	75.8

S_1=first singlet state; f_1=oscillator strength

Series 1

Table S11: Bond distances, angles, and atom displacement of compounds 1:1–1:6.

**Bond Distances, Bond Angles, Atom Displacements**	**1:1**	**1:2**	**1:3**	**1:4**	**1:5**	**1:6**
CCDC code	Base	Base-Ni	F-Mono	F-Mono-Ni	F-Di	F-Di-Ni
N–C_a (Å)	1.363(4)	1.374(13)	1.363(7)	1.374(4)	1.363(4)	1.374(14)
C_a–C_b (Å)	1.446(13)	1.44(2)	1.445(15)	1.438(5)	1.443(13)	1.436(4)
C_a–C_m (Å)	1.398(6)	1.384(5)	1.399(11)	1.384(7)	1.398(6)	1.383(5)
C_b–C_b (Å)	1.361(8)	1.355	1.361(8)	1.356(3)	1.362(8)	1.3566(2)
∠$C_aC_bC_b$ (°)	107.0(9)	106.556(17)	106.9(9)	106.5(3)	106.8(9)	106.4(5)
∠NC_aC_b (°)	109(2)	111.07(15)	109(2)	111.2(4)	109(2)	111.4(5)
∠NC_aC_m (°)	126.0(8)	125.72(12)	125.7(8)	125.3(7)	125.3(4)	125.0(8)
∠C_aNC_a (°)	107(3)	104.7417(11)	107(3)	104.5(3)	107(3)	104.267(14)
∠$C_mC_aC_b$ (°)	124(2)	123.16(7)	125(2)	123.4(4)	125(2)	123.6(2)
∠$C_aC_mC_a$ (°)	126(2)	122.9(10)	126(3)	123.4(17)	126(3)	123.9(20)
Δ24 (Å)	0.0185	0.1003	0.0194	0.1084	0.02	0.1049
ΔN (Å)	0.033(5)	0.007(2)	0.034(6)	0.008(3)	0.035(6)	0.007(3)
ΔC_a (Å)	0.013(4)	0.116(4)	0.013(4)	0.125(4)	0.014(4)	0.122(4)
ΔC_b (Å)	0.022(4)	0.081(6)	0.023(5)	0.087(8)	0.024(5)	0.084(7)
ΔC_m (Å)	0.009(6)	0.202(3)	0.01(6)	0.217(5)	0.01(7)	0.211(4)
∠ pyrrole tilt (°)	1.46(18)	6.33(6)	1.5(2)	6.84(13)	1.6(2)	6.64(8)
N···N dist (adj) (Å)	2.92(12)	2.77(2)	2.92(11)	2.77(2)	2.92(11)	2.77(2)
N···N dist (opp) (Å)	4.13(8)	3.92	4.13(8)	3.92	4.13(7)	3.9216(4)

Table S12: Bond distances, angles, and atom displacement of compounds 1:7–1:10, 1D, 2D.

**Bond Distances, Bond Angles, Atom Displacements**	**1:7**	**1:8**	**1:9**	**1:10**	**1D**	**2D**
CCDC code	Cl-Mono	Cl-Mono-Ni	Cl-Di	Cl-Di-Ni	Br-Mono	Br-Mono-Ni
N–C_a (Å)	1.364(4)	1.3739(15)	1.364(4)	1.3741(3)	1.364(4)	1.374(2)
C_a–C_b (Å)	1.446(14)	1.439(2)	1.445(14)	1.4384(7)	1.446(13)	1.4395(16)
C_a–C_m (Å)	1.399(5)	1.387(4)	1.401(3)	1.3887(9)	1.4(5)	1.387(4)
C_b–C_b (Å)	1.36(8)	1.3549(3)	1.359(8)	1.35509(16)	1.359(8)	1.3544(10)
$\angle C_aC_bC_b$ (°)	107.0(9)	106.58(15)	107.0(9)	106.6(2)	107.0(9)	106.62(12)
$\angle NC_aC_b$ (°)	109(2)	111.01(14)	109(2)	110.9(17)	109(2)	110.89(12)
$\angle NC_aC_m$ (°)	125.7(6)	125.2(7)	125.5(11)	124.8(9)	125.8(7)	125.2(7)
$\angle C_aNC_a$ (°)	107(3)	104.78(6)	107(3)	104.897(12)	107(3)	104.93(3)
$\angle C_mC_aC_b$ (°)	125(2)	123.6(6)	125(2)	124.1(7)	125(2)	123.7(7)
$\angle C_aC_mC_a$ (°)	126.3(17)	122.9(11)	126.6(9)	123.0(12)	126.3(15)	122.8(11)
Δ24 (Å)	0.0175	0.1744	0.0137	0.2249	0.0167	0.1914
ΔN (Å)	0.03(6)	0.01(3)	0.023(4)	0.008(6)	0.028(5)	0.004(4)
ΔC_a (Å)	0.011(4)	0.202(5)	0.009(4)	0.261(5)	0.011(4)	0.223(6)
ΔC_b (Å)	0.021(5)	0.141(6)	0.017(4)	0.181(11)	0.02(5)	0.155(13)
ΔC_m (Å)	0.01(6)	0.3518(18)	0.007(7)	0.457(7)	0.01(6)	0.389(14)
∠ pyrrole tilt (°)	1.4(2)	11.05(2)	1.09(18)	14.33(13)	1.3(2)	12.2(3)
N···N dist (adj) (Å)	2.92(5)	2.763(9)	2.92(20)	2.752(2)	2.92(3)	2.76(6)
N···N dist (opp) (Å)	4.13(8)	3.91(1)	4.13(7)	3.89(1)	4.13(8)	3.90(1)

Table S13: Bond distances, angles, and atom displacement of compounds **1:11–1:15**, **3D**.

**Bond Distances, Bond Angles, Atom Displacements**	**1:11**	**1:12**	**3D**	**1:13**	**1:14**	**1:15**
CCDC code	Br-Di	Br-Di-Ni	I-Mono	I-Mono-Ni	I-Di	I-Di-Ni
N–C_a (Å)	1.365(4)	1.3737(11)	1.365(5)	1.374(2)	1.366(4)	1.3742(19)
C_a–C_b (Å)	1.446(13)	1.4388(2)	1.446(14)	1.4398(18)	1.446(13)	1.4398(13)
C_a–C_m (Å)	1.401(3)	1.39(3)	1.4(6)	1.388(4)	1.402(3)	1.3914(8)
C_b–C_b (Å)	1.358(8)	1.3552(5)	1.359(8)	1.3549(14)	1.357(8)	1.3544(7)
$\angle C_aC_bC_b$ (°)	107.0(9)	106.66(9)	107.0(9)	106.66(13)	107.1(9)	106.75(5)
$\angle NC_aC_b$ (°)	109(2)	110.75(5)	109(2)	110.75(20)	109(2)	110.51(15)
$\angle NC_aC_m$ (°)	125.6(14)	124.8(9)	125.8(9)	125.2(7)	125.7(18)	124.8(9)
$\angle C_aNC_a$ (°)	107(3)	105.0935(18)	107(3)	105.12(7)	107(3)	105.36(2)
$\angle C_mC_aC_b$ (°)	125(2)	124.2(9)	125(2)	123.8(9)	125(3)	124.4(11)
$\angle C_aC_mC_a$ (°)	126.6(5)	122.8(10)	126.2(10)	122.6(8)	126.4(3)	122.4(7)
Δ24 (Å)	0.0102	0.2476	0.0155	0.2226	0.0032	0.2724
ΔN (Å)	0.017(3)	0.008(7)	0.026(5)	0.012(8)	0.0051(10)	0.009(8)
ΔC_a (Å)	0.006(3)	0.287(5)	0.01(4)	0.258(9)	0.0019(10)	0.316(5)
ΔC_b (Å)	0.013(3)	0.199(13)	0.019(5)	0.179(9)	0.0041(8)	0.219(16)
ΔC_m (Å)	0.006(5)	0.504(9)	0.009(6)	0.45(11)	0.002(17)	0.556(14)
∠ pyrrole tilt (°)	0.8(12)	15.8(13)	1.21(19)	14.1(3)	0.25(3)	17.41(14)
N···N dist (adj) (Å)	2.92(5)	2.747(7)	2.921(8)	2.753(6)	2.93(9)	2.74(12)
N···N dist (opp) (Å)	4.13(7)	3.89(1)	4.13(8)	3.89(1)	4.14(7)	3.875(3)

Table S14: Comparative tables between crystal structure and calculated structure of compounds **1**–**3** where D signifies the calculated structure.

**Bond Distances, Bond Angles, Atom Displacements**	**1**	**1D**	**2**	**2D**	**3**	**3D**
	MS563	Br-Mono	NESHUO	Br-Mono-Ni	UDERUR	I-Mono
N–C_a (Å)	1.373(4)	1.364(4)	1.384(11)	1.374(2)	1.370(5)	1.365(5)
C_a–C_b (Å)	1.437(9)	1.446(13)	1.430(17)	1.4395(16)	1.441(14)	1.446(14)
C_a–C_m (Å)	1.392(5)	1.4(5)	1.377(18)	1.387(4)	1.399(8)	1.4(6)
C_b–C_b (Å)	1.347(2)	1.359(8)	1.326(18)	1.3544(10)	1.357(9)	1.359(8)
∠$C_aC_bC_b$ (°)	107.6(5)	107.0(9)	107.6(9)	106.62(12)	107.3(7)	107.0(9)
∠NC_aC_b (°)	108.6(9)	109(2)	110.0(12)	110.89(12)	108.9(19)	109(2)
∠NC_aC_m (°)	125.2(10)	125.8(7)	125.2(13)	125.2(7)	125.8(7)	125.8(9)
∠C_aNC_a (°)	107.6(9)	107(3)	104.7(8)	104.93(3)	107(2)	107(3)
∠$C_mC_aC_b$ (°)	126.2(10)	125(2)	124.7(18)	123.7(7)	125.3(19)	125(2)
∠$C_aC_mC_a$ (°)	127.0(2)	126.3(15)	123(2)	122.8(11)	125(2)	126.2(10)
Δ24 (Å)	0.0287	0.0167	0.1769	0.1914	0.0781	0.0155
ΔN (Å)	0.023(15)	0.028(5)	0.015(12)	0.004(4)	0.030(2)	0.026(5)
ΔC_a (Å)	0.022(13)	0.011(4)	0.200(2)	0.223(6)	0.050(3)	0.01(4)
ΔC_b (Å)	0.033(18)	0.02(5)	0.140(4)	0.155(13)	0.150(5)	0.019(5)
ΔC_m (Å)	0.040(2)	0.01(6)	0.360(3)	0.389(14)	0.050(3)	0.009(6)
∠ pyrrole tilt (°)	1.8(4)	1.3(2)	11.2(11)	12.2(3)	4.0(2)	1.21(19)
N···N dist (adj) (Å)	2.910(4)	2.92(3)	2.748(15)	2.76(6)	2.920(7)	2.921(8)
N···N dist (opp) (Å)	4.118(15)	4.13(8)	3.886(12)	3.90(1)	4.120(7)	4.13(8)

Series 2

Table S15: Bond distances, angles, and atom displacement of compounds 2:1–2:6, 1D, and 1:11.

Bond Distances, Bond Angles, Atom Displacements	2:1	2:2	2:3	2:4	2:5	2:6	1D	1:11
N–C_a (Å)	1.362(4)	1.364(4)	1.364(5)	1.365(4)	1.364(5)	1.365(5)	1.364(4)	1.365(4)
C_a–C_b (Å)	1.447(13)	1.446(14)	1.445(13)	1.445(14)	1.446(13)	1.445(13)	1.446(13)	1.446(13)
C_a–C_m (Å)	1.396(5)	1.397(6)	1.399(5)	1.401(3)	1.4(6)	1.401(4)	1.4(5)	1.401(3)
C_b–C_b (Å)	1.361(8)	1.36(8)	1.359(8)	1.357(8)	1.359(8)	1.358(8)	1.359(8)	1.358(8)
∠$C_aC_bC_b$ (°)	106.9(9)	107.0(9)	107.0(9)	107.0(9)	107.0(9)	107.0(9)	107.0(9)	107.0(9)
∠NC_aC_b (°)	109(2)	109(2)	109(2)	109(2)	109(2)	109(2)	109(2)	109(2)
∠NC_aC_m (°)	125.8(6)	125.6(9)	125.4(11)	125.2(10)	125.8(13)	125.6(10)	125.8(7)	125.6(14)
∠C_aNC_a (°)	108(3)	107(3)	107(3)	107(3)	107(3)	107(3)	107(3)	107(3)
∠$C_mC_aC_b$ (°)	125(2)	125(2)	125(2)	125(2)	125(2)	125(2)	125(2)	125(2)
∠$C_aC_mC_a$ (°)	126.4(15)	126.7(6)	126.9(14)	127.2(8)	126.3(14)	126.5(12)	126.3(15)	126.6(5)
Δ24 (Å)	0.0125	0.0108	0.0148	0.012	0.0227	0.0282	0.0167	0.0102
ΔN (Å)	0.016(5)	0.012(2)	0.014(3)	0.01(2)	0.02(13)	0.018(13)	0.028(5)	0.017(3)
ΔC_a (Å)	0.006(4)	0.005(4)	0.008(5)	0.007(4)	0.01(8)	0.016(8)	0.011(4)	0.006(3)
ΔC_b (Å)	0.02(12)	0.019(9)	0.026(11)	0.023(8)	0.044(16)	0.056(16)	0.02(5)	0.013(3)
ΔC_m (Å)	0.006(3)	0.004(3)	0.007(3)	0.004(3)	0.007(15)	0.007(3)	0.01(6)	0.006(5)
∠ pyrrole tilt (°)	0.8(6)	0.6(5)	0.8(6)	0.7(4)	1.4(9)	1.6(10)	1.3(2)	0.8(12)
N···N dist (adj) (Å)	2.92(6)	2.92(3)	2.92(5)	2.93(3)	2.92(8)	2.92(2)	2.92(3)	2.92(5)
N···N dist (opp) (Å)	4.13(9)	4.13(8)	4.13(7)	4.14(7)	4.13(8)	4.13(7)	4.13(8)	4.13(7)

Series 3

Table S16: Bond distances, angles, and atom displacement of compounds 3:1-3:3, 2:4.

**Bond Distances, Bond Angles, Atom Displacements**	**3:1**	**3:2**	**2:6**	**3:3**
CCDC code	F	Cl	Br	I
$N–C_a$ (Å)	1.363(10)	1.364(4)	1.365(5)	1.366(6)
$C_a–C_b$ (Å)	1.444(17)	1.445(13)	1.445(13)	1.446(14)
$C_a–C_m$ (Å)	1.398(16)	1.401(3)	1.401(4)	1.402(6)
$C_b–C_b$ (Å)	1.362(12)	1.359(8)	1.358(8)	1.357(8)
$\angle C_aC_bC_b$ (°)	106.8(10)	107.0(9)	107.0(9)	107.1(9)
$\angle NC_aC_b$ (°)	109(2)	109(2)	109(2)	108(2)
$\angle NC_aC_m$ (°)	125.3(12)	125.5(9)	125.6(10)	125.7(10)
$\angle C_aNC_a$ (°)	107(3)	107(3)	107(3)	107(3)
$\angle C_mC_aC_b$ (°)	125(2)	125(2)	125(2)	125(2)
$\angle C_aC_mC_a$ (°)	126(2)	126.6(13)	126.5(12)	126.4(9)
Δ24 (Å)	0.0213	0.0255	0.0282	0.0324
ΔN (Å)	0.022(13)	0.019(13)	0.018(13)	0.016(12)
ΔC_a (Å)	0.01(8)	0.013(8)	0.016(8)	0.019(7)
ΔC_b (Å)	0.04(17)	0.05(16)	0.056(16)	0.066(15)
ΔC_m (Å)	0.006(3)	0.007(2)	0.007(3)	0.007(3)
∠ pyrrole tilt (°)	1.5(7)	1.5(10)	1.6(10)	1.8(9)
N···N dist (adj) (Å)	2.916(10)	2.921(8)	2.923(2)	2.926(5)
N···N dist (opp) (Å)	4.12(8)	4.13(7)	4.13(7)	4.14(7)

Crystal data and refinement

Table S17: Details of XRD data refinement.

Compound	**1**	**2A**	**4**	**5**
Identification code	MS563	MS546	DGIB005A	MS567
Empirical formula	$C_{32}H_{21}BrN_4$	$C_{34}H_{20}N_4Ni$	$C_{40}H_{29}ClN_4$	$C_{38}H_{25}BrN_4$
Formula weight	541.44	543.25	601.12	617.53
Temperature/K	296(2)	100(2)	100(2)	100(2)
Crystal system	monoclinic	monoclinic	monoclinic	triclinic
Space group	I2/c	I2/c	$P2_1/c$	$P\bar{1}$
a/Å	35.1971(15)	29.0968(16)	20.4292(7)	6.3629(3)
b/Å	6.2785(3)	6.3588(2)	10.0496(4)	12.5190(5)
c/Å	27.1730(13)	26.5311(10)	14.7840(5)	18.7822(8)
α/°	90	90	90	101.002(2)
β/°	125.029(2)	102.030(2)	95.238(2)	92.447(2)
γ/°	90	90	90	101.742(2)
Volume/$Å^3$	4917.1(4)	4796.5(3)	3022.56(19)	1432.72(11)
Z	8	8	4	2
D_{calc} g/cm^3	1.463	1.505	1.321	1.431
μ/mm^{-1}	2.503	1.421	1.396	2.223
F(000)	2208.0	2240.0	1256.0	632.0
Crystal size/mm^3	0.09×0.05×0.03	0.50×0.30×0.20	0.16×0.12×0.03	0.30×0.20×0.10
Radiation	CuK_{α}	CuK_{α}	CuK_{α}	CuK_{α}
Wavelength/Å	λ = 1.54178	λ = 1.54178	λ = 1.54178	λ = 1.54178
2ϑ/°	6.694-130.928	6.812-130.986	4.344-136.752	4.810-131.982
Reflections collected	11234	13354	23715	12765
Independent reflections	4045	4000	5526	4822
R_{int}	0.0384	0.1312	0.0782	0.0277
R_{sigma}	0.0452	0.0752	0.0766	0.0357
Restraints	30	0	0	0
Parameters	344	352	408	388
GooF	1.171	1.025	1.039	1.053
R_1 [I> 2σ (I)]	0.0509	0.0505	0.0694	0.0308
wR_2 [I> 2σ (I)]	0.1147	0.1420	0.1892	0.0775
R_1 [all data]	0.0557	0.0577	0.0968	0.0320
wR_2 [all data]	0.1167	0.1473	0.2143	0.0785
Largest peak/e $Å^{-3}$	0.69	0.58	0.65	0.46
Deepest hole/e $Å^{-3}$	-0.73	-1.04	-0.33	-0.38

Table S18: Details of XRD data refinement.

Compound	**6**	**7**	**8**	**9**
Identification code	KJF150xp	dgib007b	tcd662	MS545
Empirical formula	$C_{38}H_{41}BrN_4$	$C_{38}H_{39}BrN_4Ni$	$C_{38}H_{41}IN_4$	$C_{16}H_{10}BrN_2$
Formula weight	633.66	690.35	680.65	310.17
Temperature/K	100(2)	100(2)	100(2)	112(2)
Crystal system	triclinic	triclinic	triclinic	monoclinic
Space group	$P\bar{1}$	$P\bar{1}$	$P\bar{1}$	$P2_1/n$
a/Å	4.8845(6)	4.8416(2)	4.8665(3)	11.2118(7)
b/Å	12.1267(12)	13.9112(6)	24.1992(13)	6.2678(4)
c/Å	26.324(3)	23.8431(10)	27.2069(14)	17.6195(11)
α/°	100.441(3)	106.2510(10)	74.9098(18)	90
β/°	91.101(3)	92.6350(10)	89.9892(19)	100.350(2)
γ/°	90.923(3)	91.003(2)	89.030(2)	90
Volume/Å^3	1532.8(3)	1539.33(11)	3093.1(3)	1218.03(13)
Z	2	2	4	4
D_{calc} *g/cm^3*	1.373	1.489	1.462	1.691
μ/mm^{-1}	1.375	1.963	1.070	4.463
F(000)	664.0	716.0	1400.0	620.0
Crystal size/mm^3	0.45×0.30×0.12	0.28×0.08×0.04	1.00×0.08×0.04	0.30×0.30×0.30
Radiation	MoK_α	MoK_α	MoK_α	CuK_α
Wavelength/Å	λ = 0.71073	λ = 0.71073	λ = 0.71073	λ = 1.54178
2ϑ/°	3.148-51.252	5.304-52.880	5.342-50.500	8.694-133.954
Reflections collected	37076	25173	25863	9061
Independent reflections	5709	6288	11142	2105
R_{int}	0.0972	0.0603	0.0637	0.0279
R_{sigma}	0.0765	0.0529	0.0893	0.0207
Restraints	73	1	39	0
Parameters	454	399	746	172
GooF	1.043	1.058	1.058	1.131
R_1 *[I> 2σ (I)]*	0.0652	0.0460	0.0625	0.0261
wR_2 *[I> 2σ (I)]*	0.1525	0.1066	0.1225	0.0682
R_1 *[all data]*	0.0949	0.0713	0.1005	0.0265
wR_2 *[all data]*	0.1646	0.1215	0.1373	0.0684
Largest peak/e Å^{-3}	0.85	1.16	1.44	0.32
Deepest hole/e Å^{-3}	-0.95	-0.78	-1.76	-0.34

Table S19: Details of XRD data refinement.

Compound	**10**	**11**	**13**	**16A**
Identification code	HE006	HE015	MS556	MS529
Empirical formula	$C_{34}H_{24}Br_2N_4$	$C_{30}H_{30}Br_2N_4Ni$	$C_{35}H_{34}Br_2N_4O_3$	$C_{44}H_{42}N_4Si_2$
Formula weight	648.39	665.11	718.48	682.99
Temperature/K	100(2)	100(2)	100(2)	123(2)
Crystal system	orthorhombic	monoclinic	triclinic	triclinic
Space group	Pbcn	C2/c	$P\bar{1}$	$P\bar{1}$
a/Å	16.6909(12)	44.0507(16)	7.9893(7)	10.955(3)
b/Å	6.2095(4)	10.2192(4)	11.6397(10)	12.739(3)
c/Å	25.1966(17)	11.8461(4)	17.1706(15)	15.334(4)
α/°	90	90	90.536(3)	82.457(5)
β/°	90	91.771(3)	97.989(3)	74.585(5)
γ/°	90	90	107.786(3)	66.493(4)
Volume/Å³	2611.4(3)	5330.1(3)	1503.4(2)	1891.0(8)
Z	4	8	2	2
D_{calc} *g/cm³*	1.649	1.658	1.587	1.200
μ/mm⁻¹	4.191	4.768	3.774	0.130
F(000)	1304.0	2688.0	732.0	724.0
Crystal size/mm³	0.40×0.35×0.30	0.35×0.10×0.05	0.50×0.46×0.10	0.5 × 0.3 × 0.3
Radiation	CuK_{α}	CuK_{α}	CuK_{α}	MoK_{α}
Wavelength/Å	λ = 1.54178	λ = 1.54178	λ = 1.54178	λ = 0.71073
2ϑ/°	7.016-134.196	4.014-133.988	7.988-131.99	3.188-54.998
Reflections collected	12171	18037	17701	17504
Independent reflections	2265	4611	5093	8581
R_{int}	0.0295	0.0810	0.0269	0.0626
R_{sigma}	0.0262	0.0474	0.0299	0.0940
Restraints	0	18	0	0
Parameters	182	348	401	459
GooF	1.058	1.032	1.054	1.251
R_1 *[I> 2σ (I)]*	0.0282	0.0443	0.0253	0.1176
wR_2 *[I> 2σ (I)]*	0.0757	0.1181	0.0655	0.2190
R_1 *[all data]*	0.0295	0.0476	0.0255	0.1507
wR_2 *[all data]*	0.0772	0.1209	0.0657	0.2316
Largest peak/e Å⁻³	0.54	1.17	0.37	0.38
Deepest hole/e Å⁻³	-0.32	-1.53	-0.32	-0.44

Table S20: Details of XRD data refinement.

Compound	**24**
Identification code	HE014
Empirical formula	$C_{43}H_{58}N_4Si$
Formula weight	659.02
Temperature/K	100(2)
Crystal system	triclinic
Space group	$P\bar{1}$
a/Å	10.2392(4)
b/Å	11.2462(5)
c/Å	17.1716(7)
α/°	96.815(2)
β/°	97.845(2)
γ/°	90.696(2)
Volume/Å^3	1944.19(14)
Z	2
D_{calc} *g/cm*^{3}	1.126
μ/mm^{-1}	0.778
F(000)	716.0
Crystal size/mm^3	0.48 × 0.06 × 0.04
Radiation	CuK_α
Wavelength/Å	λ = 1.54178
2ϑ/°	10.478-115.286
Reflections collected	15036
Independent reflections	5308
R_{int}	0.0325
R_{sigma}	0.0403
Restraints	33
Parameters	470
GooF	1.037
R_1 [I> 2σ (I)]	0.0428
wR_2 [I> 2σ (I)]	0.1113
R_1 [all data]	0.0474
wR_2 [all data]	0.1154
Largest peak/e Å^{-3}	0.43
Deepest hole/e Å^{-3}	-0.42

Complete NSD tables

Table S21: NSD out-put of compounds **1**-**23**.

CCDC	COM	Dip	B2g	B1g	Eu(x)	Eu(y)	A1g	A2g	Doop	B2u	B1u	A2u	Eg(x)	Eg(y)	A1u
**MS563**	**1**	0.24	-0.12	0.02	0.03	-0.03	0.20	0.00	0.15	-0.08	0.10	0.05	-0.01	0.06	0.02
**NESHUO**	**2**	0.25	-0.06	0.02	-0.02	0.01	-0.24	-0.01	1.01	0.10	1.00	-0.06	0.03	0.06	0.00
**MS546**	**2A**	0.13	-0.06	0.02	0.02	-0.02	-0.11	-0.01	0.22	-0.06	0.20	0.04	0.01	0.07	0.01
**UDERUR**	**3**	0.29	-0.20	-0.03	-0.05	0.02	0.20	0.00	0.47	0.42	0.13	-0.06	-0.15	0.09	-0.02
**DGIB005**	**4**	0.25	-0.10	-0.03	0.00	0.00	0.23	0.01	0.31	0.05	0.02	-0.05	0.11	0.28	-0.01
**MS567**	**5**	0.21	0.01	0.04	-0.01	-0.01	0.21	-0.01	0.39	0.10	0.31	-0.10	-0.05	0.20	-0.01
**KJF150**	**6**	0.22	0.00	0.01	0.00	0.00	0.22	0.00	0.36	-0.22	-0.08	-0.02	-0.27	-0.06	-0.01
**DGIB007**	**7**	0.14	0.05	-0.01	0.02	0.02	-0.12	0.00	0.36	0.26	0.08	-0.01	0.24	0.00	0.01
**TCD662_1**	**8_1**	0.23	-0.04	0.00	-0.01	0.02	0.23	0.00	0.34	-0.16	0.10	0.05	-0.07	-0.27	0.00
**TCD662_2**	**8_2**	0.22	-0.06	-0.01	-0.01	-0.01	0.21	-0.01	0.37	0.20	-0.05	-0.02	-0.06	-0.30	0.00
**MS545**	**9**	0.25	0.00	-0.06	0.00	0.00	0.24	-0.01	0.08	0.00	0.00	0.00	-0.01	0.08	0.00
**HE006**	**10**	0.24	-0.06	-0.02	0.00	0.00	0.23	-0.01	0.24	0.00	0.00	0.00	0.03	0.23	0.00
**HE015**	**11**	0.49	-0.14	0.01	0.03	0.02	-0.47	-0.01	1.90	0.55	1.81	-0.19	0.07	-0.05	0.00
**NOGWEN**	**12**	0.19	0.02	-0.02	-0.01	-0.01	0.18	-0.01	0.17	0.08	-0.02	-0.02	-0.15	-0.01	0.00
**MS556**	**13**	0.19	0.08	0.02	-0.01	-0.02	0.17	0.00	0.90	0.86	-0.24	-0.03	0.02	-0.10	0.00
**HUMWES**	**13A**	0.19	-0.08	-0.02	-0.02	0.01	0.17	0.00	0.89	0.85	-0.24	0.03	0.10	0.02	0.00
**HUMWAO**	**14**	0.22	-0.05	-0.04	0.01	0.01	0.21	0.00	0.36	0.22	0.01	-0.06	0.26	0.11	-0.02
**LASMOK**	**15**	0.21	-0.02	-0.01	0.00	0.01	0.21	0.00	0.28	0.08	-0.01	-0.25	0.10	0.02	-0.01
**RAKGAN**	**16**	0.21	0.07	-0.01	0.00	0.00	0.20	-0.01	0.89	0.57	-0.67	-0.16	-0.01	-0.07	0.00
**MS529**	**16A**	0.17	0.04	0.01	0.00	0.00	0.16	-0.01	0.67	0.60	-0.21	-0.10	-0.14	0.11	0.01
**ZOXQUA**	**17**	0.03	-0.01	0.00	0.01	0.02	0.01	0.01	0.73	0.23	-0.69	0.02	-0.03	0.05	-0.01
**ZOXCAS**	**18**	0.18	0.01	0.04	-0.01	0.04	0.17	-0.01	0.64	0.27	-0.56	-0.01	0.11	0.05	0.00
**BASDOR**	**19**	0.23	0.00	-0.02	0.02	0.00	0.23	0.00	0.25	0.00	-0.25	0.00	0.00	0.00	0.00
**YISZAD**	**20**	0.26	-0.01	0.06	0.00	0.00	0.26	0.00	0.12	0.00	0.00	0.00	0.08	0.09	0.00
**QUGMEM**	**21**	0.47	0.02	-0.01	0.00	-0.03	-0.46	0.04	1.89	0.34	-1.85	-0.09	0.09	0.00	-0.01
**QUGMIQ**	**22**	0.42	-0.04	0.00	-0.01	-0.01	-0.41	0.00	1.74	0.00	1.72	-0.09	0.14	-0.14	0.00
**MORBEC**	**23**	0.23	-0.05	0.01	-0.01	0.02	-0.22	0.00	0.96	0.23	0.93	-0.02	0.07	-0.01	-0.02
**HE014**	**24**	0.21	-0.07	0.01	0.00	0.00	0.20	0.00	0.29	-0.10	-0.16	-0.01	0.01	0.21	0.00

Table S22: NSD out-put series 1.

CCDC	COM	Dip	B2g	B1g	Eu(x)	Eu(y)	A1g	A2g	Doop	B2u	B1u	A2u	Eg(x)	Eg(y)	A1u
	**1:1**	0.38	-0.32	-0.05	0.00	0.00	0.21	0.00	0.08	0.00	0.00	0.00	0.06	0.06	0.00
	**1:2**	0.14	-0.06	0.00	0.00	0.00	-0.12	0.00	0.57	0.00	0.57	0.01	-0.01	-0.01	0.00
	**1:3**	0.37	-0.31	-0.04	0.00	0.00	0.21	-0.01	0.09	0.00	-0.01	0.00	-0.06	-0.06	0.00
	**1:4**	0.14	-0.07	0.00	0.00	0.00	-0.13	0.00	0.61	0.00	0.61	0.01	-0.02	-0.02	0.00
	**1:5**	0.37	0.31	0.03	0.00	0.00	0.21	-0.01	0.09	0.00	0.00	0.00	-0.06	0.07	0.00
	**1:6**	0.15	-0.08	0.00	0.00	0.00	-0.12	0.00	0.59	0.00	0.59	0.01	-0.02	-0.02	0.00
	**1:7**	0.26	-0.13	-0.04	-0.02	0.02	0.22	0.00	0.08	0.00	-0.01	0.00	0.06	0.05	0.00
	**1:8**	0.19	0.04	0.00	0.02	0.02	-0.18	0.00	0.99	0.00	-0.99	0.01	-0.01	0.01	0.00
	**1:9**	0.23	-0.03	0.02	0.00	0.00	0.23	0.00	0.07	0.00	0.00	0.00	-0.04	0.05	0.00
	**1:10**	0.24	-0.01	0.00	0.00	0.00	-0.24	0.00	1.28	0.00	1.28	0.02	-0.02	-0.02	0.00
	**1D**	0.24	0.09	0.04	-0.02	-0.03	0.22	0.00	0.08	0.00	0.01	0.00	-0.05	0.06	0.00
	**2D**	0.20	-0.03	0.00	0.02	-0.02	-0.20	0.00	1.09	0.00	-1.09	-0.02	0.03	0.03	0.00
	**1:11**	0.26	-0.11	0.02	0.00	0.00	0.23	0.00	0.05	0.00	0.00	0.00	-0.03	0.04	0.00
	**1:12**	0.27	0.00	0.00	0.01	0.00	-0.27	0.00	1.41	0.00	-1.41	0.03	-0.02	0.02	0.00
	**3D**	0.23	-0.02	-0.04	0.03	-0.03	0.23	0.00	0.07	0.00	-0.01	0.00	-0.06	-0.05	0.00
	**1:13**	0.24	-0.02	0.00	-0.02	0.02	-0.24	0.00	1.26	0.00	1.26	0.03	0.00	0.00	0.00
	**1:14**	0.33	-0.22	0.02	0.00	0.00	0.24	0.00	0.02	0.00	0.00	0.00	-0.01	0.01	0.00
	**1:15**	0.31	-0.02	0.00	0.01	0.01	-0.31	0.00	1.56	0.00	-1.55	0.04	-0.02	0.02	0.00

Table S23: NSD out-put series 2.

Series 2		Dip	B2g	B1g	Eu(x)	Eu(y)	A1g	A2g	Doop	B2u	B1u	A2u	Eg(x)	Eg(y)	A1u
	**2:1**	0.28	-0.18	0.06	0.00	-0.01	0.21	0.00	0.07	-0.06	-0.01	0.00	-0.03	-0.03	0.00
	**2:2**	0.24	0.06	0.04	0.02	-0.03	0.22	0.00	0.06	-0.05	0.00	0.00	-0.02	-0.02	0.00
	**2:3**	0.28	-0.14	0.03	0.04	-0.01	0.23	0.00	0.08	-0.07	-0.01	0.00	-0.03	-0.03	0.00
	**2:4**	0.26	0.06	0.02	0.02	0.02	0.25	0.00	0.07	-0.07	0.00	0.00	-0.02	-0.02	0.00
	**2:5**	0.30	0.20	0.03	0.01	-0.02	0.22	0.00	0.14	-0.12	0.00	0.01	0.00	-0.06	0.00
	**2:6**	0.24	0.00	0.02	0.03	0.00	0.24	0.00	0.17	-0.16	0.00	0.01	0.00	-0.06	0.00
	**1D**	0.24	0.09	0.04	-0.02	-0.03	0.22	0.00	0.08	0.00	0.01	0.00	-0.05	0.06	0.00
	**1:11**	0.26	-0.11	0.02	0.00	0.00	0.23	0.00	0.05	0.00	0.00	0.00	-0.03	0.04	0.00

Table S24: NSD out-put series 3.

Series 3		Dip	B2g	B1g	Eu(x)	Eu(y)	A1g	A2g	Doop	B2u	B1u	A2u	Eg(x)	Eg(y)	A1u
	**3:1**	0.21	0.00	0.03	-0.01	0.00	0.21	0.00	0.13	-0.11	0.00	0.02	0.00	-0.06	0.00
	**3:2**	0.23	0.00	0.02	0.02	0.00	0.23	0.00	0.15	-0.14	0.00	0.01	0.00	-0.06	0.00
	**2:6**	0.24	0.00	0.02	0.03	0.00	0.24	0.00	0.17	-0.16	0.00	0.01	0.00	-0.06	0.00
	**3:3**	0.25	0.00	0.02	0.05	0.00	0.25	0.00	0.20	-0.19	0.00	0.01	0.00	-0.05	0.00
